# Supplementary material for: Ketone displacement and migration enabled by trifunctionalization of vinyl triflates
Source: Nat Commun. 2026 Feb 27;17:3294. doi: 10.1038/s41467-026-69513-x (PMC13066064; doi:10.1038/s41467-026-69513-x)
Supplement: Supplementary file 1 — Supplementary Information [file 41467_2026_69513_MOESM1_ESM.pdf]

Supplementary Information

Ketone displacement and migration enabled by trifunctionalization of vinyl triflates

Shiyang Wang[1,2], Tong Yao[1,2], Yu Liu[1,*], Yangyang Li[2,*] & Guoyin Yin[2,*]

[1]College of Chemistry and Life Science, Advanced Institute of Materials Science, Changchun University of Technology, Changchun, Jilin, China

[2]The State Key Laboratory of Metabolism and Regulation in Complex Organisms, The Institute for Advanced Studies, Wuhan University, Wuhan, Hubei, China

*Emails: Prof. Guoyin Yin, yinguoyin@whu.edu.cn, Prof. Yangyang Li, yangyangl@whu.edu.cn, and Prof. Yu Liu, yuliu@ccut.edu.cn.

Table of Contents

Supplementary methods..3

1. General information..3

2. Substrate synthesis..4

3. General procedure..5

4. Analytical data of compounds..6

5. Investigation of mechanism..17

6. Product derivatizations..18

7. Arylative ketone migration..21

8. X-ray structures and data..26

9. NMR spectra..34

Supplementary references..93

Supplementary methods

1. General information

General information: All reactions were run under a dry argon atmosphere fitted on a glass tube or vial. All glassware was oven dried at 120 °C for 2 h and cooled down under vacuum. Thin layer chromatography (TLC) employed glass 0.25 mm silica gel plates. Flash chromatography columns were packed with 200-300 mesh silica gel in petroleum (bp. 60-90 °C). GC analysis was performed on an Agilent 7890B gas chromatograph with an FID detector using a J & W DB-1 column (10 m, 0.1 mm I.D.). The high-resolution mass spectra were measured on Thermo Fisher Scientific Exactive Plus (ESI or APCI). HPLC analysis was conducted on an Agilent 1260 Series instrument. The single crystal X-Ray diffraction was measured on a XtaLAB PRO MM007HF Cu. All new compounds were characterized by ^{1}H NMR, ^{13}C NMR, ^{11}B NMR and ^{19}F NMR data were recorded with JNM-ECZ 400 and Bruker 600 MHz with tetramethylsilane as an internal standard. Data for ^{1}H, ^{13}C, ^{11}B and ^{19}F NMR are reported as follows: chemical shift (δ ppm), multiplicity (s = singlet, d = doublet, t = triplet, q = quartet, dd = doublet of doublet, dt = doublet of triplet, dq = doublet of quartet, m = multiplet), integration, and coupling constant (Hz). All chemical shifts (δ) were reported in ppm and coupling constants (J) in Hz, relative to tetramethylsilane (0 ppm for ^{1}H), Chloroform-d (77.16 ppm for ^{13}C), respectively.

Materials: $NiCl_2$·DME (CAS: 29046-78-4) was purchased from Energy Chemical and stored in the glove box. 2-butyl alcohol (^{s}BuOH), 1,2-Dimethoxyethane (DME), *N*-Methyl-2-pyrrolidone (NMP), 1,2-Dichloroethane (DCE) tert-butyl methyl ether (MTBE) were purchased from Sigma-Aldrich; Some vinyl triflates and aryl bromide were synthesized according to the references. Unless otherwise noted, lithium methoxide (CAS: 865-34-9) and boric acid pinacol ester (CAS: 73183-34-3) were purchased from Adamas beta® and used without further purification.

2. Substrate synthesis

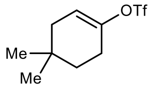

1a

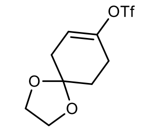

1b

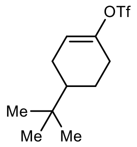

1c

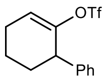

1d

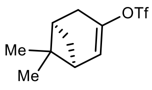

1e

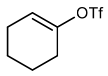

1f

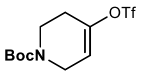

1g

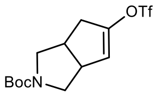

1h

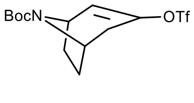

1i

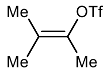

1j

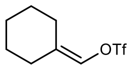

1k

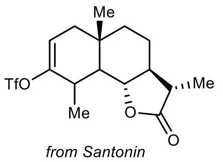

from Santonin

1l

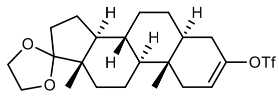

from epiandrosterone derivative

1m

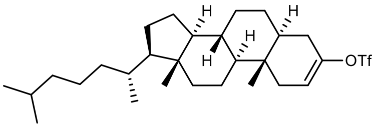

from dihyrocholesterone derivative

1n

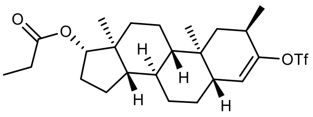

from drostanolone propionate

1o

Alkenyl triflates **1a, 1b, 1c, 1d, 1e, 1f**, **1h**, **1j**, **5k**, **1l**, **1m**, **1n** and **1o** are known compounds and prepared based on the literature[1-3]. Alkenyl triflates **1g** and **1i** were purchased from Adamas beta®.

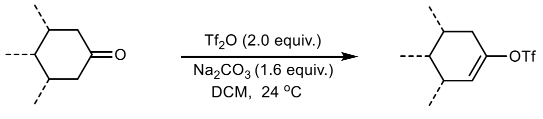

ketone 1

General Procedure S-1: To a solution of ketone (20 mmol, 1.0 equiv.) in dichloromethane (30 mL) was added anhydrous sodium carbonate (3.4 g, 32.0 mmol, 1.6 equiv.). A solution of trifluoromethanesulfonic anhydride (40 mmol, 2.0 equiv.) in DCM (30 mL) was added over a period of 10 minutes. The reaction mixture was stirred for 24 h at room temperature. It was washed with sodium hydrogen carbonate, water and dried with Na_2SO_4, filtered, and the solvent was removed in vacuo. Flash column chromatography provided the vinyl triflate.

Following the general procedure S-1 to produce **1a, 1b, 1c, 1g, 1f, 1j.**

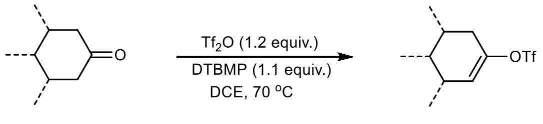

ketone 1

General Procedure S-2: To a solution of ketone (20.0 mmol) in DCM (80 mL) was added 2,6-di-tert-butyl-4-methylpyridine (DTBMP, 22.0 mmol, 1.1 equiv.) at 0 °C. Tf_2O (24.0 mmol, 1.2 equiv.) was added dropwise. The reaction

mixture was then allowed to warm to room temperature, stirred overnight and evaporated to dryness. Petroleum ether was added and the mixture was filtered to remove pyridinium triflate. The petroleum ether solution was washed with cool HCl (1 M), brine, dried over anhydrous Na_2SO_4 and concentrated under reduced pressure. The crude product was purified by flash chromatography on silica gel to give the resulting vinyl triflate.

Following the general procedure S-2 to produce **1k.**

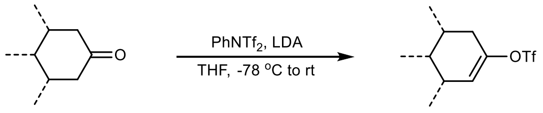

ketone 1

General Procedure S-3: To a dry 3-necked rounL-bottomed flask (100 mL) loaded with 11 mL of LDA (22 mmol, 1.1 equiv). The flask was cooled to -78 °C and stirred for 5 minutes under nitrogen. A solution of ketone (20 mmol, 1.0 equiv) in 10 mL of dry THF was added dropwise to the cooled LDA over a period of 5 minutes. The mixture was stirred for additional 2 h, enabling the complete formation of the enolate. At which point, N-phenyl triflimide (22 mmol, 1.1 equiv) was added in one portion to the above enolate and the suspension was stirred continuously and warmed to room temperature. After additional stirring for 12 h, the reaction mixture was quenched with saturated ammonium chloride solution and extracted with ether. The ether extract was washed with water, 5% sodium bicarbonate solution and dried over anhydrous sodium sulfate. The solvent was removed under reduced pressure and the crude product was purified by column chromatography.

Following the general procedure S-3 to produce **1d, 1e, 1h, 5l, 1m, 1n.1o.**

3. General procedure

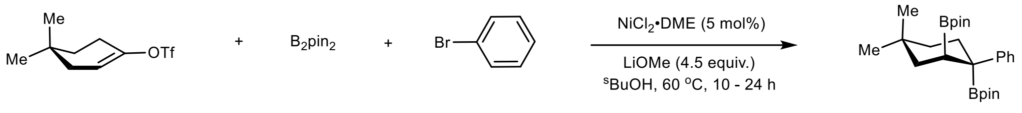

1a 2 3a 4a

General Procedure A: Under a nitrogen atmosphere, an oven-dried 10 mL reaction tube equipped with a magnetic stir bar and sealed with a rubber stopper was used. Sequentially, $NiCl_2{\cdot}DME$ (4.4 mg, 0.02 mmol, 5 mol%), MeOLi (68.3 mg, 1.0 mmol, 4.5 equiv.) and B_2pin_2 (406.2 mg, 1.6 mmol, 4.0 equiv.). Then anhydrous ^sBuOH (1.5 mL), **1a** (0.4 mmol, 1.0 equiv.), **3a** (0.8 mmol, 2.0 equiv.) and anhydrous ^sBuOH (1.0 mL) were added in this order, and the mixture was stirred at 60 °C for 10 - 24 h. Then the mixture was filtered through a silica plug with ethyl acetate and concentrated. The crude material was separated on a silica gel column affording the desired product.

General Procedure B: Under a nitrogen atmosphere, an oven-dried 10 mL reaction tube equipped with a magnetic stir bar and sealed with a rubber stopper was used. Sequentially, $NiCl_2$·DME (4.4 mg, 0.02 mmol, 5 mol%), MeOLi (68.3 mg, 1.0 mmol, 4.5 equiv.) and B_2pin_2 (406.2 mg, 1.6 mmol, 4.0 equiv.). Then anhydrous ^{s}BuOH (1.5 mL), **1a** (0.4 mmol, 1.0 equiv.), **3a** (0.8 mmol, 2.0 equiv.) and anhydrous ^{s}BuOH (1.0 mL) were added in this order, and the mixture was stirred at 60 °C for 10 - 24 h. Then the mixture was filtered through a silica plug with ethyl acetate and concentrated under vacuum. The corresponding alcohols can be obtained by oxidation using H_2O_2 (33 wt%, 2 mL) in a mixture of THF (0.1 M) and NaOH (3.0 M, 2.0 mL), performed overnight at room temperature.

4. Analytical data of compounds

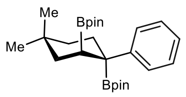

2,2'-(4,4-dimethyl-1-phenylcyclohexane-1,2-diyl)bis(4,4,5,5-tetramethyl-1,3,2-dioxaborolane) (4a): This compound was synthesized according to general procedure **A**. The residue was purified by column chromatography on silica gel to afford the product **4a** (petroleum ether/ethyl acetate = 30:1144, 4 mg, 82% yield) as a white solid. **^{1}H NMR** (600 MHz, Chloroform-*d*) δ 7.34 – 7.29 (m, 2H), 7.19 (dd, *J* = 8.3, 7.2 Hz, 2H), 7.06 – 7.01 (m, 1H), 2.17 – 2.09 (m, 2H), 2.04 (td, *J* = 13.0, 12.3, 3.5 Hz, 1H), 1.69 – 1.61 (m, 2H), 1.47 – 1.37 (m, 1H), 1.26 (td, *J* = 13.4, 12.5, 3.6 Hz, 1H), 1.12 (d, *J* = 23.5 Hz, 12H), 1.01 (s, 6H), 0.92 (d, *J* = 7.2 Hz, 6H), 0.88 – 0.81 (m, 6H) ppm; **^{13}C NMR** (151 MHz, Chloroform-*d*) δ 146.69, 127.36, 127.21, 124.20, 83.04, 82.27, 39.60, 37.96, 34.15, 29.82, 27.05, 24.61, 24.47, 24.32, 24.19 ppm; **^{11}B NMR** (193 MHz, Chloroform-*d*) δ 33.77 ppm; **HRMS (ESI)** calculated $[M+H]^+$ for $C_{26}H_{43}O_4B_2^+$ = 441.3342, found: 441.3352.

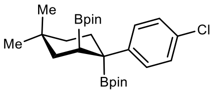

2,2'-(1-(4-chlorophenyl)-4,4-dimethylcyclohexane-1,2-diyl)bis(4,4,5,5-tetramethyl-1,3,2-dioxaborolane) (4b): This compound was synthesized according to general procedure **A**. The residue was purified by column chromatography on silica gel to afford the product **4b** (petroleum ether/ethyl acetate = 30:1, 138.8 mg, 73% yield) as a white solid. **^{1}H NMR** (600 MHz, Chloroform-*d*) δ 7.25 (d, *J* = 8.7 Hz, 2H), 7.17 (d, *J* = 8.6 Hz, 2H), 2.11 – 2.04 (m, 2H), 1.99 (td, *J* = 12.9, 12.1, 3.5 Hz, 1H), 1.64 – 1.58 (m, 2H), 1.38 (d, *J* = 13.2 Hz, 1H), 1.27 – 1.23 (m, 1H), 1.15 (s, 6H), 1.11 (s, 6H), 1.03 (s, 6H), 0.93 – 0.85 (m, 12H) ppm; **^{13}C NMR** (151 MHz, Chloroform-*d*) δ 145.45, 129.89, 128.73, 127.36, 83.21, 82.46, 39.42, 37.69, 33.86, 29.78, 27.25, 24.66, 24.50, 24.35, 24.22 ppm; **^{11}B NMR** (193 MHz, Chloroform-*d*) δ 34.29 ppm; **HRMS (ESI)** calculated $[M+H]^+$ for $C_{26}H_{42}O_4B_2Cl^+$ = 475.2952, found: 475.2968.

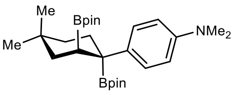

4-(4,4-dimethyl-1,2-bis(4,4,5,5-tetramethyl-1,3,2-dioxaborolan-2-yl)cyclohexyl)-N,N-dimethylaniline (4c): This compound was synthesized according to general procedure **A**. The residue was purified by column chromatography on silica gel to afford the product **4c** (petroleum ether/ethyl acetate = 5:1, 156 mg, 80 % yield) as a white solid. **^{1}H NMR** (600 MHz, Chloroform-*d*) δ 7.17 (d, *J* = 8.8 Hz, 2H), 6.66 (d, *J* = 8.8 Hz, 2H), 2.86 (s, 6H), 2.13 – 1.93 (m, 3H), 1.66 – 1.55 (m, 2H), 1.39 (d, *J* = 13.3 Hz, 1H), 1.26 (d, *J* = 2.9 Hz, 1H), 1.15 (s, 6H), 1.11 (s, 6H), 1.03 (s, 6H), 0.90 (t, *J* = 5.3 Hz, 12H) ppm; **^{13}C NMR** (151 MHz, Chloroform-*d*) δ 148.09, 135.29, 127.78, 112.94, 82.87, 82.20, 41.26, 39.39, 37.84, 32.86, 29.84, 27.42, 24.72, 24.58, 24.37, 24.26 ppm; **^{11}B NMR** (193 MHz, Chloroform-*d*) δ 34.60 ppm. **HRMS (ESI)** calculated $[M+H]^+$ for $C_{28}H_{48}O_4NB_2^+$ = 484.3764, found: 484.3776.

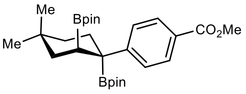

methyl 4-(4,4-dimethyl-1,2-bis(4,4,5,5-tetramethyl-1,3,2-dioxaborolan-2-yl)cyclohexyl)benzoate (4d): This compound was synthesized according to general procedure **A**. The residue was purified by column chromatography on silica gel to afford the product **4d** (petroleum ether/ethyl acetate = 5:1, 99 mg, 49% yield) as a colorless transparent solid. **^{1}H NMR** (600 MHz, Chloroform-*d*) δ 7.89 (d, *J* = 8.5 Hz, 2H), 7.40 (d, *J* = 8.5 Hz, 2H), 3.88 (s, 3H), 2.14 (dt, *J* = 12.7, 4.5 Hz, 2H), 2.05 (ddd, *J* = 12.8, 10.0, 3.5 Hz, 1H), 1.69 – 1.61 (m, 2H), 1.43 – 1.33 (m, 1H), 1.29 – 1.24 (m, 1H), 1.14 (s, 6H), 1.10 (s, 6H), 1.02 (s, 6H), 0.94 – 0.80 (m, 12H) ppm; **^{13}C NMR** (151 MHz, Chloroform-*d*) δ 167.58, 153.04, 128.72, 127.28, 126.01, 83.29, 82.50, 51.74, 39.48, 37.74, 35.07, 29.81, 27.20, 24.67, 24.44, 24.32, 24.21 ppm; **^{11}B NMR** (193 MHz, Chloroform-*d*) δ 34.29 ppm; **HRMS (ESI)** calculated $[M+H]^+$ for $C_{28}H_{45}B_2O_6^+$ = 499.3396, found: 499.3415.

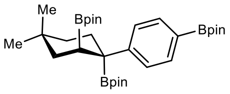

2,2'-(4,4-dimethyl-1-(4-(4,4,5,5-tetramethyl-1,2,3l2-oxadiborolan-2-yl)phenyl)cyclohexane-1,2-diyl)bis(4,4,5,5-tetramethyl-1,3,2-dioxaborolane) (4e): This compound was synthesized according to general procedure **A**. The residue was purified by column chromatography on silica gel to afford the product **4e** (petroleum ether/ethyl acetate = 5:1, 158.5 mg, 70% yield) as a white solid. **^{1}H NMR** (600 MHz, Chloroform-*d*) δ 7.64 (d, *J* = 8.2 Hz, 2H), 7.34 – 7.29 (m, 2H), 2.14 – 2.01 (m, 3H), 1.66 – 1.56 (m, 3H), 1.35 (s, 1H), 1.33 (d, *J* = 2.1 Hz, 12H), 1.13 (d, *J* = 19.4 Hz, 12H), 1.04 (s, 6H), 0.96 – 0.83 (m, 12H) ppm; **^{13}C NMR** (151 MHz, Chloroform-*d*) δ 150.15, 133.94, 133.86, 126.98, 83.37, 83.10, 82.47, 29.89, 24.92, 24.86, 24.80, 24.68, 24.55,

24.39, 24.28 ppm; **^{11}B NMR** (193 MHz, Chloroform-*d*) δ 34.69, 24.53 ppm; **HRMS (ESI)** calculated $[M+H]^+$ for $C_{32}H_{54}B_3O_6^+$ = 567.4194, found: 567.4202.

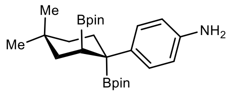

4-(4,4-dimethyl-1,2-bis(4,4,5,5-tetramethyl-1,3,2-dioxaborolan-2-yl)cyclohexyl)aniline (4f): This compound was synthesized according to general procedure **A**. The residue was purified by column chromatography on silica gel to afford the product **4f** (petroleum ether/ethyl acetate = 2:1, 122 mg, 67% yield) as a pale yellow crystalline solid. **^{1}H NMR** (600 MHz, Chloroform-*d*) δ 7.10 (d, *J* = 8.5 Hz, 2H), 6.59 (d, *J* = 8.5 Hz, 2H), 3.22 (s, 2H), 2.10 – 1.93 (m, 3H), 1.66 – 1.58 (m, 2H), 1.40 (d, *J* = 13.3 Hz, 1H), 1.27 – 1.21 (m, 1H), 1.15 (s, 6H), 1.11 (s, 6H), 1.04 (s, 6H), 0.96 – 0.86 (m, 12H) ppm; **^{13}C NMR** (151 MHz, Chloroform-*d*) δ 142.68, 136.91, 128.00, 114.79, 82.90, 82.26, 39.45, 37.88, 29.83, 24.71, 24.52, 24.36, 24.24 ppm; **^{11}B NMR** (193 MHz, Chloroform-*d*) δ 33.66 ppm; **HRMS (ESI)** calculated $[M+H]^+$ for $C_{26}H_{44}B_2N_1O_4^+$ = 456.3451, found: 456.3460.

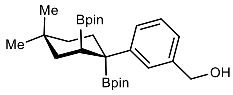

(3-(4,4-dimethyl-1,2-bis(4,4,5,5-tetramethyl-1,3,2-dioxaborolan-2-yl)cyclohexyl)phenyl)methano (4g): This compound was synthesized according to general procedure **A**. The residue was purified by column chromatography on silica gel to afford the product **4g** (petroleum ether/ethyl acetate = 3:1, 133.6 mg, 71% yield) as a colorless oil; **^{1}H NMR** (600 MHz, Chloroform-*d*) δ 7.30 (d, *J* = 1.8 Hz, 1H), 7.26 – 7.24 (m, 1H), 7.20 (t, *J* = 7.6 Hz, 1H), 7.08 (dt, *J* = 7.5, 1.5 Hz, 1H), 4.63 (s, 2H), 2.15 – 2.03 (m, 3H), 1.70 – 1.60 (m, 4H), 1.35 (s, 1H), 1.15 (s, 6H), 1.12 (s, 6H), 1.01 (s, 6H), 0.91 (s, 6H), 0.86 (s, 6H) ppm; **^{13}C NMR** (151 MHz, Chloroform-*d*) δ 170.91, 147.35, 134.59, 127.62, 127.46, 124.41, 83.18, 82.31, 67.03, 39.74, 38.02, 29.86, 26.93 24.63, 24.58, 24.30, 24.22, 21.04 ppm; **^{11}B NMR** (193 MHz, Chloroform-*d*) δ 34.13 ppm; **HRMS (ESI)** calculated $[M+H]^+$ for $C_{27}H_{45}B_2O_5^+$ = 471.3447.

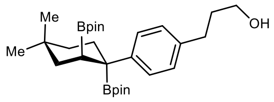

3-(4-(4,4-dimethyl-1,2-bis(4,4,5,5-tetramethyl-1,3,2-dioxaborolan-2-yl)cyclohexyl)phenyl)propan-1-ol (4h): This compound was synthesized according to general procedure **A**. The residue was purified by column chromatography on silica gel to afford the product **4h** (petroleum ether/ethyl acetate = 3:1, 119.6 mg, 60% yield) as a colorless oil. **^{1}H NMR** (600 MHz, Chloroform-*d*) δ 7.25 – 7.15 (m, 2H), 7.05 – 6.98 (m, 2H), 3.65 (t, *J* = 6.5 Hz, 2H), 2.62 (dd, *J* = 8.5, 6.8 Hz, 2H), 2.17 – 1.98 (m, 3H), 1.89 – 1.76 (m, 2H), 1.72 – 1.56 (m, 2H), 1.42 (d, *J* = 13.5 Hz, 1H), 1.32 (s, 1H), 1.29 – 1.23 (m, 1H), 1.14 (s, 6H), 1.10 (s, 6H),

1.01 (s, 6H), 0.91 (d, *J* = 12.7 Hz, 6H), 0.84 (s, 6H) ppm; **^{13}C NMR** (151 MHz, Chloroform-*d*) δ 144.24, 137.38, 127.46, 127.17, 83.02, 82.25, 62.45, 39.71, 38.09, 34.50, 33.78, 31.57, 29.83, 26.93, 24.63, 24.50, 24.32, 24.19 ppm; **^{11}B NMR (193 MHz, Chloroform-*d*)** δ 33.48 ppm; **HRMS (ESI)** calculated $[M+H]^+$ for $C_{29}H_{49}O_5B_2^+$ = 499.3760, found: 499.3779.

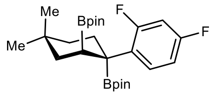

2,2'-(1-(2,4-difluorophenyl)-4,4-dimethylcyclohexane-1,2-diyl)bis(4,4,5,5-tetramethyl-1,3,2-dioxaborolane) (4i): This compound was synthesized according to general procedure **A**. The residue was purified by column chromatography on silica gel to afford the product **4i** (petroleum ether/ethyl acetate = 30:1, 122 mg, 64% yield) as a white solid. **^{1}H NMR** (600 MHz, Chloroform-*d*) δ 7.35 (td, *J* = 9.0, 6.6 Hz, 1H), 6.78 – 6.71 (m, 1H), 6.66 (ddd, *J* = 11.8, 8.9, 2.7 Hz, 1H), 2.15 (ddd, *J* = 13.0, 8.4, 4.3 Hz, 1H), 2.07 – 1.90 (m, 2H), 1.81 – 1.71 (m, 1H), 1.62 – 1.56 (m, 1H), 1.20 (d, *J* = 16.5 Hz, 14H), 1.06 (s, 6H), 1.00 (s, 6H), 0.93 (s, 3H), 0.89 (s, 3H) ppm; **^{13}C NMR** (151 MHz, Chloroform-*d*) δ 161.79 (dd, J = 74.0, 11.9 Hz), 160.16 (dd, J = 70.1, 12.0 Hz), 130.54 (t, *J* = 8.4 Hz), 129.67, 109.63 (dd, *J* = 20.0, 3.3 Hz), 103.35 (dd, *J* = 28.3, 24.5 Hz), 83.19, 82.60, 37.73, 36.75, 29.77, 27.98, 24.68, 24.62, 24.56, 24.46 ppm; **^{11}B NMR** (193 MHz, Chloroform-*d*) δ 33.75 ppm; **^{19}F NMR** (565 MHz, Chloroform-*d*) δ -116.70 ppm; **HRMS (ESI)** calculated $[M+H]^+$ for $C_{26}H_{41}O_4B_2F_2^+$ = 477.3153, found: 477.3167.

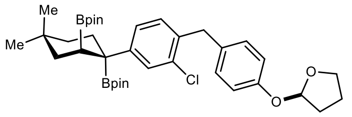

2,2'-(1-(4-chloro-3-(4-((tetrahydrofuran-3-yl)oxy)benzyl)phenyl)-4,4-dimethylcyclohexane-1,2-diyl)bis(4,4,5,5-tetramethyl-1,3,2-dioxaborolane) (4j): This compound was synthesized according to general procedure **A**. The residue was purified by column chromatography on silica gel to afford the product **4j** (petroleum ether/ethyl acetate = 5:1, 170 mg, 65% yield) as a white solid. **^{1}H NMR** (600 MHz, Chloroform-*d*) δ 7.22 – 7.17 (m, 2H), 7.12 (dd, *J* = 8.6, 2.0 Hz, 3H), 6.77 – 6.72 (m, 2H), 4.89 – 4.85 (m, 1H), 4.04 – 3.93 (m, 5H), 3.90 – 3.86 (m, 1H), 2.19 – 2.11 (m, 2H), 2.07 – 2.02 (m, 2H), 1.99 – 1.91 (m, 1H), 1.61 (d, *J* = 5.4 Hz, 2H), 1.36 (d, *J* = 14.1 Hz, 1H), 1.20 (q, *J* = 7.8, 6.2 Hz, 1H), 1.10 (d, *J* = 15.4 Hz, 12H), 1.01 (s, 6H), 0.92 – 0.78 (m, 12H). ppm; **^{13}C NMR** (151 MHz, Chloroform-*d*) δ 155.57, 145.78, 137.15, 132.73, 130.35, 129.87, 129.79, 128.37, 126.79, 115.12, 83.17, 82.42, 73.11, 67.15, 38.54, 38.27, 37.66, 33.83, 32.96, 29.79, 27.28, 24.77, 24.68, 24.45, 24.37, 24.23 ppm; **^{1}B NMR** (193 MHz, Chloroform-*d*) δ 35.41 ppm; **HRMS (ESI)** calculated $[M+H]^+$ for $C_{37}H_{54}O_6B_2Cl^+$ = 651.3789, found: 651.3801.

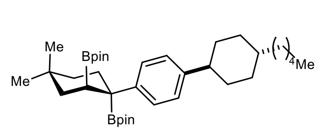

2,2'-(4,4-dimethyl-1-(4-(4-pentylcyclohexyl)phenyl)cyclohexane-1,2-diyl)bis(4,4,5,5-tetramethyl-1,3,2-dioxaborolane) (4k): This compound was synthesied according to general procedure **A**. The residue was purified by column chromatography on silica gel to afford the product **4k** (petroleum ether/ethyl acetate = 30:1, 162 mg, 68% yield) as a white solid. **^{1}H NMR** (600 MHz, Chloroform-*d*) δ 7.23 – 7.18 (m, 2H), 7.02 (d, *J* = 8.3 Hz, 2H), 2.37 (tt, *J* = 12.1, 3.2 Hz, 1H), 2.13 (t, *J* = 5.0 Hz, 1H), 2.10 – 2.00 (m, 2H), 1.86 – 1.78 (m, 4H), 1.68 (dd, *J* = 13.7, 6.4 Hz, 1H), 1.63 – 1.57 (m, 2H), 1.49 – 1.35 (m, 3H), 1.34 – 1.17 (m, 11H), 1.12 (d, *J* = 21.4 Hz, 12H), 0.98 (s, 6H), 0.93 (s, 3H), 0.91 – 0.86 (m, 6H), 0.80 (s, 6H) ppm; **^{13}C NMR** (151 MHz, Chloroform-*d*) δ 144.08, 143.67, 126.84, 125.85, 83.01, 82.16, 44.12, 39.93, 38.32, 37.43, 37.35, 34.52, 34.46, 33.73, 33.71, 32.20, 29.82, 26.65, 24.62, 24.57, 24.30, 24.14, 22.71, 14.11 ppm; **^{11}B NMR** (193 MHz, Chloroform-*d*) δ 35.62 ppm; **HRMS (ESI)** calculated $[M+H]^+$ for $C_{37}H_{63}O_4B_2^+$ = 593.4907, found: 593.4921.

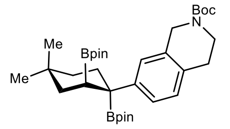

tert-butyl 7-(4,4-dimethyl-1,2-bis(4,4,5,5-tetramethyl-1,3,2-dioxaborolan-2-yl)cyclohexyl)-3,4-dihydroisoquinoline-2(1H)-carboxylate (4l): This compound was synthesized according to general procedure **A**. The residue was purified by column chromatography on silica gel to afford the product **4l** (petroleum ether/ethyl acetate = 5:1, 123.9 mg, 52% yield) as a white solid. **^{1}H NMR** (600 MHz, Chloroform-*d*) δ 7.11 (dd, *J* = 8.1, 2.0 Hz, 1H), 7.03 (s, 1H), 6.96 (d, *J* = 8.0 Hz, 1H), 4.51 (d, *J* = 5.0 Hz, 2H), 3.60 (s, 2H), 2.81 – 2.67 (m, 2H), 2.10 – 1.99 (m, 3H), 1.73 (s, 1H), 1.61 (qd, *J* = 8.8, 8.0, 3.1 Hz, 2H), 1.49 (s, 9H), 1.40 – 1.33 (m, 1H), 1.15 (d, *J* = 14.5 Hz, 12H), 1.02 (s, 6H), 0.94 – 0.84 (m, 12H) ppm; **^{13}C NMR** (151 MHz, Chloroform-*d*) δ 155.04, 144.54, 130.46, 127.73, 125.72, 83.14, 82.36, 79.50, 46.33, 45.54, 41.00, 38,98, 37.92, 33.71, 29.87, 28.52, 24.66, 24.34, 24.30 ppm; **^{11}B NMR** (193 MHz, Chloroform-*d*) δ 33.25 ppm.

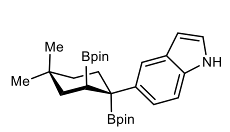

5-(4,4-dimethyl-1,2-bis(4,4,5,5-tetramethyl-1,3,2-dioxaborolan-2-yl)cyclohexyl)-1H-indole (4m): This compound was synthesized according to general procedure **A**. The residue was purified by column chromatography on silica gel to afford the product **4m** (petroleum ether/ethyl acetate = 2:1, 155 mg, 80% yield) as a light yellow solid. **^{1}H NMR** (600 MHz, Chloroform-*d*) δ 8.02 (s, 1H), 7.45 (d, *J* = 8.3 Hz, 1H), 7.37 – 7.33 (m, 1H), 7.14 – 7.07 (m, 2H), 6.42 (ddd, *J* = 3.1, 2.0, 0.9 Hz, 1H), 2.25 – 2.20 (m, 1H), 2.18 (dddd, *J* = 13.1, 5.3, 3.7, 1.7 Hz, 1H), 2.11 (td, *J* = 12.2, 3.4 Hz, 1H), 1.71 (dd, *J* = 13.4, 6.2 Hz, 1H), 1.65 (ddd, *J* = 13.6, 4.5, 1.8 Hz, 1H), 1.50 – 1.41 (m, 1H), 1.30 (dd, *J* = 12.2, 3.7 Hz, 1H), 1.14 (s, 6H), 1.10 (s, 6H), 0.97 – 0.91 (m, 12H), 0.74 (s, 6H) ppm; **^{13}C NMR** (151 MHz,

Chloroform-*d*) δ 140.99, 136.41, 124.88, 122.98, 120.13, 119.31, 109.29, 101.81, 83.01, 82.18, 39.75, 38.18, 29.90, 27.19, 25.00, 24.59, 24.57, 24.37, 24.07 ppm; **^{11}B NMR** (193 MHz, Chloroform-*d*) δ 35.40 ppm; **HRMS (ESI)** calculated $[M+H]^+$ for $C_{28}H_{44}O_4NB_2^+$ = 480.3451, found: 480.3470.

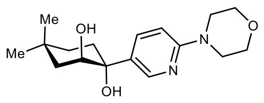

4,4-dimethyl-1-(6-morpholinopyridin-3-yl)cyclohexane-1,2-dio (4n): This compound was synthesized according to general procedure **B**. The residue was purified by column chromatography on silica gel to afford the product **4n** (petroleum ether/ethyl acetate = 1:1, 38 mg, 31% yield) as a white solid. **^{1}H NMR** (600 MHz, Chloroform-*d*) δ 8.44 (d, *J* = 2.5 Hz, 1H), 7.82 (dd, *J* = 9.0, 2.6 Hz, 1H), 6.62 (d, *J* = 8.6 Hz, 1H), 3.91 (dd, *J* = 7.3, 4.0 Hz, 1H), 3.81 (t, *J* = 4.8 Hz, 4H), 3.49 (t, *J* = 4.9 Hz, 4H), 2.38 (ddd, *J* = 13.5, 8.9, 3.7 Hz, 1H), 2.24 – 1.89 (m, 2H), 1.77 (dd, *J* = 13.9, 4.1 Hz, 1H), 1.68 (ddd, *J* = 13.2, 8.2, 3.6 Hz, 1H), 1.56 (dt, *J* = 13.1, 6.5 Hz, 2H), 1.32 (ddd, *J* = 13.0, 8.2, 3.6 Hz, 1H), 1.02 (d, *J* = 17.7 Hz, 6H).ppm; **^{13}C NMR** (151 MHz, Chloroform-*d*) δ 158.67, 146.73, 137.03, 129.05, 106.39, 74.77, 74.48, 66.70, 45.52, 42.21, 35.01, 30.34, 29.56, 24.81 ppm; **HRMS (ESI)** calculated $[M+H]^+$ for $C_{17}H_{27}N_2O_3^+$ = 307.2016, found: 307.2016.

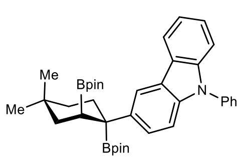

3-(4,4-dimethyl-1,2-bis(4,4,5,5-tetramethyl-1,3,2-dioxaborolan-2-yl)cyclohexyl)-9-phenyl-9H-carbazole (4o): This compound was synthesized according to general procedure **A**. The residue was purified by column chromatography on silica gel to afford the product **4o** (petroleum ether/ethyl acetate = 20:1, 190 mg, 78% yield) as a white solid. **^{1}H NMR** (600 MHz, Chloroform-*d*) δ 8.13 – 8.08 (m, 2H), 7.58 – 7.51 (m, 4H), 7.45 – 7.36 (m, 3H), 7.34 (ddd, *J* = 8.2, 6.9, 1.2 Hz, 1H), 7.29 (d, *J* = 8.6 Hz, 1H), 7.26 – 7.20 (m, 1H), 2.35 – 2.13 (m, 3H), 1.74 (d, *J* = 5.4 Hz, 2H), 1.53 – 1.43 (m, 1H), 1.37 – 1.28 (m, 1H), 1.13 (d, *J* = 25.6 Hz, 12H), 0.97 (d, *J* = 9.0 Hz, 12H), 0.81 (s, 6H) ppm; **^{13}C NMR** (151 MHz, Chloroform-*d*) δ 140.76, 138.56, 138.35, 138.16, 129.65, 126.83, 126.72, 126.12, 125.10, 123.95, 122.86, 120.12, 119.39, 118.70, 109.42, 108.66, 83.02, 82.28, 39.49, 37.93, 33.89, 29.91, 25.65, 24.67, 24.59, 24.35, 24.19 ppm; **^{11}B NMR** (193 MHz, Chloroform-*d*) δ 33.94 ppm; **HRMS (ESI)** calculated $[M+H]^+$ for $C_{38}H_{50}O_4NB_2^+$ = 606.3920, found: 606.3927.

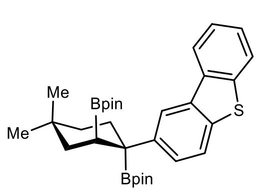

2,2'-(1-(dibenzo[b,d]thiophen-2-yl)-4,4-dimethylcyclohexane-1,2-diyl)bis(4,4,5,5-tetramethyl-1,3,2-dioxaborolane (4p): This compound was synthesized according to general procedure **A**. The residue was purified by column chromatography on silica gel to afford the

product **4p** (petroleum ether/ethyl acetate = 20:1, 127 mg, 58% yield) as a colorless solid. **^{1}H NMR** (600 MHz, Chloroform-*d*) δ 8.21 – 8.12 (m, 2H), 7.84 – 7.77 (m, 1H), 7.69 (d, *J* = 8.5 Hz, 1H), 7.49 (dd, *J* = 8.5, 1.9 Hz, 1H), 7.44 – 7.36 (m, 2H), 2.32 – 2.10 (m, 3H), 1.73 (d, *J* = 5.2 Hz, 2H), 1.46 (d, *J* = 13.0 Hz, 1H), 1.37 – 1.28 (m, 1H), 1.13 (d, *J* = 25.9 Hz, 12H), 0.95 (d, *J* = 3.3 Hz, 12H), 0.77 (s, 6H) ppm; **^{13}C NMR** (151 MHz, Chloroform-*d*) δ 143.44, 139.64, 136.11, 135.38, 135.07, 127.00, 126.03, 123.97, 122.70, 121.54, 121.43, 120.22, 83.19, 82.38, 39.58, 37.88, 34.19, 29.88, 24.89, 24.82, 24.58, 24.32, 24.18 ppm; **^{11}B NMR** (193 MHz, Chloroform-*d*) δ 35.45 ppm; **HRMS (ESI)** calculated $[M+H]^+$ for $C_{32}H_{45}O_4B_2S^+$ = 547.3219, found: 547.3227.

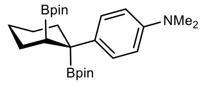

4-(1,2-bis(4,4,5,5-tetramethyl-1,3,2-dioxaborolan-2-yl)cyclohexyl)-N,N-dimethylaniline (5a): This compound was synthesized according to general procedure **A**. The residue was purified by column chromatography on silica gel to afford the product **5a** (petroleum ether/ethyl acetate = 50:1, 160 mg, 87% yield) as a white solid. **^{1}H NMR** (600 MHz, Chloroform-*d*) δ 7.19 (d, *J* = 8.9 Hz, 2H), 6.66 (d, *J* = 8.9 Hz, 2H), 2.84 (s, 6H), 2.24 – 2.11 (m, 2H), 1.98 (td, *J* = 12.5, 3.5 Hz, 1H), 1.89 – 1.81 (m, 1H), 1.79 – 1.73 (m, 1H), 1.66 (tdd, *J* = 13.3, 5.3, 3.6 Hz, 1H), 1.52 (dt, *J* = 11.9, 3.4 Hz, 1H), 1.43 – 1.29 (m, 2H), 1.12 (d, *J* = 16.8 Hz, 12H), 0.92 (d, *J* = 20.3 Hz, 12H) ppm; **^{13}C NMR** (151 MHz, Chloroform-*d*) δ 148.34, 135.82, 127.20, 113.09, 82.84, 81.91, 41.22, 29.32, 27.25, 26.02, 24.62, 24.48, 24.30, 23.85 ppm; **^{11}B NMR** (193 MHz, Chloroform-*d*) δ 32.69 ppm; **HRMS (ESI)** calculated $[M+H]^+$ for $C_{26}H_{44}B_2N_1O_4^+$ = 456.3451, found: 456.3463.

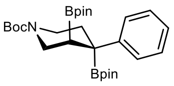

tert-butyl 4-phenyl-3,4-bis(4,4,5,5-tetramethyl-1,3,2-dioxaborolan-2-yl)piperidine-1-carboxylate (5b): This compound was synthesized according to general procedure **A**. The residue was purified by column chromatography on silica gel to afford the product **5b** (petroleum ether/ethyl acetate = 5:1, 119.1 mg, 58% yield) as a white solid. **^{1}H NMR** (600 MHz, Chloroform-*d*) δ 7.30 (d, *J* = 7.8 Hz, 2H), 7.22 (t, *J* = 7.8 Hz, 2H), 7.10 – 7.05 (m, 1H), 4.38 – 4.13 (m, 2H), 3.05 (dd, *J* = 53.0, 13.6 Hz, 1H), 2.89 – 2.69 (m, 1H), 2.29 – 2.13 (m, 3H), 1.45 (s, 9H), 1.12 (s, 6H), 1.10 (s, 6H), 0.93 (s, 6H), 0.83 (s, 6H) ppm; **^{13}C NMR** (151 MHz, Chloroform-*d*) δ 154.13, 145.39, 127.81, 126.57, 125.01, 83.47, 82.43, 78.57, 60.37, 45.04, 44.09, 42.83, 28.52, 24.61, 24.32, 24.00 ppm; **^{11}B NMR** (193 MHz, Chloroform-*d*) δ 33.43 ppm; **HRMS (ESI)** calculated $[M+NH_4]^+$ for $C_{28}H_{46}O_6NB_2^+$ = 514.3505, found: 514.3519.

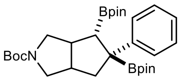

tert-butyl 5-phenyl-4,5-bis(4,4,5,5-tetramethyl-1,3,2-dioxaborolan-2-yl)hexahydrocyclopenta[c]pyrrole-2(1H)-carboxylate (5c): his compound was synthesized according to general procedure **A**. The residue was purified by column chromatography on silica gel to afford the product **5c** (petroleum ether/ethyl acetate = 5:1, 138.1 mg, 64% yield) as a white solid. **^{1}H NMR** (600 MHz, Chloroform-*d*) δ 7.25 (d, *J* = 8.2 Hz, 2H), 7.21 – 7.17 (m, 2H), 7.10 – 7.05 (m, 1H), 3.69 – 3.52 (m, 2H), 3.33 – 3.14 (m, 2H), 2.81 (ddt, *J* = 24.7, 9.7, 5.0 Hz, 2H), 2.39 (dd, *J* = 12.7, 8.2 Hz, 1H), 2.04 (dt, *J* = 30.5, 8.7 Hz, 2H), 1.45 (d, *J* = 1.4 Hz, 9H), 1.14 (d, *J* = 6.9 Hz, 12H), 1.04 (s, 6H), 0.98 (s, 6H) ppm; **^{13}C NMR** (151 MHz, Chloroform-*d*) δ 154.50, 145.66, 128.11, 127.46, 124.92, 83.51, 82.72, 78.72, 52.77, 52.44, 52.00, 45.41, 43.06, 42.09, 28.48, 24.62, 24.42, 24.27 ppm; **^{11}B NMR** (193 MHz, Chloroform-*d*) δ 234.35 ppm; **HRMS (ESI)** calculated $[M+H]^+$ for $C_{30}H_{48}O_6NB_2$ = 540.3662, found: 540.3672.

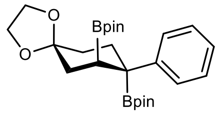

2,2'-(8-phenyl-1,4-dioxaspiro[4.5]decane-7,8-diyl)bis(4,4,5,5-tetramethyl-1,3,2-dioxaborolane) (5d): This compound was synthesized according to general procedure **A**. The residue was purified by column chromatography on silica gel to afford the product **5d** (petroleum ether/ethyl acetate = 5:1, 142 mg, 75% yield) as a white solid. **^{1}H NMR** (600 MHz, Chloroform-*d*) δ 7.35 – 7.27 (m, 2H), 7.19 (t, *J* = 7.9 Hz, 2H), 7.04 (td, *J* = 7.2, 1.2 Hz, 1H), 4.01 – 3.86 (m, 4H), 2.39 – 2.21 (m, 3H), 2.07 – 1.99 (m, 1H), 1.92 – 1.70 (m, 3H), 1.11 (d, *J* = 16.5 Hz, 12H), 1.03 (s, 6H), 0.85 (s, 6H) ppm; **^{13}C NMR** (151 MHz, Chloroform-*d*) δ 145.81, 127.29, 126.91, 124.25, 108.99, 83.29, 82.14, 64.19, 35.70, 34.49, 27.82, 26.93, 24.53, 24.42, 24.23, 24.12.ppm; **^{11}B NMR** (193 MHz, Chloroform-*d*) δ 34.51 ppm; **HRMS (ESI)** calculated $[M+H]^+$ for $C_{26}H_{41}O_6B_2^+$ = 471.3083, found: 471.3101.

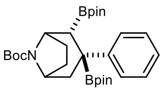

tert-butyl 3-phenyl-2,3-bis(4,4,5,5-tetramethyl-1,3,2-dioxaborolan-2-yl)-8-azabicyclo[3.2.1]octane-8-carboxylate (5e): This compound was synthesized according to general procedure **A**. The residue was purified by column chromatography on silica gel to afford the product **5e** (petroleum ether/ethyl acetate = 5:1, 120.8 mg, 56% yield) as a white solid. **^{1}H NMR** (600 MHz, Chloroform-*d*) δ 7.28 – 7.25 (m, 2H), 7.16 (t, *J* = 7.8 Hz, 2H), 7.02 (td, *J* = 7.2, 1.2 Hz, 1H), 4.49 (d, *J* = 38.9 Hz, 2H), 3.02 (dd, *J* = 9.6, 1.8 Hz, 1H), 2.89 (s, 1H), 1.87 (td, *J* = 14.0, 12.5, 4.1 Hz, 2H), 1.71 (d, *J* = 8.1 Hz, 2H), 1.60 (d, *J* = 8.0 Hz, 1H), 1.43 (s, 9H), 1.04 (dd, *J* = 26.2, 6.6 Hz, 12H), 0.96 (s, 6H), 0.82 (s, 6H) ppm; **^{13}C NMR** ((151 MHz, Chloroform-*d*) δ 157.03, 145.32, 127.43,

127.26, 124.44, 83.02, 82.50, 79.21, 54.57, 52.23, 36.79, 32.71, 28.55, 28.34, 24.81, 24.60, 24.53, 24.45 ppm; **^{11}B NMR** (193 MHz, Chloroform-*d*) δ 33.57 ppm.

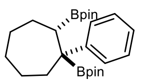

2,2'-(1-phenylcycloheptane-1,2-diyl)bis(4,4,5,5-tetramethyl-1,3,2-dioxaborolane) (5f): This compound was synthesized according to general procedure **A**. The residue was purified by column chromatography on silica gel to afford the product **5f** (petroleum ether/ethyl acetate = 30:1, 61.5 mg, 36% yield) as a white solid. **^{1}H NMR** (600 MHz, Chloroform-*d*) δ 1H NMR (600 MHz, Chloroform-d) δ 7.34 (d, J = 7.7 Hz, 2H), 7.19 (t, J = 7.3 Hz, 2H), 7.04 (t, J = 7.2 Hz, 1H), 2.31 (dd, J = 13.7, 7.7 Hz, 1H), 2.13 (d, J = 7.2 Hz, 1H), 1.96 (dt, J = 37.2, 10.9 Hz, 2H), 1.81 – 1.67 (m, 4H), 1.60 (t, J = 5.7 Hz, 1H), 1.54 – 1.42 (m, 2H), 1.18 (d, J = 18.8 Hz, 12H), 0.98 (d, J = 13.7 Hz, 12H) ppm; **^{13}C NMR** (151 MHz, Chloroform-*d*) δ 147.99, 127.70, 127.32, 124.33, 83.02, 82.41, 35.64, 28.53, 27.61, 26.63, 24.75, 24.60, 24.49 ppm; **^{11}B NMR** (193 MHz, Chloroform-*d*) δ 33.95 ppm; **HRMS (ESI)** calculated $[M+H]^+$ for $C_{28}H_{47}B_2O_4^+$ = 427.3185, found: 427.3175.

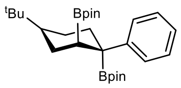

2,2'-(4-(tert-butyl)-1-phenylcyclohexane-1,2-diyl)bis(4,4,5,5-tetramethyl-1,3,2-dioxaborolane) (5g): This compound was synthesized according to general procedure **A**. The residue was purified by column chromatography on silica gel to afford the product **5g** (petroleum ether/ethyl acetate = 30:1, 104 mg, 55% yield) as a white solid. **^{1}H NMR** (600 MHz, Chloroform-*d*) δ 7.35 – 7.32 (m, 2H), 7.19 (t, *J* = 7.8 Hz, 2H), 7.04 (td, *J* = 7.3, 1.2 Hz, 1H), 2.36 – 2.27 (m, 2H), 2.04 (td, *J* = 12.5, 3.4 Hz, 1H), 1.93 (dt, *J* = 12.5, 3.0 Hz, 1H), 1.85 (dq, *J* = 13.2, 2.7 Hz, 1H), 1.46 (td, *J* = 12.7, 5.3 Hz, 1H), 1.22 (d, *J* = 8.3 Hz, 2H), 1.13 (s, 6H), 1.10 (s, 6H), 0.93 (s, 6H), 0.85 (m, 15H) ppm; **^{13}C NMR** (151 MHz, Chloroform-*d*) δ 147.08, 127.54, 126.73, 124.45, 83.06, 82.07, 44.72, 32.36, 29.46, 28.38, 27.47, 26.75, 24.50, 24.43, 24.32, 24.12

ppm; **^{11}B NMR** (193 MHz, Chloroform-*d*) δ 33.75 ppm. **HRMS (ESI)** calculated $[M+H]^+$ for $C_{28}H_{47}B_2O_4^+$ = 469.3655, found: 469.3670; The structure of **5g** was determined by X-ray single-crystal analysis of its oxidation product **5g-OH**.

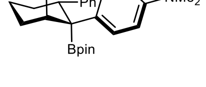

N,N-dimethyl-4-(2-phenyl-1,6-bis(4,4,5,5-tetramethyl-1,3,2-dioxaborolan-2-yl)cyclohexyl)aniline (5h): This compound was synthesized according to general procedure **A**. The residue was purified by column chromatography on silica gel to afford the product **5h** (petroleum ether/ethyl acetate = 5:1, 145 mg, 68% yield) as a white solid. **^{1}H NMR** (600 MHz, Chloroform-*d*) δ 7.30 (d, *J* = 7.6 Hz, 2H), 7.14 (t, *J* = 7.5 Hz, 2H), 7.06 (t, *J* = 8.7 Hz, 3H), 6.48 (d, *J* = 8.4 Hz, 2H), 3.66 (dd, *J* = 12.2, 3.5 Hz, 1H), 2.78 (s, 6H), 2.14 (dd, *J* =

18.4, 5.3 Hz, 2H), 2.05 – 1.96 (m, 1H), 1.94 – 1.85 (m, 1H), 1.71 (d, *J* = 11.4 Hz, 3H), 1.21 (d, *J* = 10.2 Hz, 12H), 1.00 (d, *J* = 13.0 Hz, 12H) ppm; **^{13}C NMR** (151 MHz, Chloroform-*d*) δ 147.84, 147.08, 134.94, 129.97, 129.60, 127.10, 124.67, 112.08, 82.76, 82.29, 46.26, 40.99, 33.75, 26.31, 24.97, 24.66, 24.64, 24.59, 24.55 ppm; **^{11}B NMR** (193 MHz, Chloroform-*d*) δ 34.50 ppm; **HRMS (ESI)** calculated $[M+H]^+$ for $C_{34}H_{52}B_2NO_4^+$ = 560.40770, found: 560.4077.

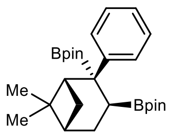

2,2'-((1R,5R)-6,6-dimethyl-2-phenylbicyclo[3.1.1]heptane-2,3-diyl)bis(4,4,5,5-tetramethyl-1,3,2-dioxaborolane (5i): This compound was synthesized according to general procedure **A**. The residue was purified by column chromatography on silica gel to afford the product **5i** (petroleum ether/ethyl acetate = 30:1, 100 mg, 55% yield) as a white solid. **^{1}H NMR** (600 MHz, Chloroform-*d*) δ 7.23 (d, *J* = 8.4 Hz, 2H), 7.18 (t, *J* = 7.6 Hz, 2H), 7.03 (t, *J* = 7.2 Hz, 1H), 2.60 (t, *J* = 5.5 Hz, 1H), 2.52 (dd, *J* = 11.0, 2.4 Hz, 1H), 2.31 (dt, *J* = 10.7, 6.0 Hz, 1H), 2.11 – 2.02 (m, 1H), 1.88 (d, *J* = 9.7 Hz, 1H), 1.82 (t, *J* = 5.3 Hz, 1H), 1.77 (ddd, *J* = 13.1, 4.5, 2.3 Hz, 1H), 1.30 (s, 3H), 1.17 (s, 6H), 1.00 (d, *J* = 24.6 Hz, 15H), 0.76 (s, 6H) ppm; **^{13}C NMR** (151 MHz, Chloroform-*d*) δ 147.81, 127.90, 127.87, 124.62, 83.15, 82.13, 44.86, 38.88, 38.82, 27.63, 27.22, 26.62, 24.89, 24.76, 24.43, 22.66. ppm; **^{11}B NMR (193 MHz, Chloroform-*d*)** δ 33.66 ppm; **HRMS (ESI)** calculated $[M+H]^+$ for $C_{27}H_{43}O_4B_2^+$ = 453.3342, found: 453.3358.

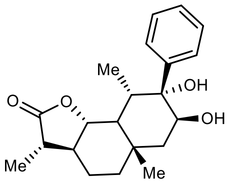

(3S,3aS,5aS,9S,9aS,9bS)-3,5a,9-trimethyl-8-phenyl-7,8-bis(4,4,5,5-tetramethyl-1,3,2-dioxaborolan-2-yl)decahydronaphtho[1,2-b]furan-2(3H)-one (5j): This compound was synthesized according to general procedure **B**. The residue was purified by column chromatography on silica gel to afford the product **5j** (petroleum ether/ethyl acetate = 1:1, 48.2 mg, 35% yield) as a white solid. **^{1}H NMR** (600 MHz, Chloroform-*d*) δ 7.64 – 7.58 (m, 2H), 7.36 (t, *J* = 7.6 Hz, 2H), 7.30 (t, *J* = 7.3 Hz, 1H), 4.15 – 4.11 (m, 1H), 3.84 (t, *J* = 10.1 Hz, 1H), 2.83 (s, 1H), 2.22 (tp, *J* = 13.4, 6.8, 6.2 Hz, 2H), 2.04 (s, 1H), 1.93 (t, *J* = 13.2 Hz, 1H), 1.87 – 1.80 (m, 3H), 1.70 (dt, *J* = 13.3, 2.9 Hz, 1H), 1.65 – 1.56 (m, 2H), 1.49 (dd, *J* = 13.0, 4.2 Hz, 1H), 1.18 (d, *J* = 7.1 Hz, 6H), 0.89 (d, *J* = 6.9 Hz, 3H) ppm; **^{13}C NMR** (151 MHz, Chloroform-*d*) δ 179.21, 139.52, 128.09, 127.97, 127.53, 82.61, 80.75, 53.71, 49.44, 47.44, 41.26, 40.86, 40.52, 37.52, 22.69, 19.86, 14.20, 12.34 ppm.

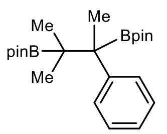

2,2'-(2-methyl-3-phenylbutane-2,3-diyl)bis(4,4,5,5-tetramethyl-1,3,2-dioxaborolane) (5k): This compound was synthesized according to general procedure **A**. The residue was purified by column chromatography on silica gel to afford the product **5k** (petroleum ether/ethyl acetate = 30:1, 100.8 mg, 63% yield) as a colorless oil. **^{1}H NMR** (600 MHz, Chloroform-*d*) δ 7.37 – 7.32 (m, 2H), 7.23 (t, *J* = 7.7 Hz, 2H), 7.15 – 7.11 (m, 1H), 1.47 (s, 3H), 1.26 (s, 6H), 1.24 – 1.22 (m, 18H), 0.96 (s, 3H), 0.72 (s, 3H). ppm; **^{13}C NMR** (151 MHz, Chloroform-*d*) δ 143.75, 129.88, 126.71, 125.09, 82.92, 82.69, 24.87, 24.81, 24.61, 24.57, 23.38, 20.21, 19.51 ppm; **^{11}B NMR** (193 MHz, Chloroform-*d*) δ 35.02 ppm; **HRMS (ESI)** calculated $[M+H]^+$ for $C_{23}H_{39}O_4B_2^+$ = 401.3029, found: 401.3038.

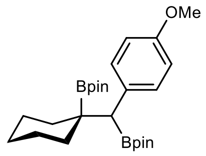

2-((4-methoxyphenyl)(1-(4,4,5,5-tetramethyl-1,3,2-dioxaborolan-2-yl)cyclohexyl)methyl)-4,4,5,5-tetramethyl-1,3,2-dioxaborolane (5l): This compound was synthesized according to general procedure **A**. The residue was purified by column chromatography on silica gel to afford the product **5l** (petroleum ether/ethyl acetate = 20:1, 94 mg, 51% yield) as a colorless oil. **^{1}H NMR** (600 MHz, Chloroform-*d*) δ 7.18 (d, *J* = 8.3 Hz, 2H), 6.74 (d, *J* = 8.2 Hz, 2H), 3.76 (s, 3H), 2.29 (s, 1H), 1.90 – 1.82 (m, 2H), 1.65 – 1.55 (m, 3H), 1.35 (dt, *J* = 13.0, 3.3 Hz, 1H), 1.25 (s, 7H), 1.22 – 1.16 (m, 18H), 1.14 – 1.06 (m, 2H), 1.04 – 0.97 (m, 1H) ppm; **^{13}C NMR** (151 MHz, Chloroform-*d*) δ 157.39, 132.25, 132.13, 112.67, 82.96, 82.86, 55.05, 34.66, 34.52, 26.40, 25.53, 25.28, 25.20, 25.00, 24.85, 24.72 ppm; **^{11}B NMR** (193 MHz, Chloroform-*d*) δ 34.81 ppm; **HRMS (ESI)** calculated $[M+H]^+$ for $C_{26}H_{43}B_2O_5^+$ = 457.3291, found: 457.3306.

5. Investigation of mechanism

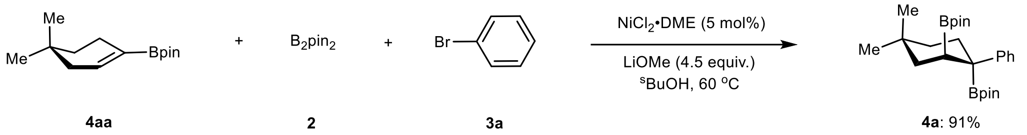

Under a nitrogen atmosphere, an oven-dried 10 mL reaction tube equipped with a magnetic stir bar and sealed with a rubber stopper was used. Sequentially, $NiCl_2$·DME (4.4 mg, 0.02 mmol, 5 mol%), MeOLi (38.0 mg, 1.0 mmol, 4.5 equiv.) and B_2pin_2 (406.2 mg, 1.6 mmol, 4.0 equiv.). Then anhydrous ^{s}BuOH (1.5 mL), **4aa** (0.4 mmol, 1.0 equiv.), **3a** (0.8 mmol, 2.0 equiv.) and anhydrous ^{s}BuOH (1.0 mL) were added in this order, and the mixture was stirred at 60 °C for 10 h. Then the mixture was filtered through a silica plug with ethyl acetate and concentrated. The crude product was purified by flash chromatography on silica gel to provide the compound **4a** as a white solid (160.2 mg, 91% yield).

6. Product derivatizations

Scale-up experiment

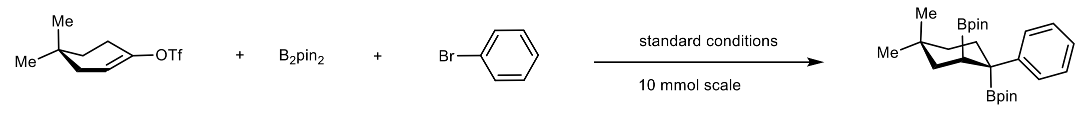

1a **2** **3a** **4a:** 72%, 3.2 g

Under a nitrogen atmosphere, an oven-dried 100 mL reaction tube equipped with a magnetic stir bar and sealed with a rubber stopper was used. Sequentially, $NiCl_2$·DME (110 mg, 0.5 mmol, 5 mol%), MeOLi (1.71 g, 45 mmol, 4.5 equiv.) and B_2pin_2 (10.2 g, 40 mmol, 4.0 equiv.). Then anhydrous ^{s}BuOH (30 mL), **1a** (10 mmol, 1.0 equiv.), **3a** (20 mmol, 2.0 equiv.) and anhydrous ^{s}BuOH (30 mL) were added in this order, and the mixture was stirred at 60 °C for 10 h. Then the reaction mixture was cooled to room temperature, quenched with water (20 mL) and further diluted with ethyl acetate (40 mL). Finally, the mixture was extracted with ethyl acetate and the combined organic layers were dried over anhydrous Na_2SO_4, filtered, and concentrated under vacuum. The mixture was purified by flash column chromatography to provide the title compound **4a** as a white solid (3.2 g, 72% yield).

Transformations of products

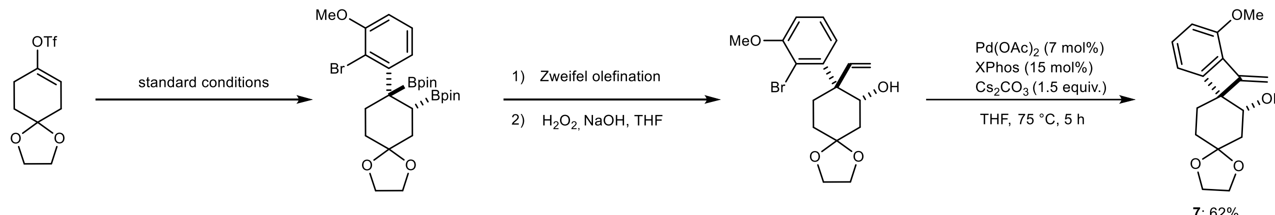

1-d **5l**: 48%; dr > 20:1 **6**: 47%; dr > 20:1 **7**: 62% dr > 20:1

2,2'-(8-(2-bromo-3-methoxyphenyl)-1,4-dioxaspiro[4.5]decane-7,8-diyl)bis(4,4,5,5-tetramethyl-1,3,2-dioxaborolane) (5l): This compound was synthesized according to general procedure **A**, 0.4 mmol scale, petroleum ether/ethyl acetate = 5:1, white solid, isolated yield: 112 mg, 48%, dr > 20:1. **^{1}H NMR** (600 MHz, Chloroform-*d*) δ 7.15 (d, *J* = 5.6 Hz, 2H), 6.73 (dd, *J* = 5.5, 4.0 Hz, 1H), 3.98 (tdd, *J* = 9.2, 4.3, 1.8 Hz, 1H), 3.94 – 3.88 (m, 3H), 3.85 (s, 3H), 2.84 (m, 1H), 2.66 (td, *J* = 12.9, 3.9 Hz, 1H), 2.57 (m, 1H), 2.09 (d, *J* = 12.2 Hz, 1H), 1.90 (dt, *J* = 13.5, 3.1 Hz, 1H), 1.83 – 1.72 (m, 2H), 1.24 (d, *J* = 8.0 Hz, 12H), 1.00 (s, 6H), 0.84 (s, 6H) ppm; **^{13}C NMR** (151 MHz, Chloroform-*d*) δ 155.46, 126.54, 123.26, 113.92, 109.11, 108.95, 83.25, 82.05, 64.31, 64.19, 56.48, 33.72, 30.28, 25.38, 25.18, 24.86, 24.45, 24.02 ppm; **^{11}B NMR (**193 MHz, Chloroform-*d*) δ 34.30 ppm.

According to a literature procedure[4],To an oven-dried 10 mL screwed test tube equipped with a Teflon coated magnetic stir bar, **5l** (90 mg, 0.15 mmol, 1.0 equiv.) and anhydrous THF (1 mL) were added under nitrogen atmosphere. Then, the solution was cooled to 0 °C and vinylmagnesium bromide (137.8 g, 1.05 mL, 1 M, 1.05 mmol, 7.0 equiv.) was slowly added. The mixture was warmed to room temperature and stirred for 2 h. The reaction was then cooled to -78 °C, and I_2 (266.5 mg, 1.05 mmol, 7.0 equiv.) in anhydrous THF (3 mL) was slowly added and stirred for 30 min at this temperature. Then, sodium methanolate (64.8 mg, 1.2 mmol, 8.0 equiv.) in anhydrous methanol (3 mL) was added and the reaction mixture was allowed to warm to room temperature. After stirring for 3 h, a saturated aqueous solution of $Na_2S_2O_3$ (2 mL) was added. The reaction mixture was diluted with water, and the aqueous layer was extracted with ethyl acetate (3 x 3 mL), the combined organic phase was dried over Na_2SO_4 and concentrated. Then, the corresponding alcohols can be obtained by oxidation with H_2O_2 (33 wt%, 2 mL). The mixture was quenched with Na_3SO_4 and extracted with ethyl acetate. The organic layer was washed with saturated brine, dried over anhydrous Na_2SO_4, and concentrated under reduced pressure. The residue was purified by column chromatography on silica gel to afford the oxidation product **6** (petroleum ether/ethyl acetate = 2:1, 26 mg, 47% yield, dr > 20:1) as a colorless oil. **^{1}H NMR** (600 MHz, Chloroform-*d*) δ 7.19 (t, *J* = 8.1 Hz, 1H), 6.94 (dd, *J* = 8.1, 1.4 Hz, 1H), 6.82 (dd, *J* = 8.2, 1.3 Hz, 1H), 5.95 (dt, *J* = 17.0, 10.0 Hz, 1H), 4.68 (dd, *J* = 17.0, 2.0 Hz, 1H), 4.62 (dd, *J* = 10.1, 2.1 Hz, 1H), 4.00 – 3.92 (m, 4H), 3.89 (s, 3H), 3.70 (ddt, *J* = 10.0, 5.2, 2.4 Hz, 1H), 2.61 – 2.52 (m, 1H), 2.41 (dd, *J* = 13.5, 5.6 Hz, 1H), 2.29 (td, *J* = 13.2, 3.8 Hz, 1H), 2.03 – 1.97 (m, 1H), 1.72 (ddt, *J* = 26.8, 13.6, 3.0 Hz, 2H), 1.26 (s, 1H) ppm; **^{13}C NMR** (151 MHz, Chloroform-*d*) δ 156.06, 144.86, 139.17, 127.61, 120.88, 115.46, 111.14, 110.91, 108.27, 75.85, 64.23, 63.95, 56.51, 46.95, 35.47, 31.20, 30.58 ppm.

A Schlenk tube was charged with $Pd(OAc)_2$ (0.8 mg, 7 mol%), XPhos (3.6 mg, 1.5 mol%), Cs_2CO_3 (24 mg, 1.5 equiv.), followed by a solution of the **6** (18.5 mg, 0.05 mmol, 1.0 equiv.) in THF (1.0 mL). The mixture was heated to 75 ºC under argon with stirring until the starting material had been consumed as judged by GC analysis. The mixture was cooled to room temperature, diluted with ether (3 mL), filtered, and concentrated in vacuo. The crude product was purified by flash chromatography on silica gel to provide the compound **7** as a colorless oil (petroleum ether/ethyl acetate = 2:1, 9 mg, 62% yield, dr > 20:1). **^{1}H NMR** (600 MHz, Chloroform-*d*) δ ^{1}H NMR (600 MHz, Chloroform-*d*) δ 7.28 – 7.25 (m, 1H), 7.02 (d, *J* = 7.5 Hz, 1H), 6.84 (d, *J* = 8.1 Hz, 1H), 5.95 (t, *J* = 1.6 Hz, 1H), 5.22 (t, *J* = 1.4 Hz, 1H), 4.04 – 3.99 (m, 1H), 3.99 – 3.92 (m, 3H), 3.90 (s, 3H), 3.00 (td, *J* = 7.9, 7.0, 1.8 Hz, 1H), 2.16 (s, 1H), 2.11 – 2.05 (m, 2H), 1.93 – 1.85 (m, 1H), 1.66 – 1.59 (m, 2H), 1.26 (s, 11H) ppm; **^{13}C NMR** (151 MHz, Chloroform-*d*) δ157.36, 150.20, 148.28, 129.97, 126.37, 114.57, 110.47, 109.00, 108.54, 64.26, 64.22, 55.22, 53.04, 36.30, 31.88, 30.93 ppm.

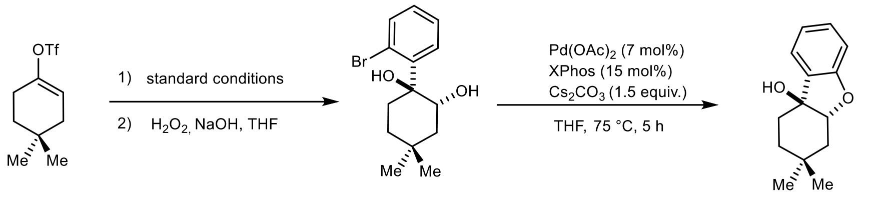

7,7-dimethyl-6,7,8,9-tetrahydrodibenzo[b,d]furan-9a(5aH)-ol (8): The vinyl triflate **1a** was treated according to General Procedure **B** to afford **4q-OH**. This material was carried forward without purification into the next transformation. According to a literature procedure[5], A Schlenk tube was charged with Pd(OAc)$_2$ (4.8 mg, 7 mol%), XPhos (21.6 mg, 1.5 mol%), Cs_2CO_3 (146 mg, 1.5 equiv.), followed by a solution of the **4q-OH** (89.8 mg, 0.3 mmol, 1.0 equiv.) in THF (1.0 mL). The mixture was heated to 75 °C under argon with stirring until the starting material had been consumed as judged by GC analysis. The mixture was cooled to room temperature, diluted with ether (3 mL), filtered, and concentrated in vacuo. The crude product was purified by flash chromatography on silica gel to provide the compound **8** as a colorless oil (petroleum ether/ethyl acetate = 5:1, 46 mg, 52% yield, dr > 20:1). **^{1}H NMR** (600 MHz, Chloroform-*d*) δ 7.31 – 7.22 (m, 2H), 6.95 (td, *J* = 7.4, 0.9 Hz, 1H), 6.87 (d, *J* = 8.0 Hz, 1H), 4.63 (dd, *J* = 9.6, 6.5 Hz, 1H), 2.29 (dt, *J* = 14.3, 4.3 Hz, 1H), 2.15 – 2.00 (m, 2H), 1.87 (ddd, *J* = 13.8, 6.5, 2.2 Hz, 1H), 1.39 (dtd, *J* = 13.7, 4.5, 2.2 Hz, 1H), 1.14 – 1.04 (m, 2H), 1.02 (s, 3H), 0.88 (s, 3H).ppm; **^{13}C NMR** (151 MHz, Chloroform-*d*) δ 159.00, 131.15, 130.44, 122.77, 120.97, 111.58, 88.55, 78.72, 41.94, 34.94, 31.36, 29.97, 28.99, 25.34.ppm; **HRMS (ESI)** calculated $[M+H]^+$ for $C_{14}H_{19}O_2^+$ = 220.1413, found: 220.17033.

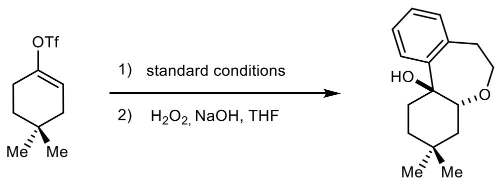

3,3-dimethyl-2,3,4,4a,6,7-hexahydrodibenzo[b,d]oxepin-11b(1H)-ol (**9**): This compound was synthesized according to general procedure **B**, the residue was purified by column chromatography on silica gel to afford the product **9** (petroleum ether/ethyl acetate = 5:1, 38 mg, 39% yield, dr > 20:1) as a colorless oil. **^{1}H NMR** (600 MHz, Chloroform-*d*) δ 7.48 – 7.45 (m, 1H), 7.22 – 7.18 (m, 2H), 7.15 – 7.11 (m, 1H), 3.99 (ddd, *J* = 11.1, 6.0, 4.8 Hz, 1H), 3.87 – 3.76 (m, 2H), 2.83 (td, *J* = 6.4, 5.9, 4.6 Hz, 2H), 2.31 (ddd, *J* = 14.1, 12.3, 4.0 Hz, 1H), 1.90 (dd, *J* = 14.2, 4.0 Hz, 1H), 1.74 (td, *J* = 12.8, 4.0 Hz, 1H), 1.59 – 1.57 (m, 1H), 1.56 – 1.53 (m, 1H), 1.32 (dtd, *J* = 13.3, 4.2, 1.9 Hz, 1H), 1.20 (s, 3H), 0.98 (s,

3H) ppm; **^{13}C NMR** (151 MHz, Chloroform-*d*) δ 139.26, 134.98, 129.21, 126.98, 126.50, 125.66, 72.04, 59.48, 41.15, 34.21, 32.85, 30.04, 29.65, 29.02, 28.07 ppm; **HRMS (ESI)** calculated $[M + H]^+$ for $C_{16} H_{23} O_2^+$ = 247.1692, found: 247.1696.

7. Arylative ketone migration

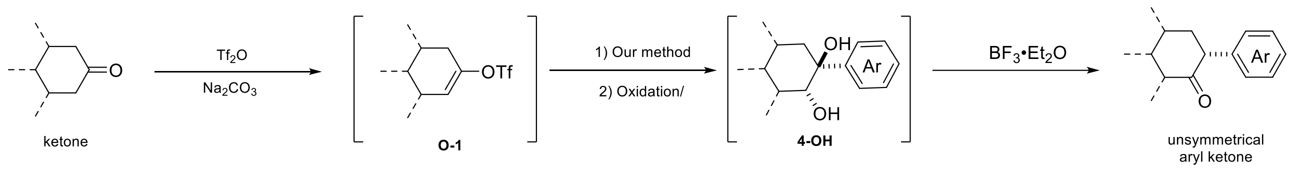

General procedure C: To a flame-dried 50 mL vial were added the ketone substrate (2 mmol, 1.0 equiv.), 336 mg Na_2CO_3 (3.2 mmol, 1.6 equiv.), 10 mL DCM in a glovebox under nitrogen atmosphere. 1.13g Tf_2O (4 mmol, 2.0 equiv.) was then added in one portion at the room temperature. The reaction vial was sealed and taken out the glovebox. It was stirred at the room temperature for 6 hours. Then the mixture was filtered through a silica plug with DCM and concentrated. The crude product **O-1** was used without further purification in the next reaction. The crude product **O-1** was treated according to general procedure **B** to give **4-OH**. This material was carried forward without purification into the next transformation. To a flame-dried 50 mL vial were added the **4-OH** (1.0 equiv.), DCM (0.2 M). Subsequently, BF_3•Et_2O (1.0 equiv.) was added at 0 °C. The reaction mixture was stirred at room temperature for 2 hours. Water (3 mL) was then added to quench the reaction. The mixture was then extracted 3 times with DCM and the combined organic extracts were dried with magnesium sulfate, filtered, and then purified via silica gel chromatography.

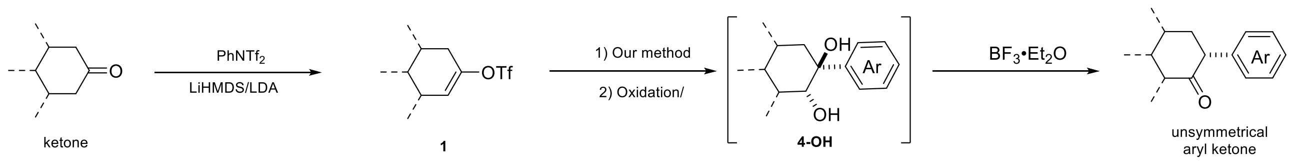

General procedure D: Step 1: To a solution of ketone (10.0 mmol) in THF (45 mL) was dropwise added lithium LiHMDS/LDA (6.0 mmol, 1.2 equiv.) at -78 °C. After stirring for 2 h, a solution of $PhNTf_2$ (12.0 mmol, 1.2 equiv.) in THF (10 mL) was added dropwise. The reaction mixture was allowed to warm to room temperature and stirred for 16 h. The mixture was quenched with NH_4Cl and extracted with ethyl acetate. The organic layer was washed with saturated brine, dried over anhydrous Na_2SO_4, and concentrated under reduced pressure. The crude product was purified by flash chromatography on silica gel to give the resulting vinyl triflate **1**

Step 2: The product **1** was treated according to general procedure **B** to give **4-OH**. This material was carried forward

without purification into the next transformation. To a flame-dried 50 mL vial were added the **4-OH** (1.0 equiv.), DCM (0.2 M). Subsequently, BF_3•Et_2O (1.0 equiv.) was added at 0 °C. The reaction mixture was stirred at room temperature for 2 hours. Water (3 mL) was then added to quench the reaction. The mixture was then extracted 3 times with DCM and the combined organic extracts were dried with magnesium sulfate, filtered, and then purified via silica gel chromatography.

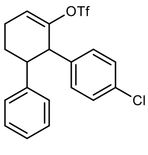

4''-chloro-1',2',5',6'-tetrahydro-[1,1':2',1''-terphenyl]-3'-yl trifluoromethanesulfonate (1-22): This compound was synthesized according to general procedure **D** (Step 1). The residue was purified by column chromatography on silica gel to afford the product **1-22** (petroleum ether/ethyl acetate = 50:1, 1.5 mmol scale, 81% yield) as a pale yellow oil. **^{1}H NMR** (600 MHz, Chloroform-*d*) δ 7.19 (dt, *J* = 24.0, 7.6 Hz, 5H), 6.97 (d, *J* = 7.6 Hz, 2H), 6.88 (d, *J* = 7.9 Hz, 2H), 6.15 – 6.09 (m, 1H), 3.78 (d, *J* = 7.7 Hz, 1H), 2.92 – 2.83 (m, 1H), 2.50 – 2.37 (m, 2H), 2.04 – 1.95 (m, 2H) ppm; **^{13}C NMR** (151 MHz, Chloroform-*d*) δ 149.09, 142.34, 137.00, 132.92, 129.66, 128.53, 128.46, 127.33, 126.86, 121.23, 119.66 – 114.86 (m), 51.73, 50.81, 28.41, 23.86 ppm.

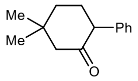

5,5-dimethyl-2-phenylcyclohexan-1-one (10): This compound was synthesized according to general procedure **C**. 2 mmol scale, petroleum ether/ethyl acetate = 20:1, white solid, isolated yield: 182.1 mg, 45%. **^{1}H NMR** (600 MHz, Chloroform-*d*) δ 7.34 (t, *J* = 7.6 Hz, 2H), 7.25 (tt, *J* = 7.1, 1.3 Hz, 1H), 7.16 – 7.13 (m, 2H), 3.52 (dd, *J* = 11.2, 6.9 Hz, 1H), 2.36 (dt, *J* = 13.1, 0.9 Hz, 1H), 2.26 (dd, *J* = 13.1, 2.3 Hz, 1H), 2.20 – 2.11 (m, 2H), 1.81 (ddd, *J* = 13.6, 11.1, 5.4 Hz, 1H), 1.72 (dtd, *J* = 13.7, 3.9, 2.3 Hz, 1H), 1.11 (s, 3H), 1.01 (s, 3H) ppm; **^{13}C NMR** (151 MHz, Chloroform-*d*) δ 209.96, 138.59, 128.55, 128.32, 126.86, 56.26, 54.94, 38.24, 36.85, 31.41, 30.78, 25.73 ppm; **HRMS (ESI)** calculated $[M+H]^+$ for $C_{14}H_{19}O^+$ = 203.14376, found: 203.1437.

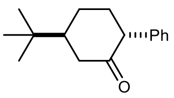

5,5-dimethyl-2-phenylcyclohexan-1-one (11): This compound was synthesized according to general procedure **C**. 1.2 mmol scale, petroleum ether/ethyl acetate = 20:1, white solid, isolated yield: 69.1 mg, 25%, dr > 20:1. **^{1}H NMR** (600 MHz, Chloroform-*d*) δ 7.36 – 7.31 (m, 2H), 7.25 (d, *J* = 6.3 Hz, 1H), 7.14 – 7.10 (m, 2H), 3.54 (dd, *J* = 13.5, 5.7 Hz, 1H), 2.60 (ddd, *J* = 13.1, 4.0, 2.2 Hz, 1H), 2.35 – 2.28 (m, 1H), 2.25 (td, *J* = 13.2, 1.4 Hz, 1H), 2.07 (dq, *J* = 13.0, 3.2 Hz, 1H), 1.90 (qd, *J* = 13.2, 3.3 Hz, 1H), 1.71 – 1.64 (m, 1H), 1.59 – 1.54 (m, 1H), 0.94 (s, 9H) ppm; **^{13}C NMR** (151 MHz, Chloroform-*d*) δ 210.67, 138.78, 128.70, 128.29, 126.90, 57.16, 50.27, 44.06, 34.35, 32.81, 27.23, 26.87 ppm; **HRMS (ESI)** calculated $[M+H]^+$ for $C_{16}H_{23}O^+$ = 231.1743, found: 231.1746.

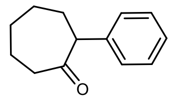

2-phenylcycloheptan-1-one (13): This compound was synthesized according to general procedure **C**. 0.8 mmol scale, petroleum ether/ethyl acetate = 20:1, white solid, isolated yield: 21.1 mg, 14%, dr > 20:1. **^{1}H NMR** (600 MHz, Chloroform-*d*) δ 7.31 (t, *J* = 7.2 Hz, 2H), 7.23 (t, *J* = 8.6 Hz, 3H), 3.72 (dd, *J* = 11.4, 3.7 Hz, 1H), 2.69 (t, *J* = 12.7 Hz, 1H), 2.55 – 2.47 (m, 1H), 2.14 (dt, *J* = 13.0, 5.3 Hz, 1H), 2.07 – 1.94 (m, 4H), 1.64 (d, *J* = 11.4 Hz, 1H), 1.46 (t, *J* = 9.9 Hz, 2H) ppm; **^{13}C NMR** (151 MHz, Chloroform-*d*) δ 213.50, 140.35, 128.49, 127.83, 126.86, 58.76, 42.71, 31.95, 29.98, 28.54, 25.30 ppm; **HRMS (ESI)** calculated $[M+H]^+$ for $C_{16}H_{23}O^+$ = 190.1307, found: 190.1301.

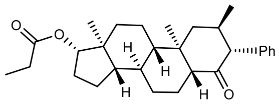

(2R,3R,5R,8R,9S,10R,13S,14S,17S)-2,10,13-trimethyl-4-oxo-3-phenylhexadecahydro-1H-cyclopenta[a]phenanthren-17-yl propionate (15): This compound was synthesized according to general procedure **D**. The residue was purified by column chromatography on silica gel to afford the product **15** (petroleum ether/ethyl acetate = 5:1, 45.4 mg, 24% yield, dr > 20:1) as a white solid (The synthesis was carried out in two steps: Step 1 (0.6 mmol scale, 92% yield) followed by Step 2 (0.4 mmol scale, 26% yield), affording the target compound in 24% overall yield). **^{1}H NMR** (600 MHz, Chloroform-*d*) 7.41 (d, *J* = 7.6 Hz, 2H), 7.31 (q, *J* = 7.3 Hz, 2H), 7.25 (s, 1H), 4.62 (t, *J* = 7.5 Hz, 1H), 3.61 (d, *J* = 4.9 Hz, 1H), 2.57 – 2.44 (m, 2H), 2.32 (tt, *J* = 7.6, 5.0 Hz, 2H), 2.16 (td, *J* = 9.3, 4.7 Hz, 1H), 1.87 – 1.76 (m, 3H), 1.73 – 1.62 (m, 3H), 1.57 (d, *J* = 3.1 Hz, 4H), 1.41 (d, *J* = 11.2 Hz, 2H), 1.32 – 1.24 (m, 4H), 1.14 (q, *J* = 7.4 Hz, 3H), 0.91 (dd, *J* = 7.4, 3.2 Hz, 3H), 0.87 (d, *J* = 3.3 Hz, 3H), 0.80 (d, *J* = 3.3 Hz, 3H) ppm; **^{13}C NMR** (151 MHz, Chloroform-*d*) δ 212.40, 174.61, 136.89, 129.70, 128.57, 126.93, 82.49, 62.07, 54.72, 53.93, 50.56, 43.81, 42.64, 40.93, 36.90, 34.73, 31.19, 29.90, 27.82, 27.54, 23.43, 20.98, 20.45, 18.51, 14.94, 12.14, 9.27 ppm; **HRMS (ESI)** calculated $[M+H]^+$ for $C_{29}H_{41}O_3^+$ = 437.3050, found: 437.3052.

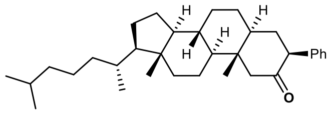

(3S,5S,8R,9S,10S,13R,14S,17R)-10,13-dimethyl-17-((R)-6-methylheptan-2-yl)-3-phenylhexadecahydro-2H-cyclopenta[a]phenanthren-2-one (17): This compound was synthesized according to general procedure **D**. The residue was purified by column chromatography on silica gel to afford the product **17** (petroleum ether/ethyl acetate = 20:1, 113 mg, 54% yield, dr > 20:1) as a white solid (The synthesis was carried out in two steps: Step 1 (2.6 mmol scale, 89% yield) followed by Step 2 (0.4 mmol scale, 61% yield), affording the target compound in 54% overall yield). **^{1}H NMR** (600 MHz, Chloroform-*d*) δ 7.32 (t, *J* = 7.5 Hz, 2H), 7.26 – 7.22 (m, 1H), 7.13 – 7.07 (m, 2H), 3.58 (dd, *J* = 12.6, 6.7 Hz, 1H), 2.54 (d, *J* = 13.0 Hz, 1H), 2.15 (d, *J* = 12.9 Hz, 1H), 2.03 – 1.72 (m, 6H), 1.62 – 1.48 (m, 3H), 1.46 – 1.40 (m, 1H), 1.33 (dddd, *J* = 33.0, 17.1, 9.4, 3.5 Hz, 7H), 1.20 – 1.06 (m,

6H), 1.05 – 0.95 (m, 4H), 0.91 (d, J = 6.5 Hz, 3H), 0.89 – 0.85 (m, 9H), 0.66 (s, 3H) ppm; **^{13}C NMR** (151 MHz, Chloroform-*d*) δ 210.02, 138.73, 128.68, 128.23, 126.78, 57.34, 56.28, 56.21, 54.19, 53.92, 45.87, 42.46, 41.36, 39.72, 39.46, 38.22, 36.11, 35.73, 34.78, 31.63, 28.18, 27.95, 27.63, 24.16, 23.80, 22.78, 22.52, 21.08, 18.62, 12.69, 11.97 ppm; **HRMS (ESI) calculated** $[M+H]^+$ for $C_{28}H_{37}O_2^+$ = 405.2788, found: 405.2782.

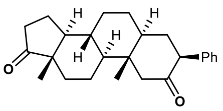

(3S,5S,8R,9S,10S,13S,14S)-10,13-dimethyl-3-phenyltetradecahydro-1H-cyclopenta[a]phenanthrene-2,17-dione (19). This compound was synthesized according to general procedure D. The residue was purified by column chromatography on silica gel to afford the product **19** (petroleum ether/ethyl acetate = 5:1, 80.2 mg, 47% yield, dr > 20:1) as a colorless solid (The synthesis was carried out in two steps: Step 1 (4.5 mmol scale, 86% yield) followed by Step 2 (0.4 mmol scale, 55% yield), affording the target compound in 47% overall yield). **^{1}H NMR** (600 MHz, Chloroform-*d*) δ 7.33 (t, J = 7.6 Hz, 2H), 7.27 – 7.23 (m, 1H), 7.14 – 7.09 (m, 2H), 3.61 (dd, J = 12.5, 6.6 Hz, 1H), 2.56 (d, J = 12.9 Hz, 1H), 2.46 (ddd, J = 19.3, 8.9, 1.1 Hz, 1H), 2.21 – 2.17 (m, 1H), 2.09 (dt, J = 19.1, 9.0 Hz, 1H), 2.02 (ddd, J = 13.2, 6.6, 3.5 Hz, 1H), 1.99 – 1.92 (m, 2H), 1.91 – 1.82 (m, 3H), 1.61 – 1.51 (m, 4H), 1.37 – 1.26 (m, 4H), 1.11 – 1.03 (m, 2H), 0.90 (s, 3H), 0.88 (s, 3H) ppm; **^{13}C NMR** (151 MHz, Chloroform-*d*) δ 211.57, 209.54, 138.52, 128.68, 128.29, 126.89, 57.29, 54.07, 53.95, 51.23, 47.64, 45.87, 41.44, 38.06, 35.74, 34.37, 31.33, 30.48, 27.33, 21.72, 20.37, 13.73, 12.72 ppm; **HRMS (ESI) calculated** $[M+H]^+$ for $C_{25}H_{33}O_2{}^+$ = 365.2475, found: 365.2492.

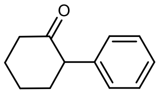

2-phenylcyclohexan-1-one (21): This compound was synthesized according to general procedure **C**. 10 mmol scale, petroleum ether/ethyl acetate = 20:1, white solid, isolated yield: 749.2 mg, 43%. **^{1}H NMR** (600 MHz, Chloroform-*d*) δ 7.31 (t, J = 7.6 Hz, 2H), 7.23 (t, J = 7.6 Hz, 1H), 7.12 (d, J = 7.5 Hz, 2H), 3.58 (dd, J = 12.4, 5.3 Hz, 1H), 2.46 (ddd, J = 36.9, 12.9, 5.3 Hz, 2H), 2.23 (dt, J = 12.4, 4.0 Hz, 1H), 2.15 – 2.07 (m, 1H), 2.05 – 1.91 (m, 2H), 1.85 – 1.73 (m, 2H) ppm; **^{13}C NMR** (151 MHz, Chloroform-*d*) δ 210.03, 138.62, 128.35, 128.13, 126.66, 57.15, 41.99, 34.92, 27.62, 25.11 ppm; **HRMS (ESI) calculated** $[M+H]^+$ for $C_{12}H_{15}O^+$ = 175.1117, found: 175.1119.

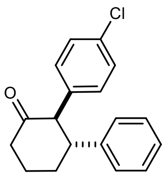

2-(4-chlorophenyl)-3-phenylcyclohexan-1-one (22): This compound was synthesized according to general procedure **D**. The residue was purified by column chromatography on silica gel to afford the product **22** (petroleum ether/ethyl acetate = 5:1, 555.3 mg, 35% yield, dr > 20:1) as a colorless oil (The

synthesis was carried out in two steps: Step 1 (20 mmol scale, 90% yield) followed by Step 2 (5 mmol scale, 39% yield), affording the target compound in 35% overall yield). **^{1}H NMR** (600 MHz, Chloroform-*d*) δ 7.21 – 6.95 (m, 7H), 6.85 (d, *J* = 8.1 Hz, 2H), 3.80 (d, *J* = 12.2 Hz, 1H), 3.17 (t, *J* = 12.4 Hz, 1H), 2.62 (d, *J* = 20.2 Hz, 2H), 2.30 – 2.05 (m, 3H), 1.95 (t, *J* = 13.9 Hz, 1H) ppm; **^{13}C NMR** (151 MHz, Chloroform-*d*) δ 208.89, 142.59, 135.18, 132.30, 130.72, 128.41, 128.10, 127.12, 126.54, 62.94, 52.42, 41.96, 34.55, 26.19 ppm; **HRMS (ESI)** calculated $[M+H]^+$ for $C_{18}H_{18}OCl^+$ = 285.1040, found: 285.1045.

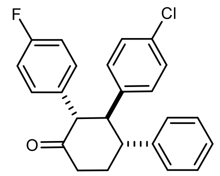

3-(4-chlorophenyl)-2-(4-fluorophenyl)-4-phenylcyclohexan-1-one (23): This compound was synthesized according to general procedure D. The residue was purified by column chromatography on silica gel to afford the product **23** (petroleum ether/ethyl acetate = 5:1, 77.7 mg, 33% yield, dr > 20:1) as a pale yellow oil (The synthesis was carried out in two steps: Step 1 (1.5 mmol scale, 81% yield) followed by Step 2 (0.5 mmol scale, 41% yield), affording the target compound in 33% overall yield). **^{1}H NMR** (600 MHz, Chloroform-*d*) δ 7.13 (t, *J* = 7.5 Hz, 2H), 7.07 (t, *J* = 7.4 Hz, 1H), 6.99 (d, *J* = 7.6 Hz, 2H), 6.94 – 6.80 (m, 6H), 6.71 (d, *J* = 8.0 Hz, 2H), 3.92 (d, *J* = 11.8 Hz, 1H), 3.40 – 3.28 (m, 2H), 2.86 (td, *J* = 14.0, 5.8 Hz, 1H), 2.78 (dt, *J* = 13.9, 3.5 Hz, 1H), 2.39 (ddt, *J* = 13.3, 6.4, 3.0 Hz, 1H), 2.26 (qd, *J* = 13.5, 3.8 Hz, 1H) ppm; **^{13}C NMR** (151 MHz, Chloroform-*d*) δ 208.00, 162.36, 161.54 (d, J = 245.1 Hz), 139.39, 131.86 (d, J = 3.3 Hz), 131.74, 130.87 (d, J = 8.0 Hz), 128.97, 128.41, 128.15, 127.33, 126.63, 115.01 (d, J = 21.3 Hz), 62.47, 57.90, 50.44, 42.06, 34.35 ppm; **^{19}F NMR** (565 MHz, Chloroform-*d*) δ -115.81; **HRMS (ESI)** calculated $[M+H]^+$ for $C_{24}H_{21}OClF^+$ = 379.1259, found: 379.1272.

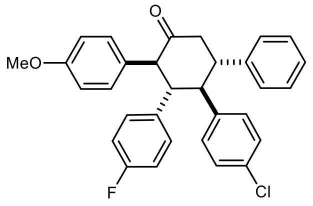

4-(4-chlorophenyl)-3-(4-fluorophenyl)-2-(4-methoxyphenyl)-5-phenylcyclohexan-1-one (24): This compound was synthesized according to general procedure D. The residue was purified by column chromatography on silica gel to afford the product **24** (petroleum ether/ethyl acetate = 2:1, 60 mg, 25% yield, dr > 20:1) as a pale yellow oil (The synthesis was carried out in two steps: Step 1 (0.6 mmol scale, 92% yield) followed by Step 2 (0.5 mmol scale, 27% yield), affording the target compound in 25% overall yield). **^{1}H NMR** (600 MHz, Chloroform-*d*) δ 7.16 (t, J = 7.2 Hz, 2H), 7.11 – 7.05 (m, 3H), 6.89 (t, J = 6.4 Hz, 4H), 6.82 – 6.67 (m, 6H), 6.64 (t, J = 8.1 Hz, 2H), 4.08 (d, J = 12.1 Hz, 1H), 3.71 (s, 3H), 3.57 – 3.40 (m, 3H), 3.13 (t, J = 13.1 Hz, 1H), 2.92 (d, J = 13.7 Hz, 1H) ppm; **^{13}C NMR** (151 MHz, Chloroform-*d*) δ 207.29, 161.68, 160.06, 158.24, 141.68, 139.02, 136.42 (d, *J* = 3.2 Hz), 132.53 – 125.43 (m), 114.91 (d, *J* = 21.3 Hz),

113.56, 62.47, 57.37, 56.23, 55.01, 50.47, 49.69 ppm; ^{19}**F NMR** (565 MHz, Chloroform-*d*) δ -116.11; **HRMS (ESI)** calculated $[M+H]^+$ for $C_{24}H_{21}OClF^+$ = 485.1678, found: 485.1668.

8. X-ray structures and data

Crystals suitable for X-ray single-crystal diffraction analysis were obtained from Hexane/ ethyl acetate using slow evaporation under air at room temperature.

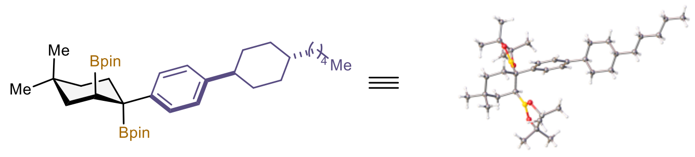

4k CCDC = 2465089

Table 1 Crystal data and structure refinement for 2465089

Identification code	Wsy-2-174-8
Empirical formula	$C_{37}H_{62}B_2O_4$
Formula weight	592.551
Temperature/K	293(2)
Crystal system	triclinic
Space group	P-1
a/Å	10.2654(10)
b/Å	11.9156(11)
c/Å	16.2107(16)
α/°	72.864(4)
β/°	83.243(4)
γ/°	88.842(4)
Volume/Å^3	1881.5(3)
Z	2
ρ_{calc}g/cm^3	1.046
μ/mm^{-1}	0.330
F(000)	653.4
Crystal size/mm^3	0.1 × 0.06 × 0.05
Radiation	synchrotron (λ = 1.34139)
2Θ range for data collection/°	6.76 to 114.62
Index ranges	$-12 \le h \le 12$, $-14 \le k \le 14$, $-20 \le l \le 20$
Reflections collected	60119
Independent reflections	7741 [R_{int} = 0.0663, R_{sigma} = 0.0415]
Data/restraints/parameters	7741/223/399
Goodness-of-fit on F^2	1.048
Final R indexes [I>=2σ (I)]	R_1 = 0.0855, wR_2 = 0.1670
Final R indexes [all data]	R_1 = 0.1353, wR_2 = 0.1867
Largest diff. peak/hole / e Å^{-3}	0.45/-0.40

Crystals suitable for X-ray single-crystal diffraction analysis were obtained from Hexane/ ethyl acetate using slow evaporation under air at room temperature.

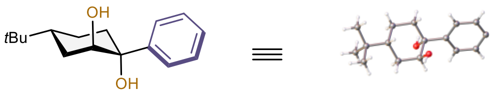

5g-OH CCDC = 2465095

Table 1 Crystal data and structure refinement for 2465095

Identification code	wsy-3-74-1
Empirical formula	$C_{32}H_{48}O_4$
Formula weight	496.70
Temperature/K	298.00(10)
Crystal system	monoclinic
Space group	$P2_1/c$
a/Å	9.6881(3)
b/Å	11.3723(3)
c/Å	26.8905(8)
α/°	90
β/°	93.128(3)
γ/°	90
Volume/Å^3	2958.25(14)
Z	4
ρ_{calc}g/cm^3	1.115
μ/mm^{-1}	0.558
F(000)	1088.0
Crystal size/mm^3	0.07 × 0.05 × 0.02
Radiation	Cu Kα (λ = 1.54184)
2Θ range for data collection/°	6.584 to 147.2
Index ranges	$-12 \le h \le 11$, $-8 \le k \le 13$, $-33 \le l \le 33$
Reflections collected	26311
Independent reflections	5759 [R_{int} = 0.0352, R_{sigma} = 0.0283]
Data/restraints/parameters	5759/6/336
Goodness-of-fit on F^2	1.037
Final R indexes [I>=2σ (I)]	R_1 = 0.0503, wR_2 = 0.1214
Final R indexes [all data]	R_1 = 0.0725, wR_2 = 0.1323
Largest diff. peak/hole / e Å^{-3}	0.18/-0.15

Note:

The observed phenomenon may be attributed to the disordered nature of atoms O31 and H31. We are confident that these structural features are valid. The following explanation has been provided for the B-level alert in CCDC entry 2465095: PLAT420_ALERT_2_B D-H Bond Without Acceptor O31 --H31. Please Check

Author Response: This problem may arise from the disordered nature of O31 and H31 atoms.

Crystals suitable for X-ray single-crystal diffraction analysis were obtained from Hexane/ ethyl acetate using slow evaporation under air at room temperature.

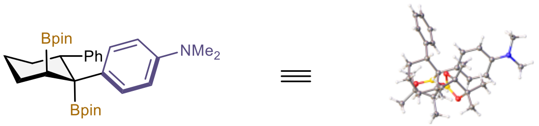

5h CCDC = 2465128

Table 1 Crystal data and structure refinement for 2465128

Identification code	wsy-3-78-1
Empirical formula	$C_{32}H_{47}B_2NO_4$
Formula weight	531.32
Temperature/K	100.00(13)
Crystal system	triclinic
Space group	P-1
a/Å	10.1591(2)
b/Å	12.1448(3)
c/Å	13.7869(3)
α/°	96.133(2)
β/°	100.7484(19)
γ/°	111.919(2)
Volume/Å^3	1521.05(7)
Z	2
ρ_{calc}g/cm^3	1.160
μ/mm^{-1}	0.576
F(000)	576.0
Crystal size/mm^3	0.22 × 0.1 × 0.04
Radiation	Cu Kα (λ = 1.54184)
2Θ range for data collection/°	6.652 to 146.738
Index ranges	$-11 \le h \le 12$, $-14 \le k \le 15$, $-17 \le l \le 13$
Reflections collected	20731
Independent reflections	5815 [R_{int} = 0.0238, R_{sigma} = 0.0242]
Data/restraints/parameters	5815/0/363
Goodness-of-fit on F^2	1.069
Final R indexes [I>=2σ (I)]	R_1 = 0.0372, wR_2 = 0.0939
Final R indexes [all data]	R_1 = 0.0428, wR_2 = 0.0977
Largest diff. peak/hole / e Å^{-3}	0.30/-0.20

Crystals suitable for X-ray single-crystal diffraction analysis were obtained from Hexane/ ethyl acetate using slow evaporation under air at room temperature.

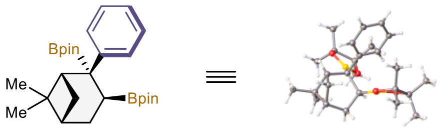

5i CCDC = 2465131

Table 1 Crystal data and structure refinement for 2465131

Identification code	wsy-2-184-3
Empirical formula	$C_{27}H_{42}B_2O_4$
Formula weight	452.22
Temperature/K	100(2)
Crystal system	orthorhombic
Space group	$P2_12_12_1$
a/Å	6.7108(9)
b/Å	19.549(3)
c/Å	19.549(3)
α/°	90
β/°	90
γ/°	90
Volume/Å^3	2564.6(6)
Z	4
ρ_{calc}g/cm^3	1.171
μ/mm^{-1}	0.373
F(000)	984.0
Crystal size/mm^3	0.1 × 0.06 × 0.05
Radiation	GaKα (λ = 1.34139)
2Θ range for data collection/°	5.562 to 105.946
Index ranges	$-7 \leq h \leq 7$, $-23 \leq k \leq 23$, $-23 \leq l \leq 23$
Reflections collected	88620
Independent reflections	4528 [R_{int} = 0.1364, R_{sigma} = 0.0552]
Data/restraints/parameters	4528/0/309
Goodness-of-fit on F^2	0.985
Final R indexes [I>=2σ (I)]	R_1 = 0.0580, wR_2 = 0.1360
Final R indexes [all data]	R_1 = 0.0849, wR_2 = 0.1526
Largest diff. peak/hole / e Å^{-3}	0.31/-0.36
Flack parameter	0.23(15)

Crystals suitable for X-ray single-crystal diffraction analysis were obtained from Hexane/ ethyl acetate using slow evaporation under air at room temperature.

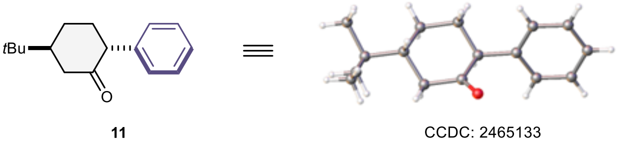

Table 1 Crystal data and structure refinement for 2465133

Identification code	wsy-3-74-1-T
Empirical formula	$C_{32}H_{44}O_2$
Formula weight	460.67

Temperature/K	298.83(10)
Crystal system	monoclinic
Space group	$P2_1/c$
a/Å	6.06396(12)
b/Å	25.1816(5)
c/Å	18.1651(4)
α/°	90
β/°	90.033(2)
γ/°	90
Volume/Å^3	2773.82(10)
Z	4
ρ_{calc}g/cm^3	1.103
μ/mm^{-1}	0.507
F(000)	1008.0
Crystal size/mm^3	0.11 × 0.07 × 0.03
Radiation	Cu Kα (λ = 1.54184)
2Θ range for data collection/°	6 to 147.1
Index ranges	$-7 \le h \le 6, -29 \le k \le 31, -22 \le l \le 22$
Reflections collected	40988
Independent reflections	5494 [R_{int} = 0.0325, R_{sigma} = 0.0206]
Data/restraints/parameters	5494/0/314
Goodness-of-fit on F^2	1.111
Final R indexes [I>=2σ (I)]	R_1 = 0.0549, wR_2 = 0.1276
Final R indexes [all data]	R_1 = 0.0731, wR_2 = 0.1369
Largest diff. peak/hole / e Å^{-3}	0.17/-0.14

Crystals suitable for X-ray single-crystal diffraction analysis were obtained from Hexane/ ethyl acetate using slow evaporation under air at room temperature.

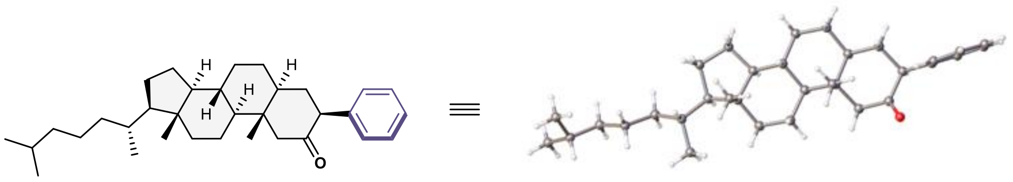

17 CCDC: 2465134

able 1 Crystal data and structure refinement for 2465134

Identification code	wsy-2-184-2
Empirical formula	$C_{33}H_{50}O$
Formula weight	462.764
Temperature/K	100.00
Crystal system	monoclinic
Space group	$P2_1$
a/Å	10.9765(4)

b/Å	12.0873(5)
c/Å	21.0187(9)
α/°	90
β/°	93.064(1)
γ/°	90
Volume/Å^3	2784.70(19)
Z	4
ρ_{calc}g/cm^3	1.104
μ/mm^{-1}	0.303
F(000)	1026.0
Crystal size/mm^3	0.3 × 0.2 × 0.2
Radiation	synchrotron (λ = 1.34139)
2Θ range for data collection/°	7.02 to 144.94
Index ranges	-14 ≤ h ≤ 15, -17 ≤ k ≤ 17, -29 ≤ l ≤ 29
Reflections collected	102783
Independent reflections	16506 [R_{int} = 0.0370, R_{sigma} = 0.0246]
Data/restraints/parameters	16506/1/624
Goodness-of-fit on F^2	1.022
Final R indexes [I>=2σ (I)]	R_1 = 0.0306, wR_2 = 0.0833
Final R indexes [all data]	R_1 = 0.0316, wR_2 = 0.0840
Largest diff. peak/hole / e Å^{-3}	0.28/-0.14
Flack parameter	-0.05(5)

Crystals suitable for X-ray single-crystal diffraction analysis were obtained from Hexane/ ethyl acetate using slow evaporation under air at room temperature.

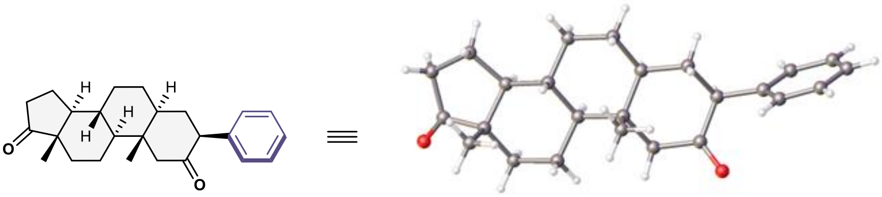

19 CCDC: 2465135

Table 1 Crystal data and structure refinement for 2465135

Identification code	wsy-3-59-4
Empirical formula	$C_{25}H_{32}O_2$
Formula weight	364.50
Temperature/K	276.3(2)
Crystal system	orthorhombic
Space group	$P2_12_12_1$
a/Å	6.5429(5)
b/Å	12.7266(8)
c/Å	24.3523(11)

α/°	90
β/°	90
γ/°	90
Volume/Å^3	2027.8(2)
Z	4
ρ_{calc}g/cm^3	1.194
μ/mm^{-1}	0.567
F(000)	792.0
Crystal size/mm^3	0.2 × 0.08 × 0.05
Radiation	Cu Kα (λ = 1.54184)
2Θ range for data collection/°	7.26 to 150.674
Index ranges	$-8 \le h \le 8$, $-15 \le k \le 15$, $-29 \le l \le 26$
Reflections collected	12613
Independent reflections	3990 [R_{int} = 0.0287, R_{sigma} = 0.0262]
Data/restraints/parameters	3990/0/247
Goodness-of-fit on F^2	1.096
Final R indexes [I>=2σ (I)]	R_1 = 0.0375, wR_2 = 0.0989
Final R indexes [all data]	R_1 = 0.0432, wR_2 = 0.1021
Largest diff. peak/hole / e Å^{-3}	0.14/-0.11
Flack parameter	0.01(14)

Crystals suitable for X-ray single-crystal diffraction analysis were obtained from Hexane/ ethyl acetate using slow evaporation under air at room temperature.

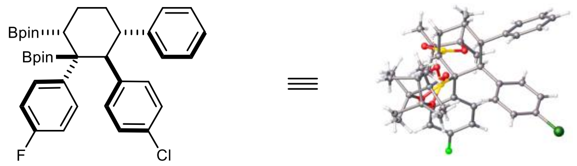

23-B

CCDC: 2504106

Table 1 Crystal data and structure refinement for 2504106.

Identification code	WSY-3-84-1-B
Empirical formula	$C_{36}H_{44}B_2ClFO_4$
Formula weight	616.78
Temperature/K	295.72(10)
Crystal system	triclinic
Space group	P-1
a/Å	9.55786(15)
b/Å	12.7189(3)
c/Å	16.1811(4)
α/°	107.354(2)
β/°	99.0722(17)
γ/°	109.0766(19)
Volume/Å3	1701.95(7)

Z	2
ρcalcg/cm3	1.204
μ/mm-1	1.328
F(000)	656.0
Crystal size/mm3	0.22 × 0.12 × 0.02
Radiation	Cu Kα (λ = 1.54184)
2Θ range for data collection/°	5.966 to 151.676
Index ranges	-11 ≤ h ≤ 9, -15 ≤ k ≤ 15, -20 ≤ l ≤ 19
Reflections collected	45158
Independent reflections	6810 [Rint = 0.0267, Rsigma = 0.0142]
Data/restraints/parameters	6810/228/482
Goodness-of-fit on F2	1.073
Final R indexes [I>=2σ (I)]	R1 = 0.0495, wR2 = 0.1388
Final R indexes [all data]	R1 = 0.0561, wR2 = 0.1447
Largest diff. peak/hole / e Å-3	0.35/-0.51

9. NMR spectra

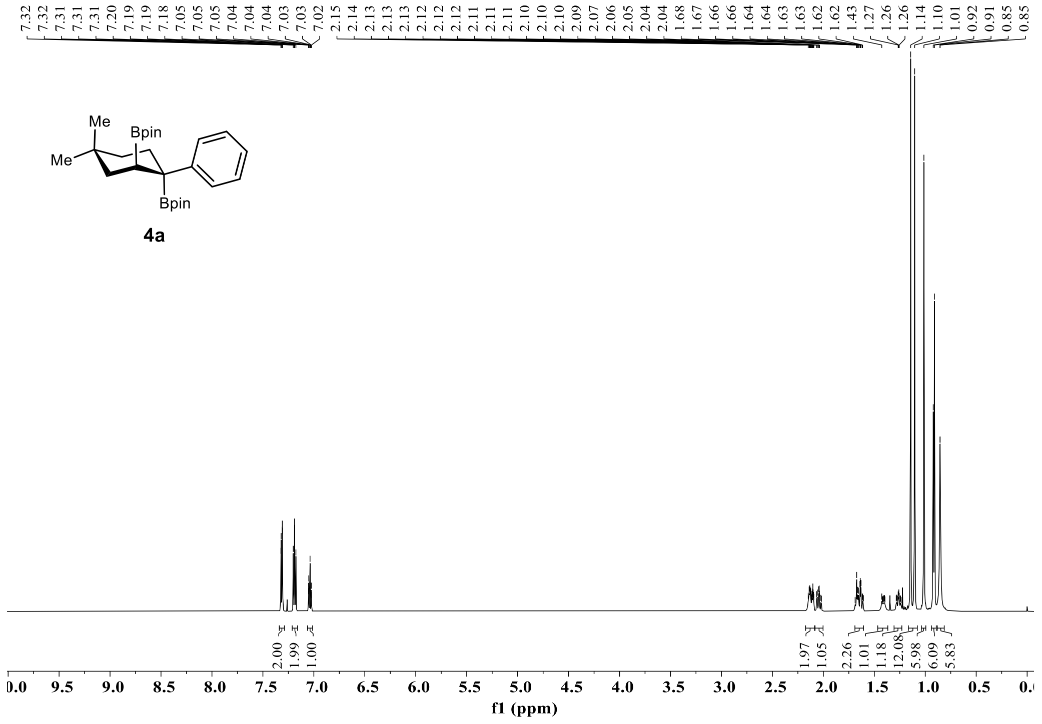

^{1}H NMR (600 MHz, CDCl₃)

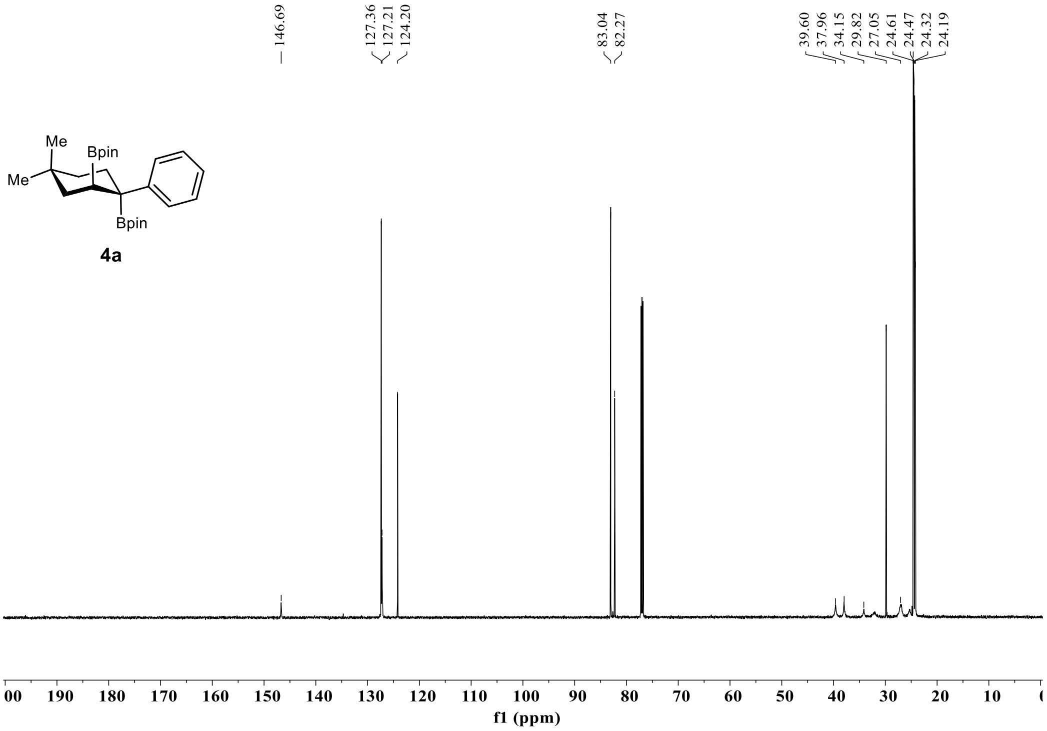

^{13}C NMR (151 MHz, CDCl₃)

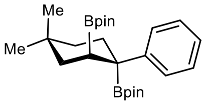

4a

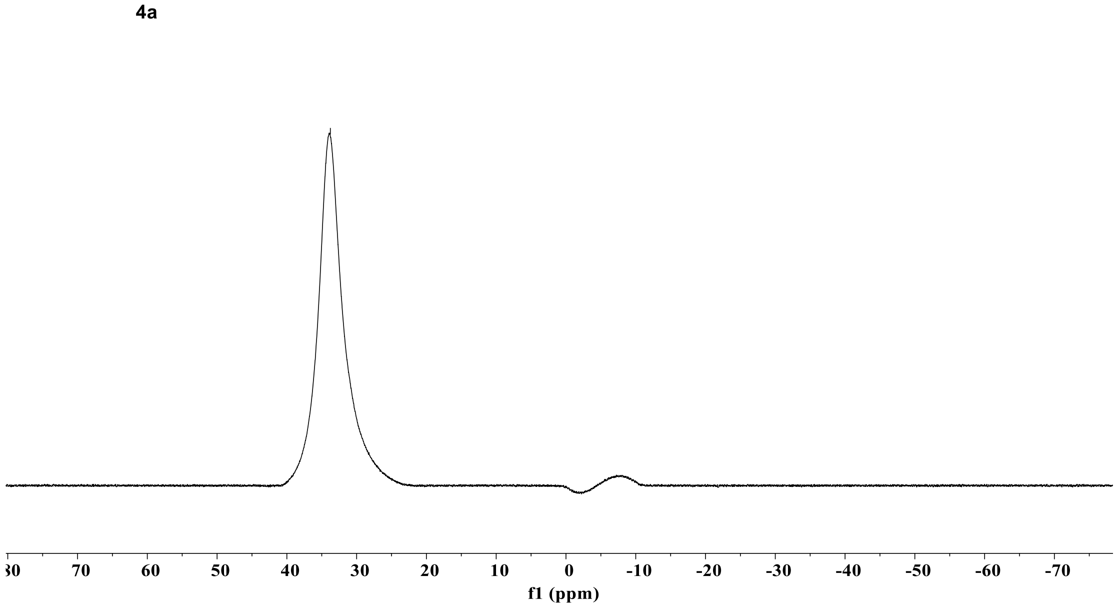

^{11}B NMR (193 MHz, CDCl$_3$)

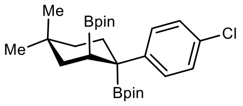

4b

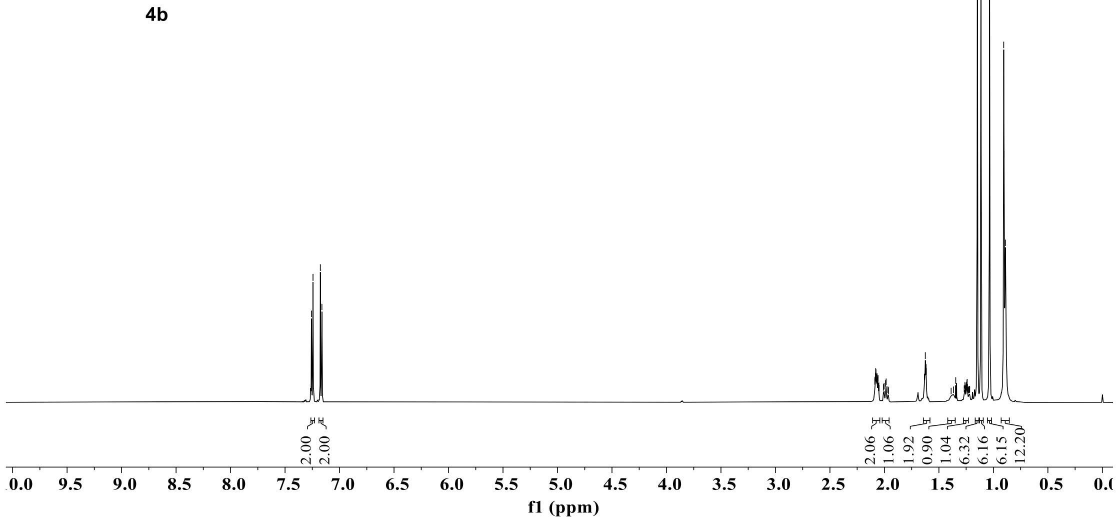

^{1}H NMR (600 MHz, CDCl$_3$)

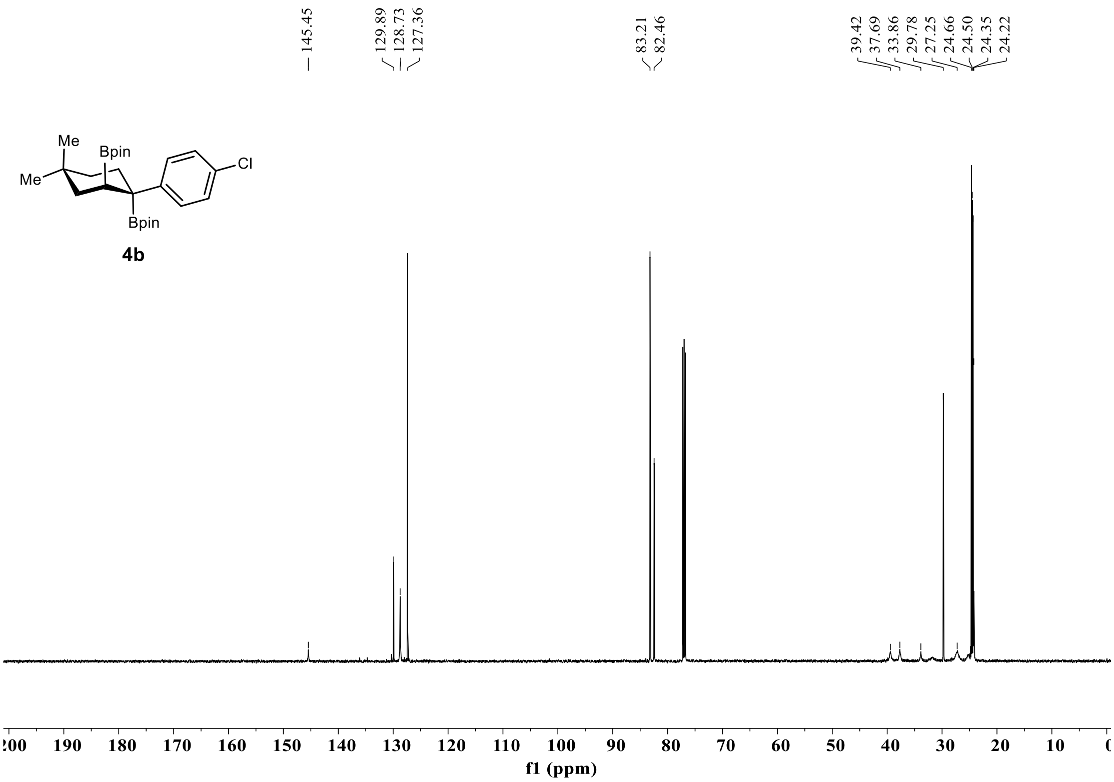

^{13}C NMR (151 MHz, $CDCl_3$)

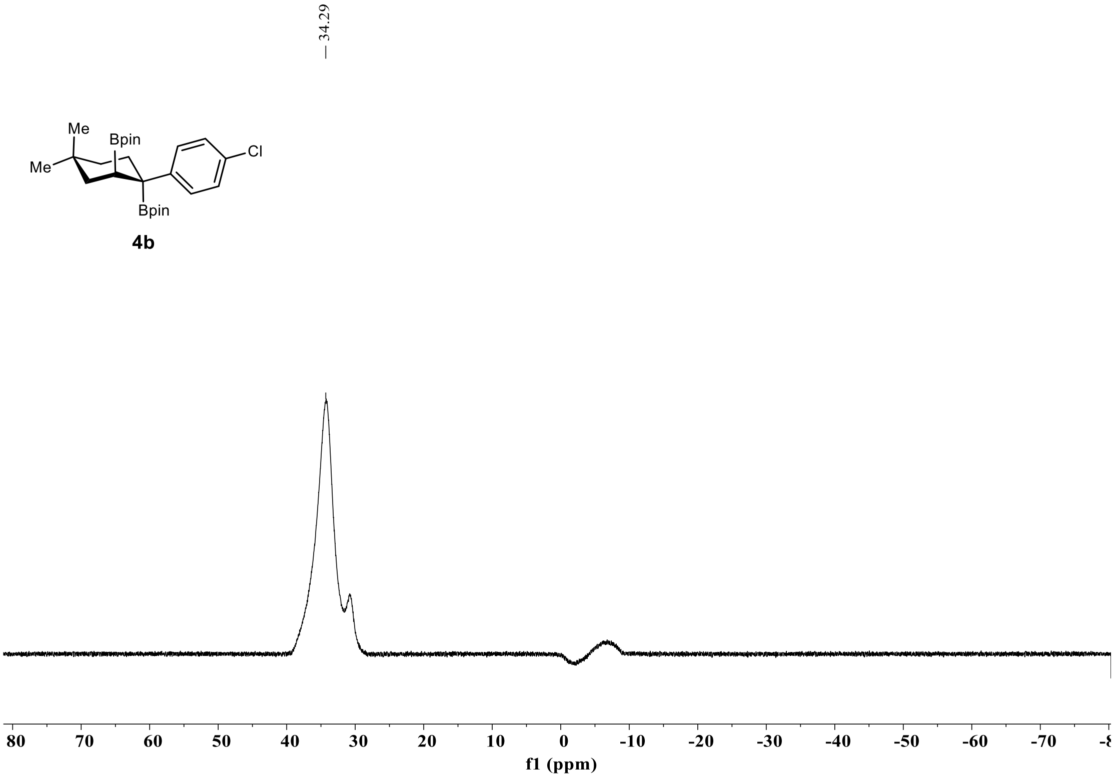

^{11}B NMR (193 MHz, $CDCl_3$)

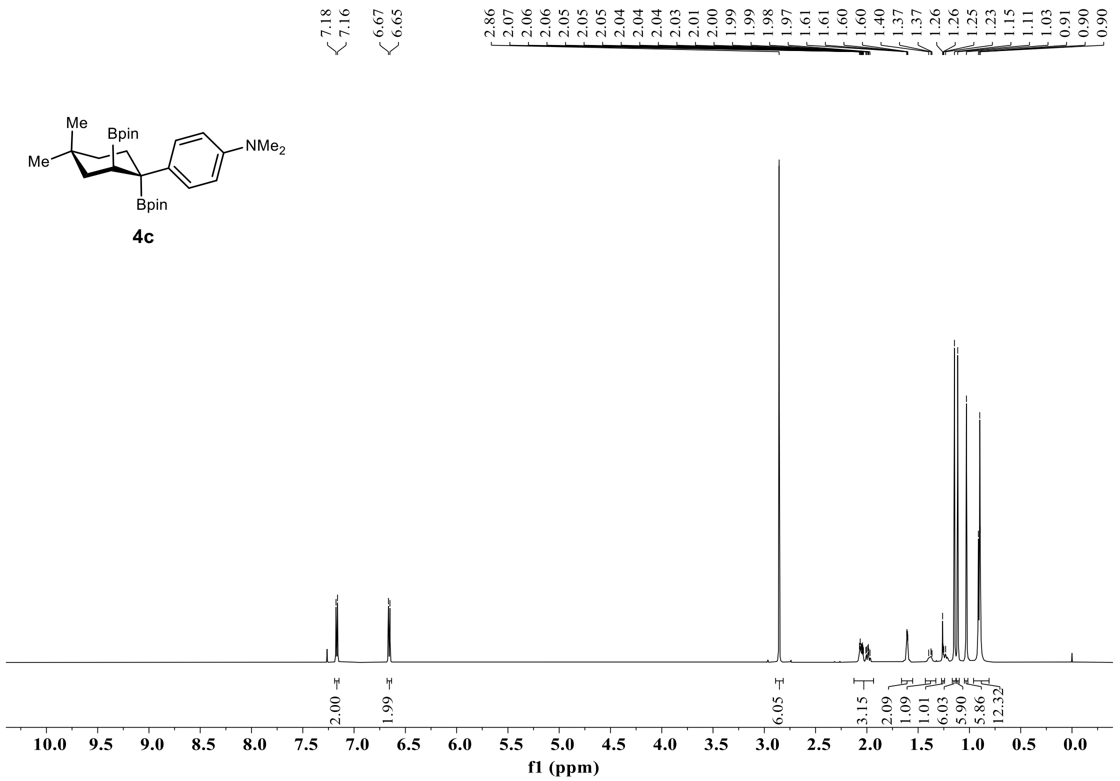

^{1}H NMR (600 MHz, CDCl$_3$)

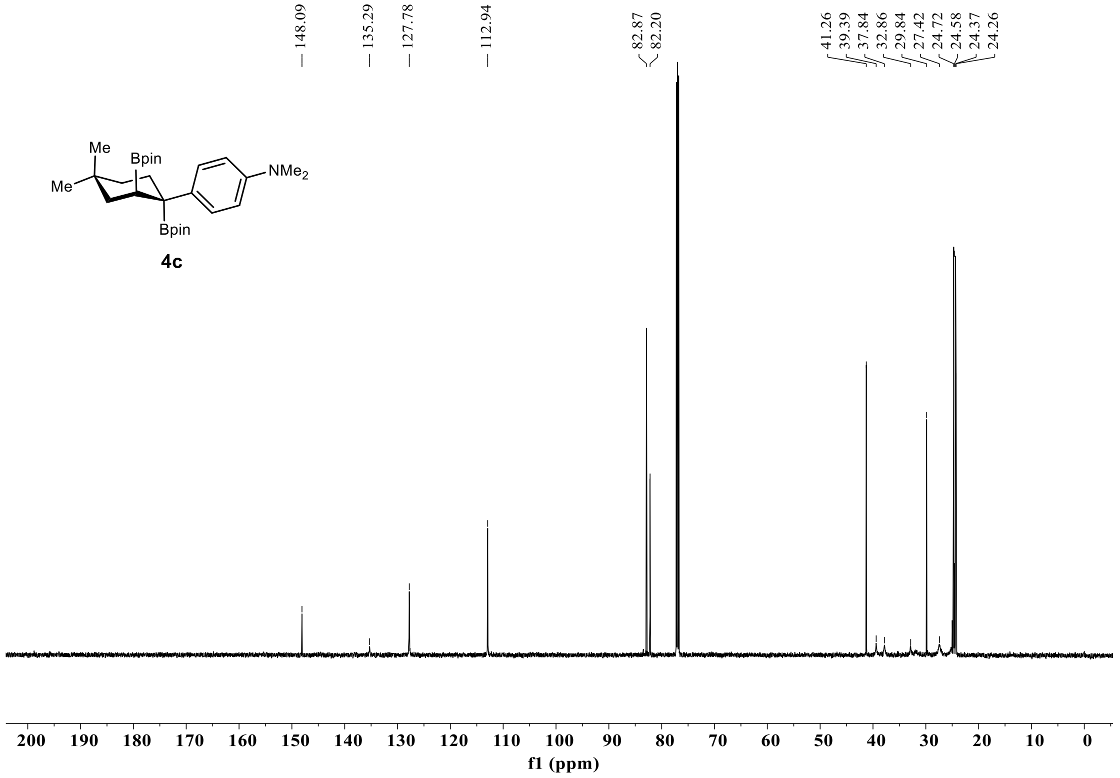

^{13}C NMR (151 MHz, CDCl$_3$)

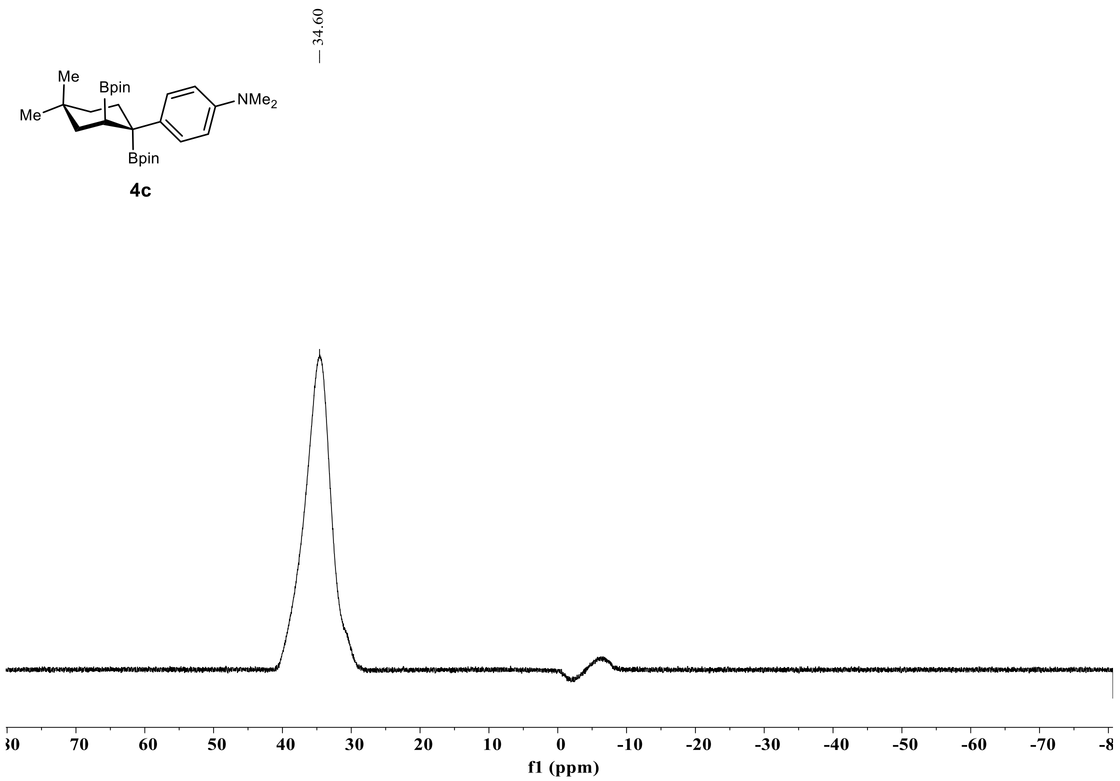

4c

^{11}B NMR (193 MHz, CDCl$_3$)

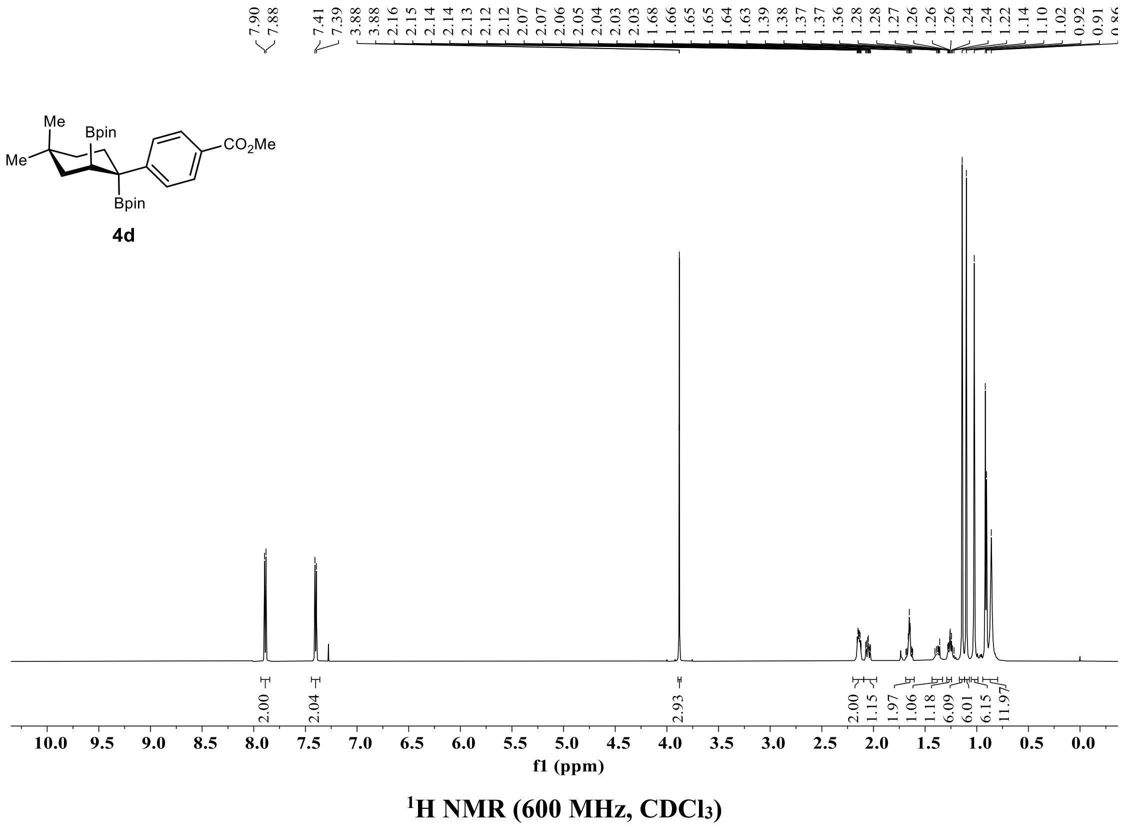

4d

^{1}H NMR (600 MHz, CDCl$_3$)

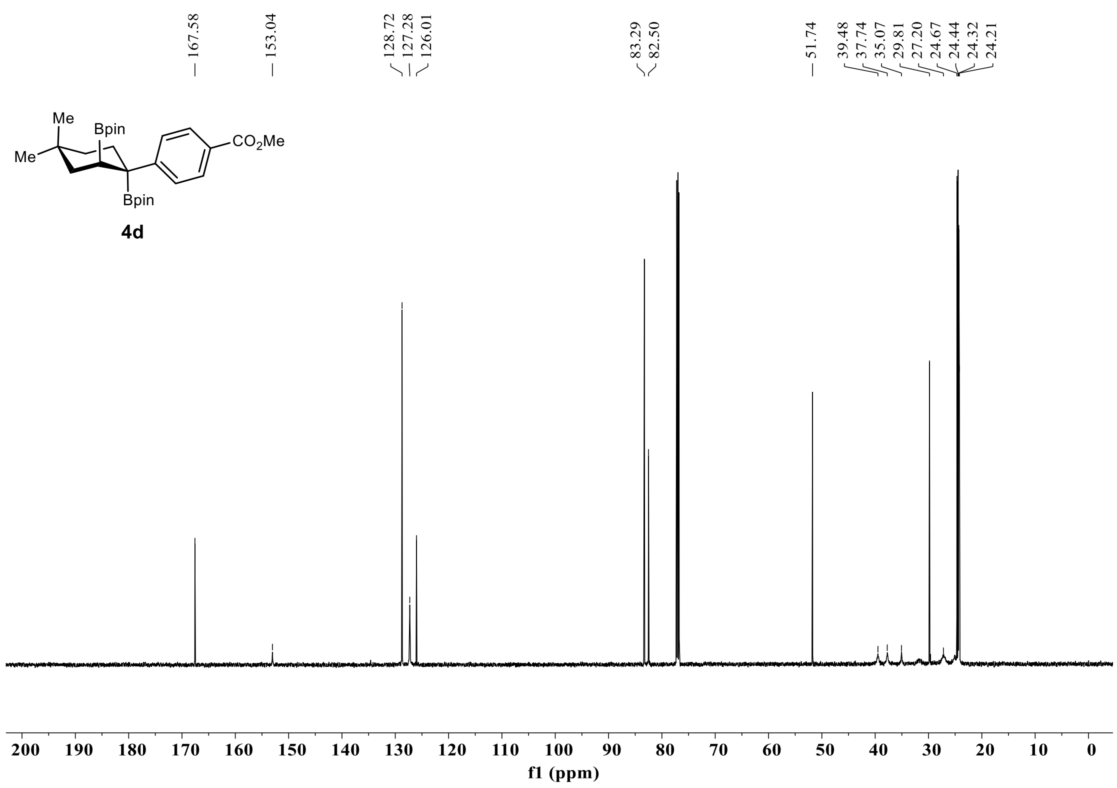

^{13}C NMR (151 MHz, CDCl3)

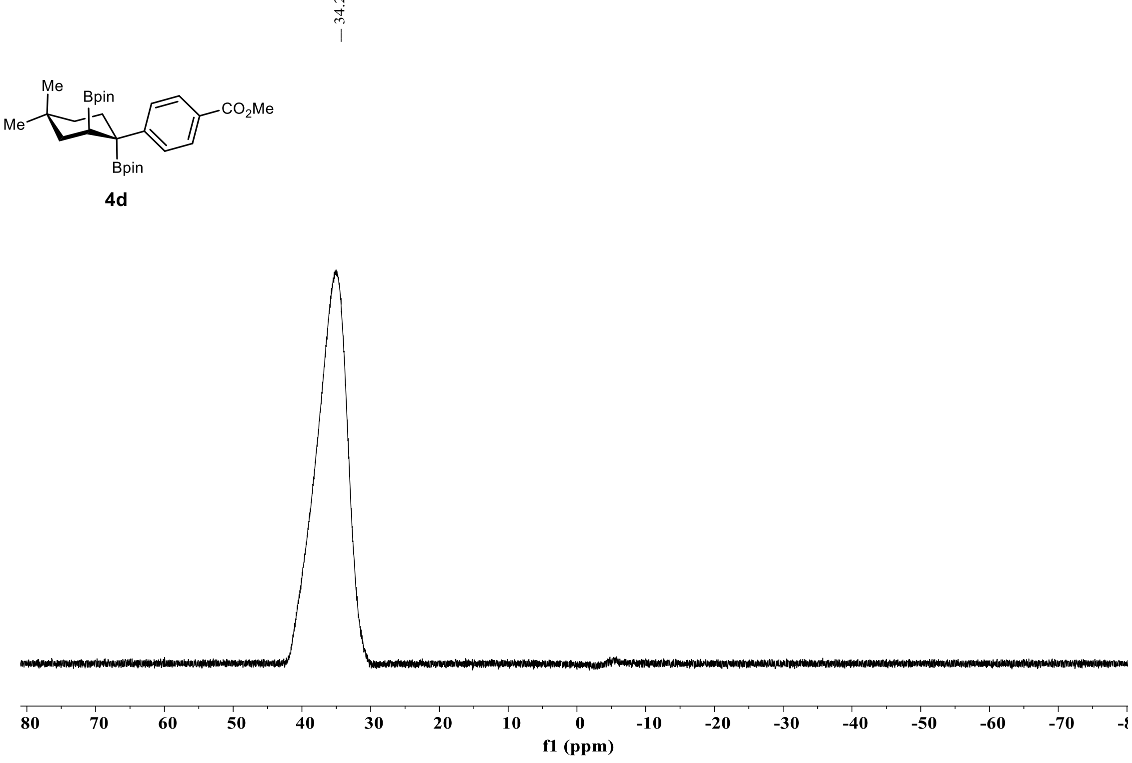

^{11}B NMR (193 MHz, CDCl3)

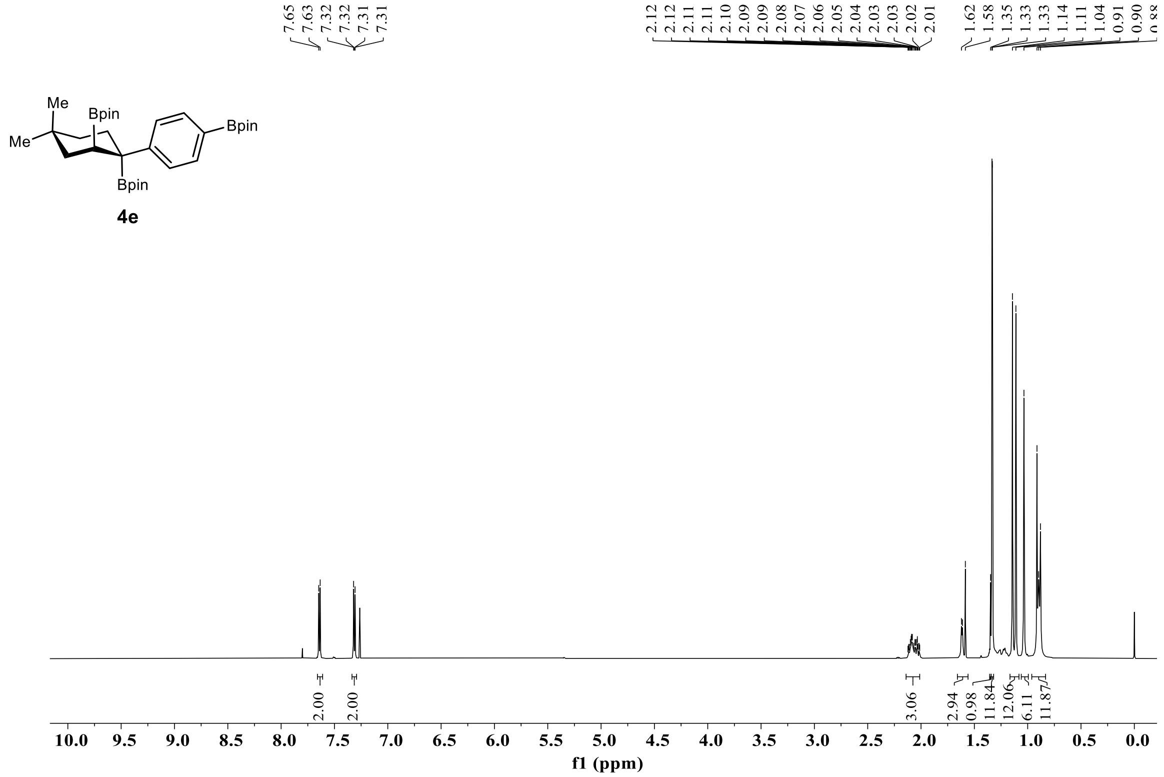

^{1}H NMR (600 MHz, $CDCl_3$)

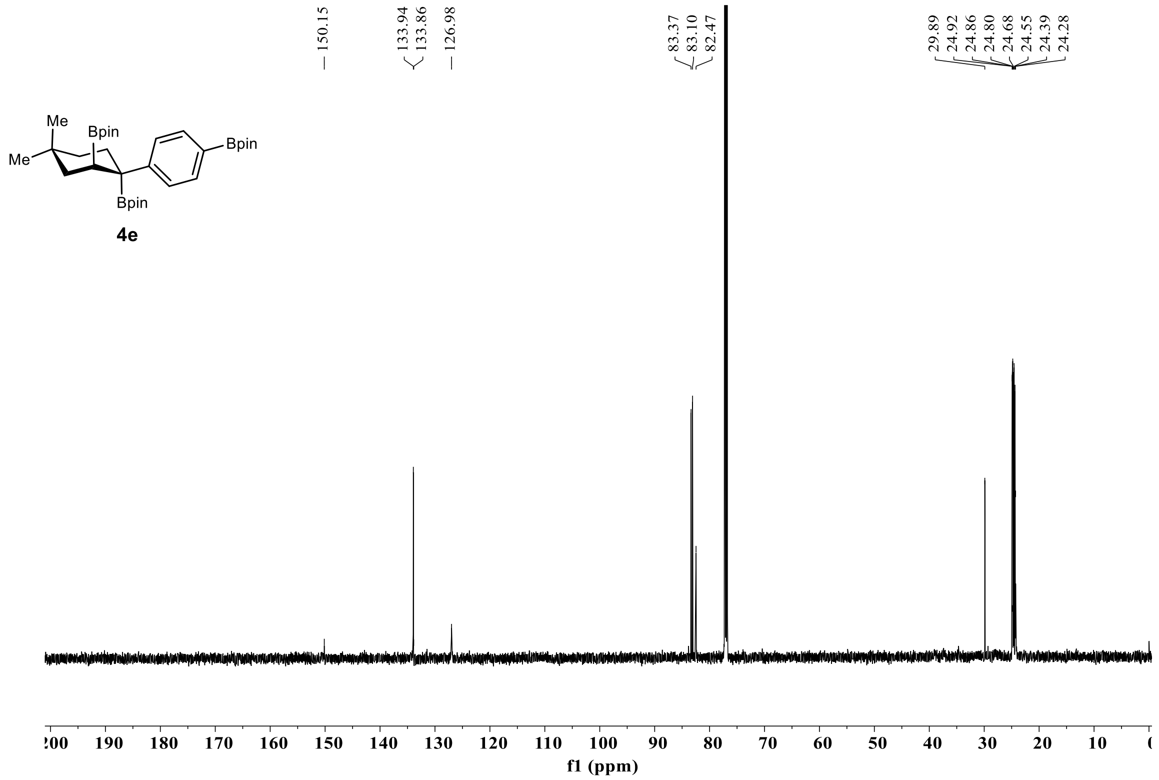

^{13}C NMR (151 MHz, $CDCl_3$)

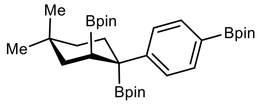

4e

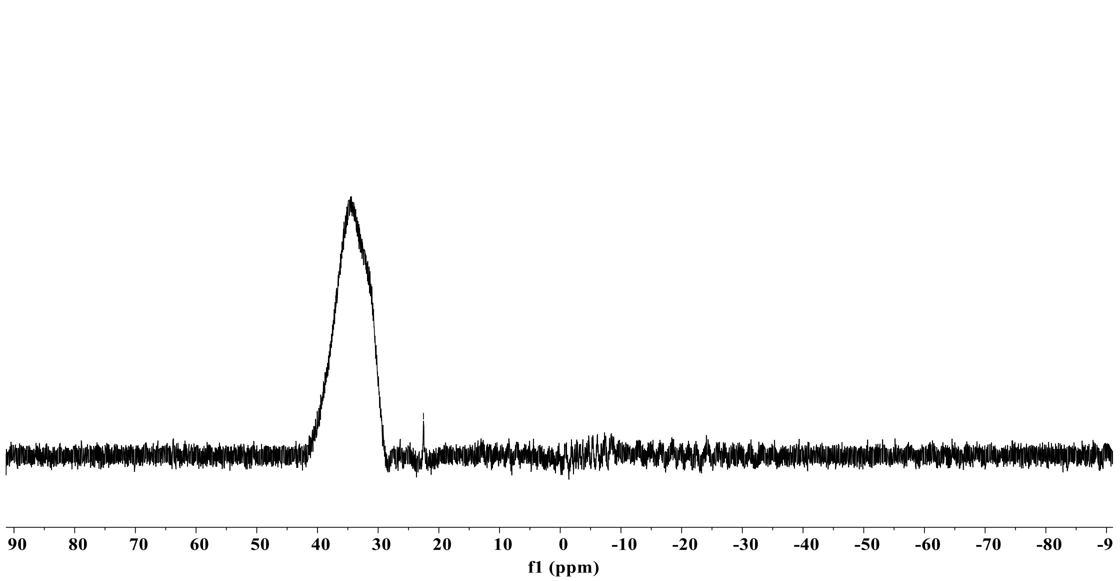

^{11}B NMR (193 MHz, CDCl$_3$)

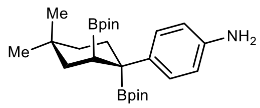

4f

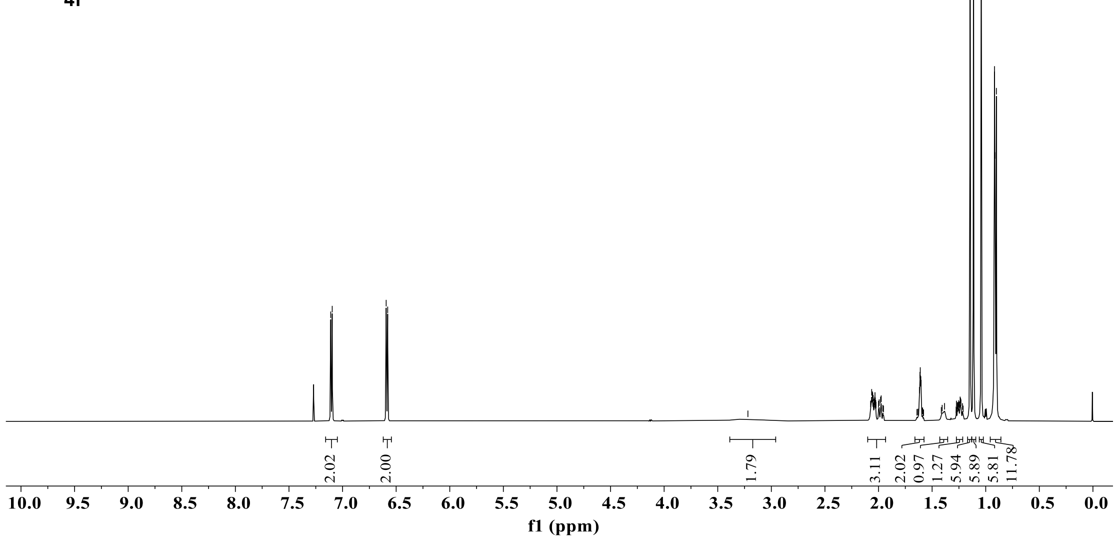

^{1}H NMR (600 MHz, CDCl$_3$)

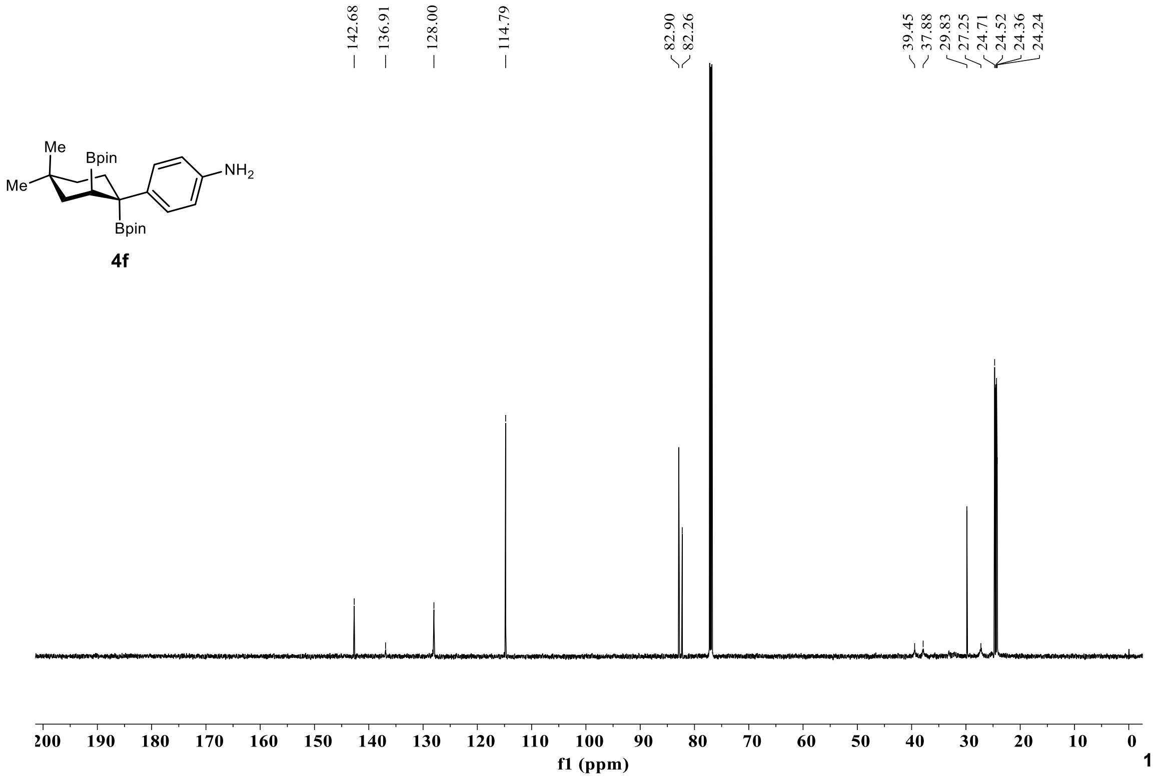

4f

^{13}C NMR (151 MHz, CDCl$_3$)

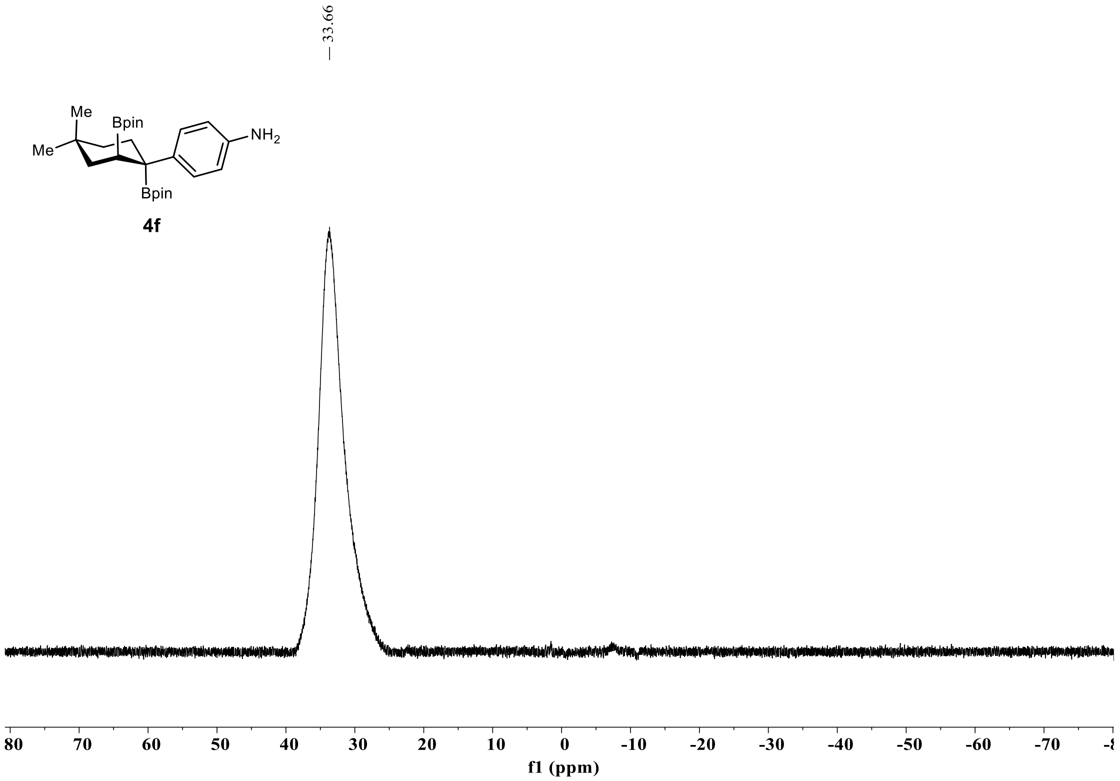

4f

^{11}B NMR (193 MHz, CDCl$_3$)

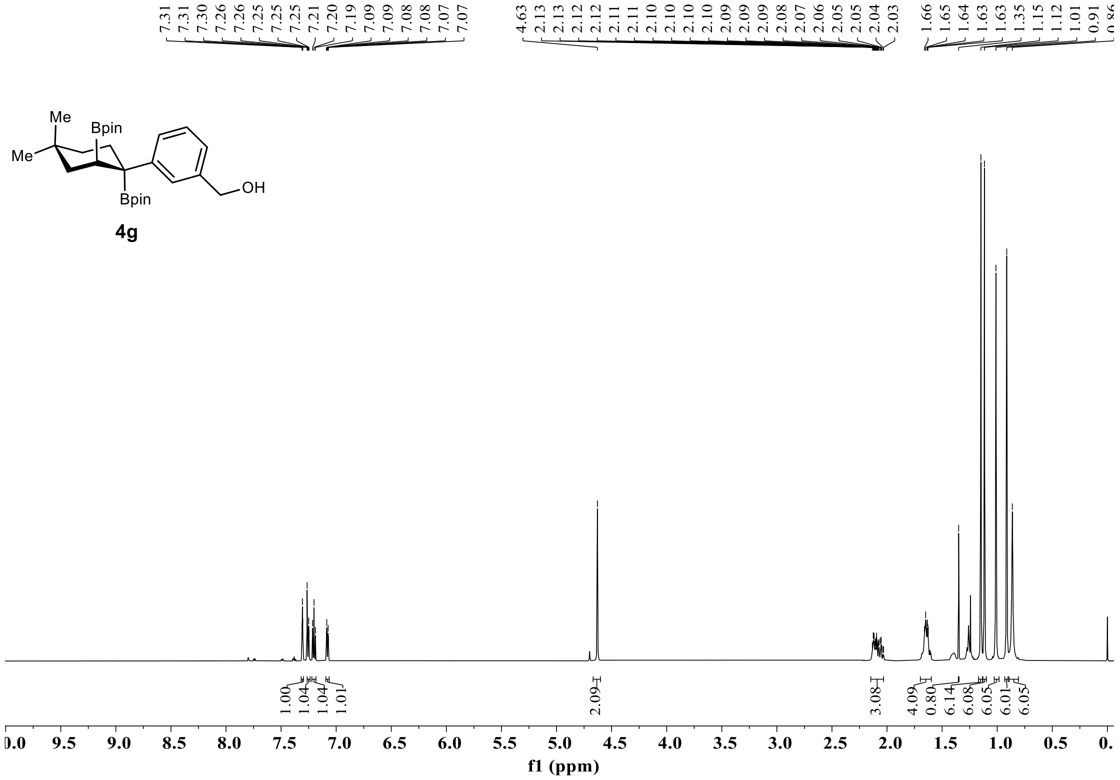

^{1}H NMR (600 MHz, $CDCl_3$)

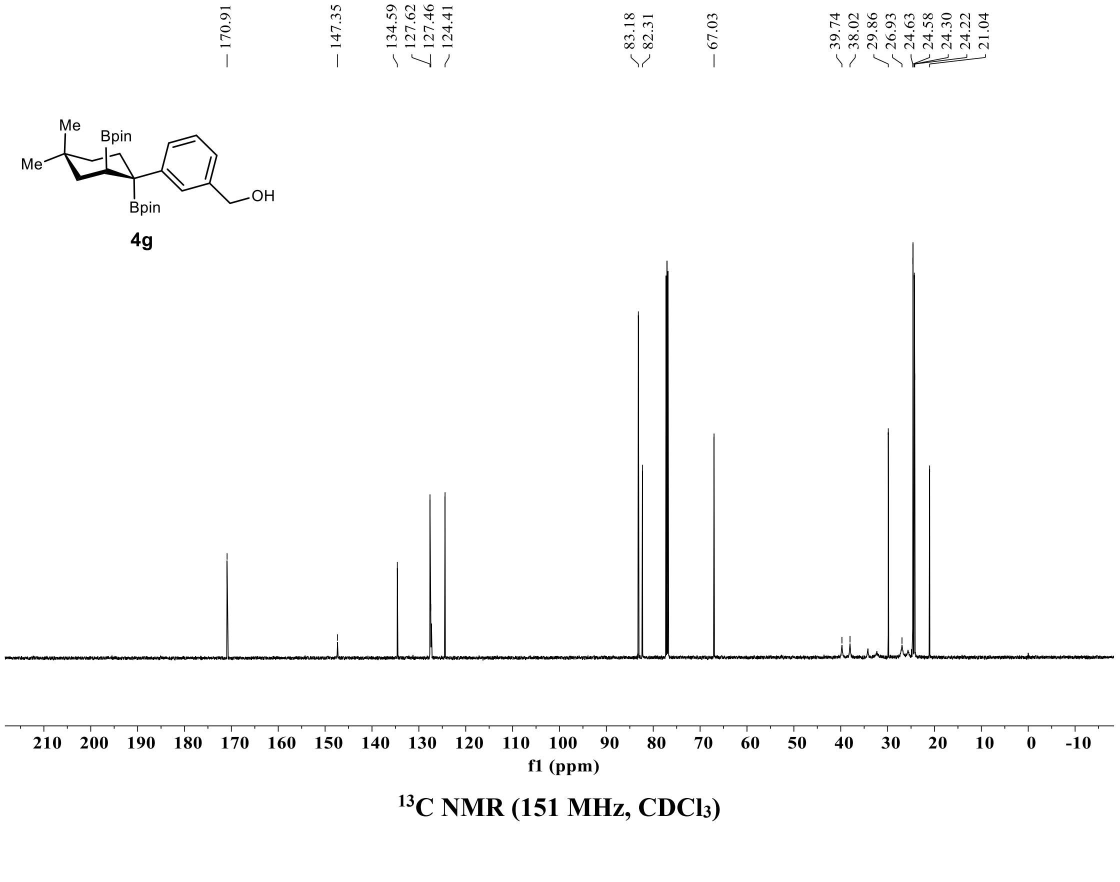

^{13}C NMR (151 MHz, $CDCl_3$)

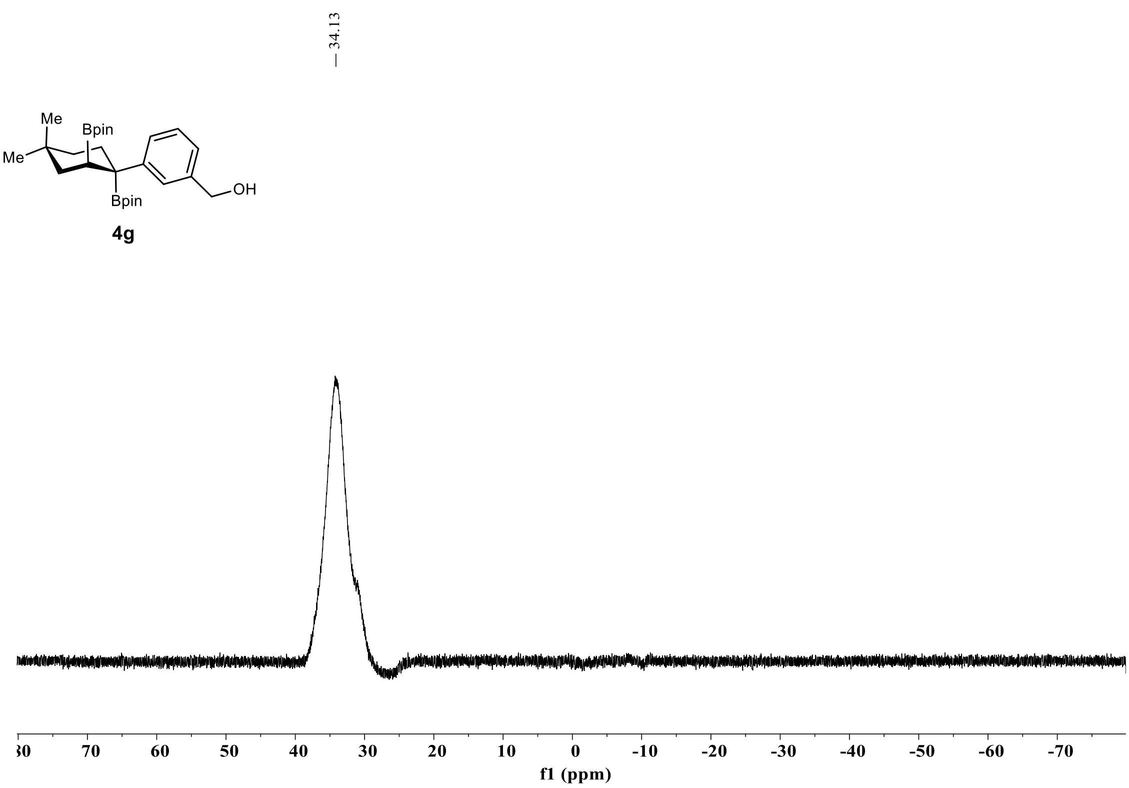

^{11}B NMR (193 MHz, $CDCl_3$)

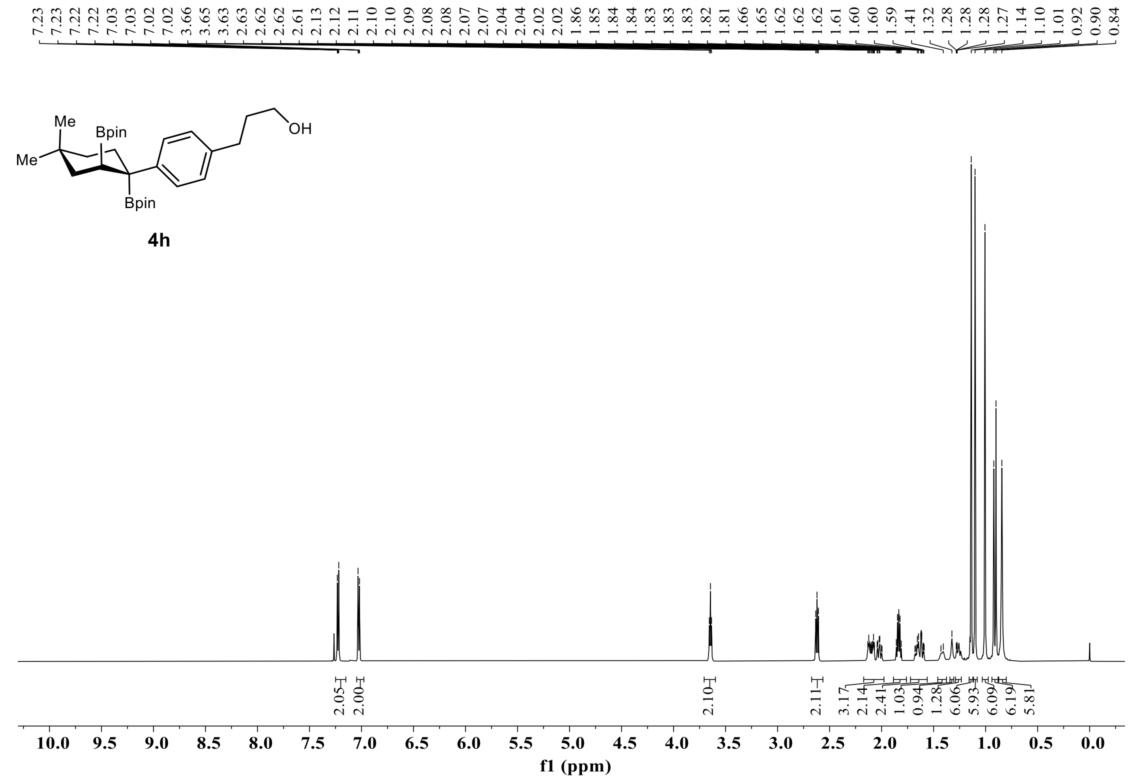

^{1}H NMR (600 MHz, $CDCl_3$)

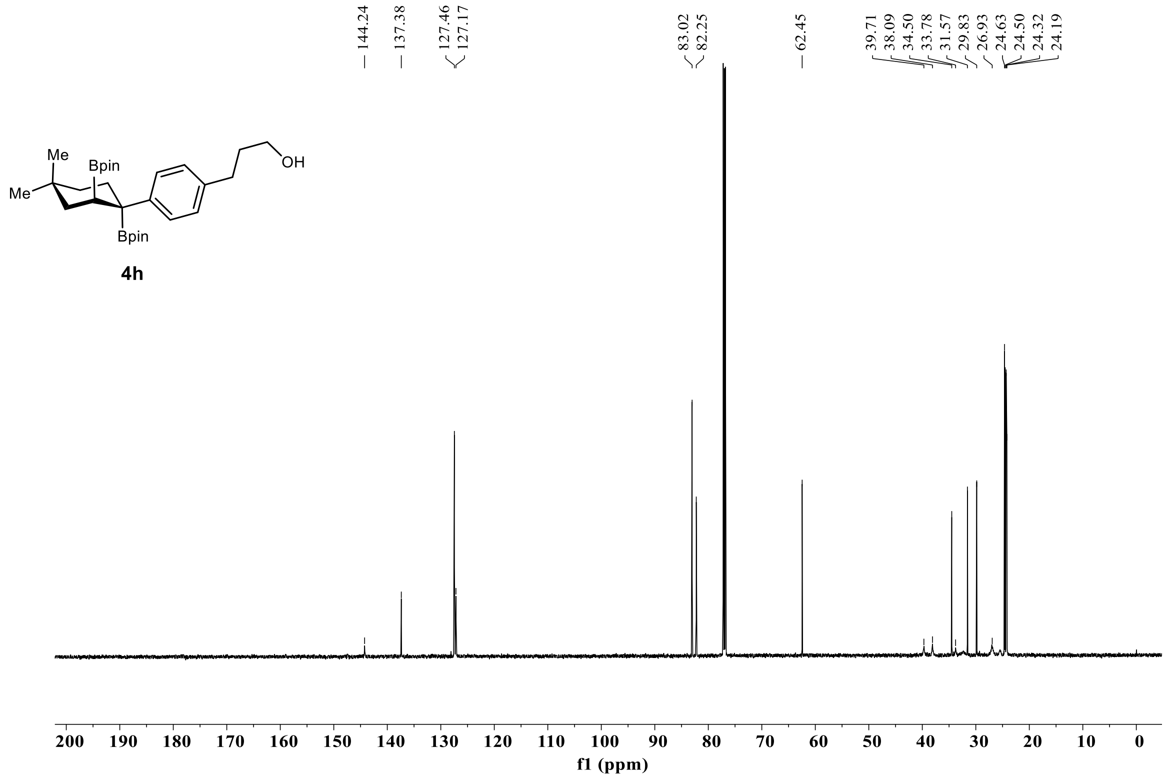

^{13}C NMR (151 MHz, $CDCl_3$)

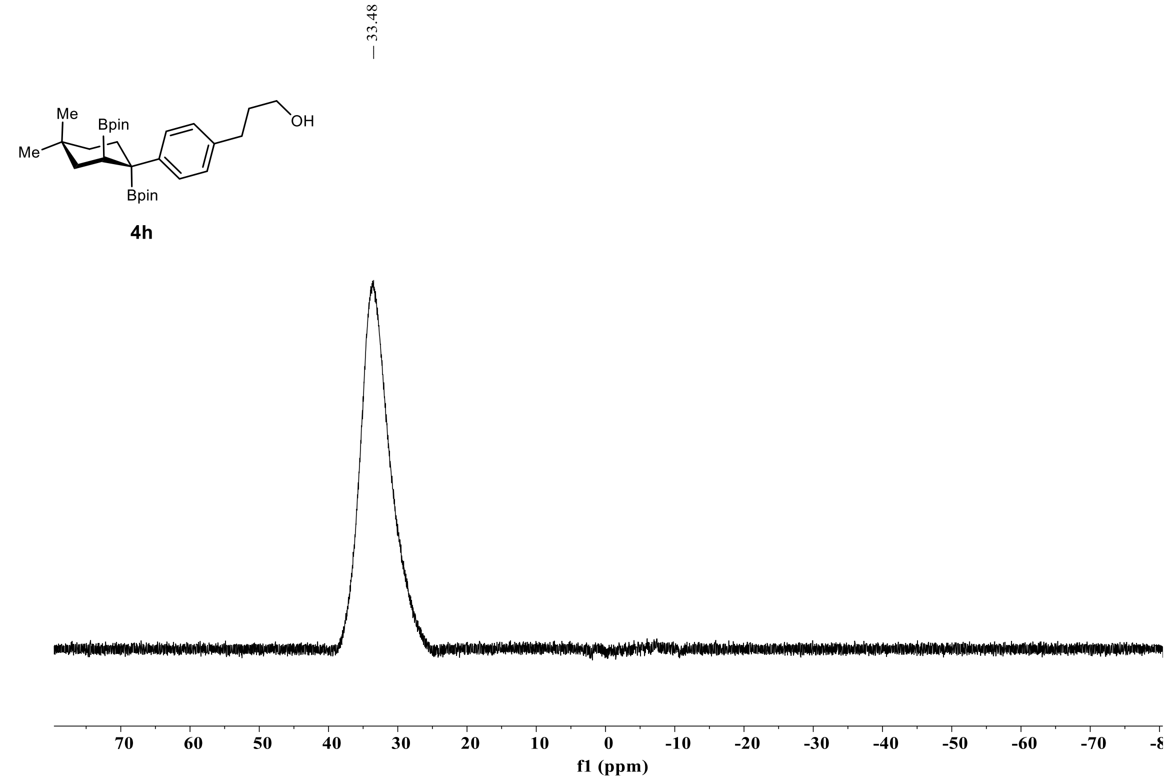

^{11}B NMR (193 MHz, $CDCl_3$)

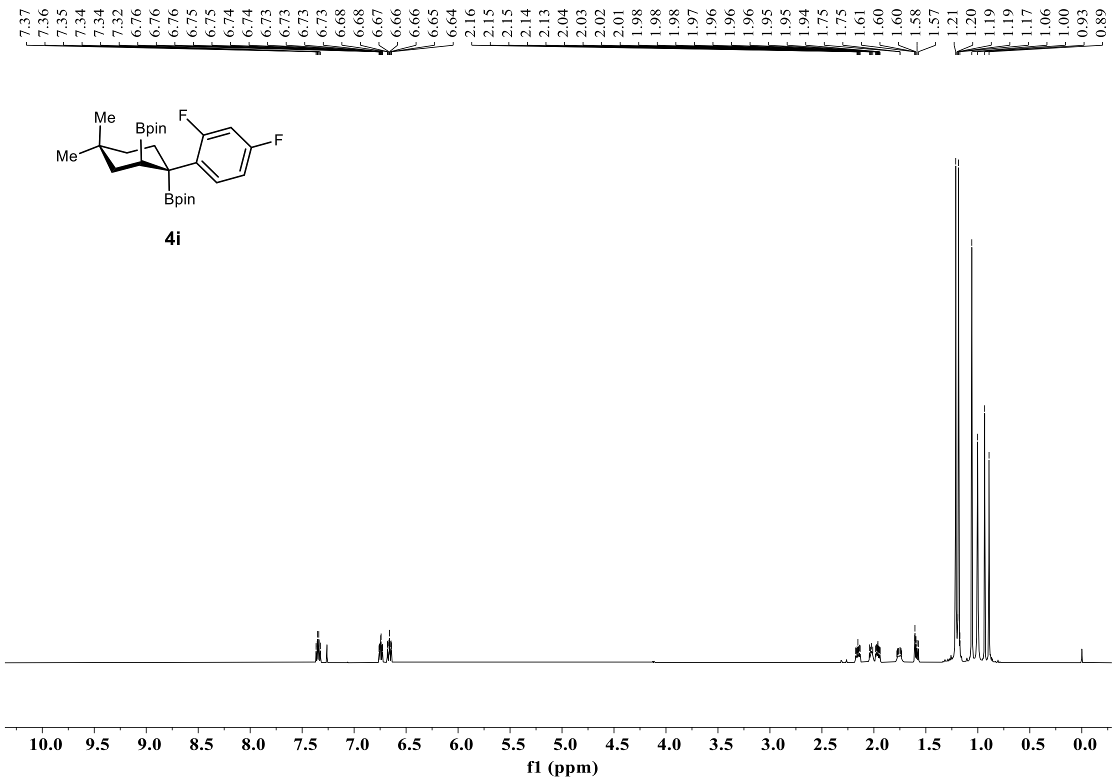

^{1}H NMR (600 MHz, CDCl3)

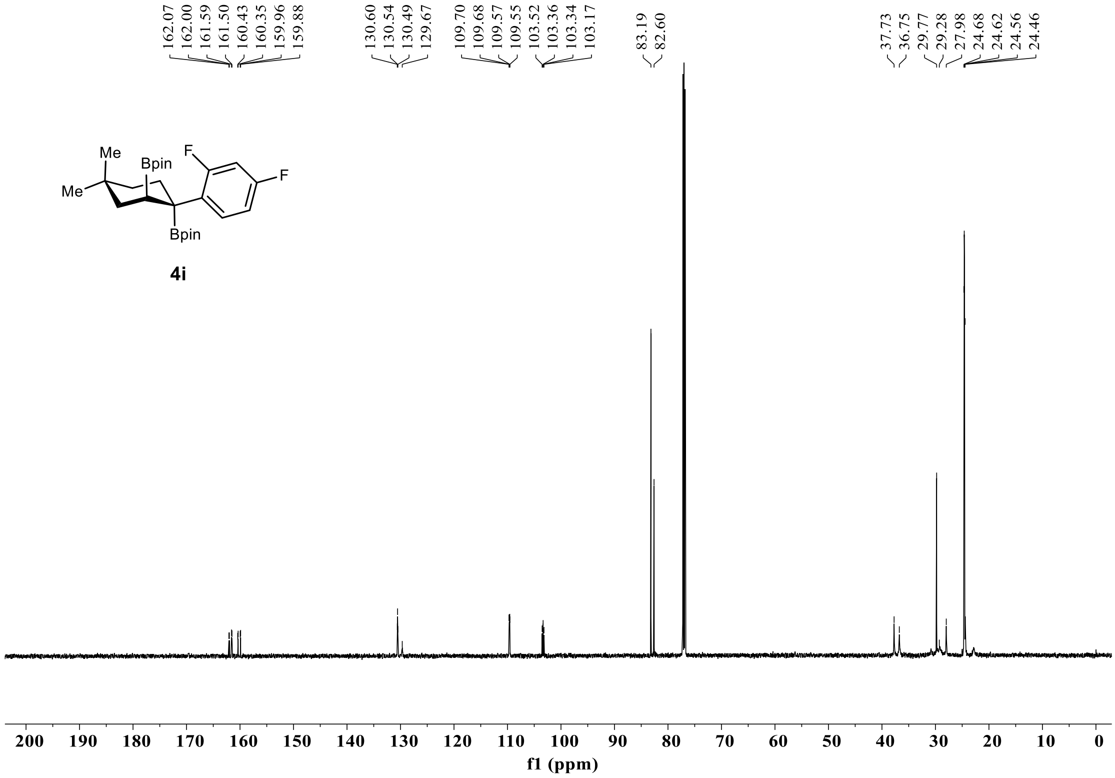

^{13}C NMR (151 MHz, CDCl3)

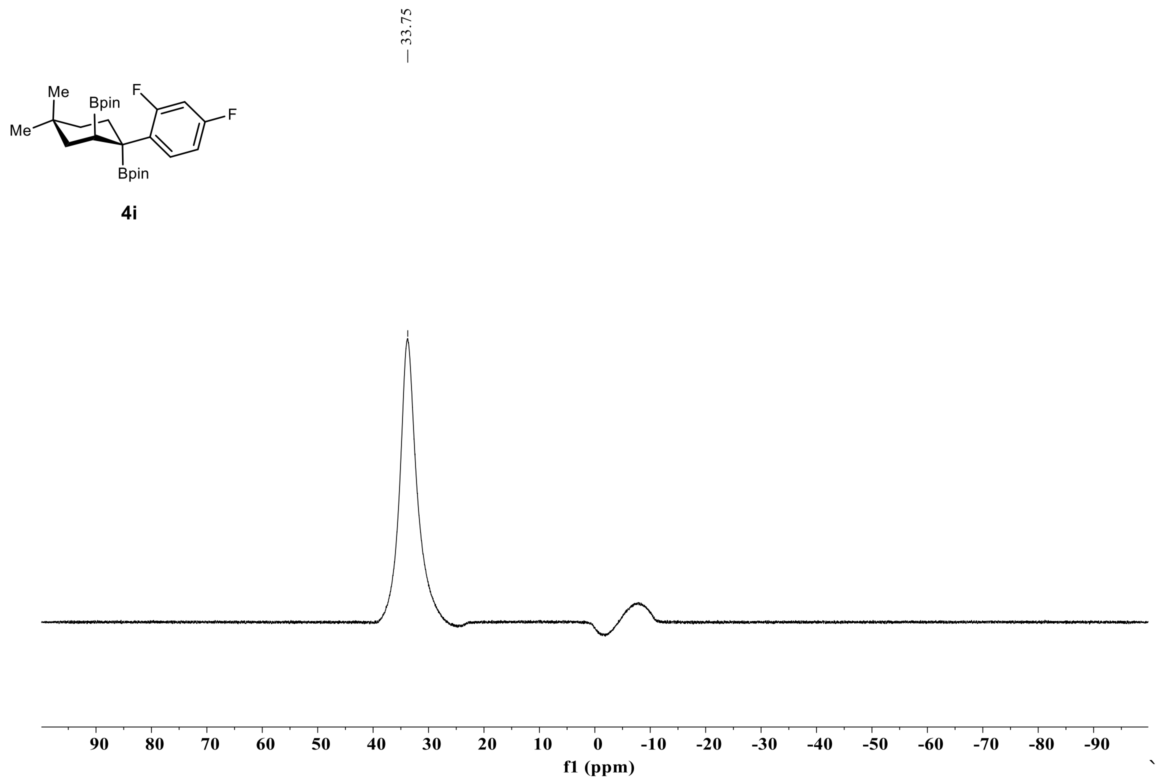

^{11}B NMR (193 MHz, $CDCl_3$)

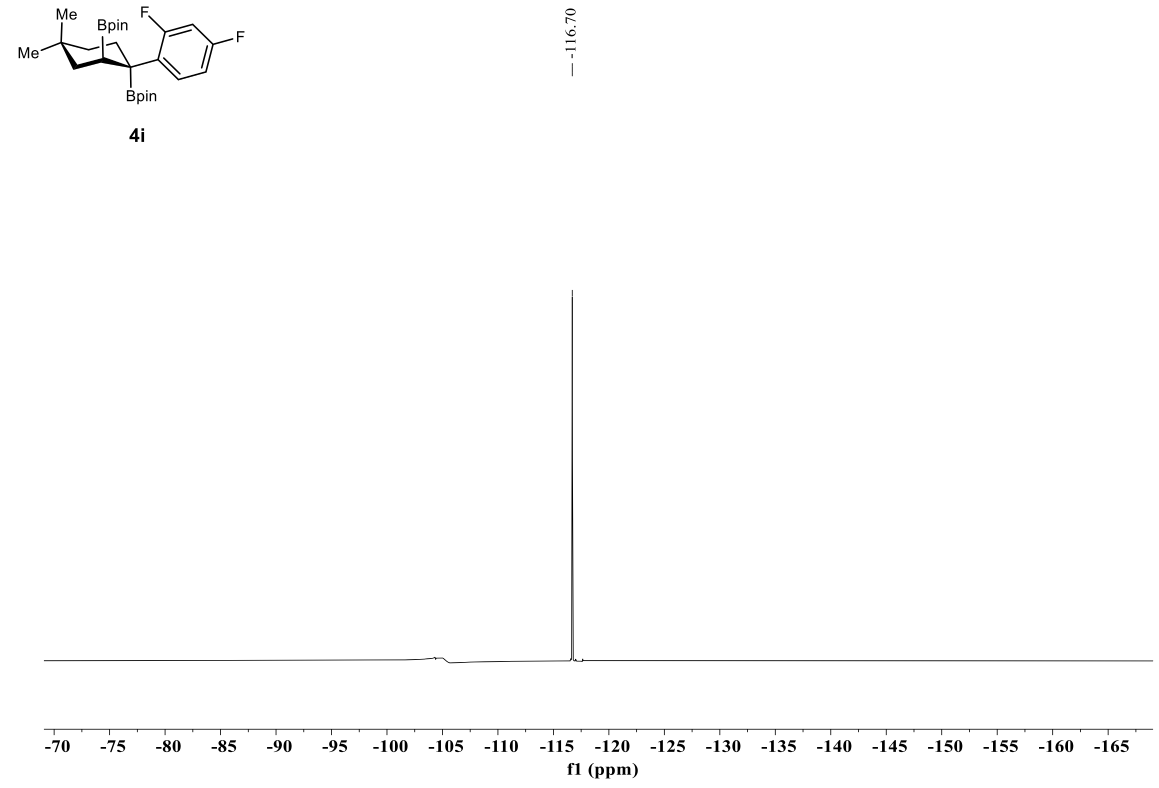

^{19}F NMR (565 MHz, $CDCl_3$)

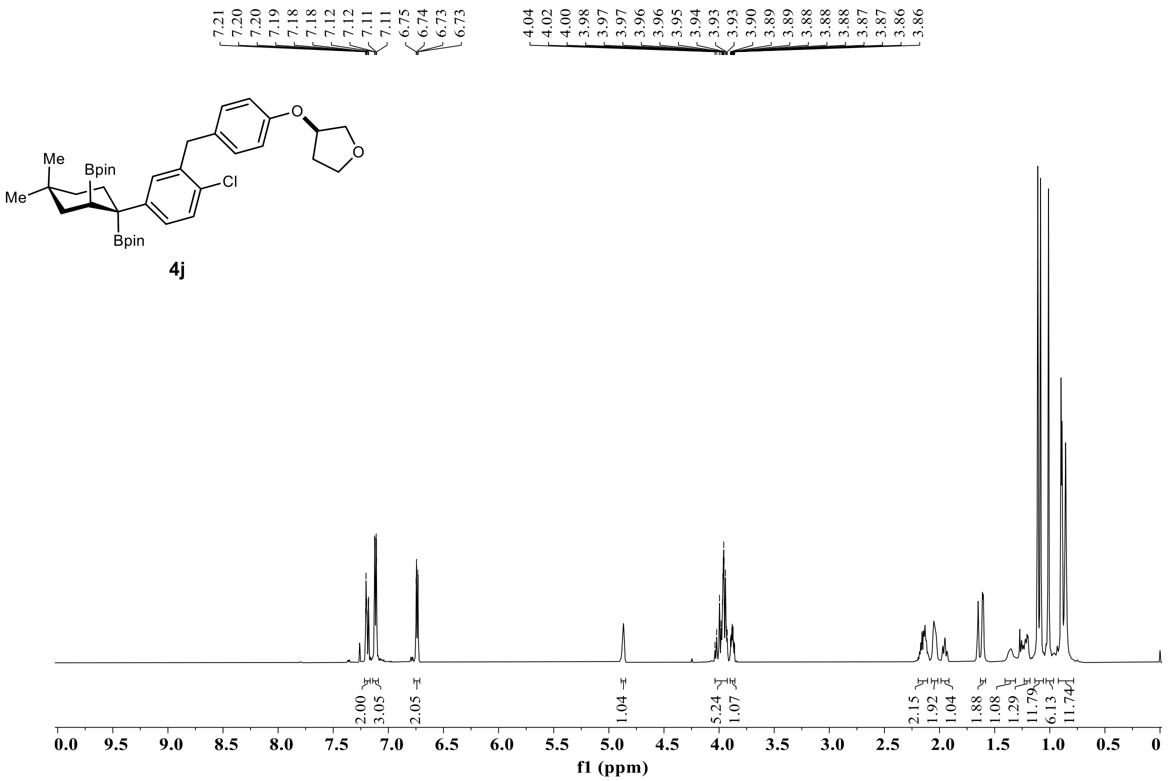

^{1}H NMR (600 MHz, CDCl$_3$)

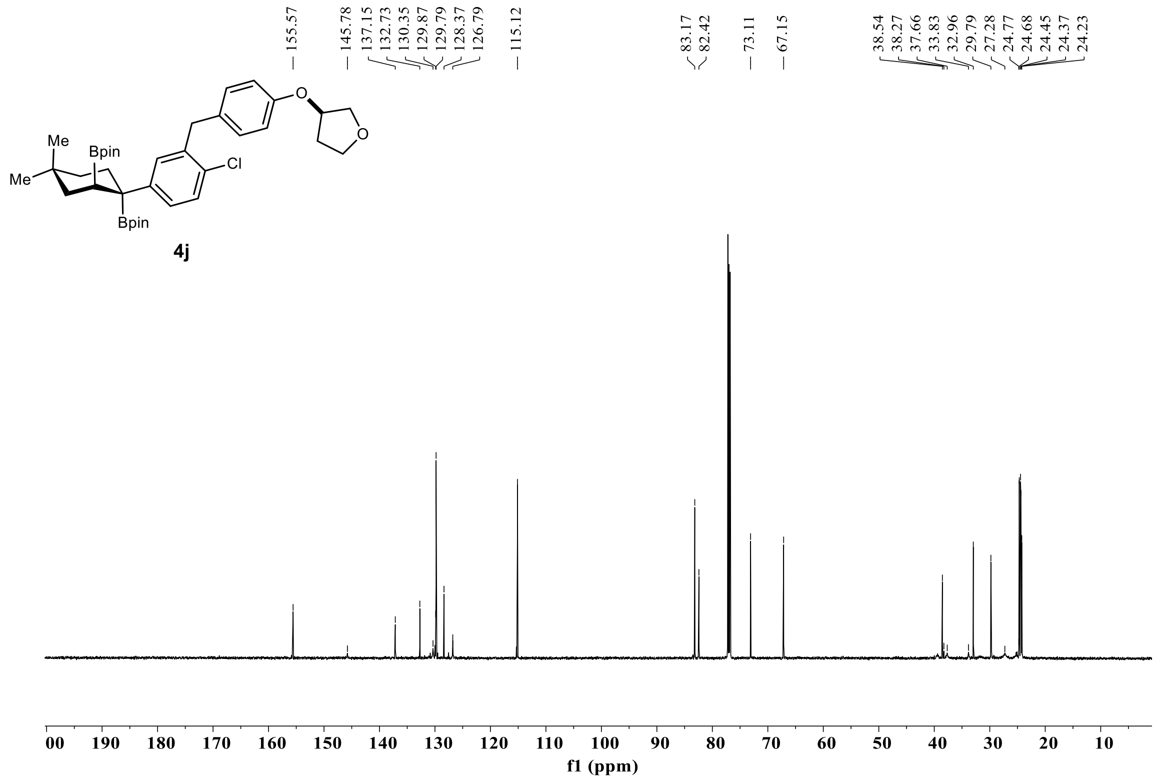

^{13}C NMR (151 MHz, CDCl$_3$)

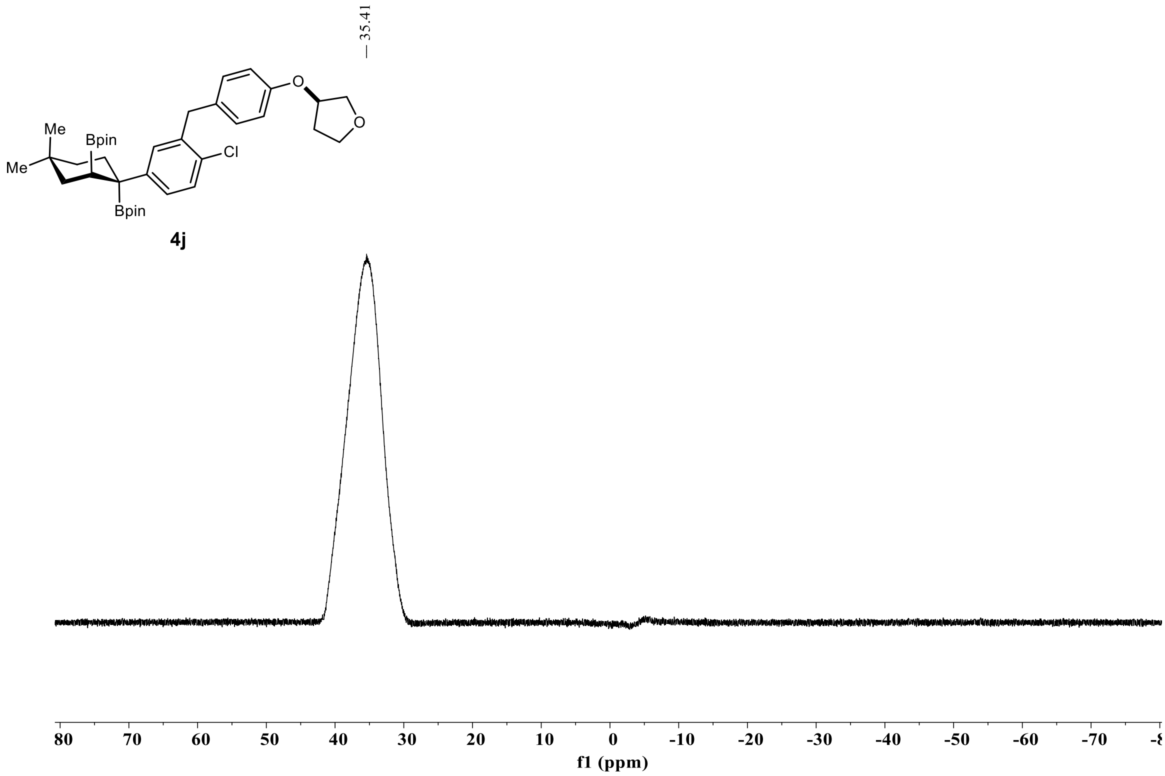

¹¹B NMR (193 MHz, CDCl₃)

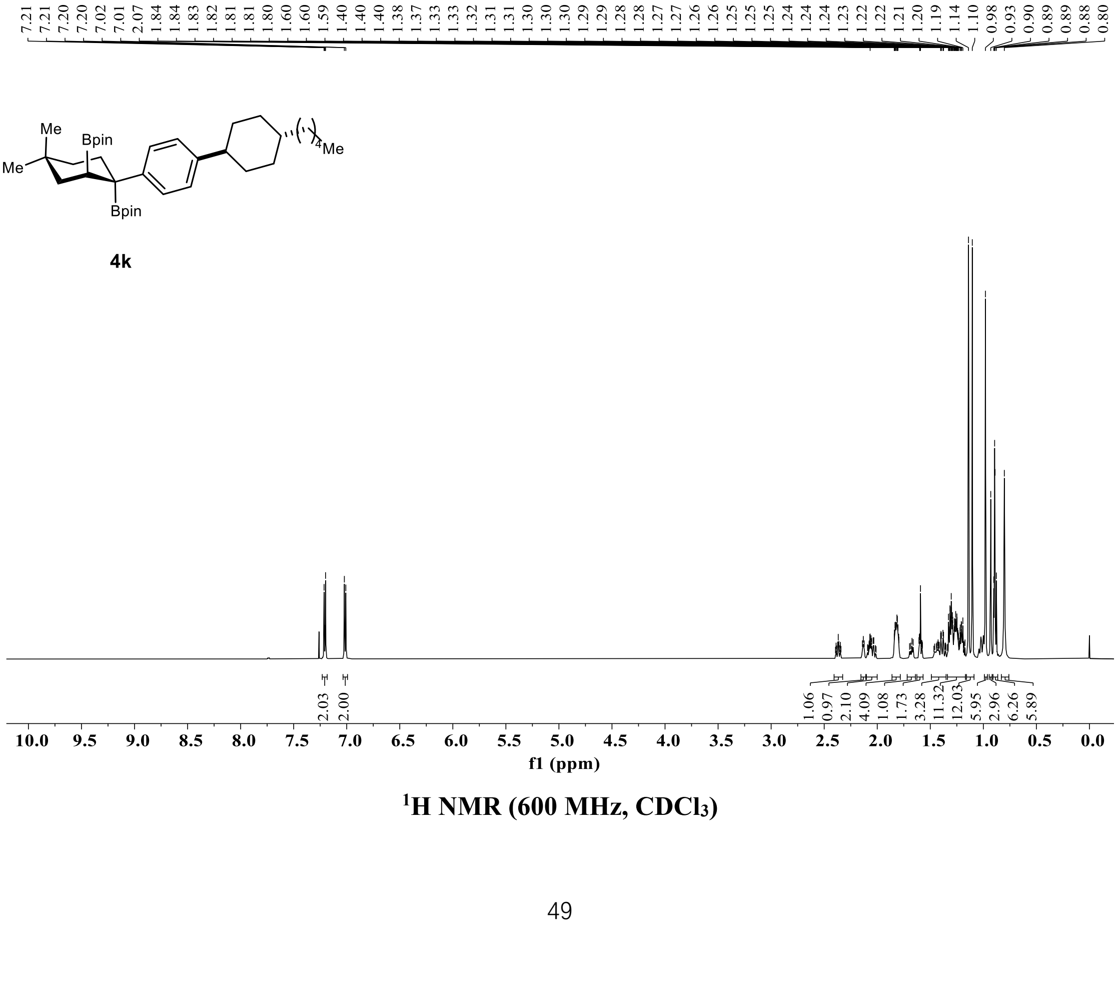

¹H NMR (600 MHz, CDCl₃)

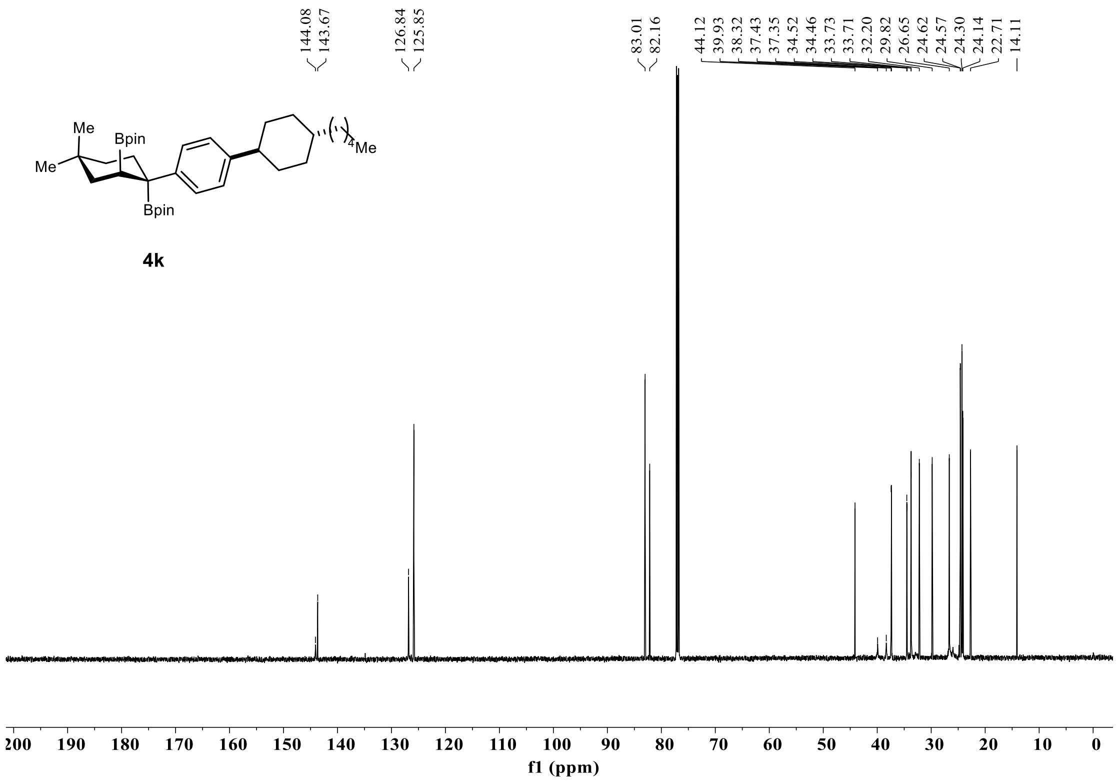

^{13}C NMR (151 MHz, CDCl$_3$)

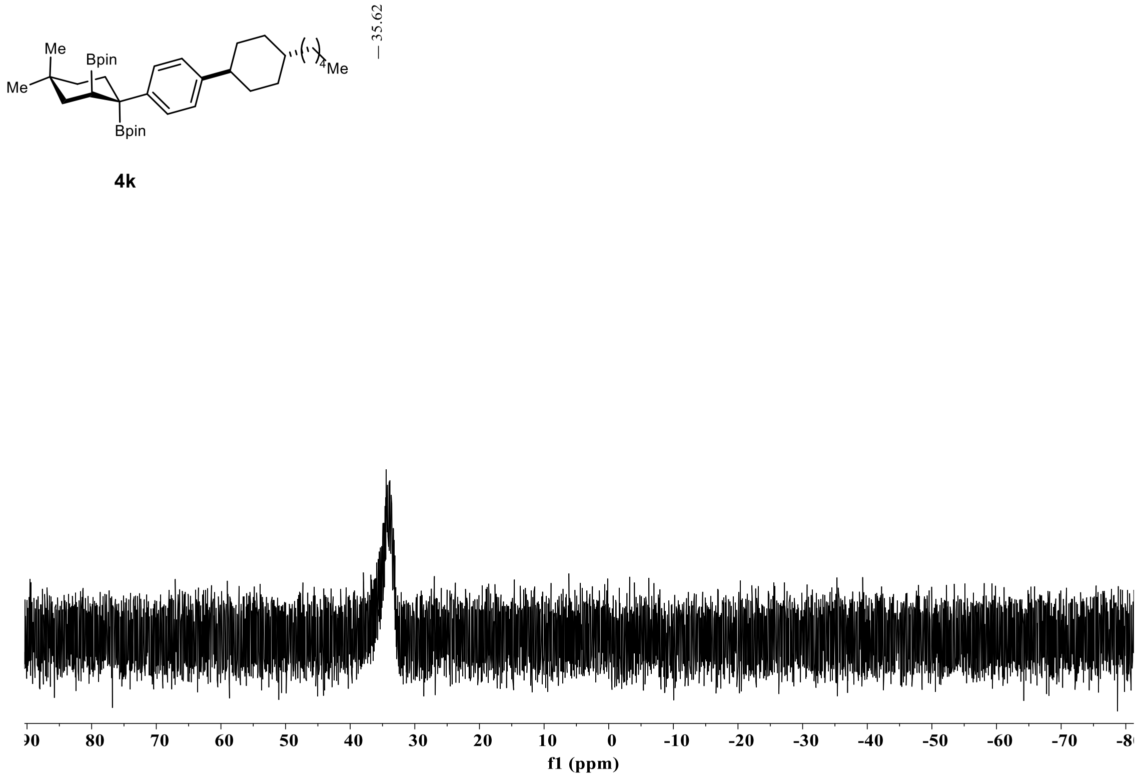

^{11}B NMR (193 MHz, CDCl$_3$)

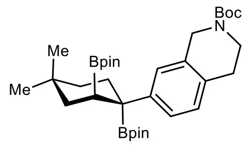

4l

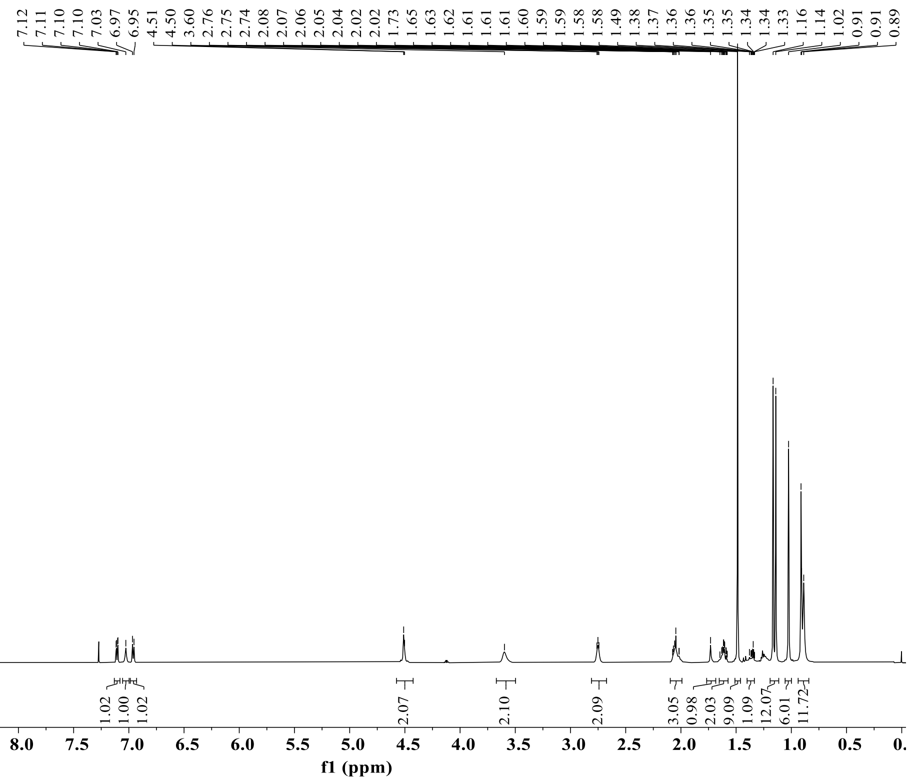

^{1}H NMR (600 MHz, $CDCl_3$)

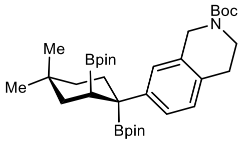

4l

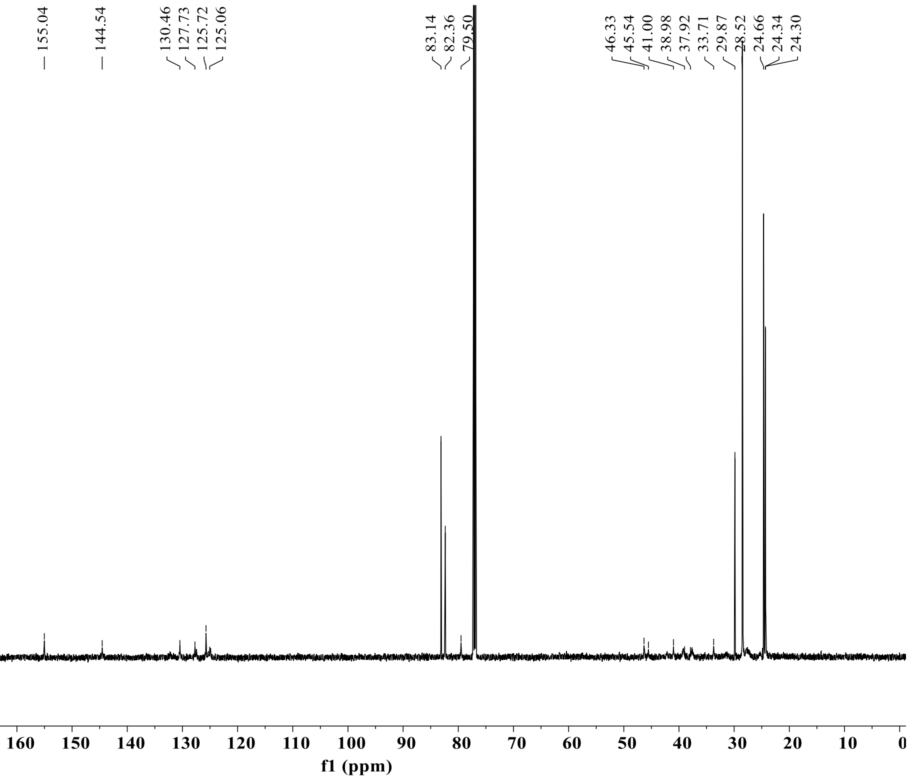

^{13}C NMR (151 MHz, $CDCl_3$)

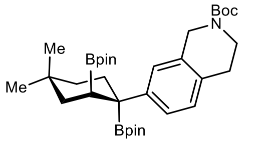

4l

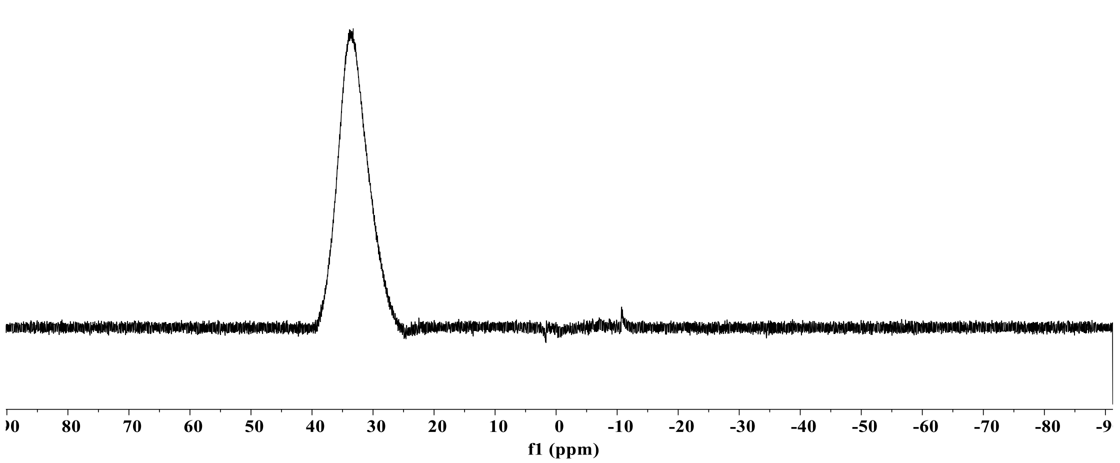

^{11}B NMR (193 MHz, CDCl$_3$)

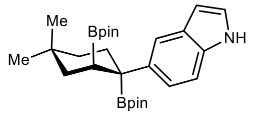

4m

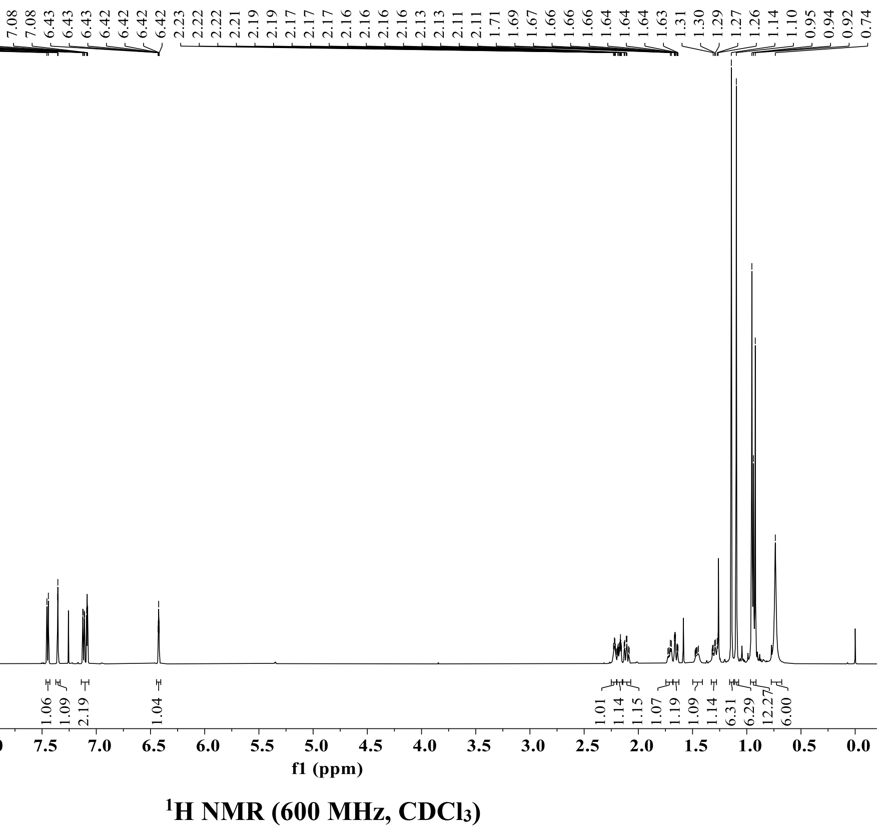

^{1}H NMR (600 MHz, CDCl$_3$)

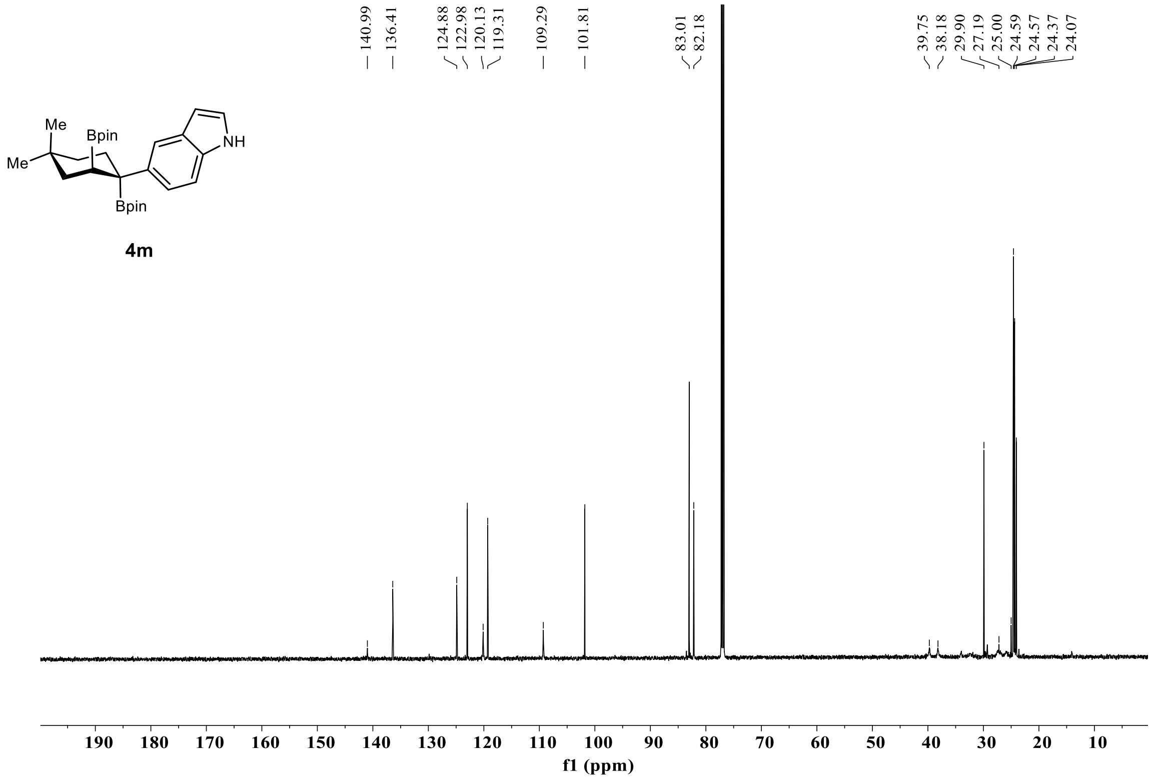

4m

^{13}C NMR (151 MHz, $CDCl_3$)

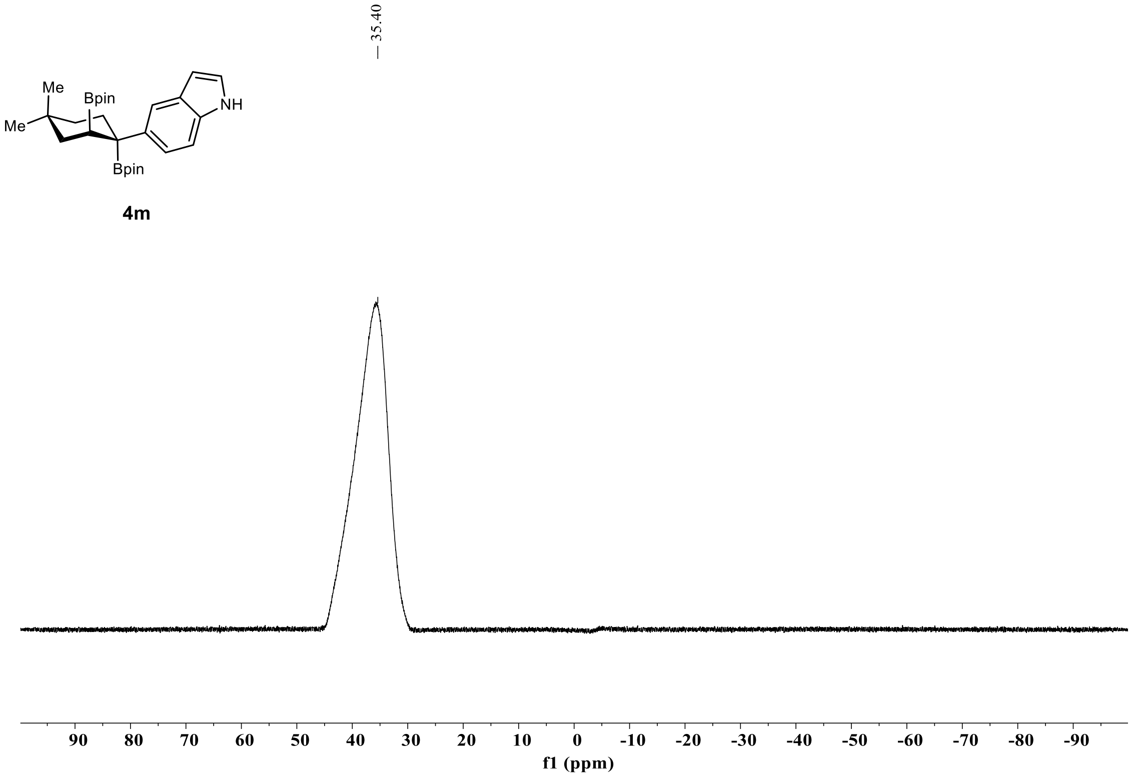

4m

^{11}B NMR (193 MHz, $CDCl_3$)

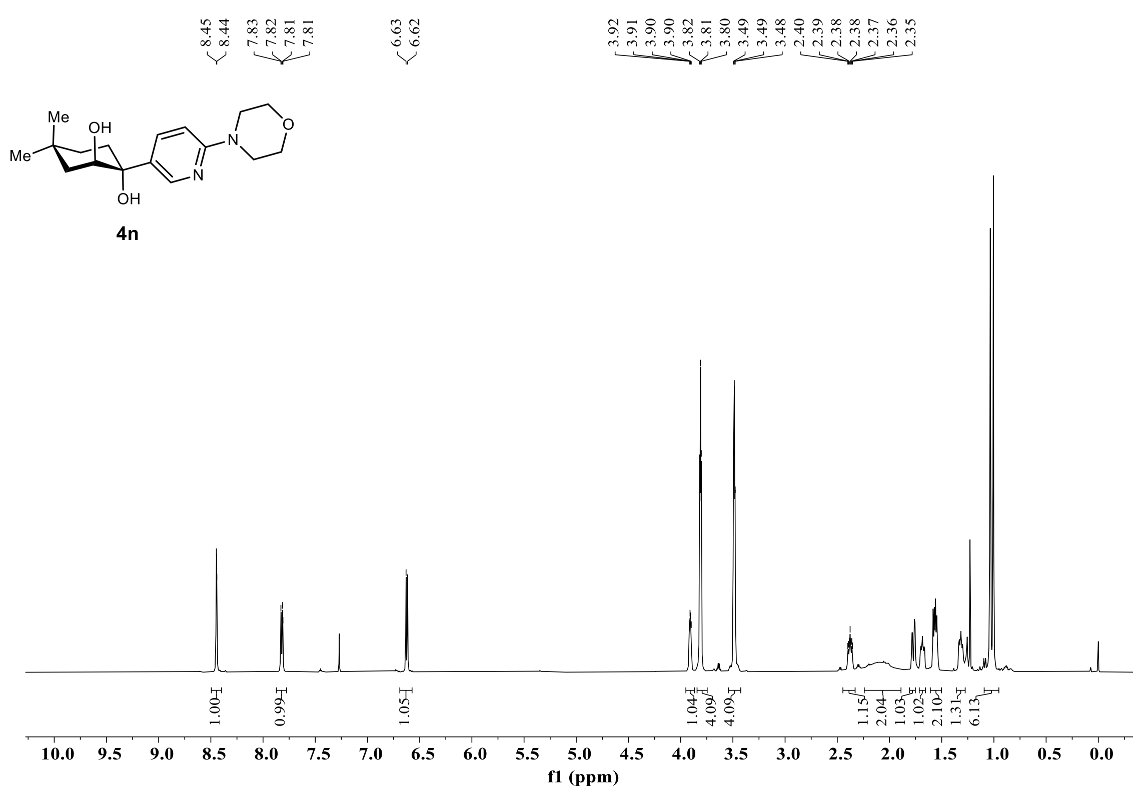

4n

^{1}H NMR (600 MHz, CDCl$_3$)

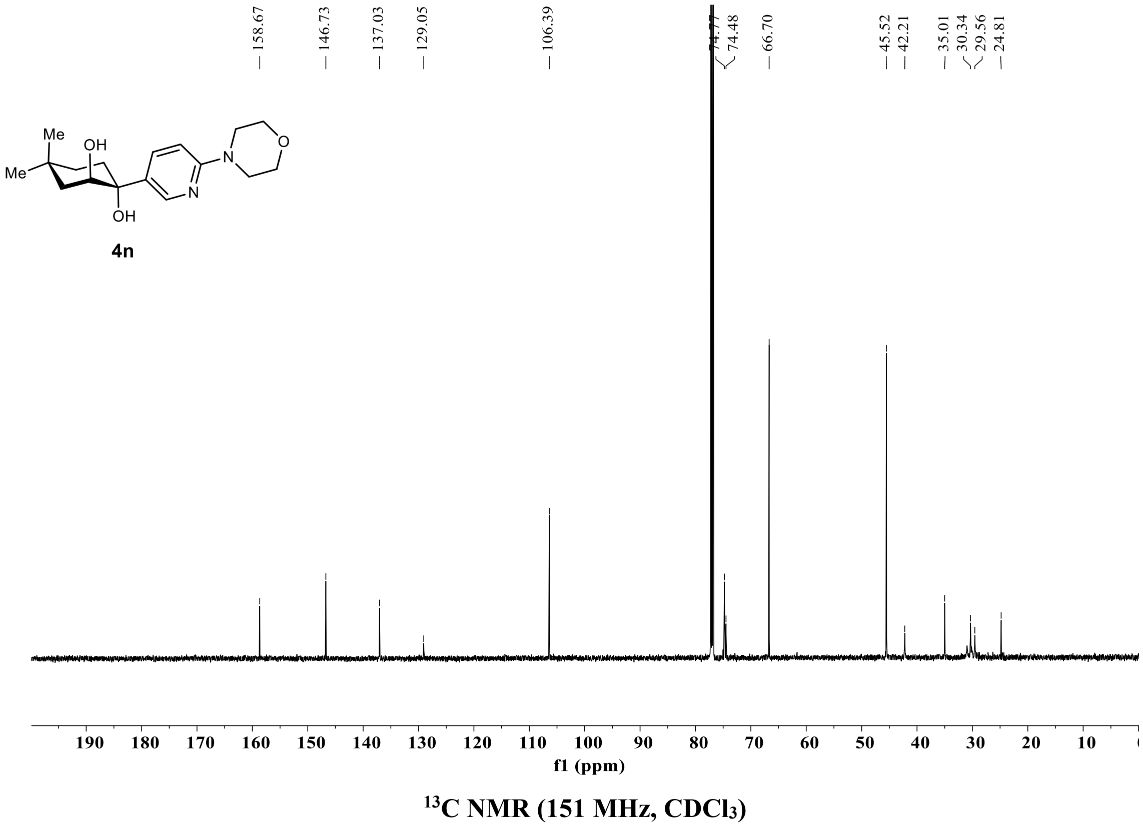

4n

^{13}C NMR (151 MHz, CDCl$_3$)

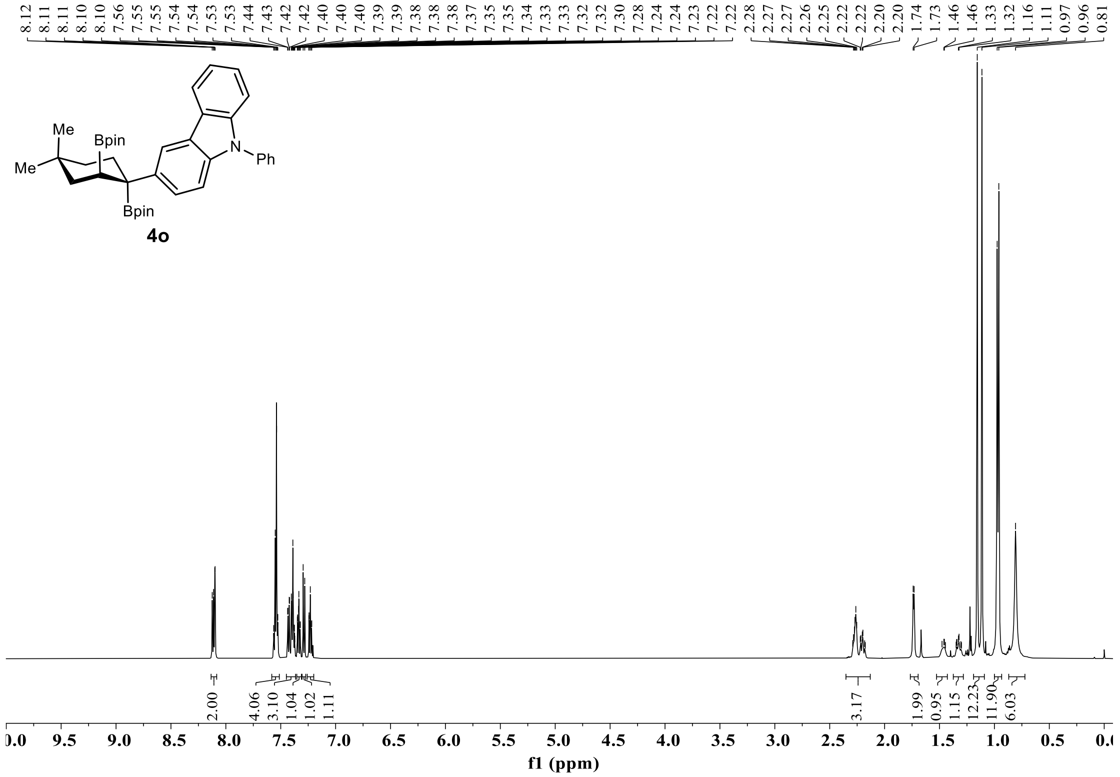

^{1}H NMR (600 MHz, CDCl$_3$)

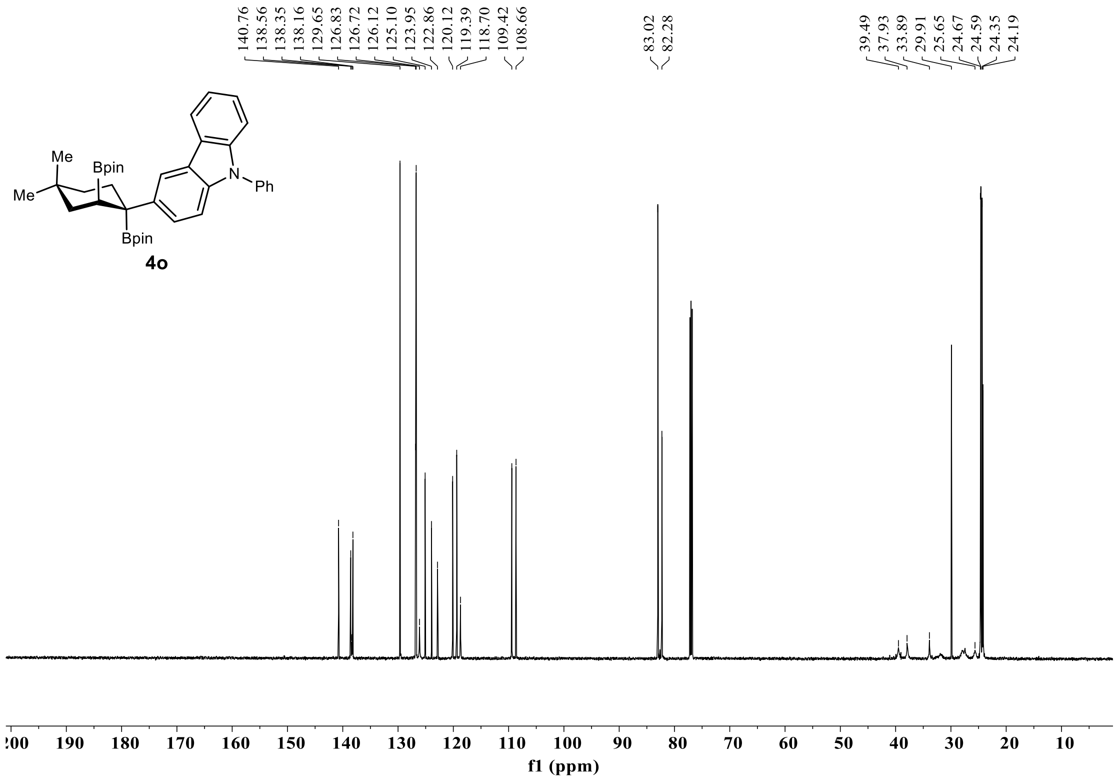

^{13}C NMR (151 MHz, CDCl$_3$)

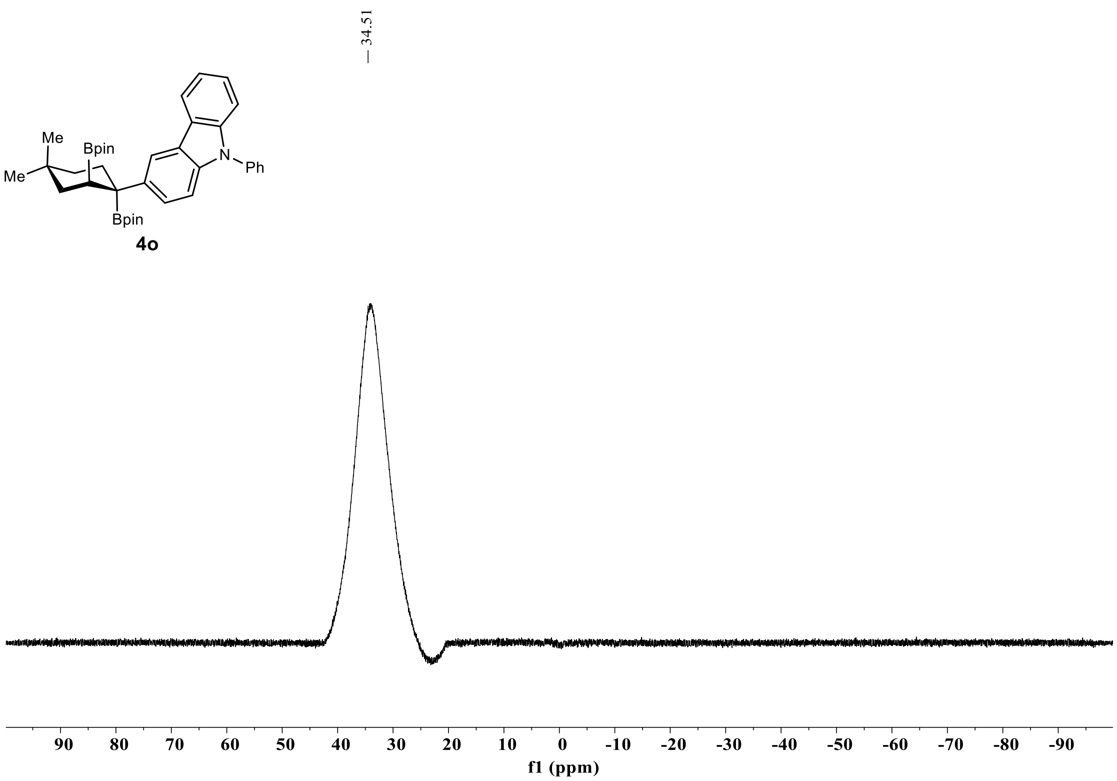

^{11}B NMR (193 MHz, $CDCl_3$)

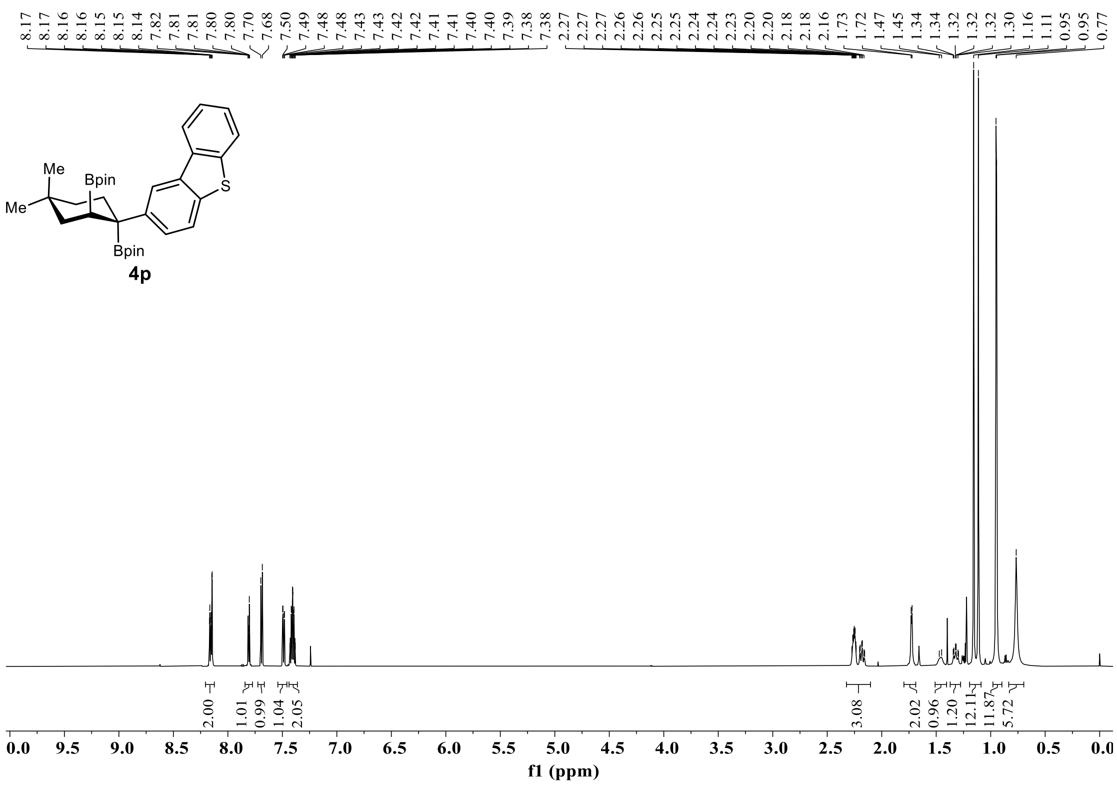

^{1}H NMR (600 MHz, $CDCl_3$)

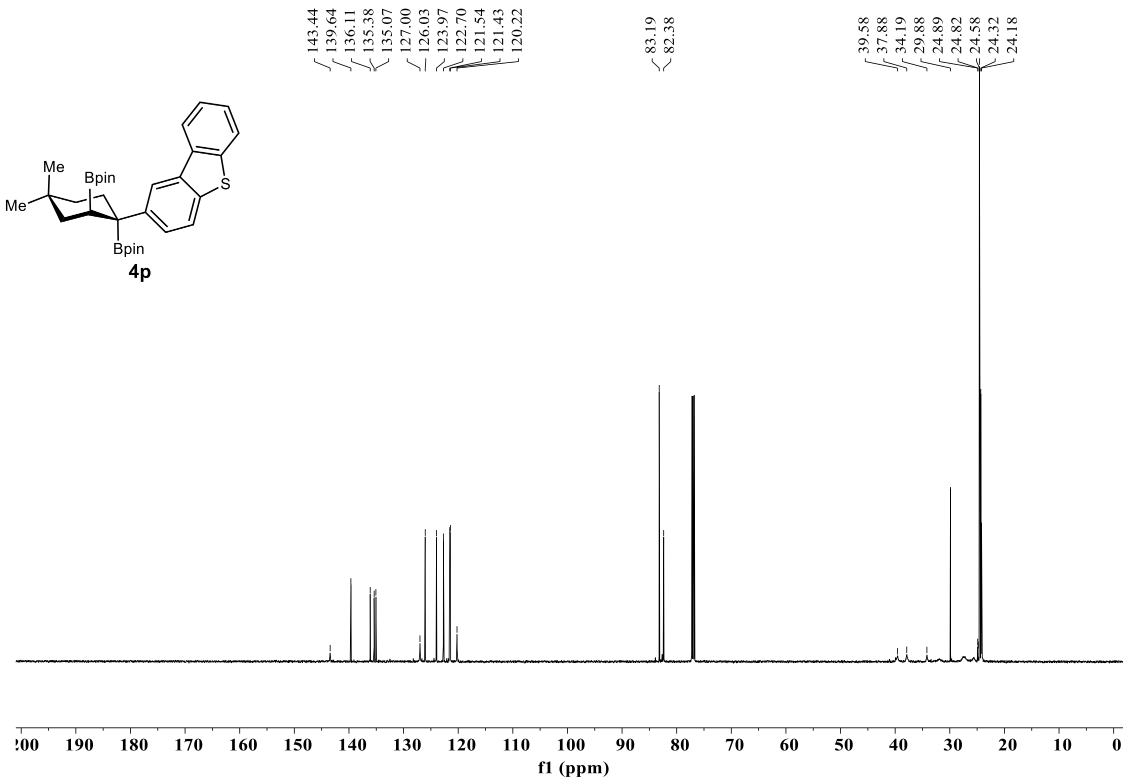

^{13}C NMR (151 MHz, CDCl$_3$)

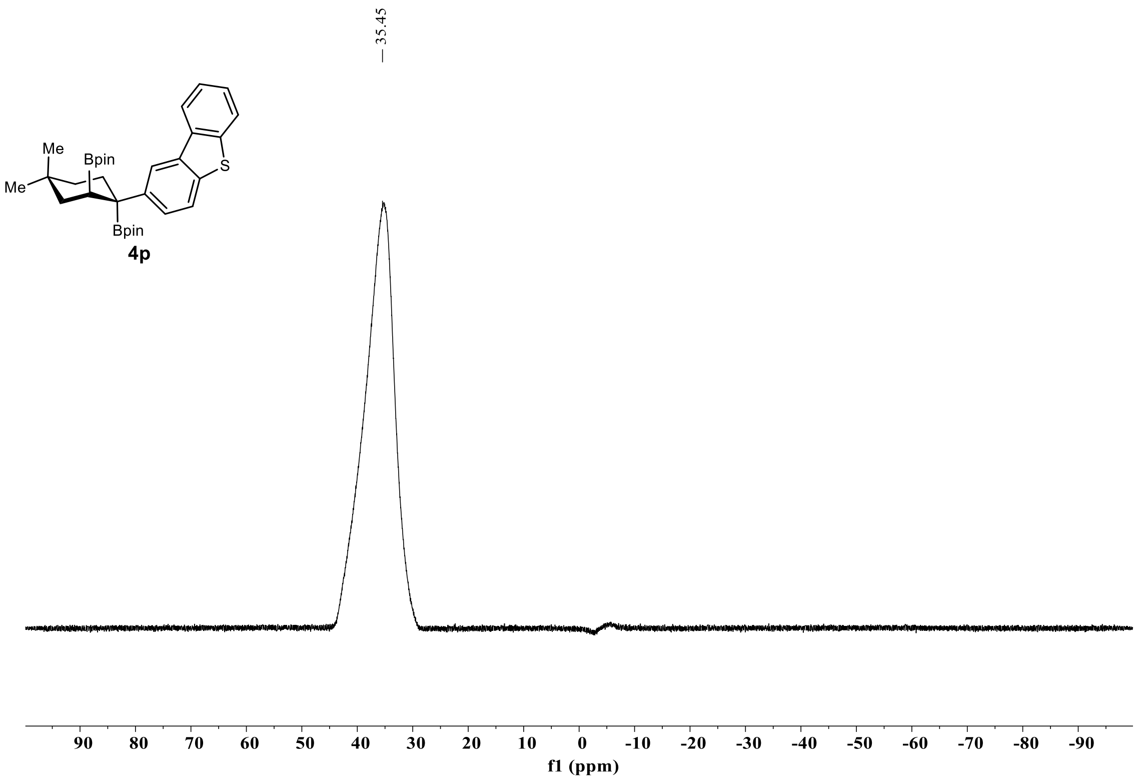

^{11}B NMR (193 MHz, CDCl$_3$)

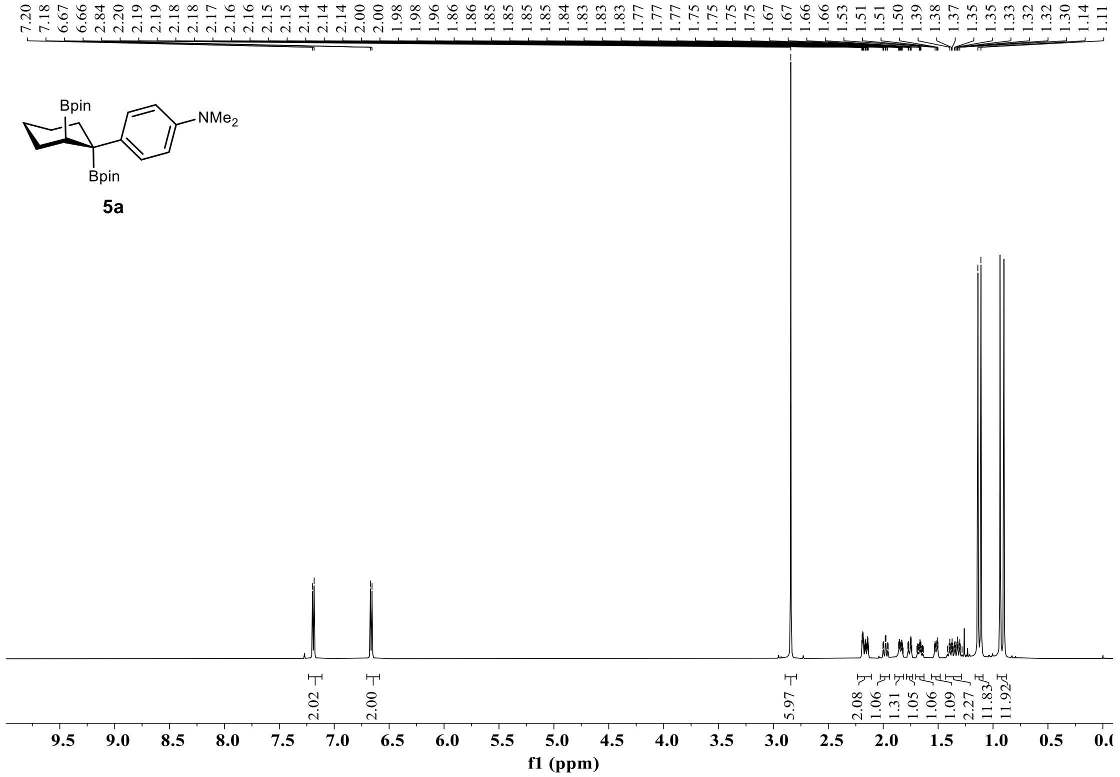

^{1}H NMR (600 MHz, CDCl3)

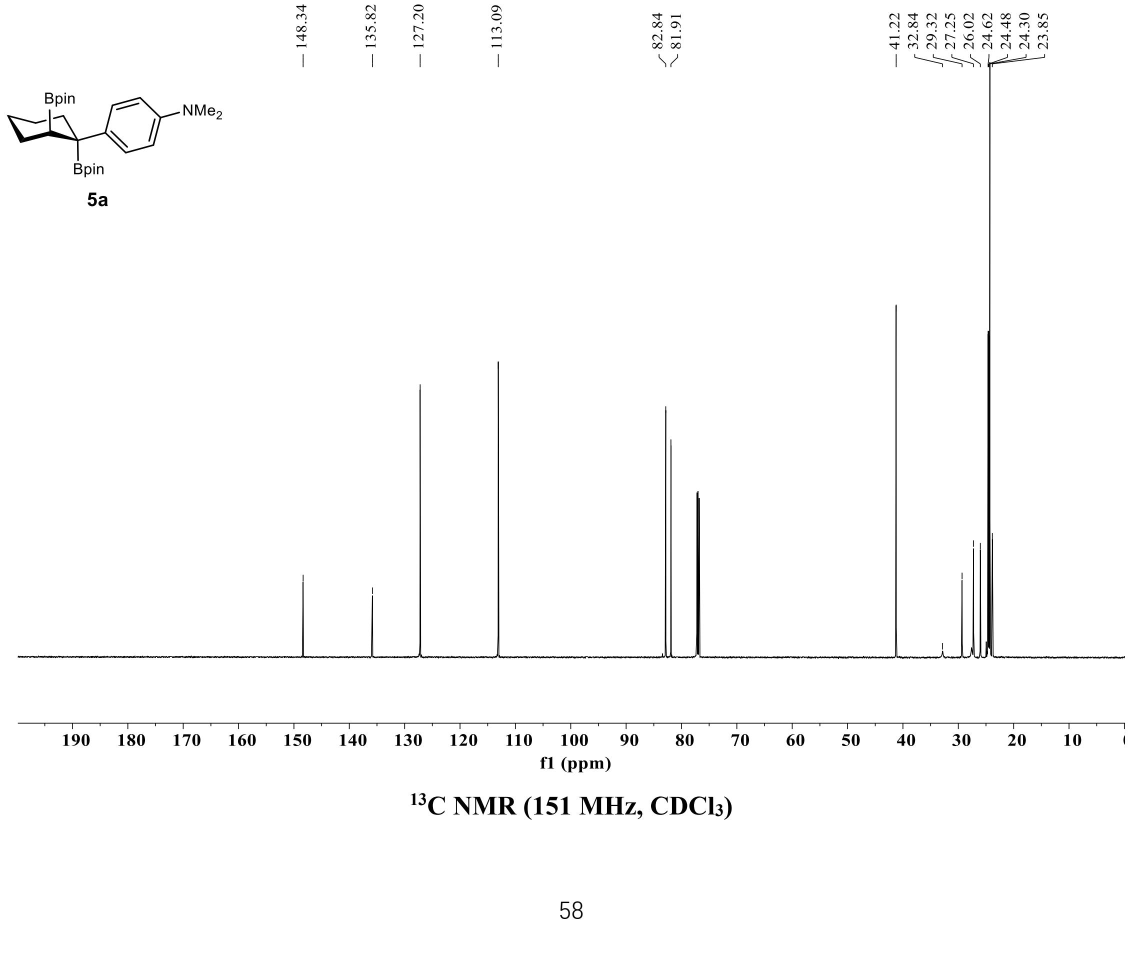

^{13}C NMR (151 MHz, CDCl3)

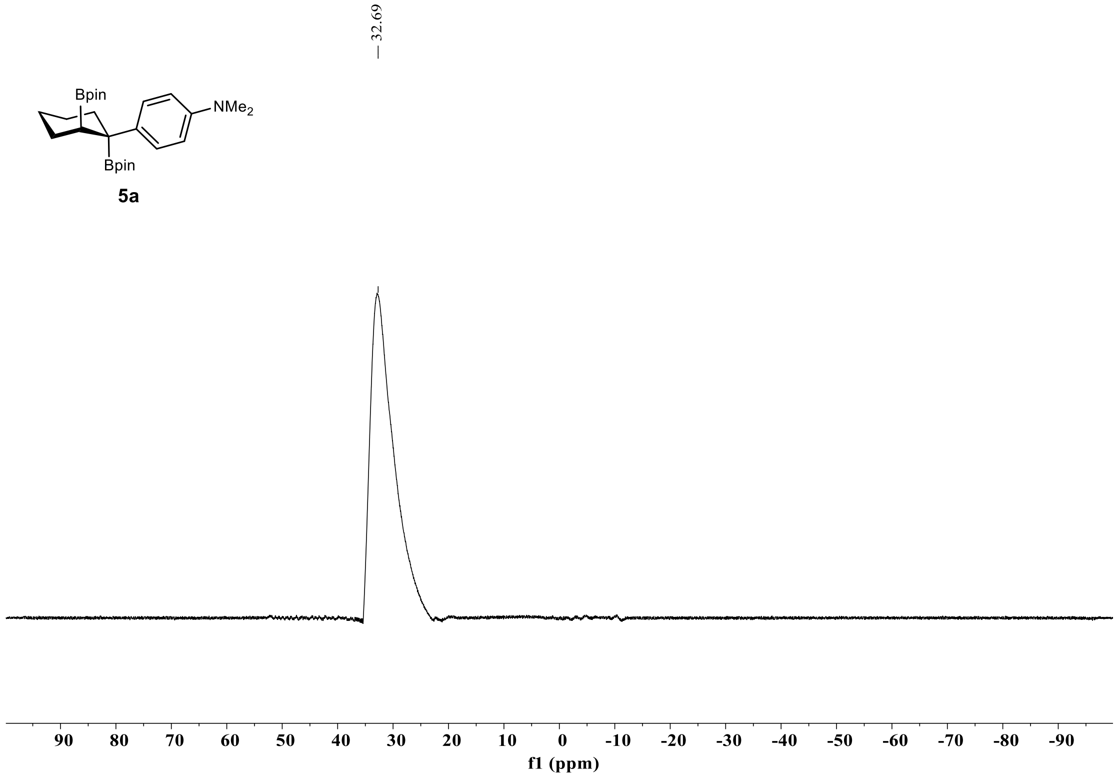

^{11}B NMR (193 MHz, CDCl$_3$)

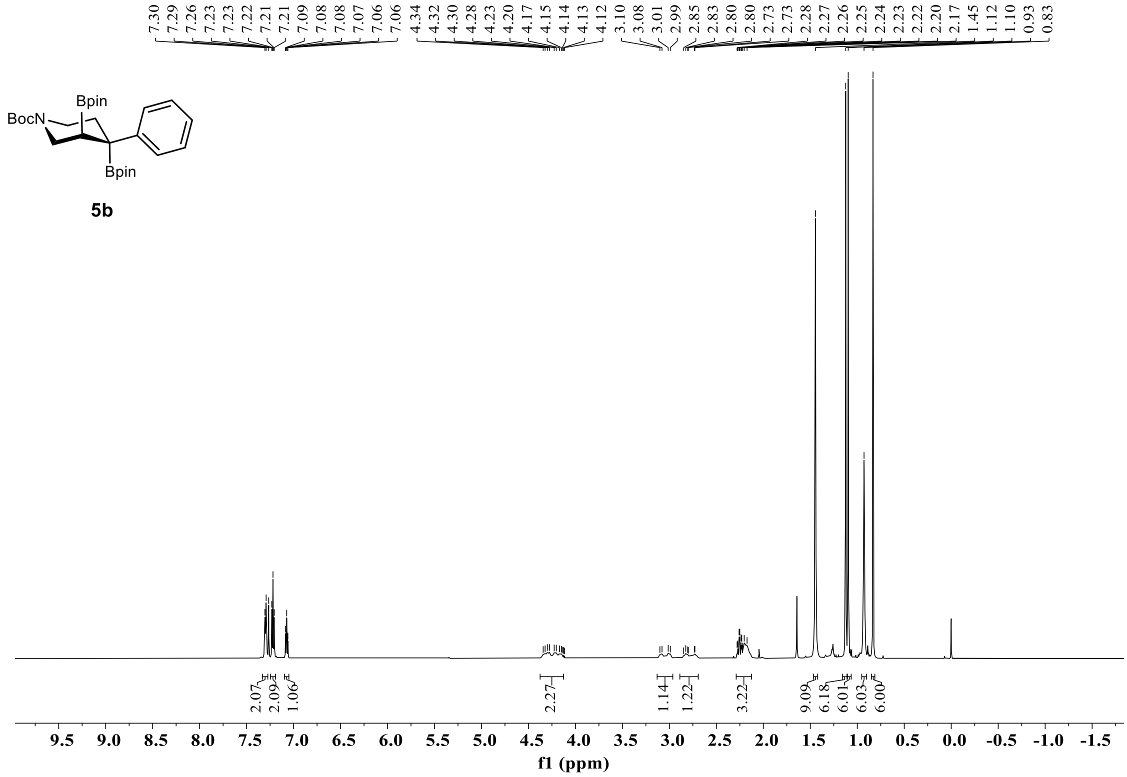

^{1}H NMR (600 MHz, CDCl$_3$)

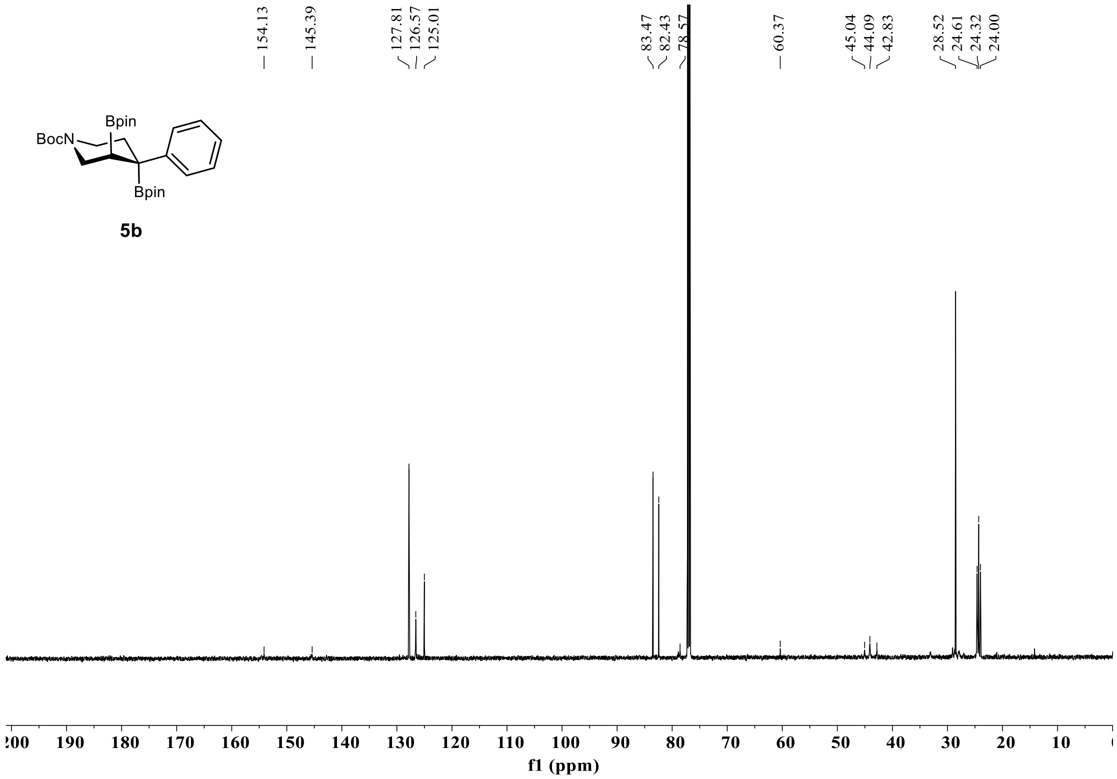

^{13}C NMR (151 MHz, CDCl$_3$)

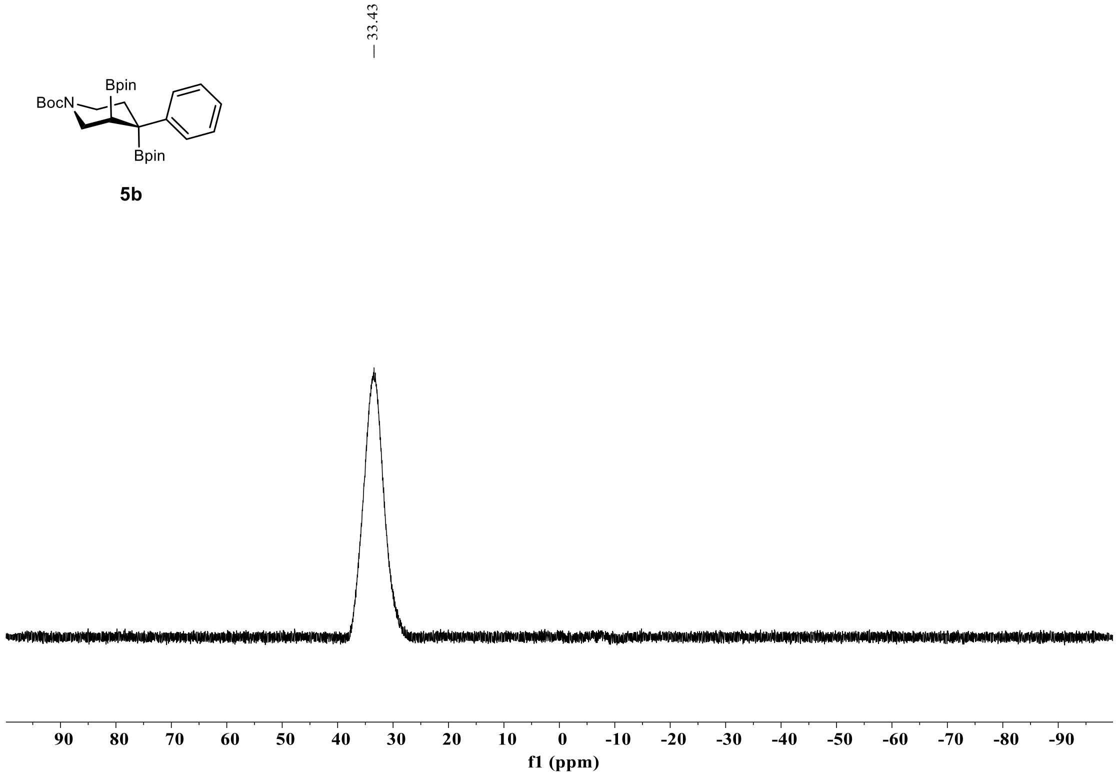

^{11}B NMR (193 MHz, CDCl$_3$)

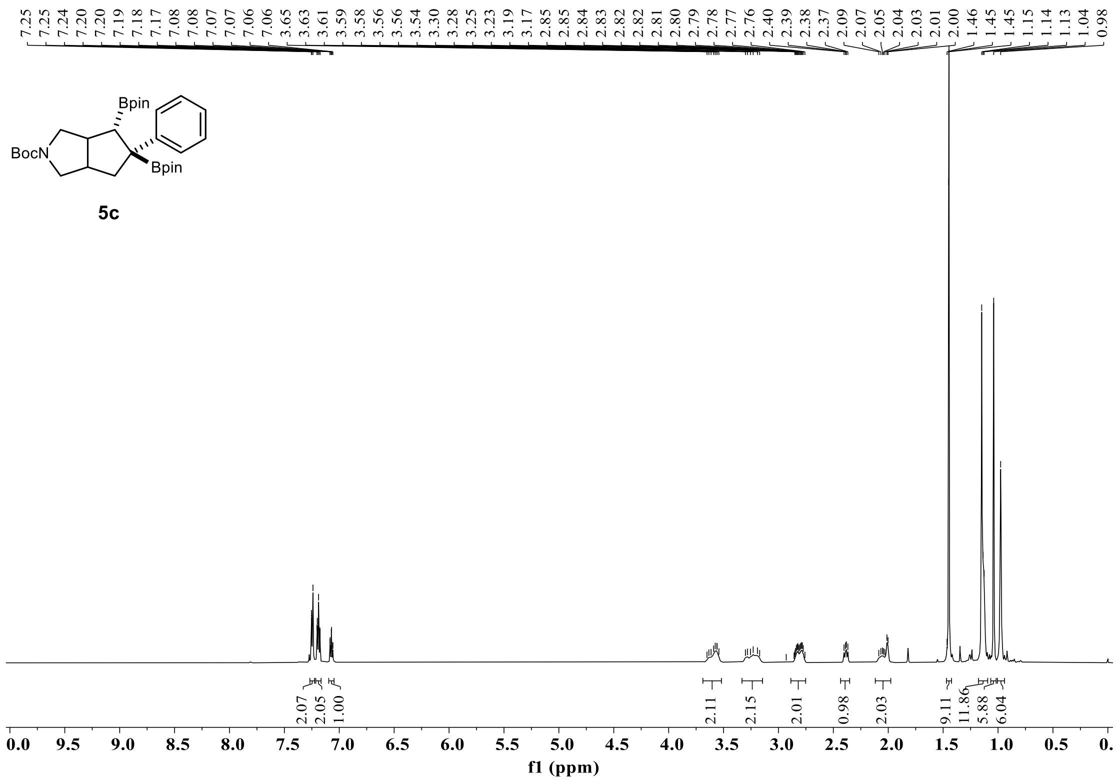

^{1}H NMR (600 MHz, $CDCl_3$)

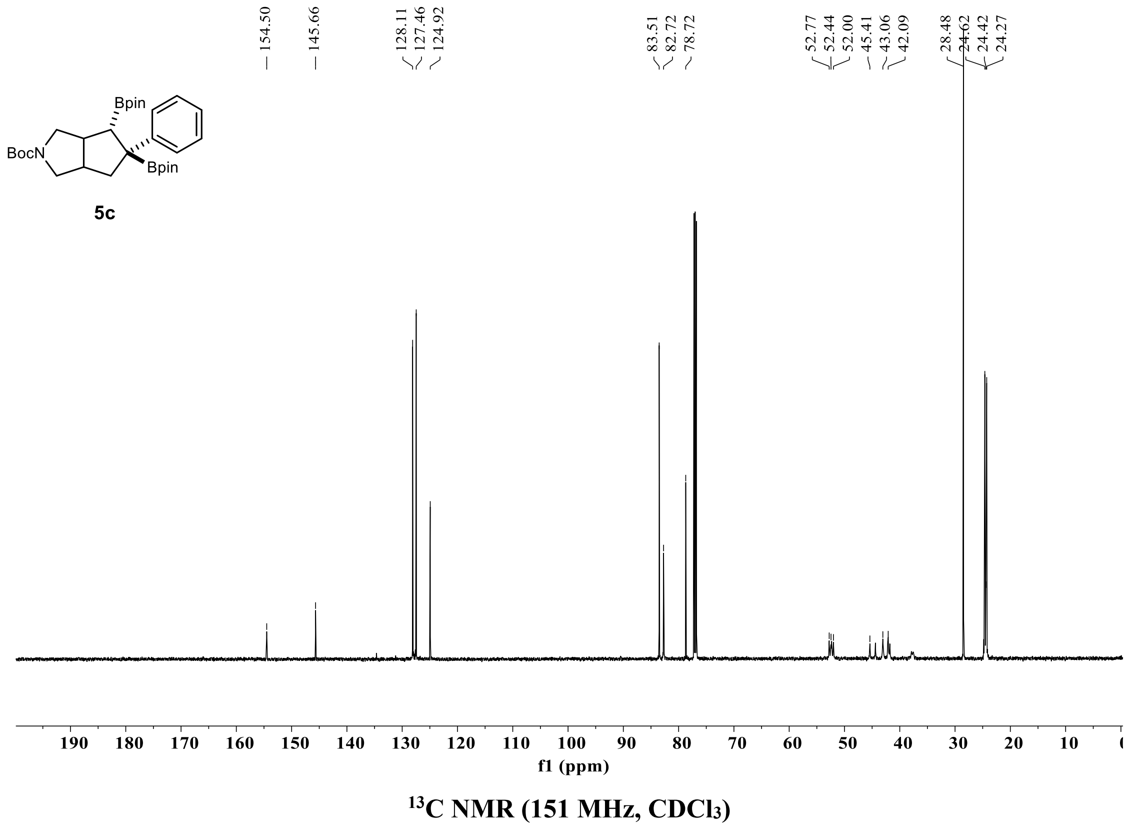

^{13}C NMR (151 MHz, $CDCl_3$)

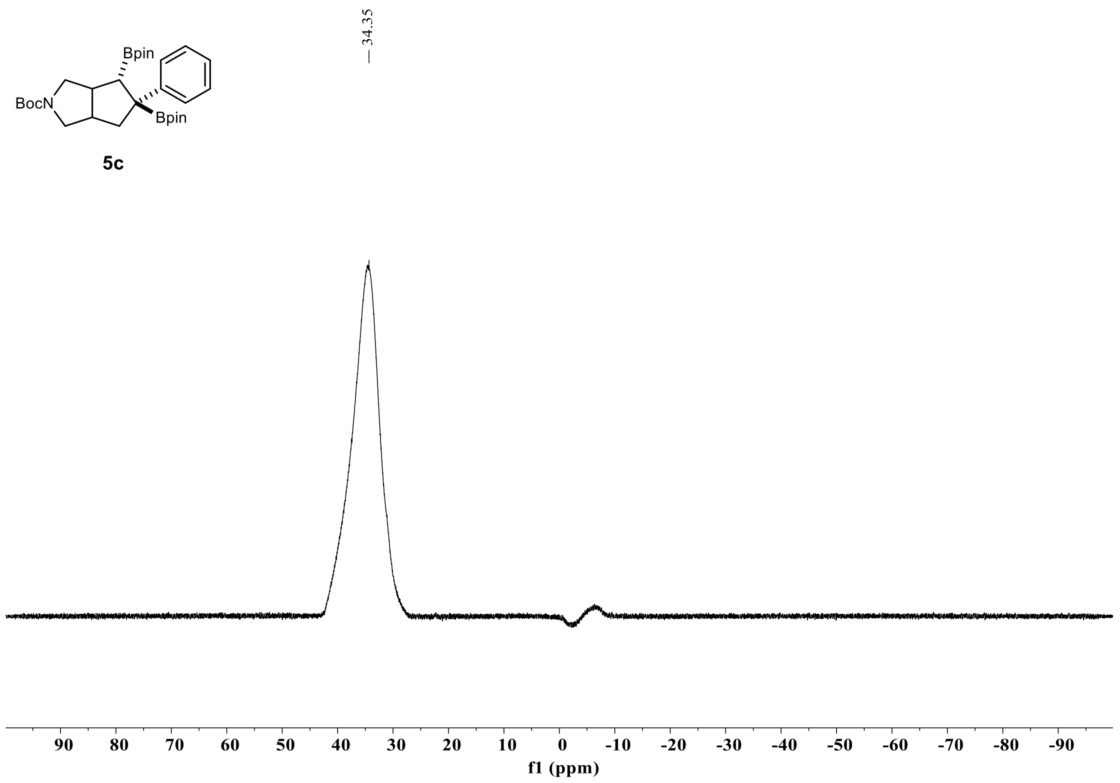

^{11}B NMR (193 MHz, CDCl$_3$)

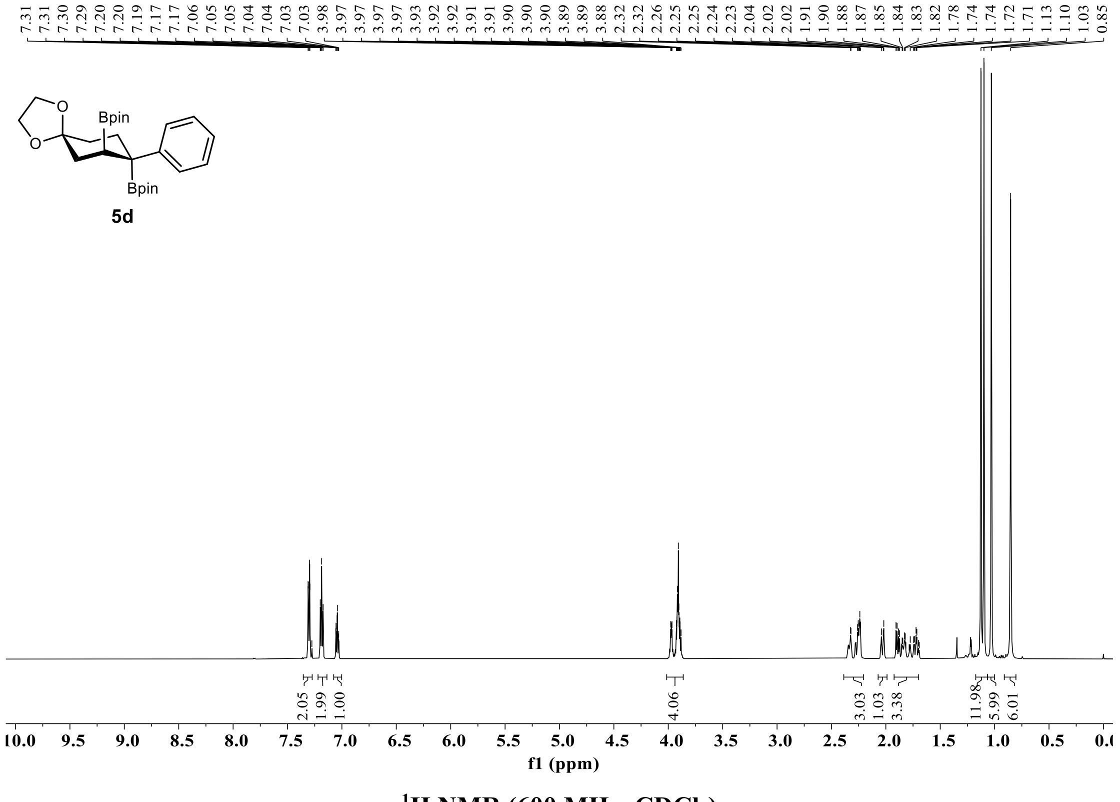

^{1}H NMR (600 MHz, CDCl$_3$)

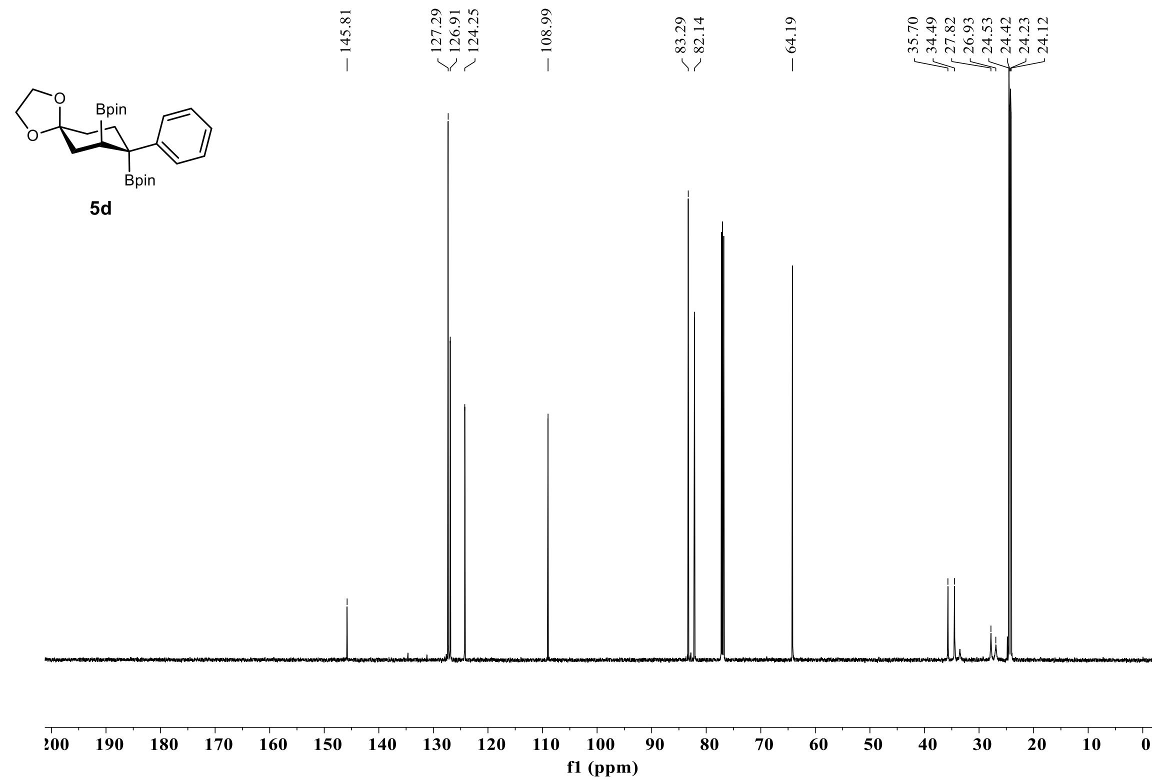

^{13}C NMR (151 MHz, CDCl$_3$)

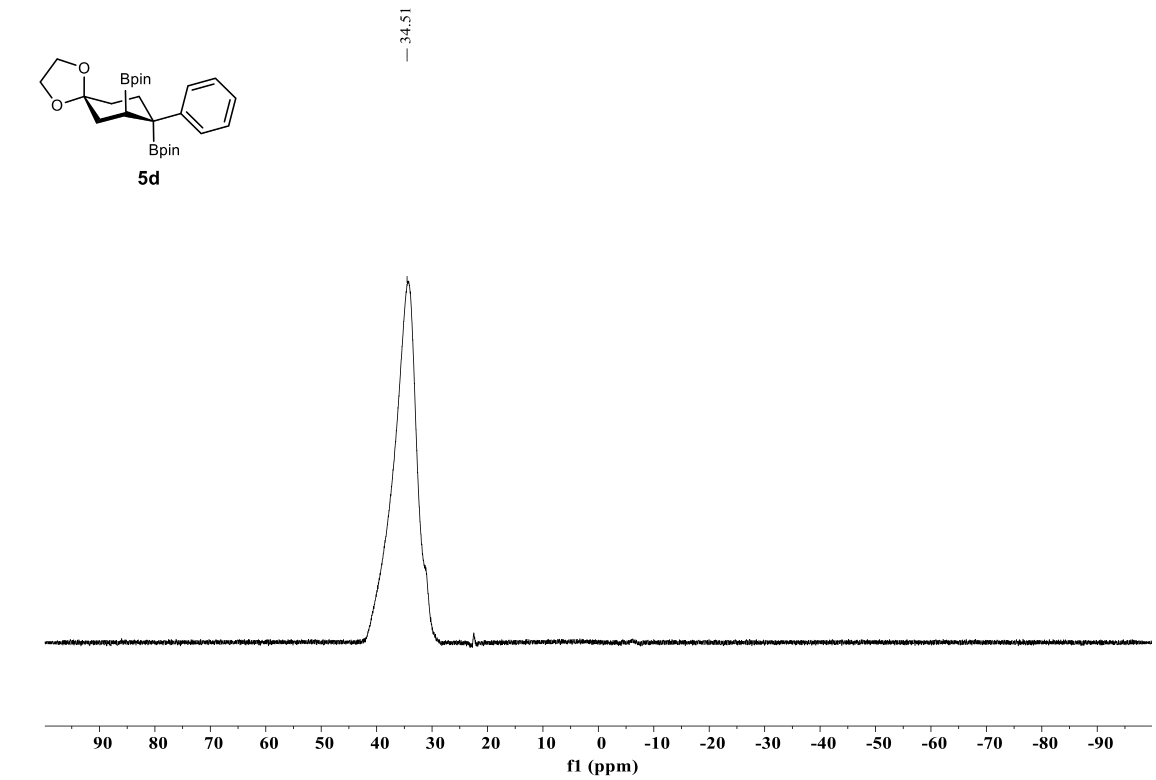

^{11}B NMR (193 MHz, CDCl$_3$)

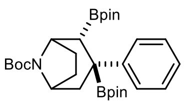

5e

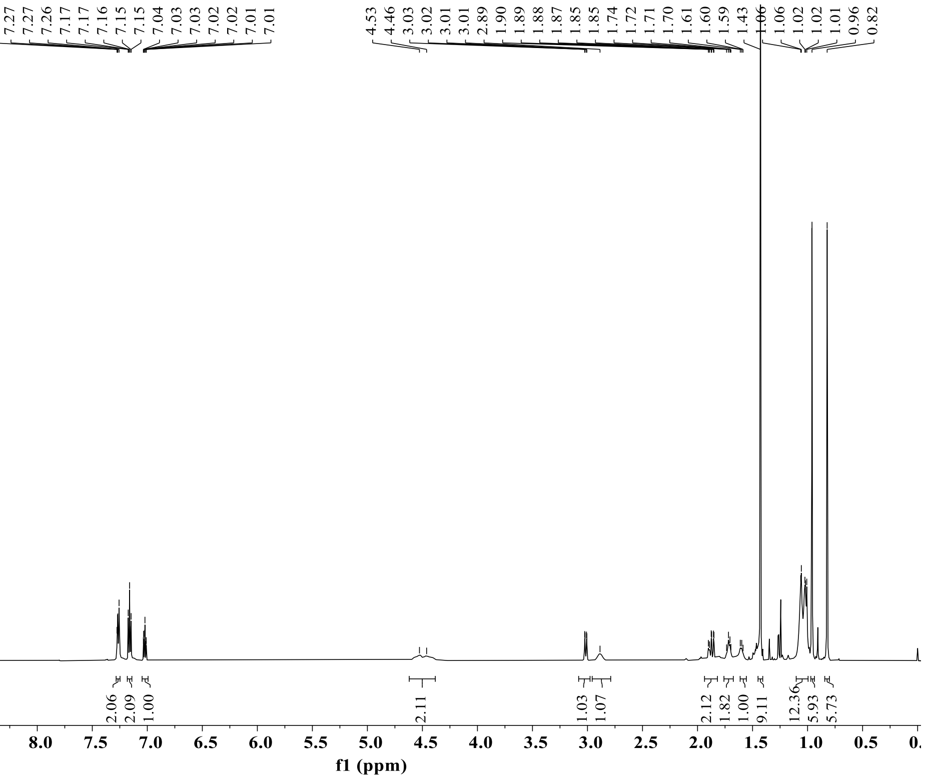

^{1}H NMR (600 MHz, CDCl3)

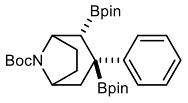

5e

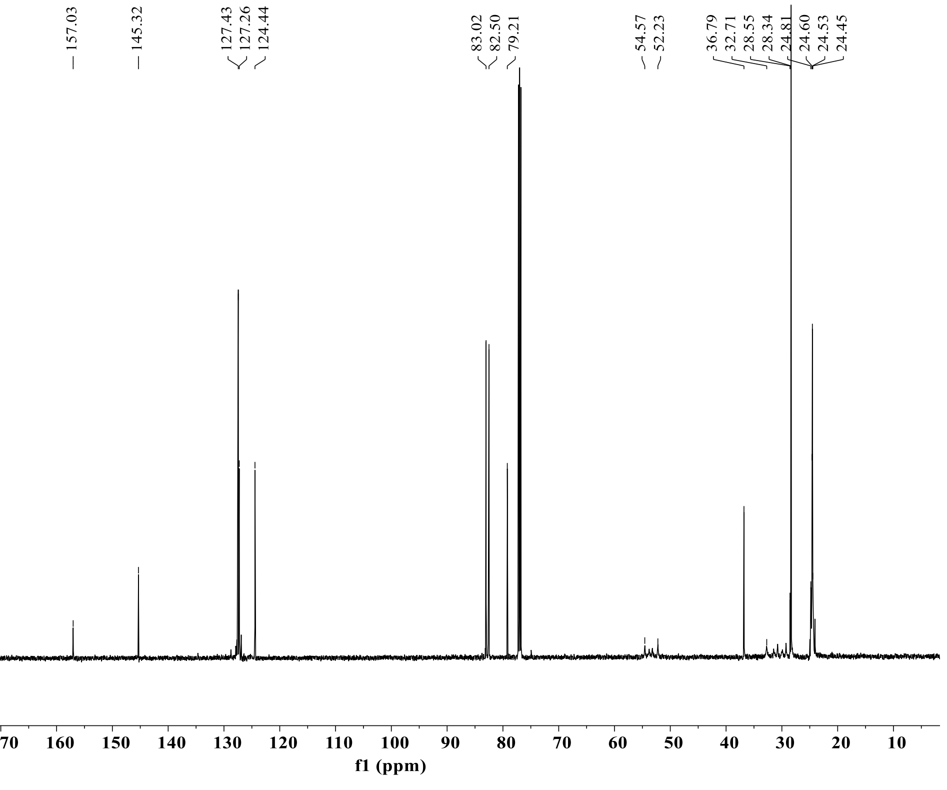

^{13}C NMR (151 MHz, CDCl3)

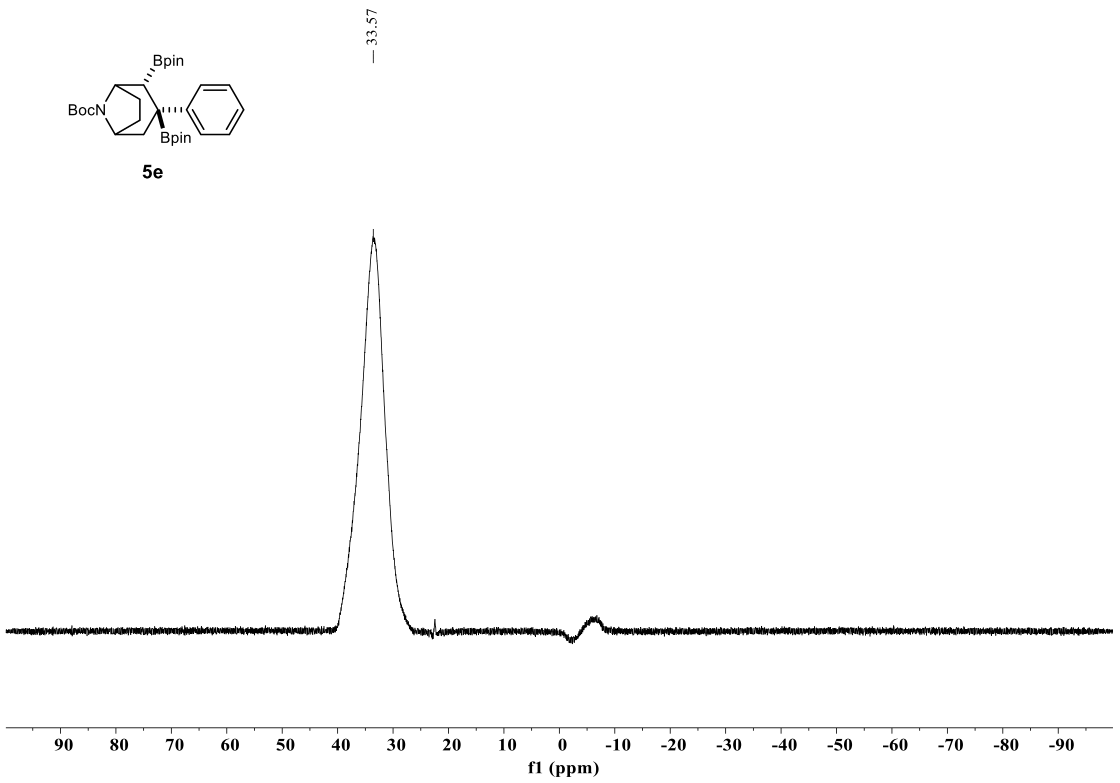

^{11}B NMR (193 MHz, $CDCl_3$)

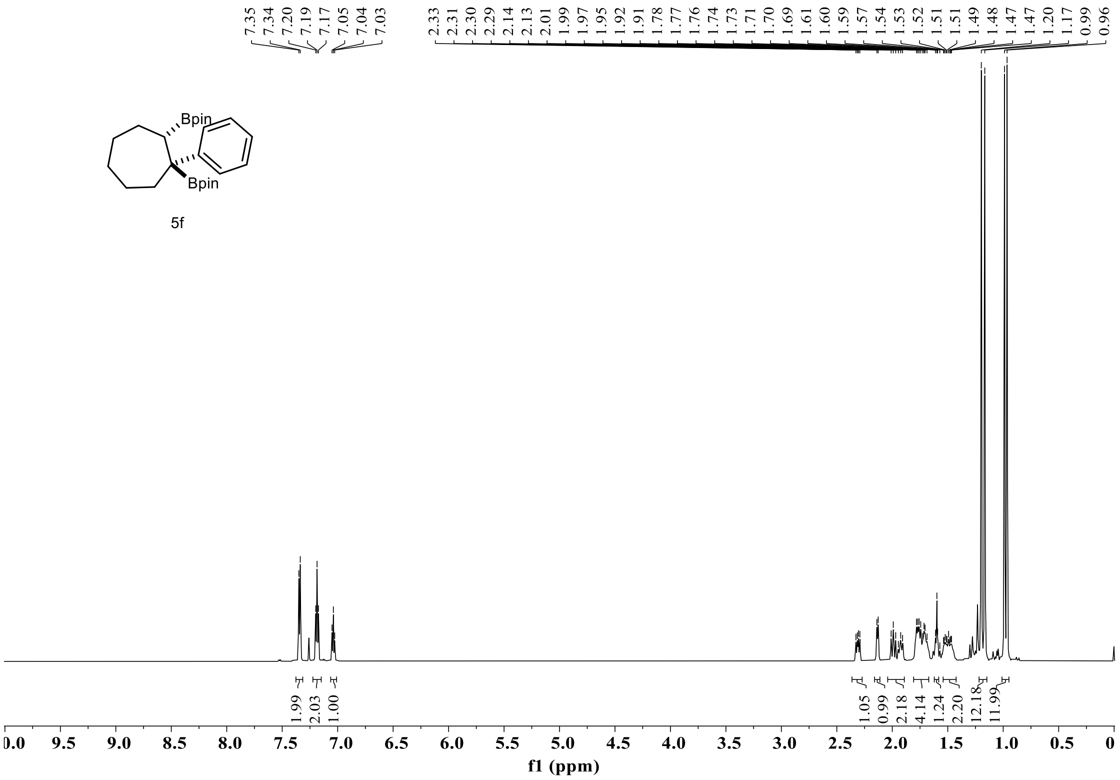

^{1}H NMR (600 MHz, $CDCl_3$)

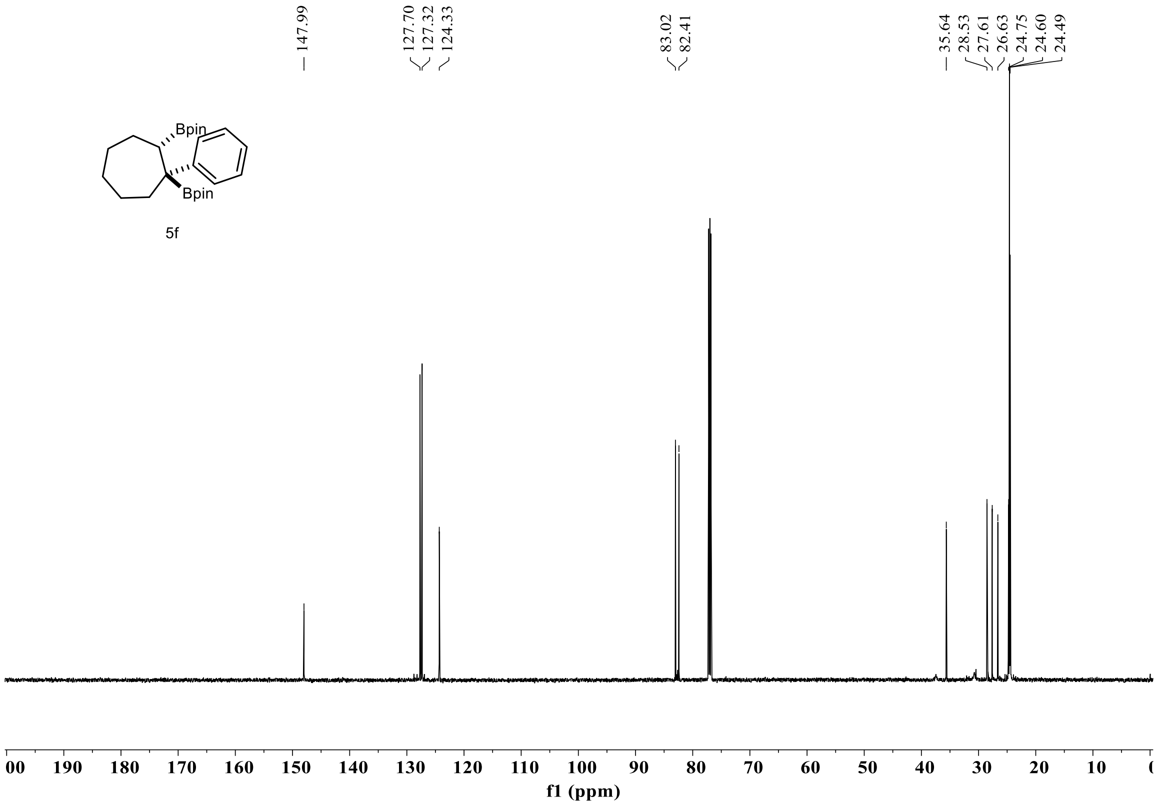

^{13}C NMR (151 MHz, $CDCl_3$)

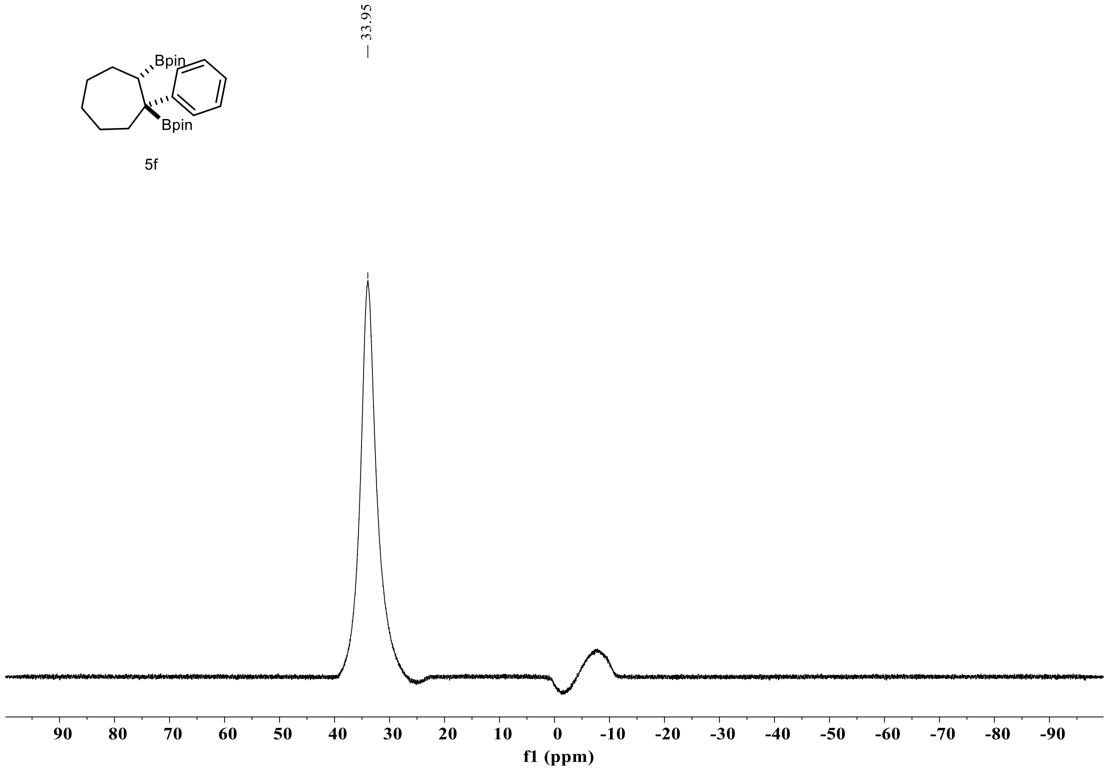

^{11}B NMR (193 MHz, $CDCl_3$)

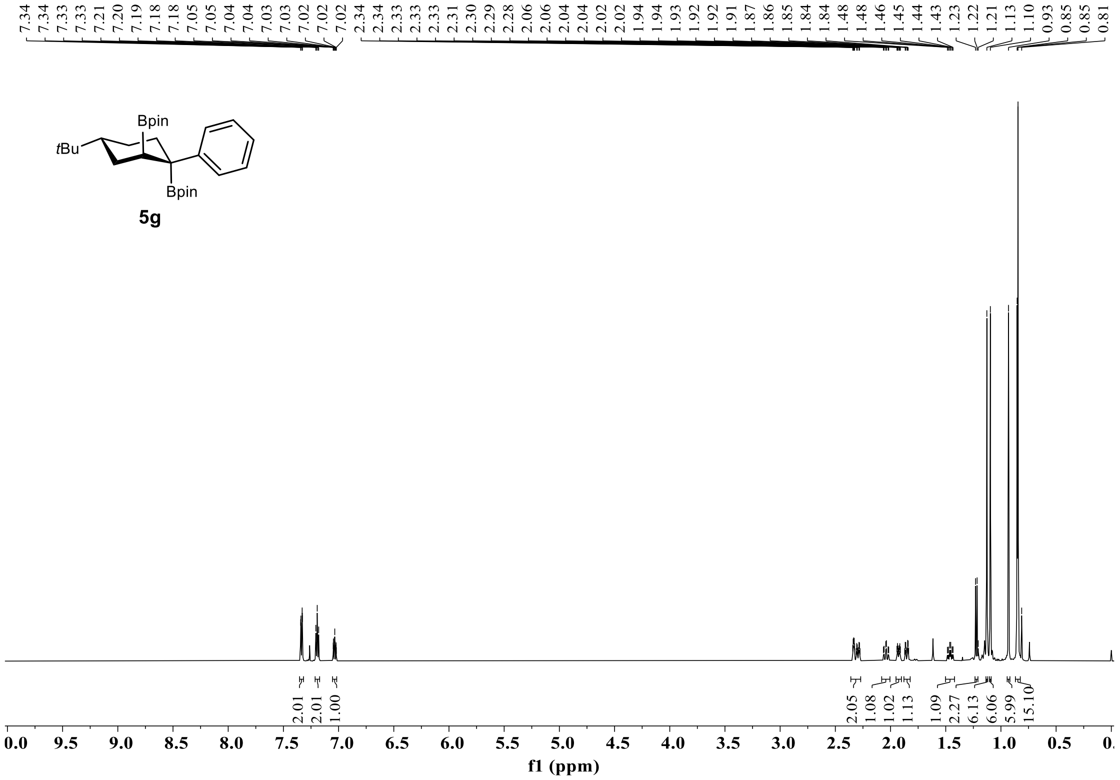

^{1}H NMR (600 MHz, $CDCl_3$)

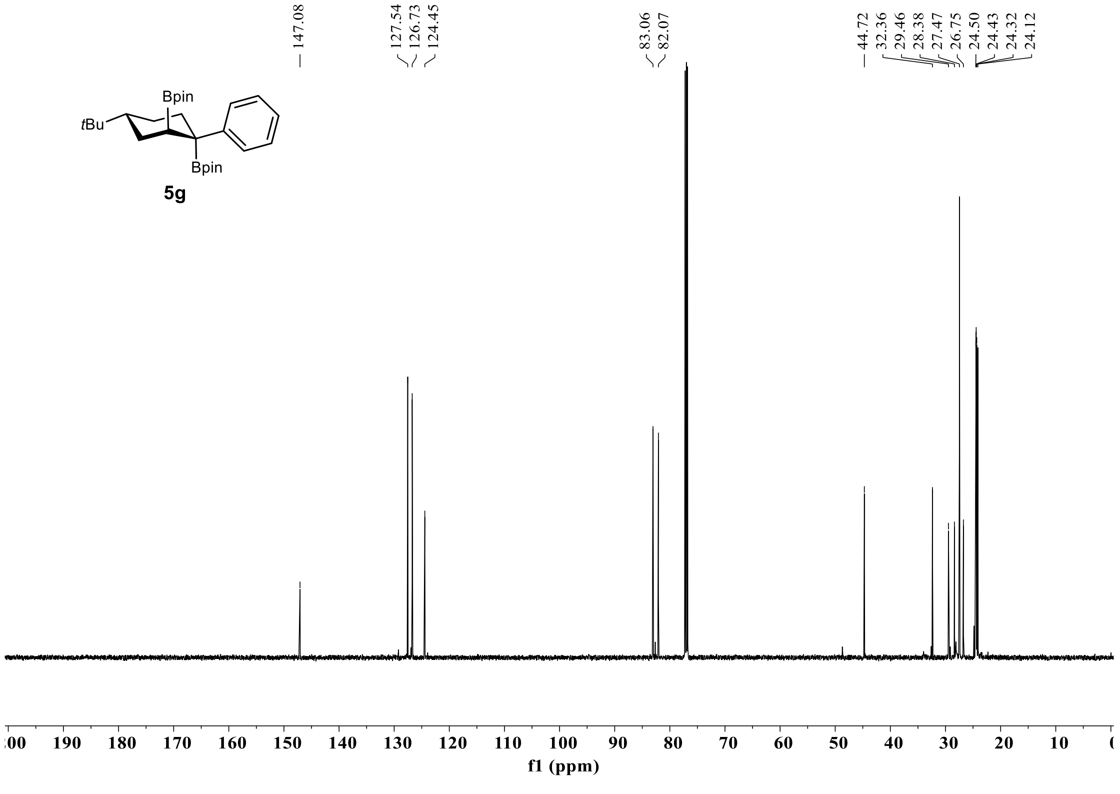

^{13}C NMR (151 MHz, $CDCl_3$)

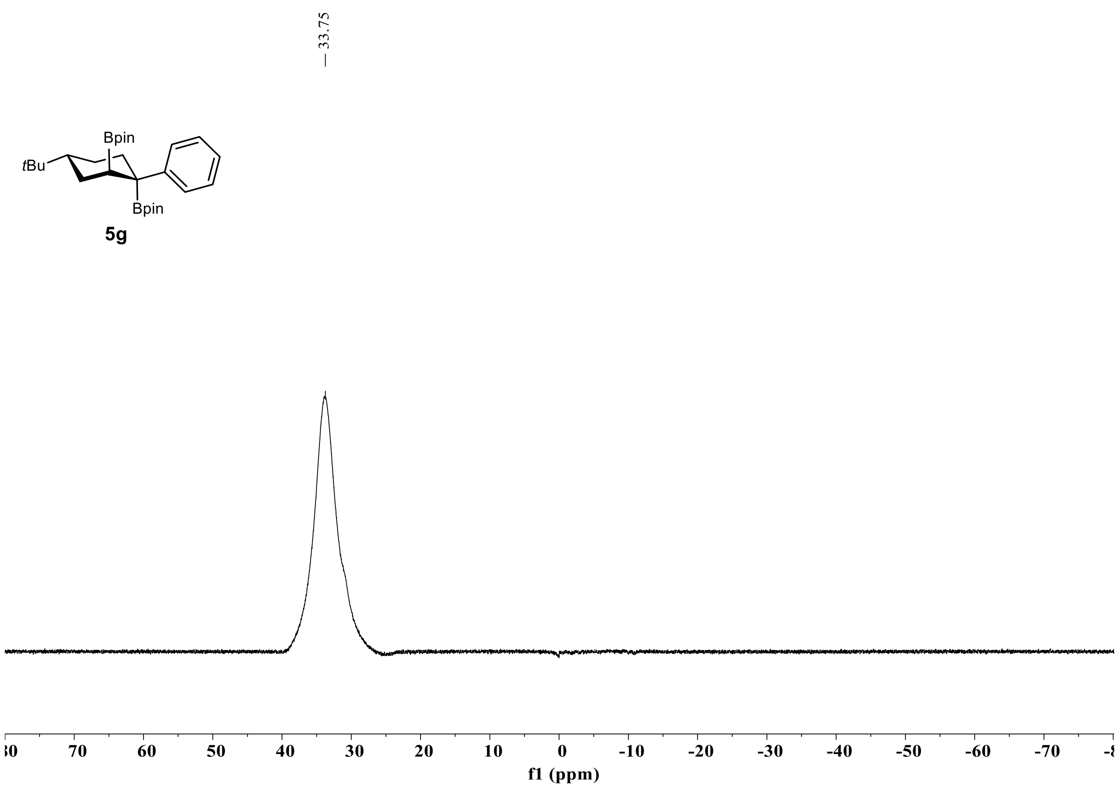

^{11}B NMR (193 MHz, CDCl$_3$)

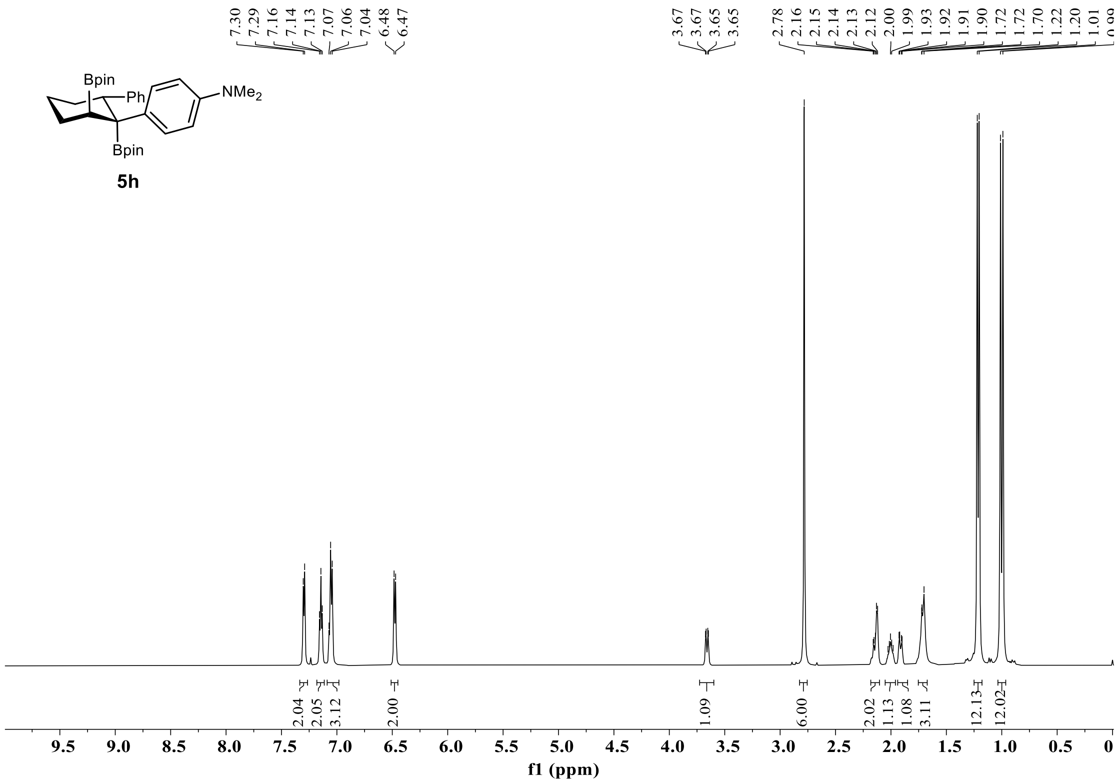

^{1}H NMR (600 MHz, CDCl$_3$)

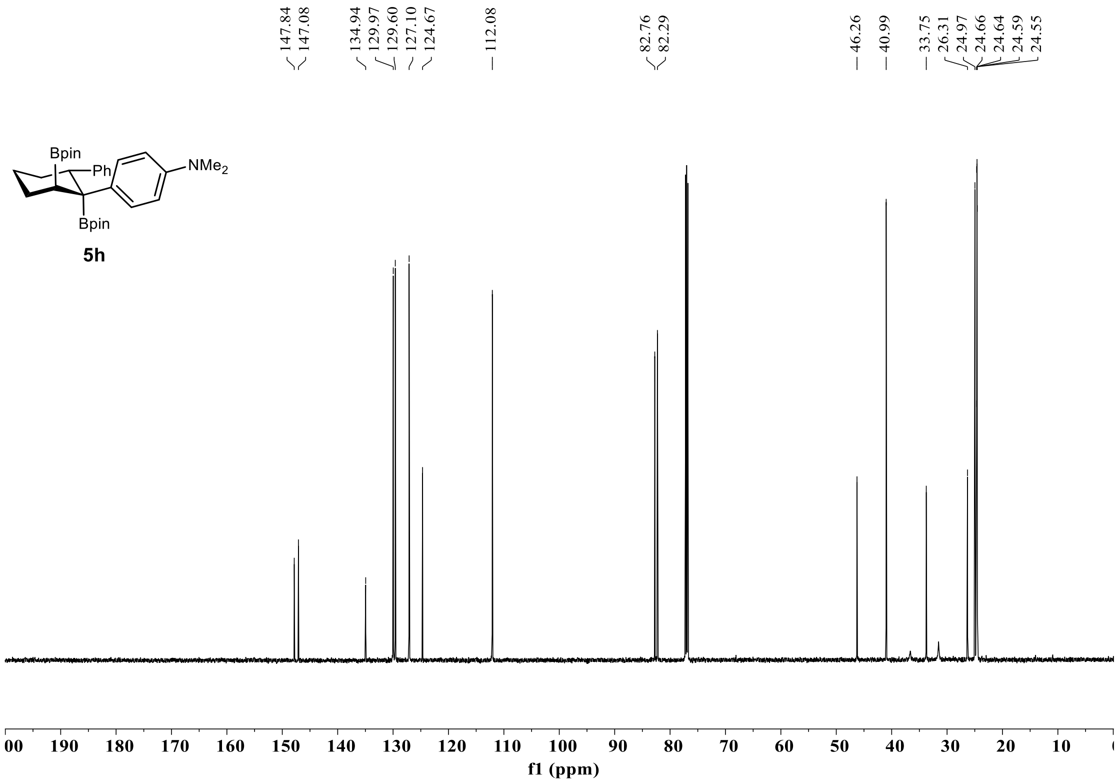

^{13}C NMR (151 MHz, CDCl$_3$)

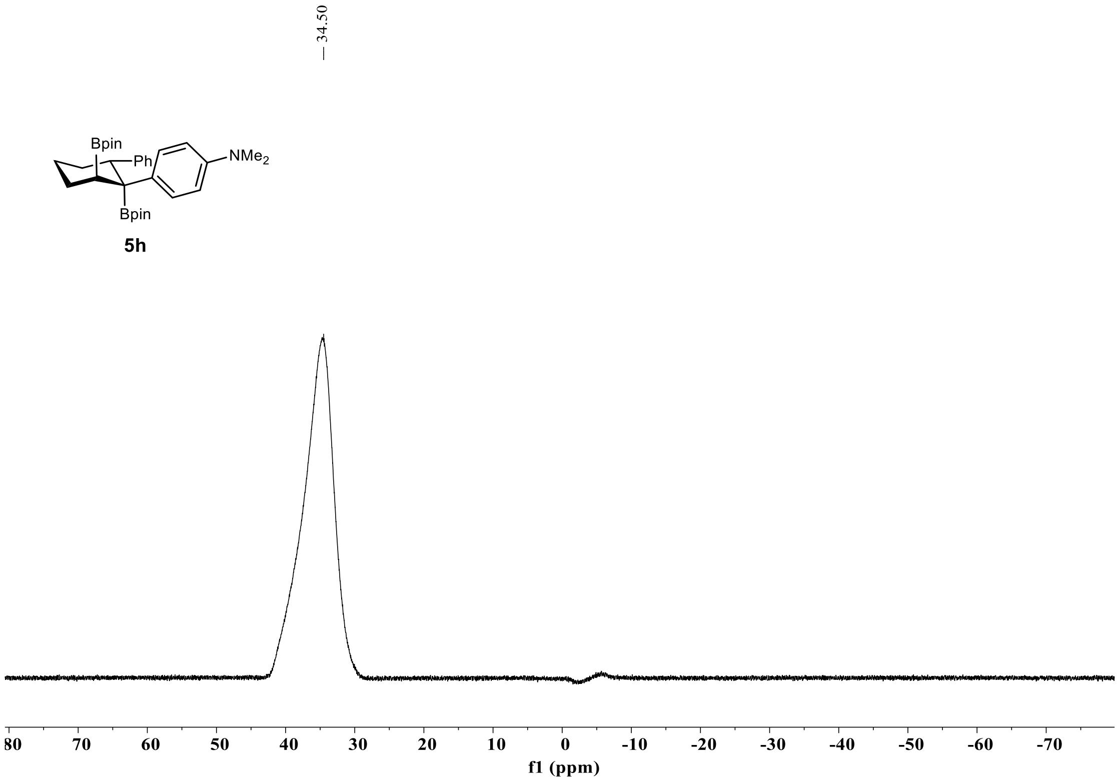

^{11}B NMR (193 MHz, CDCl$_3$)

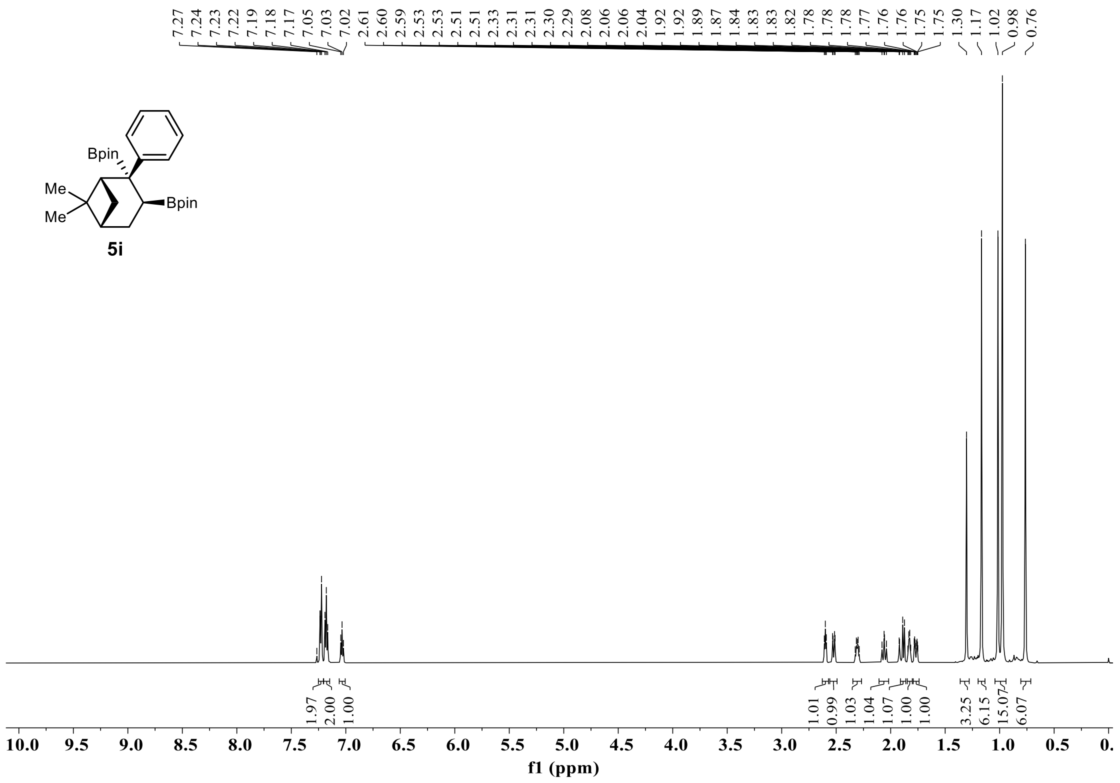

^{1}H NMR (600 MHz, CDCl$_3$)

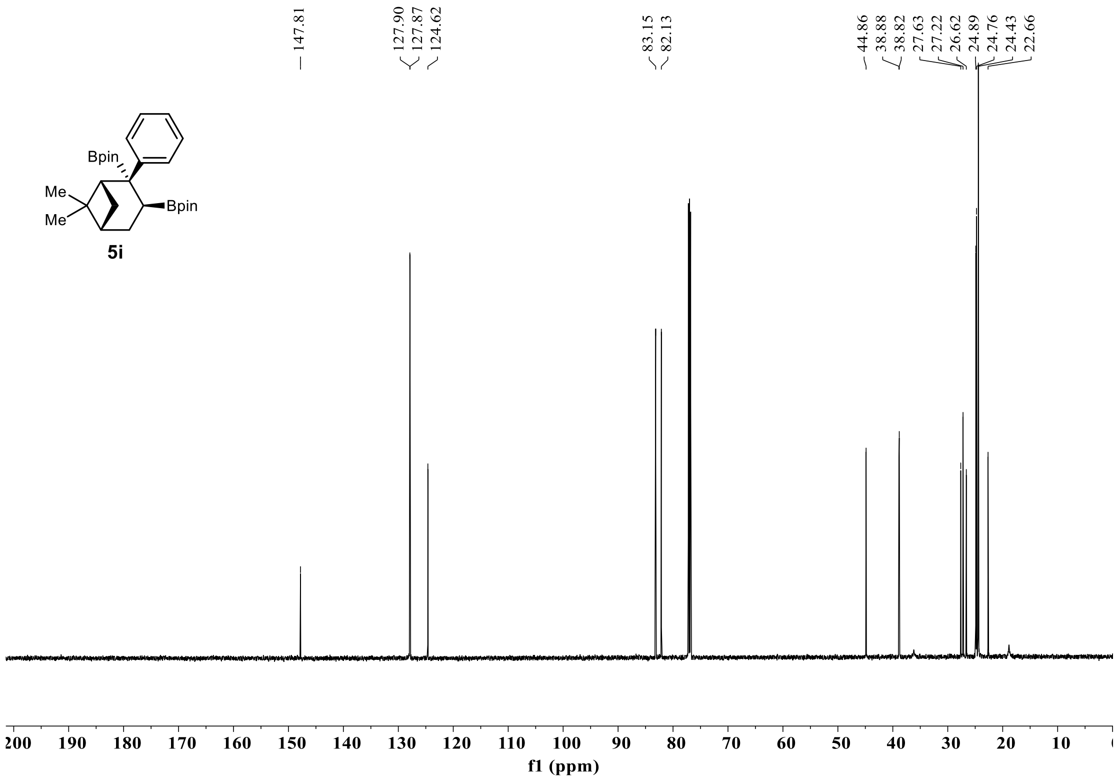

^{13}C NMR (151 MHz, CDCl$_3$)

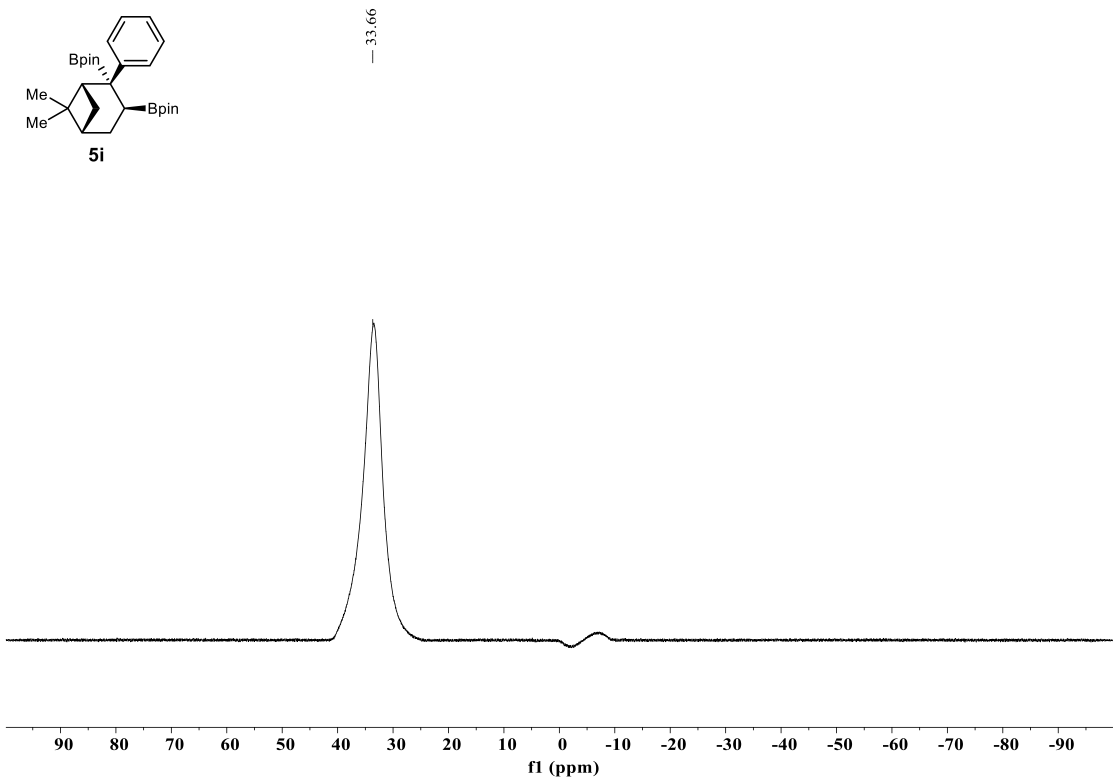

^{11}B NMR (193 MHz, CDCl$_3$)

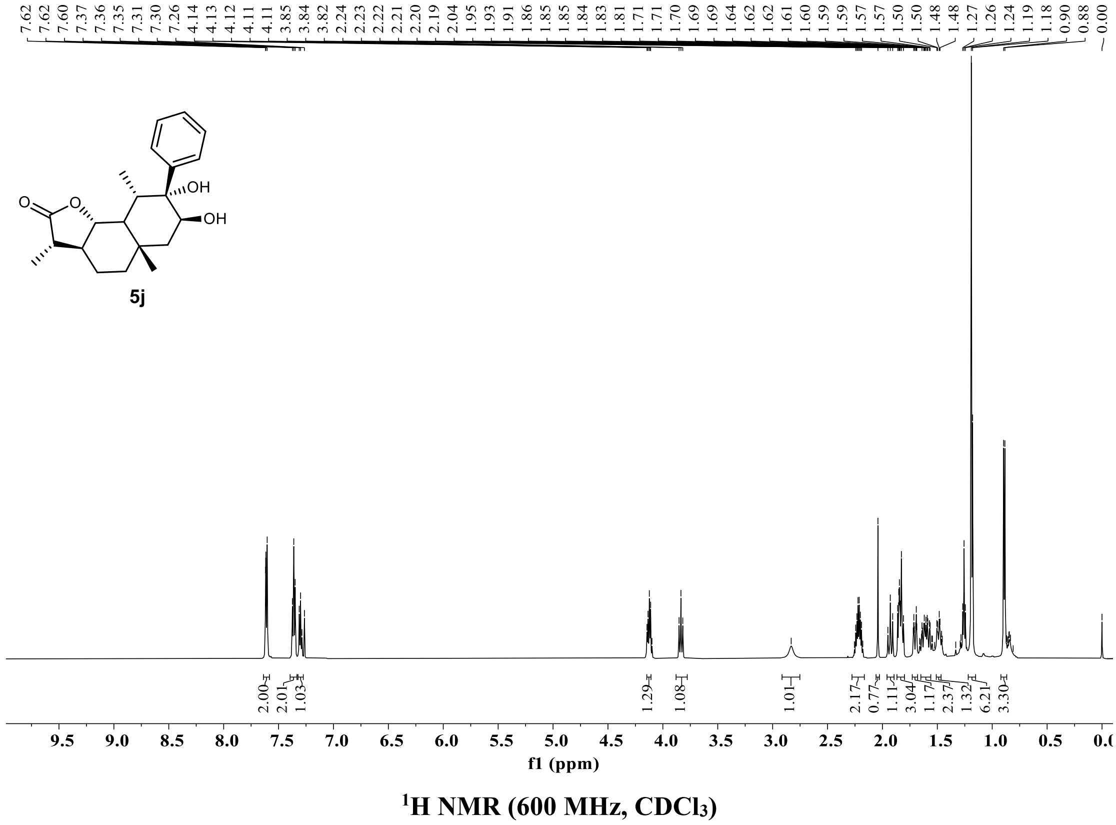

^{1}H NMR (600 MHz, CDCl$_3$)

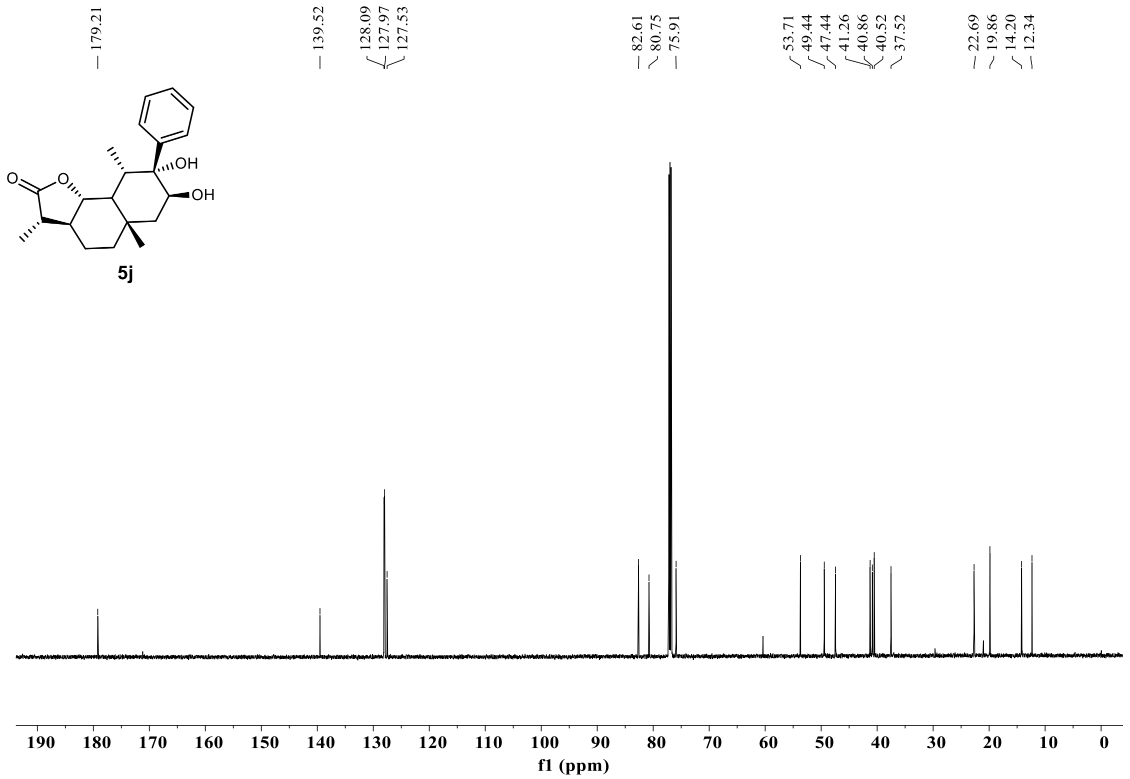

^{13}C NMR (151 MHz, $CDCl_3$)

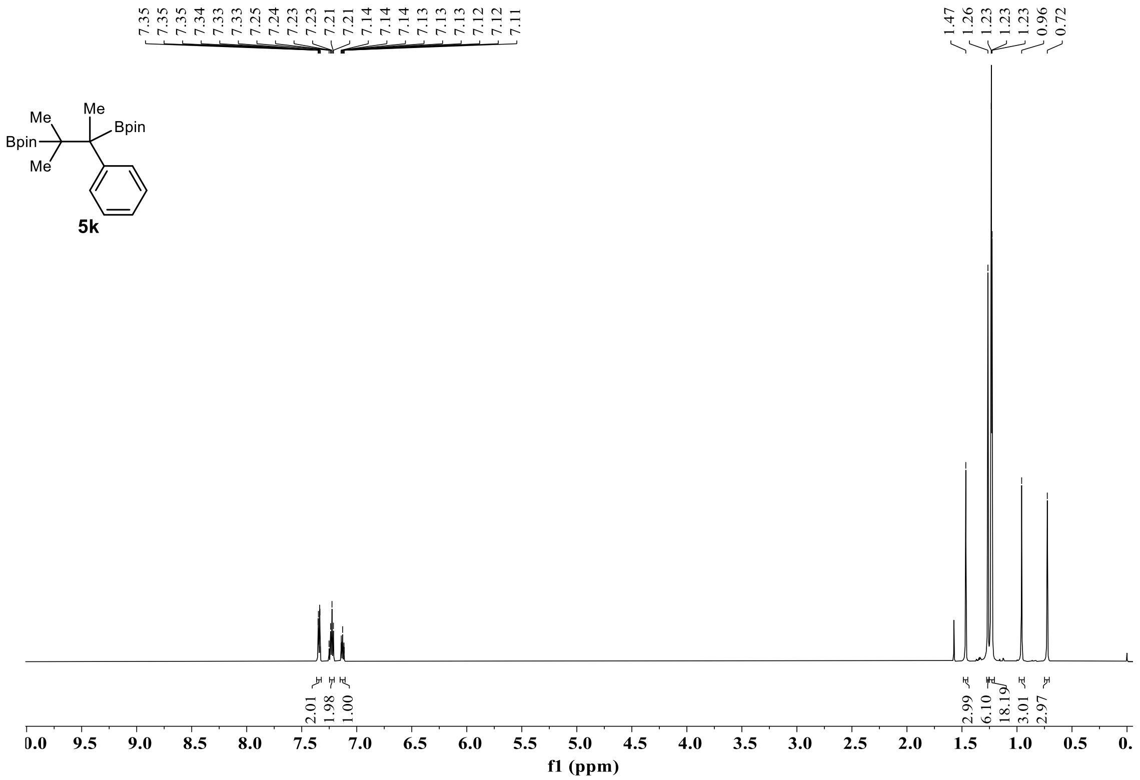

^{1}H NMR (600 MHz, $CDCl_3$)

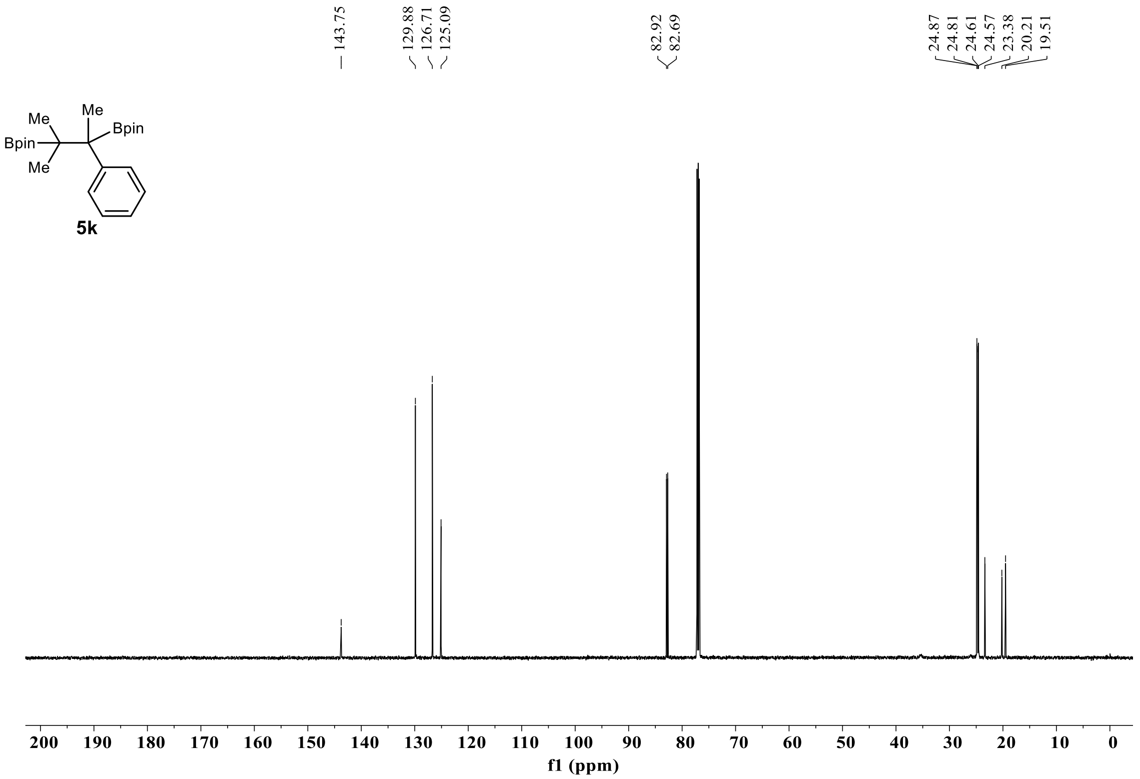

^{13}C NMR (151 MHz, $CDCl_3$)

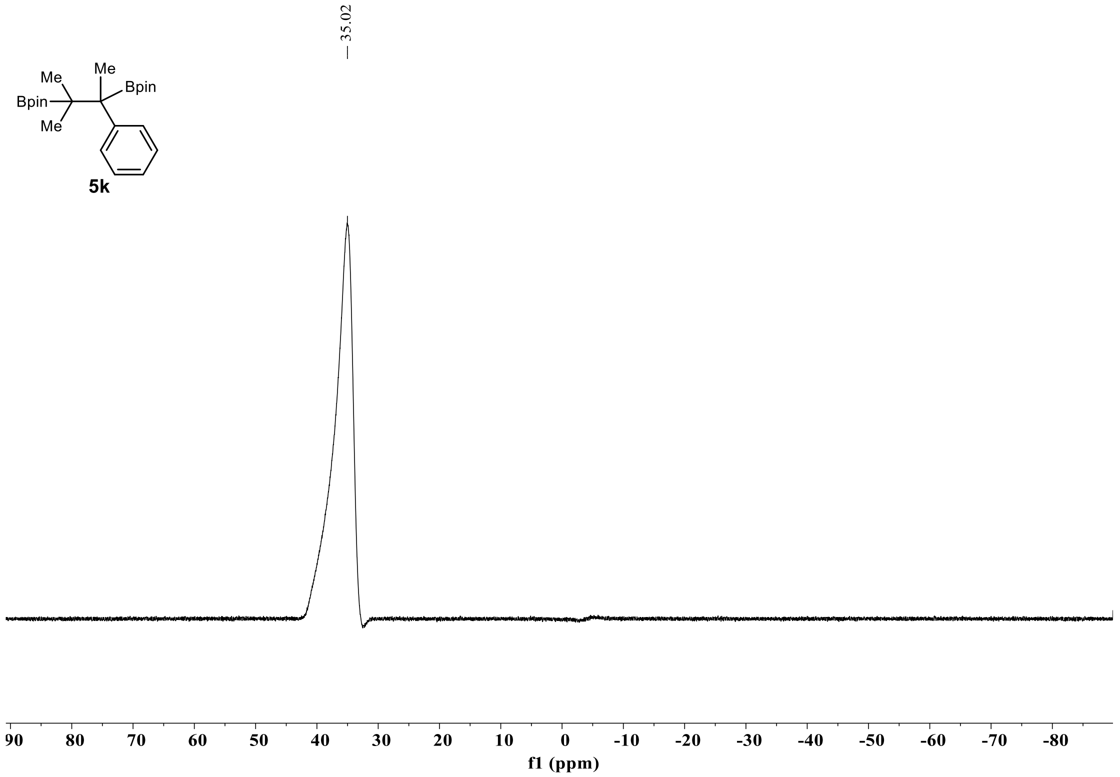

^{11}B NMR (193 MHz, $CDCl_3$)

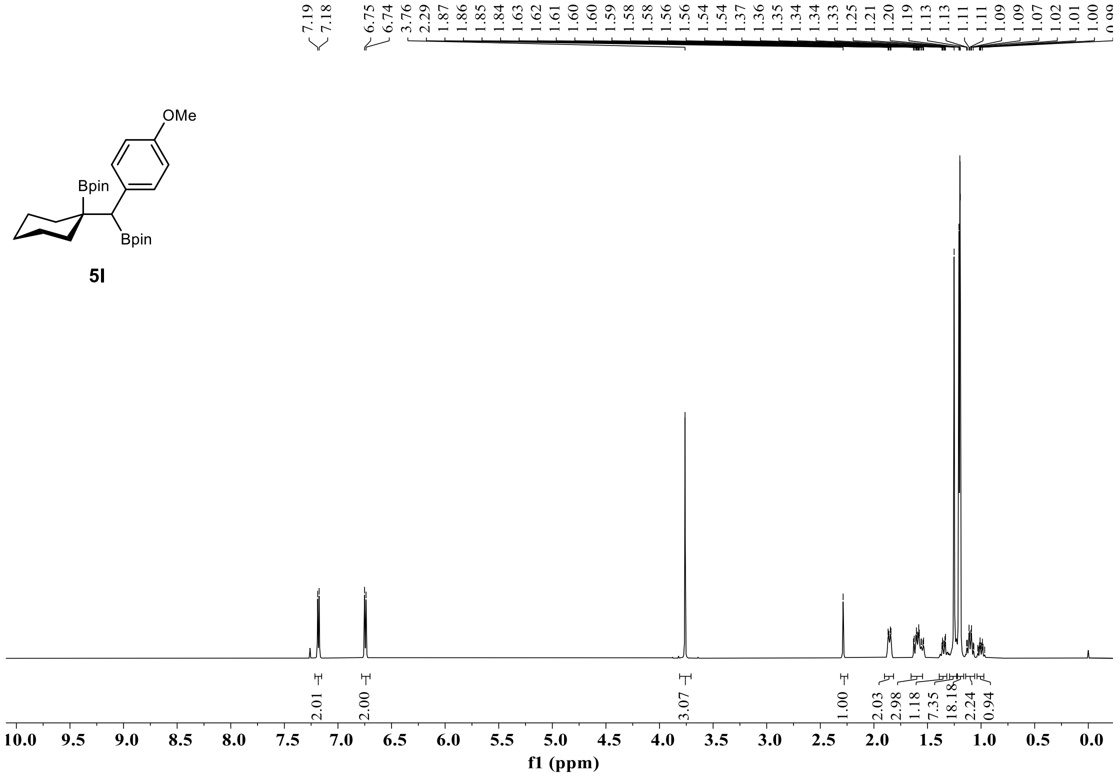

^{1}H NMR (600 MHz, $CDCl_3$)

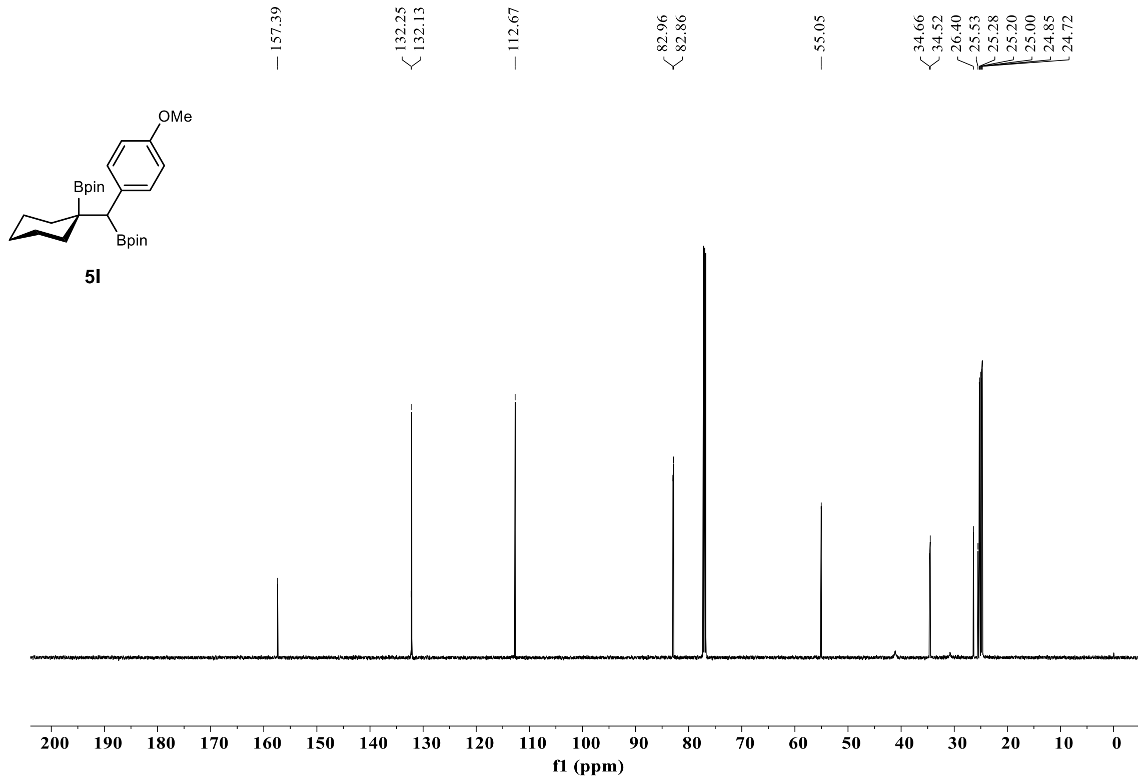

^{13}C NMR (151 MHz, $CDCl_3$)

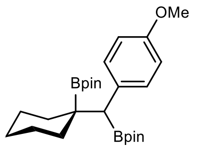

5l

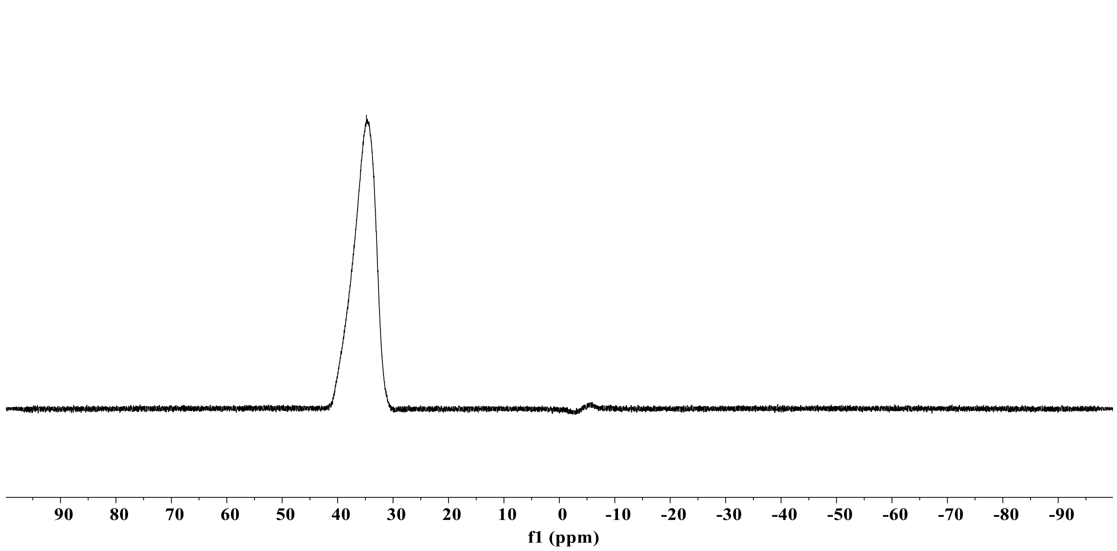

^{11}B NMR (193 MHz, CDCl$_3$)

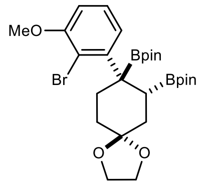

5l

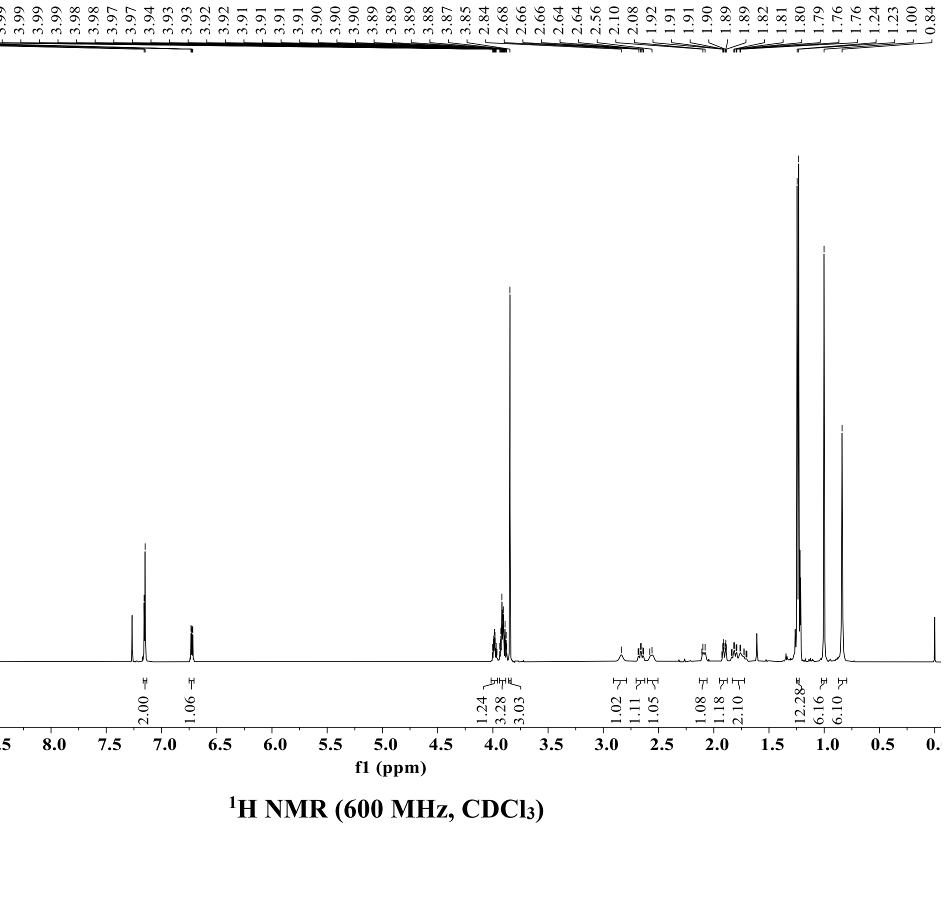

^{1}H NMR (600 MHz, CDCl$_3$)

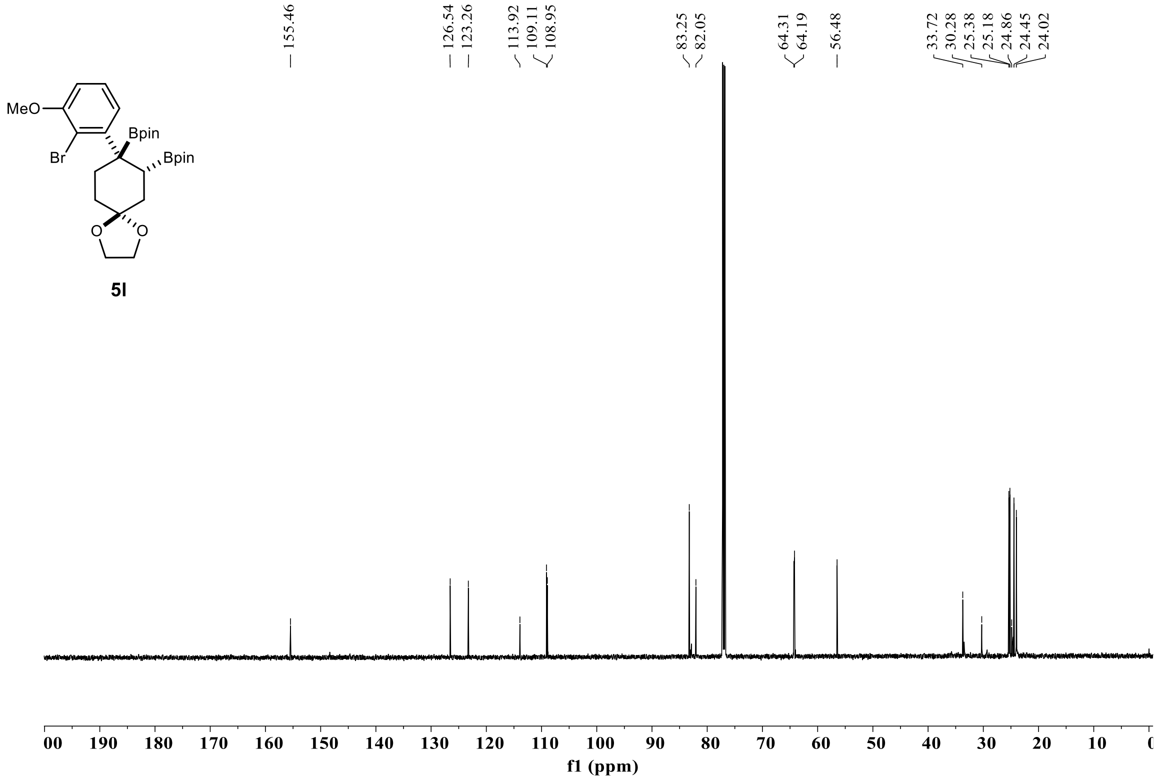

^{13}C NMR (151 MHz, $CDCl_3$)

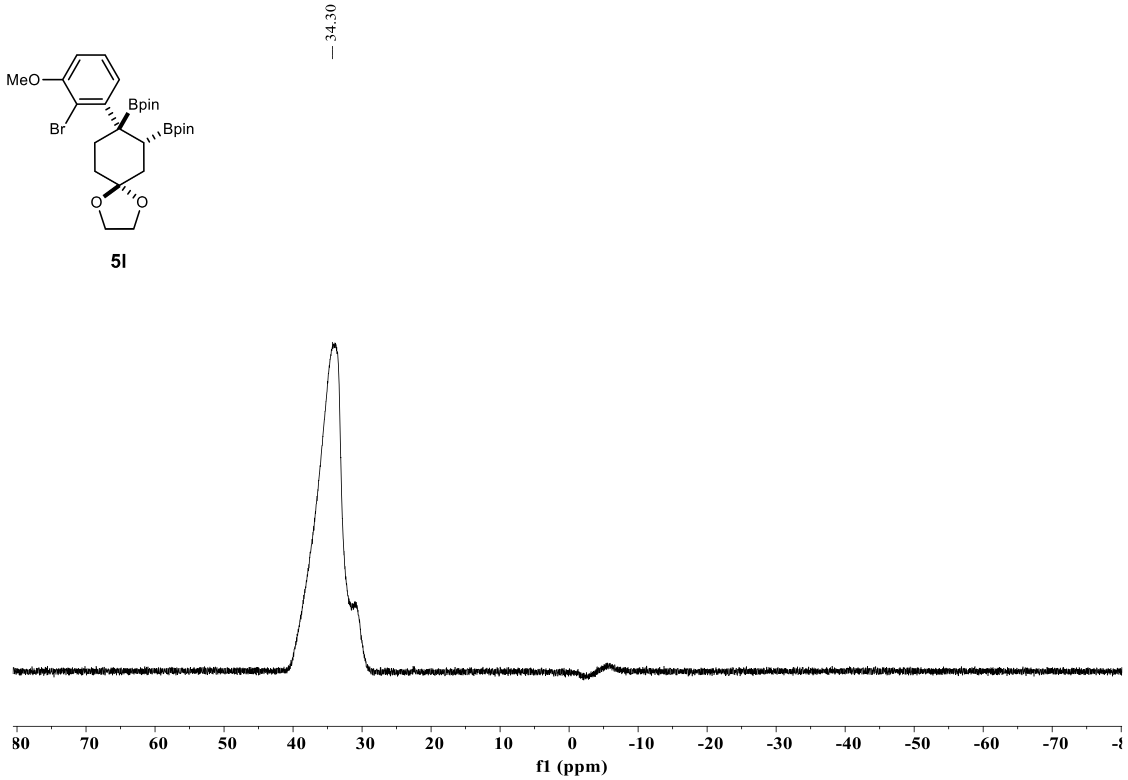

^{11}B NMR (193 MHz, $CDCl_3$)

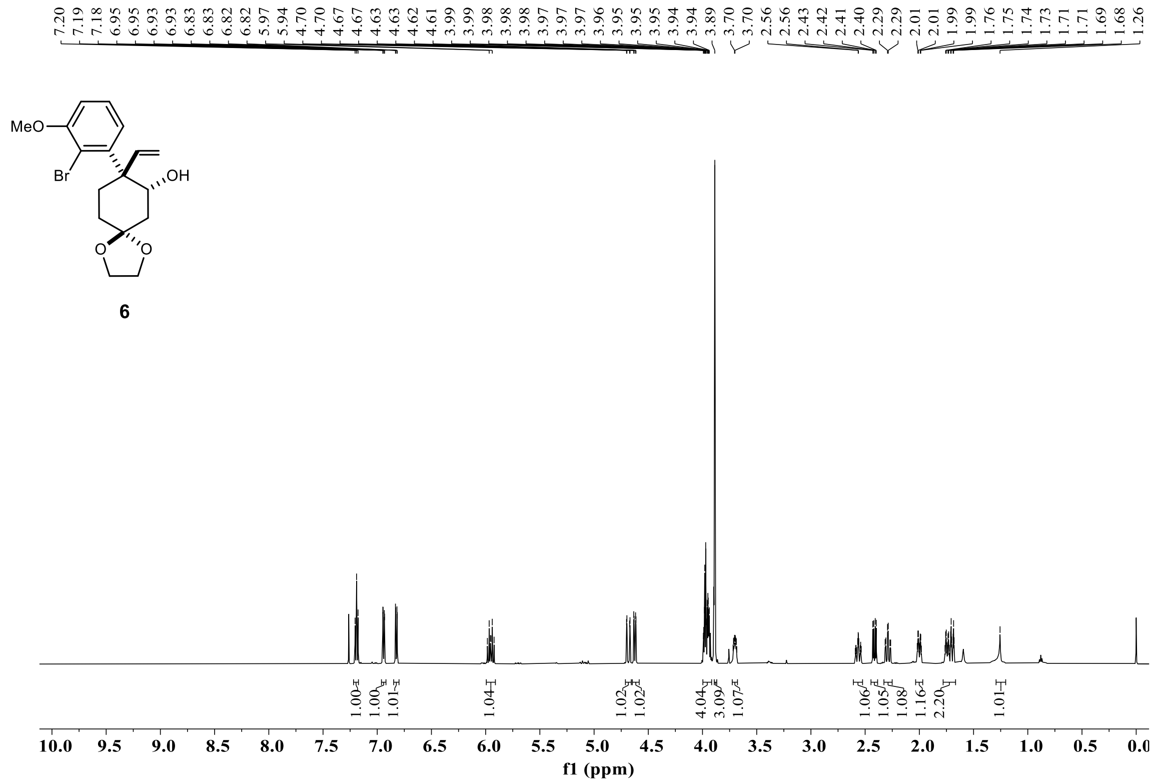

^{1}H NMR (600 MHz, CDCl$_3$)

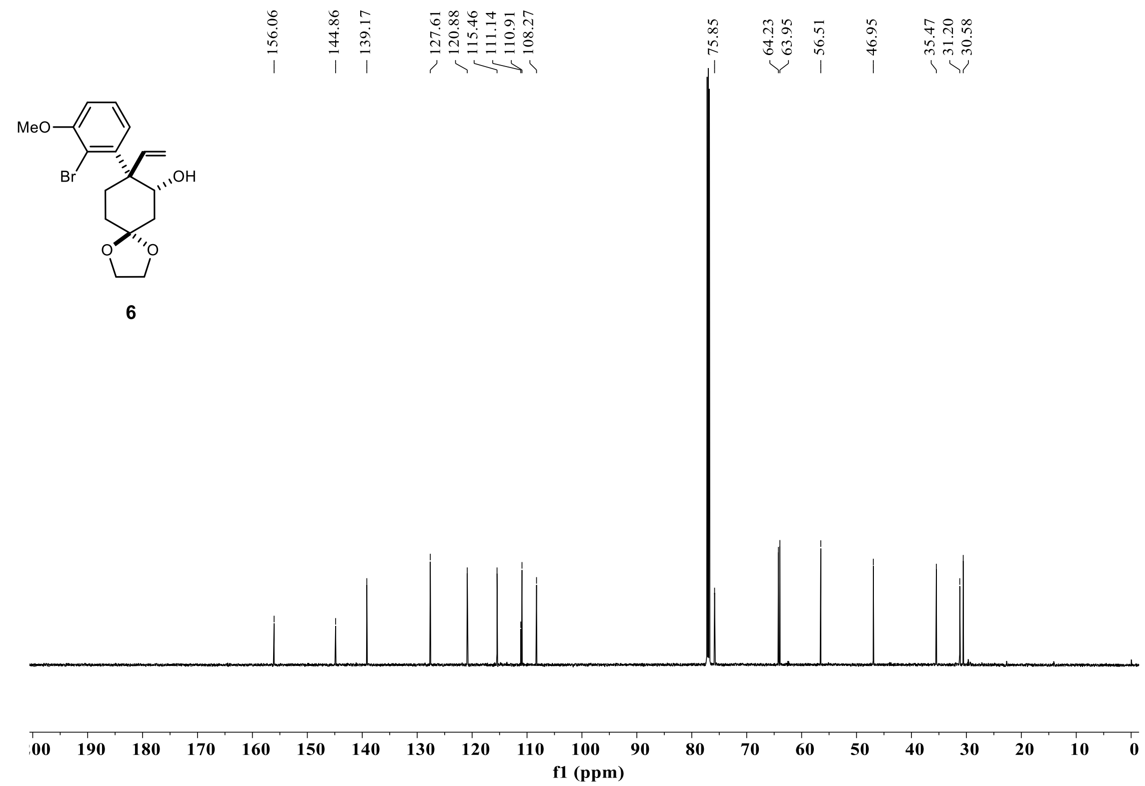

^{13}C NMR (151 MHz, CDCl$_3$)

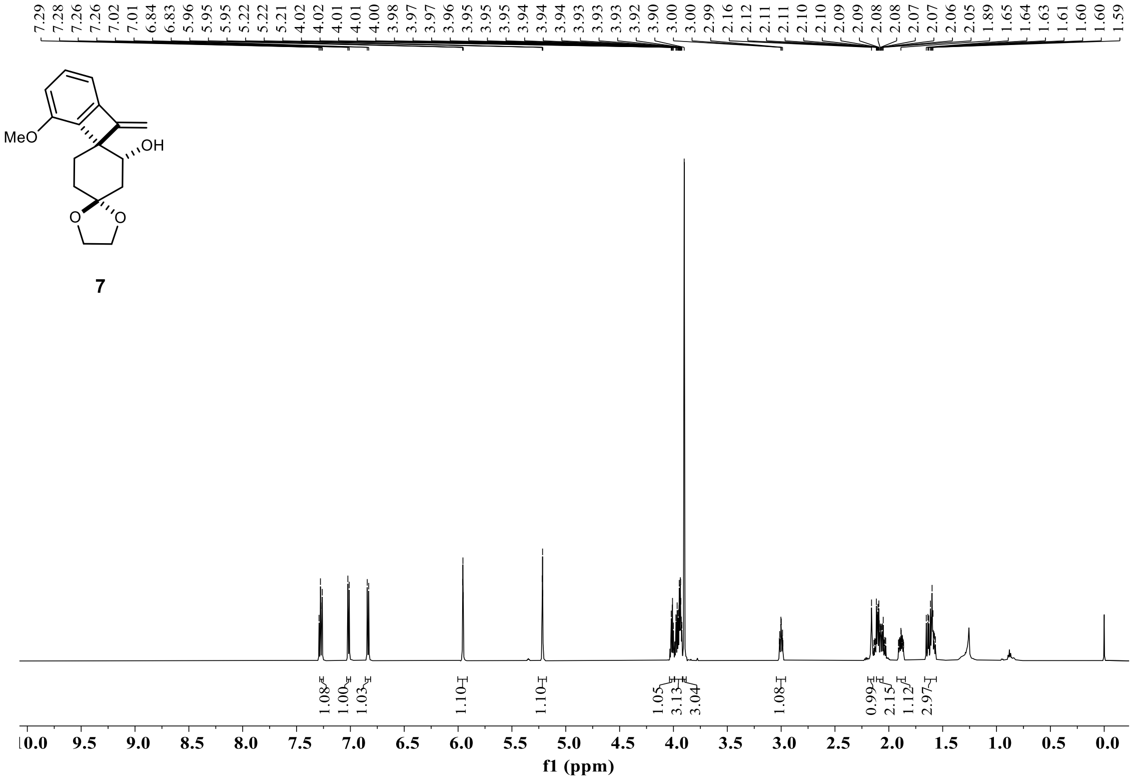

^{1}H NMR (600 MHz, CDCl₃)

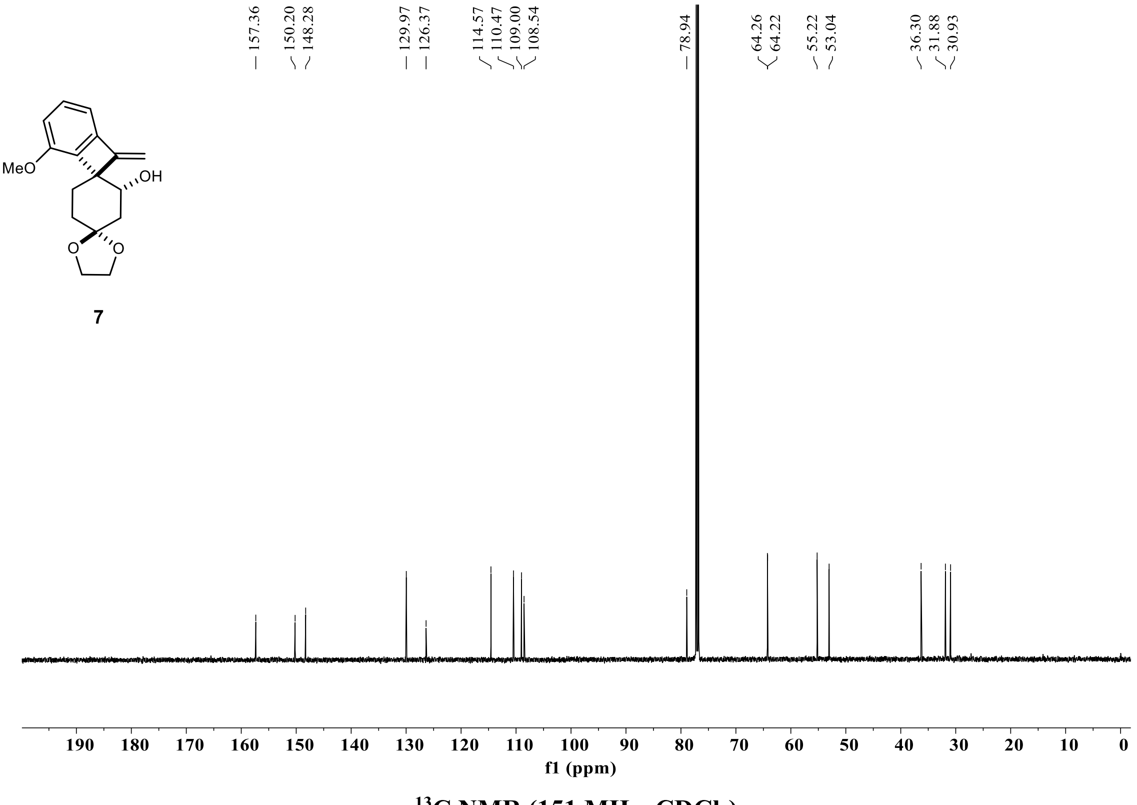

^{13}C NMR (151 MHz, CDCl₃)

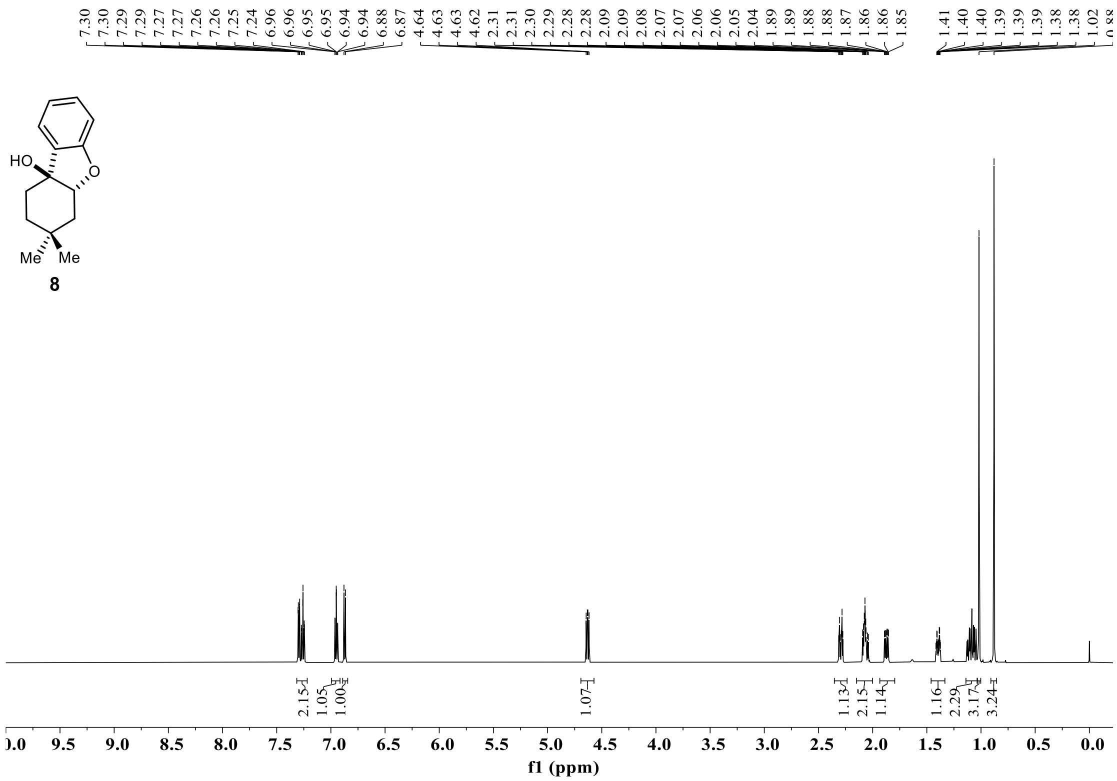

^{1}H NMR (600 MHz, $CDCl_3$)

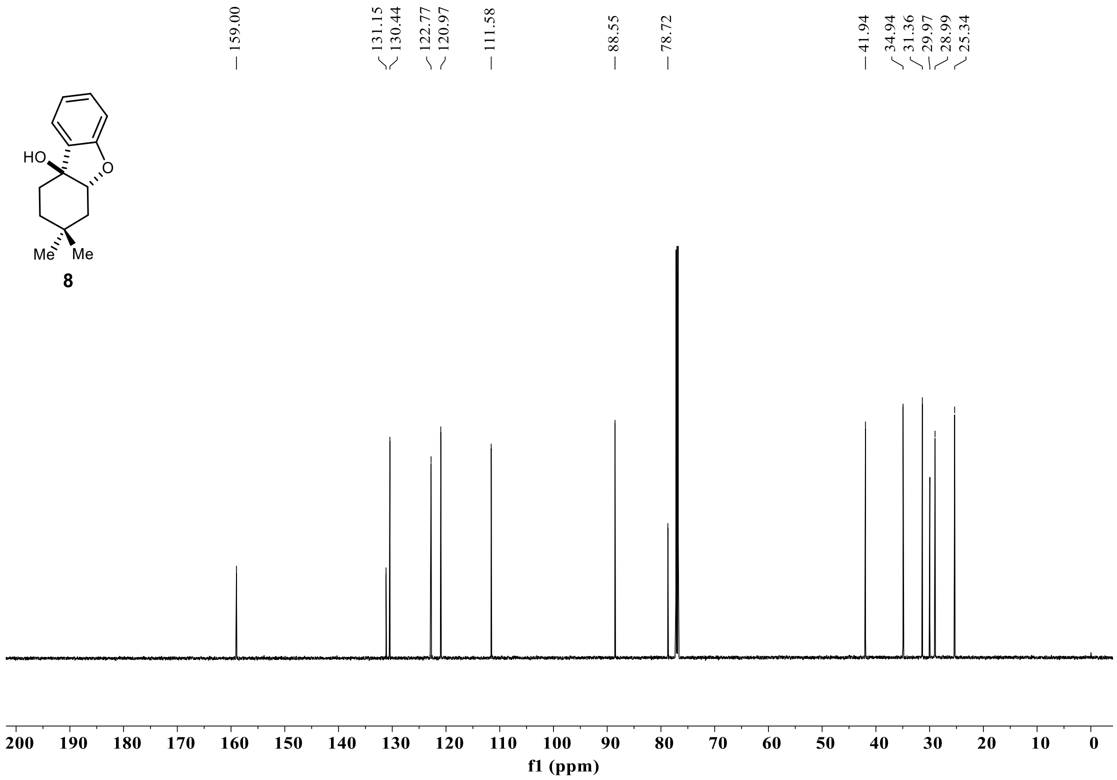

^{13}C NMR (151 MHz, $CDCl_3$)

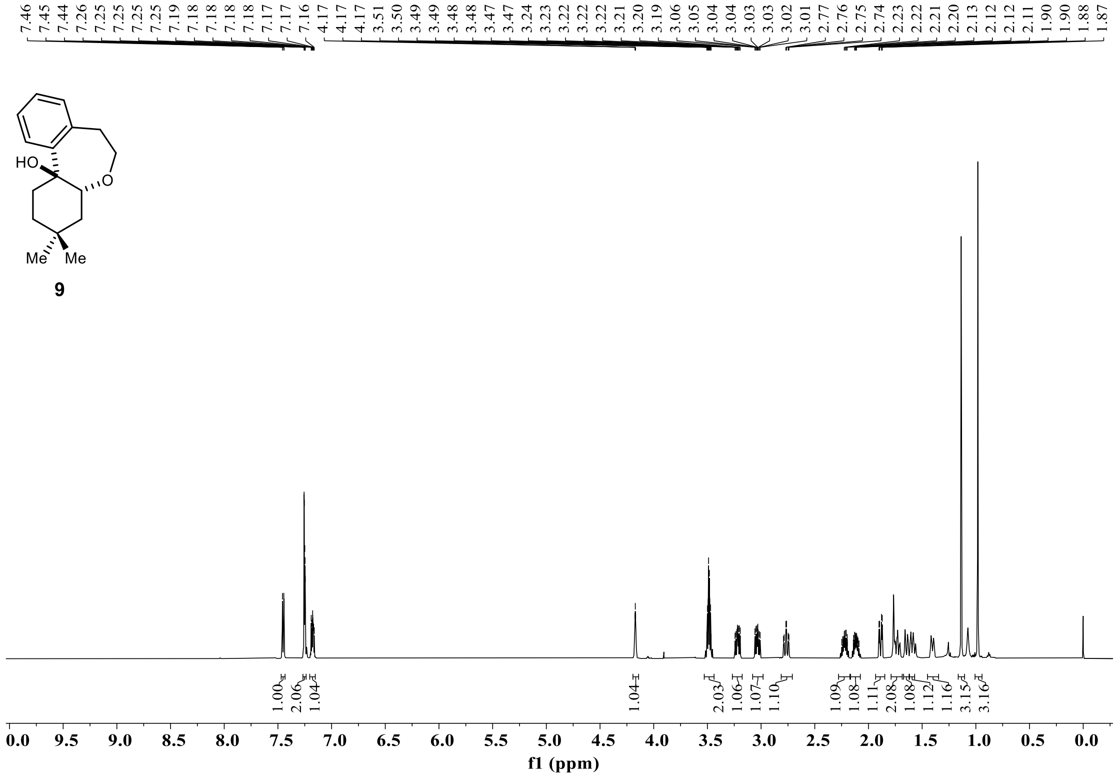

^{1}H NMR (600 MHz, CDCl$_3$)

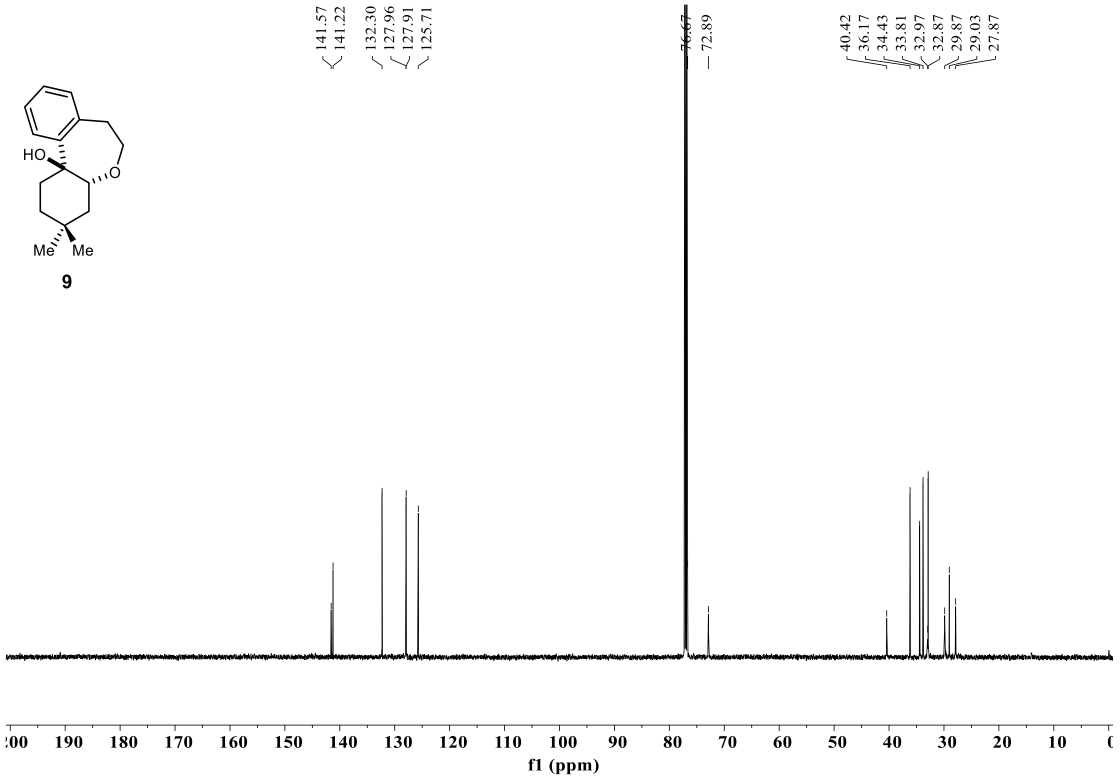

^{13}C NMR (151 MHz, CDCl$_3$)

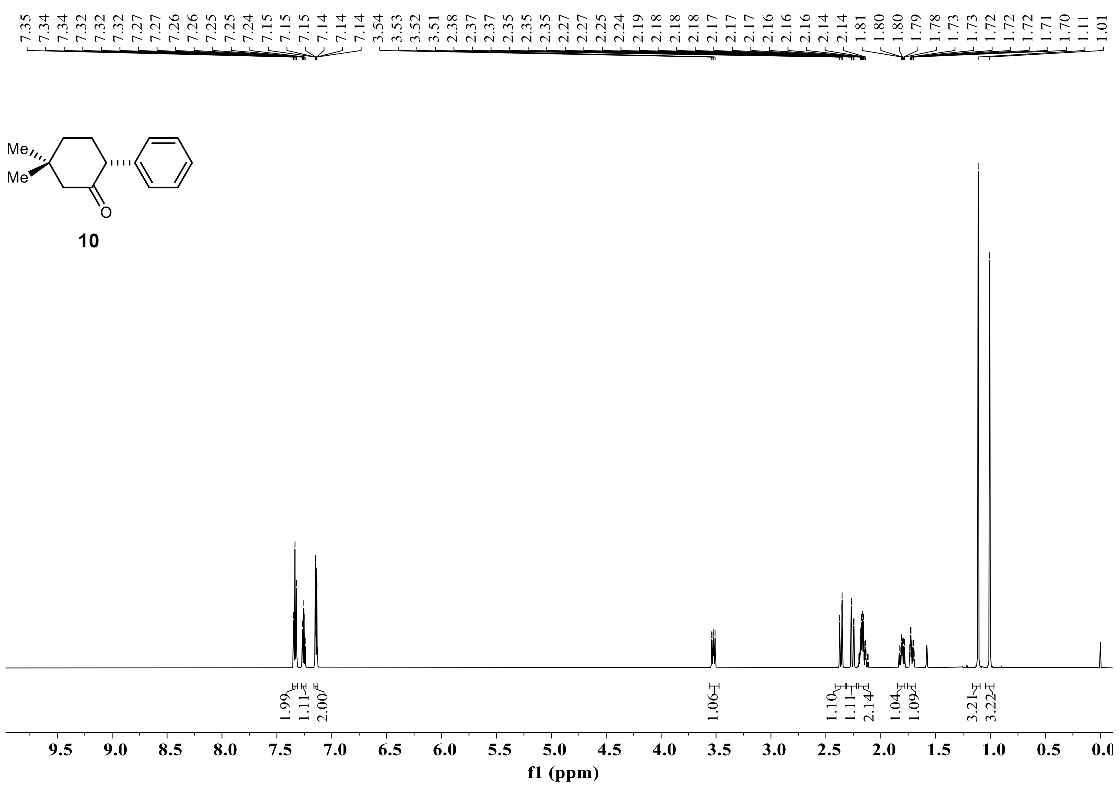

^{1}H NMR (600 MHz, CDCl3)

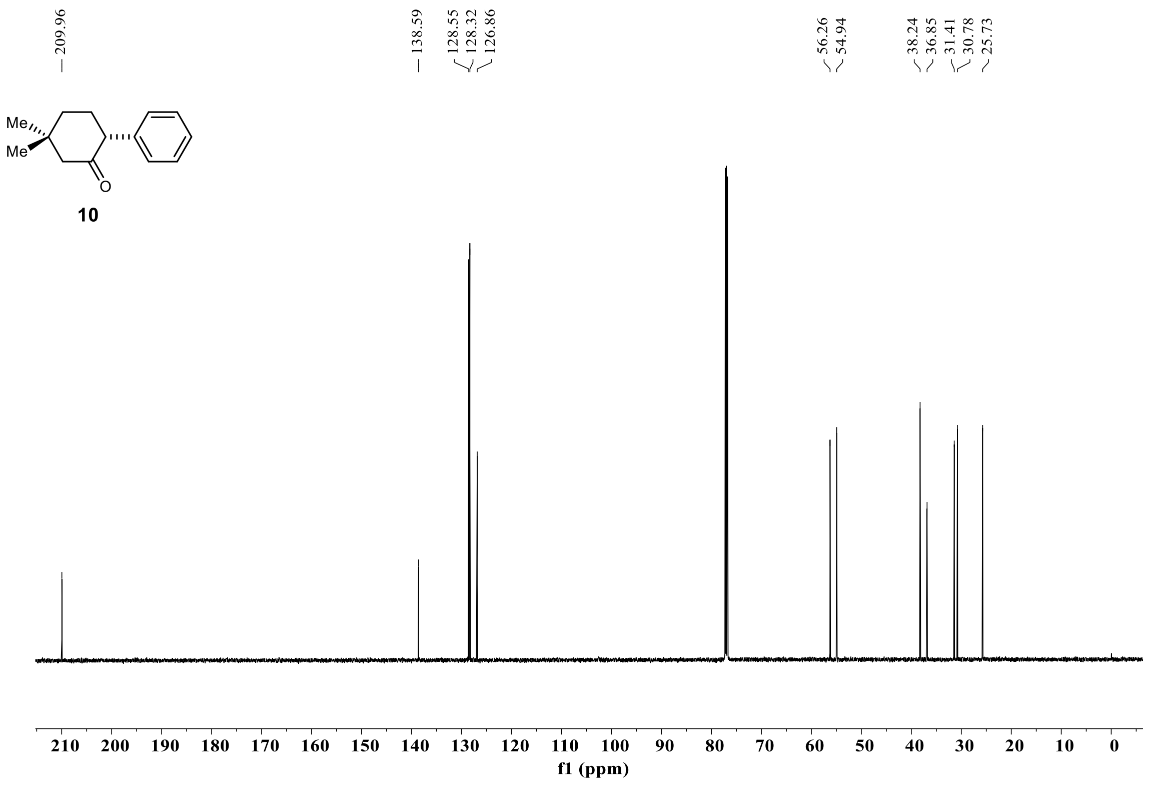

^{13}C NMR (151 MHz, CDCl3)

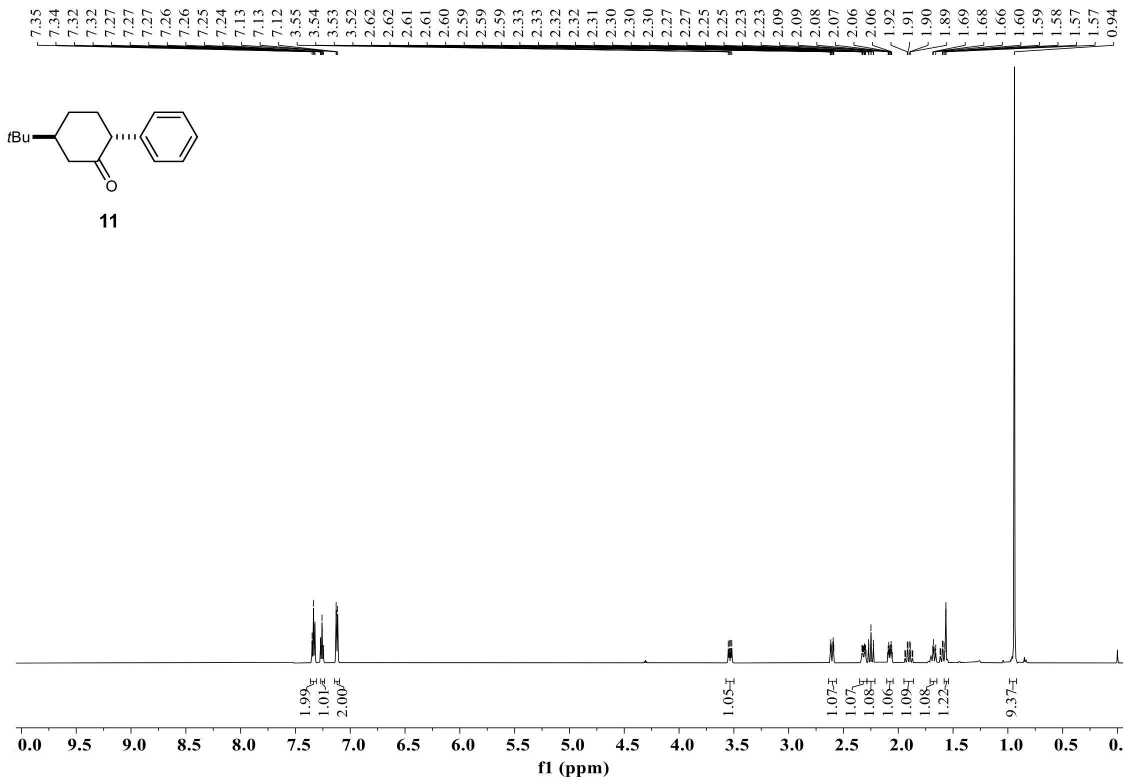

^{1}H NMR (600 MHz, CDCl$_3$)

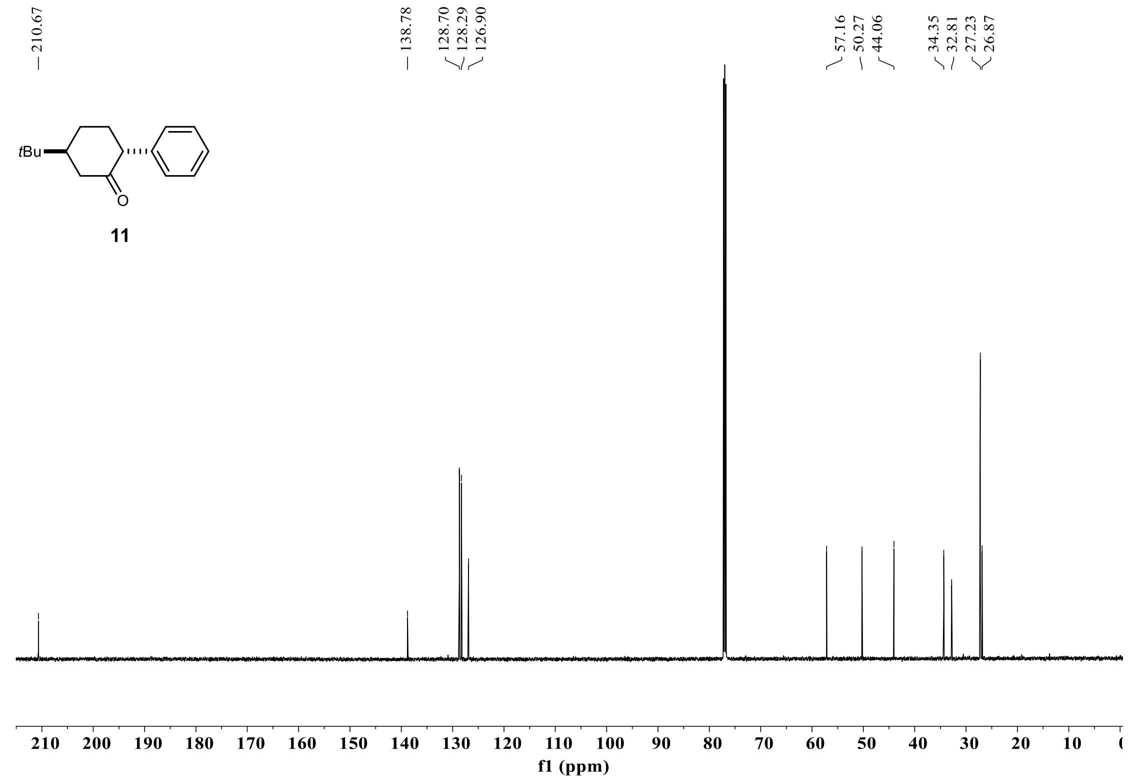

^{13}C NMR (151 MHz, CDCl$_3$)

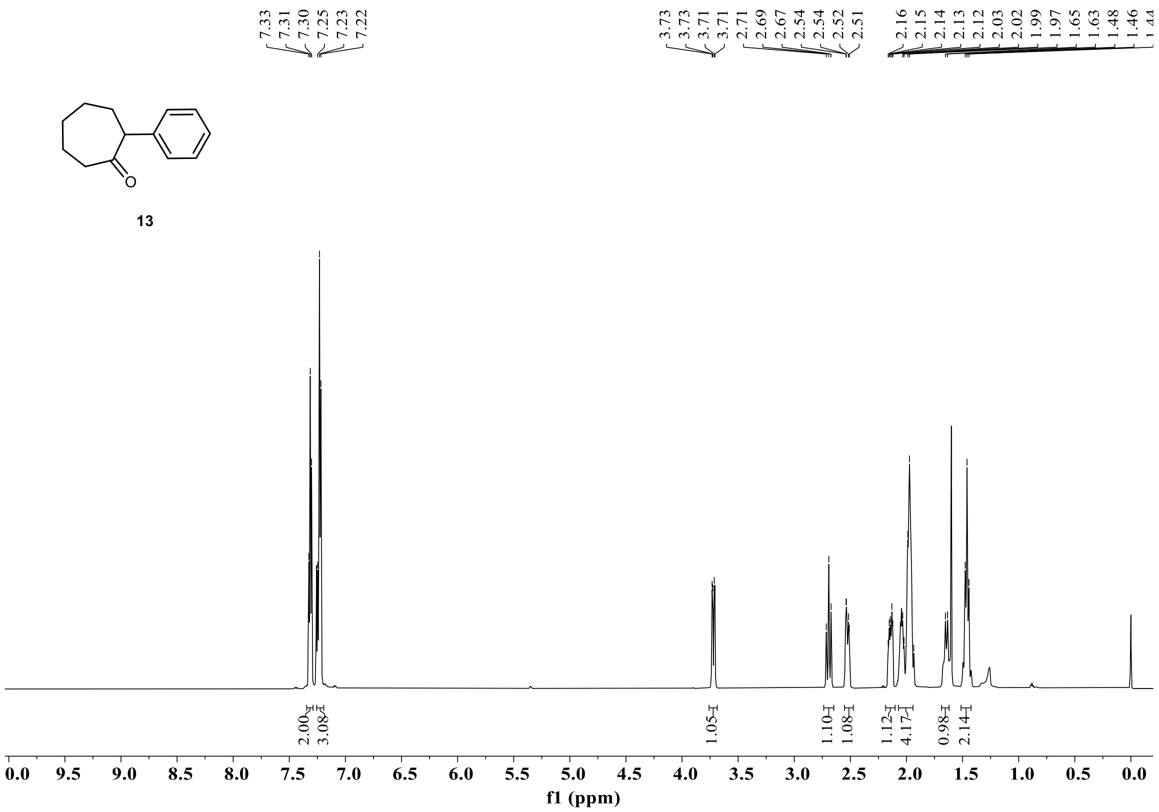

^{1}H NMR (600 MHz, $CDCl_3$)

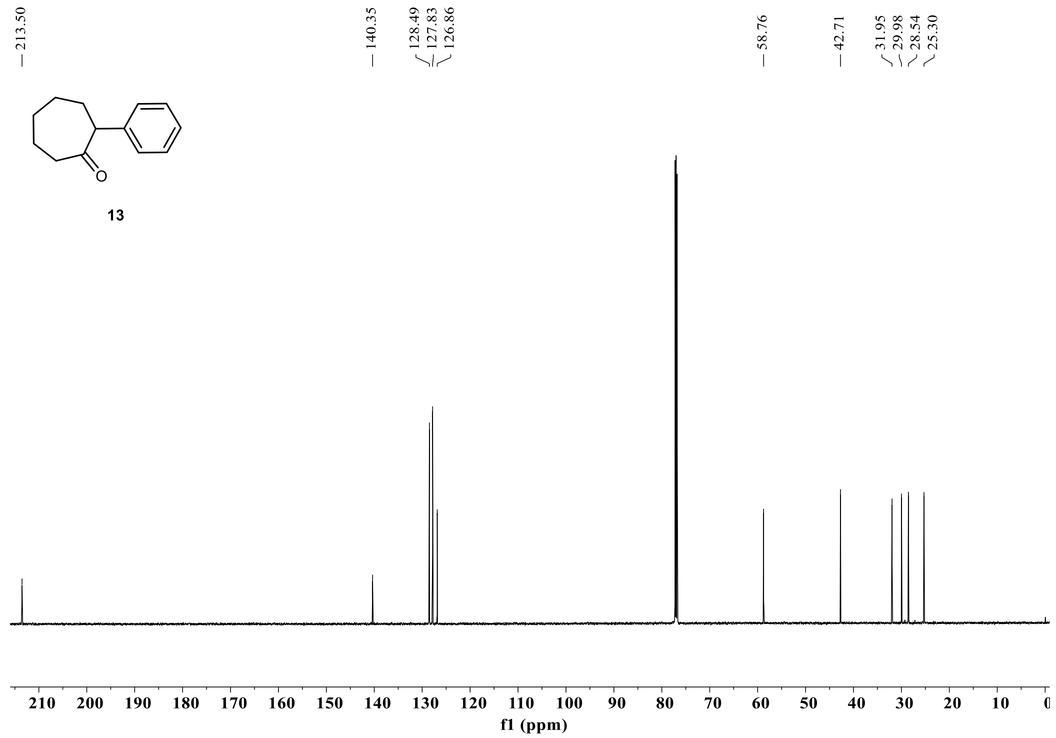

^{13}C NMR (151 MHz, $CDCl_3$)

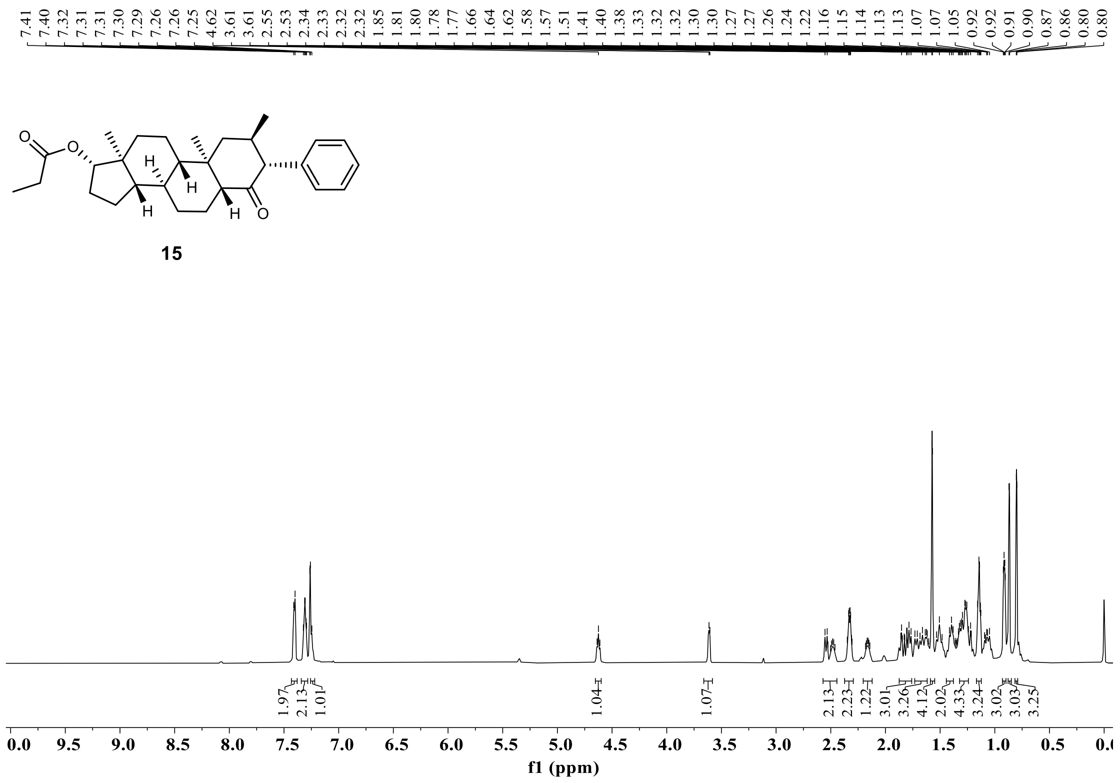

^{1}H NMR (600 MHz, CDCl3)

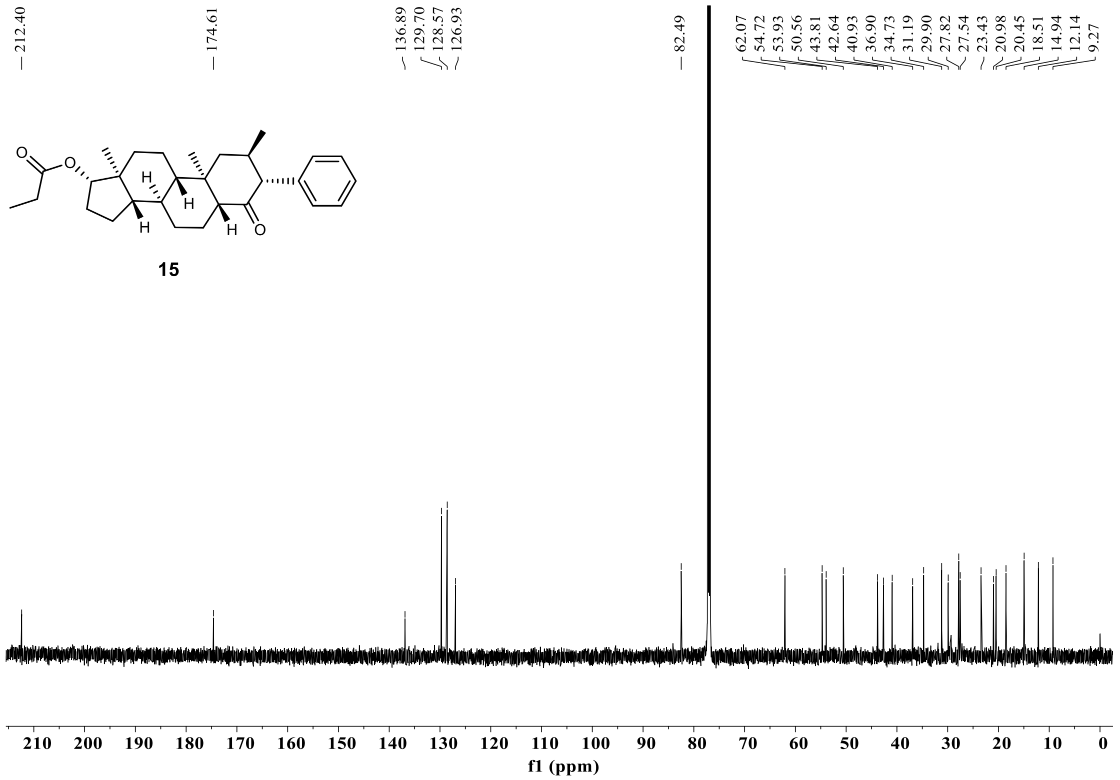

^{13}C NMR (151 MHz, CDCl3)

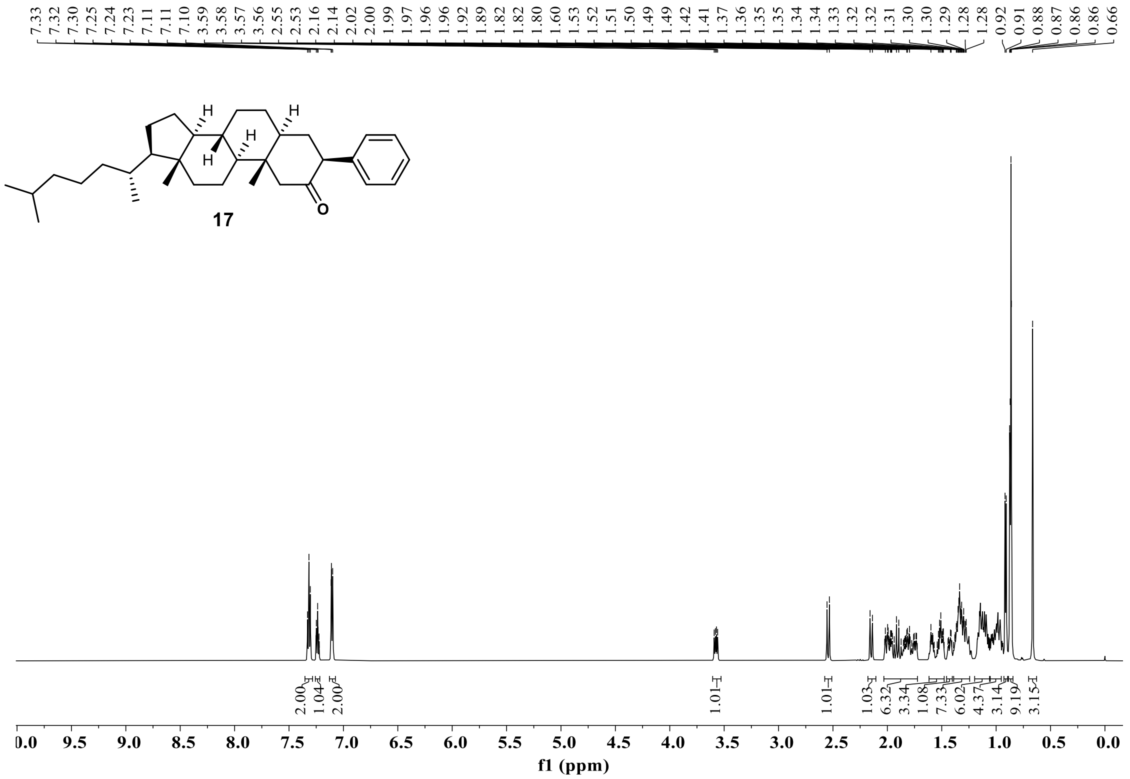

^{1}H NMR (600 MHz, $CDCl_3$)

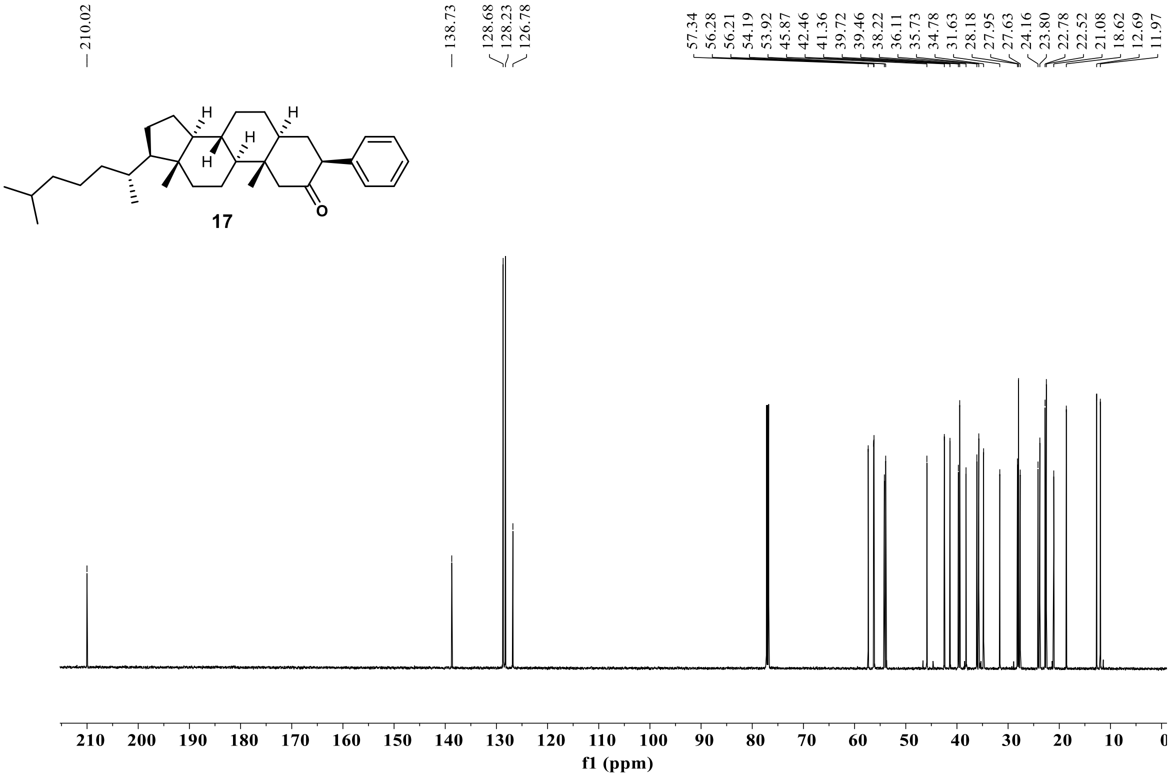

^{13}C NMR (151 MHz, $CDCl_3$)

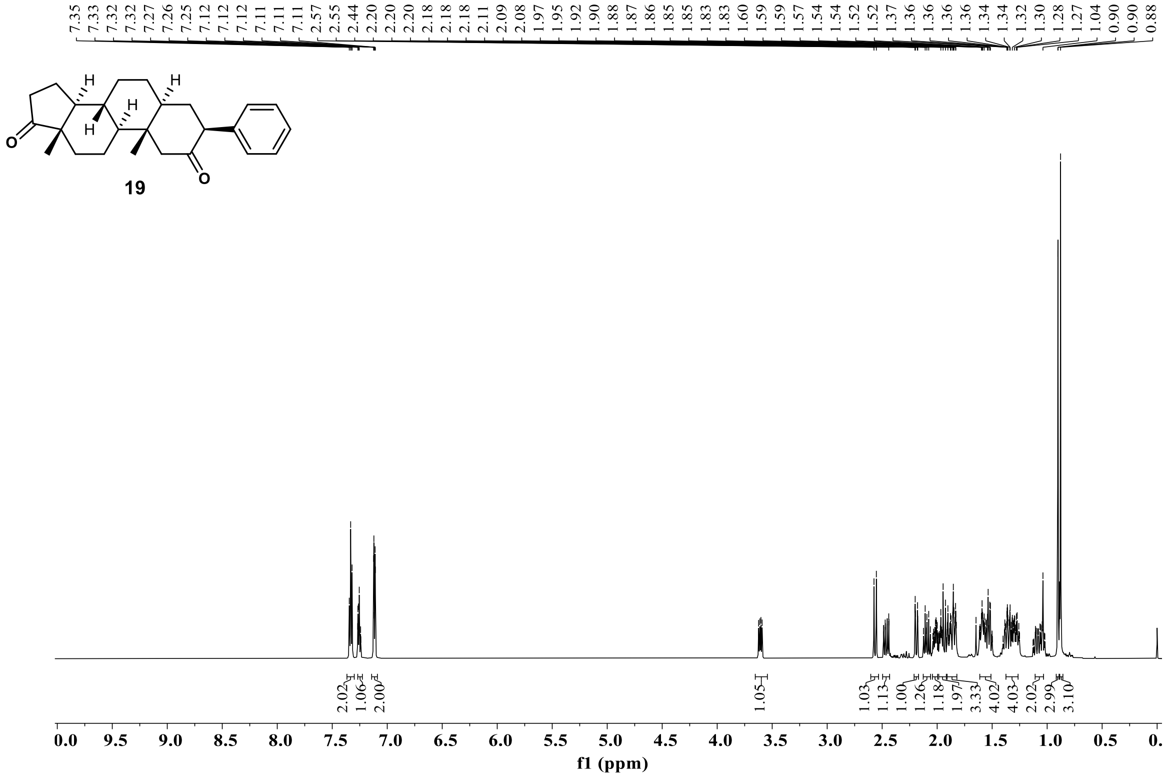

^{1}H NMR (600 MHz, CDCl3)

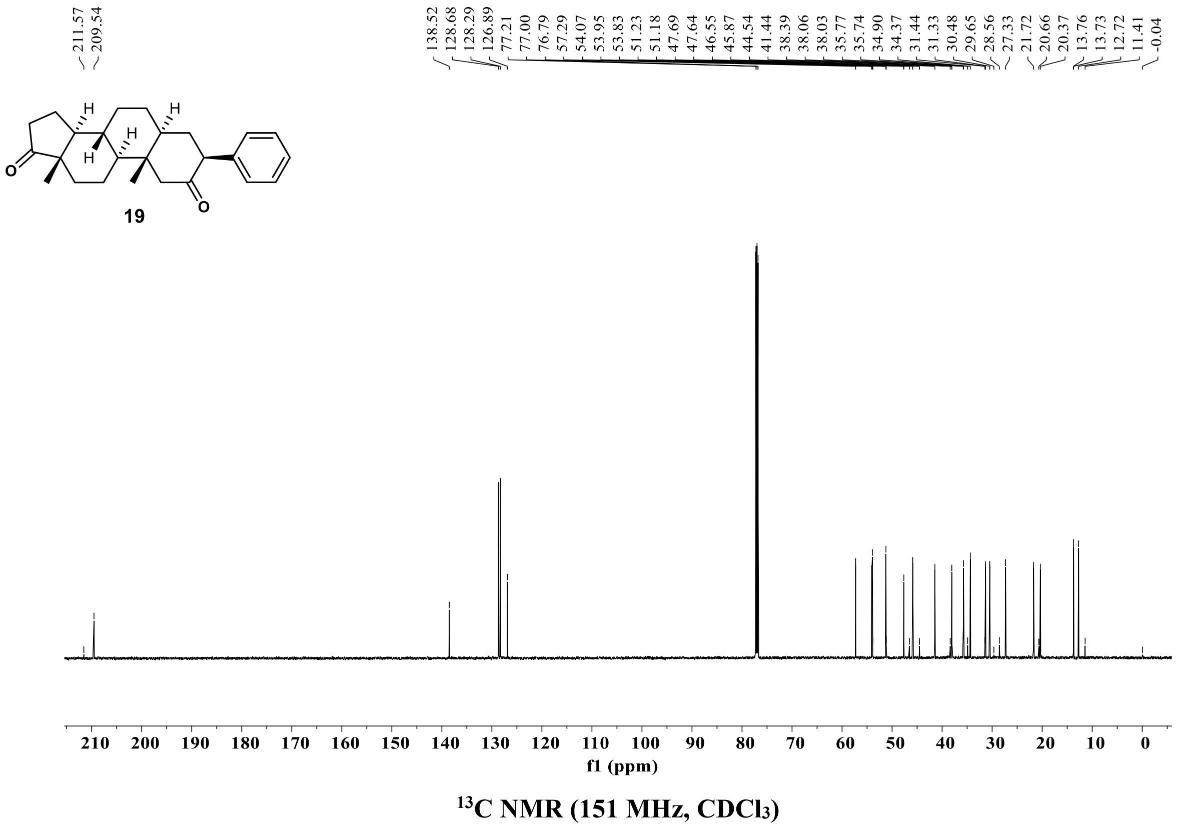

^{13}C NMR (151 MHz, CDCl3)

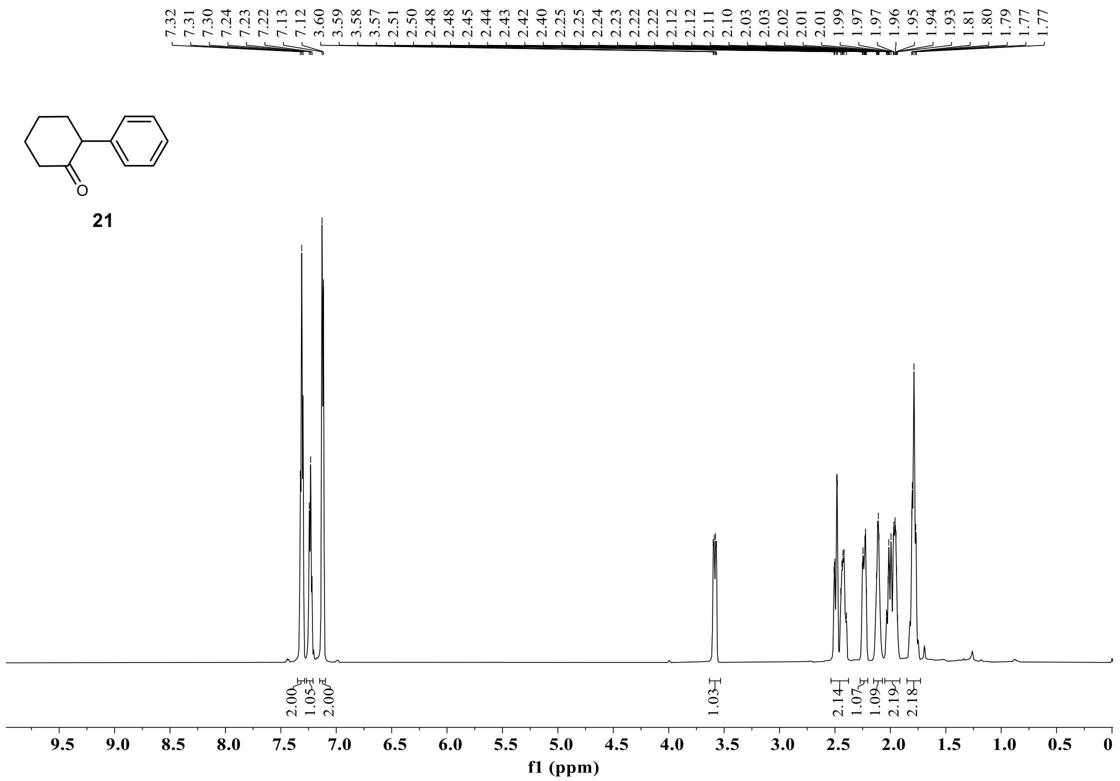

^{1}H NMR (600 MHz, $CDCl_3$)

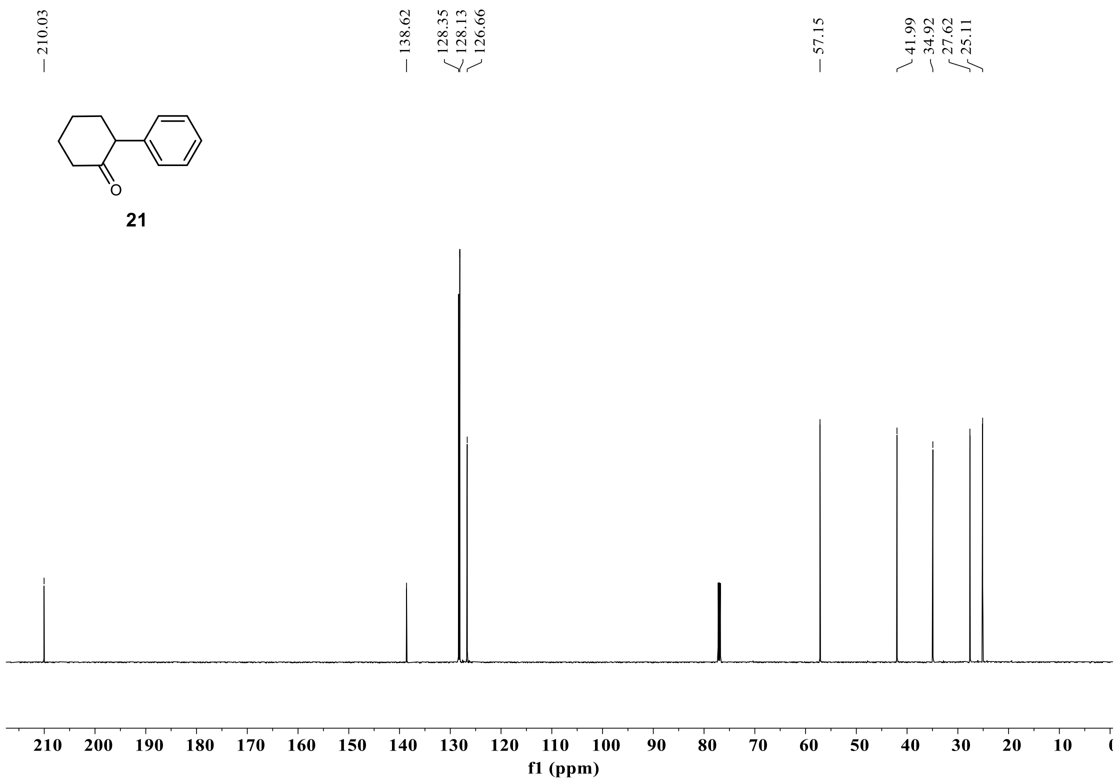

^{13}C NMR (151 MHz, $CDCl_3$)

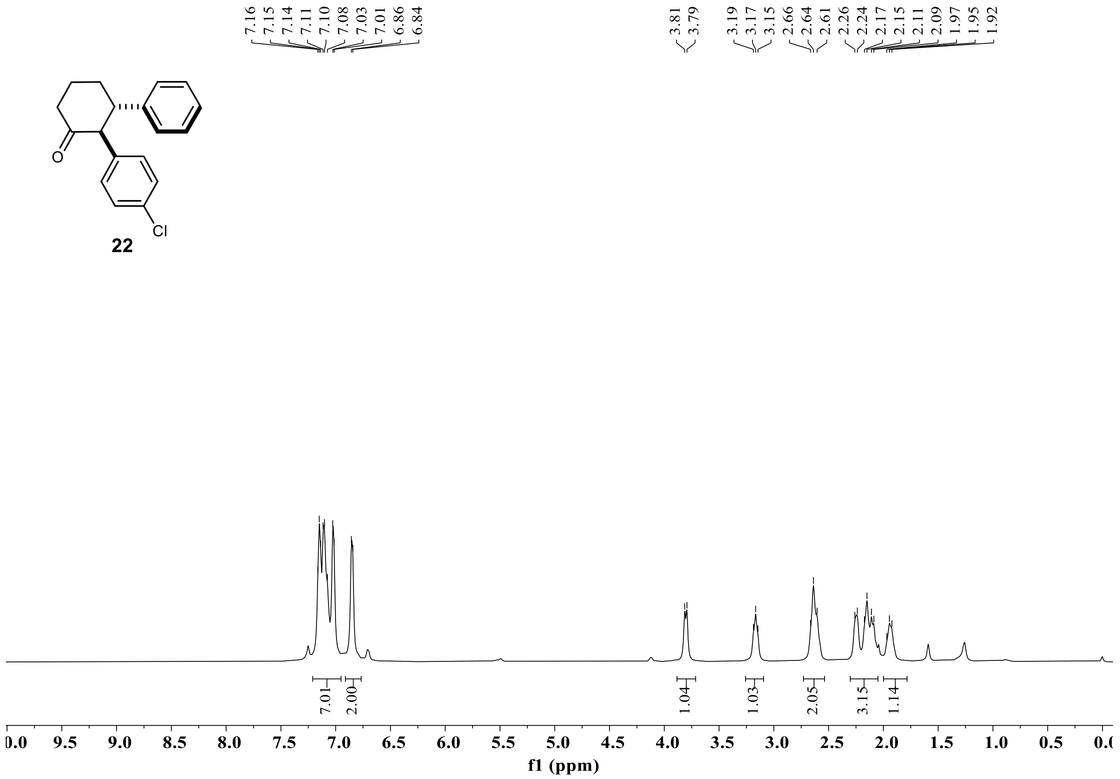

^{1}H NMR (600 MHz, CDCl₃)

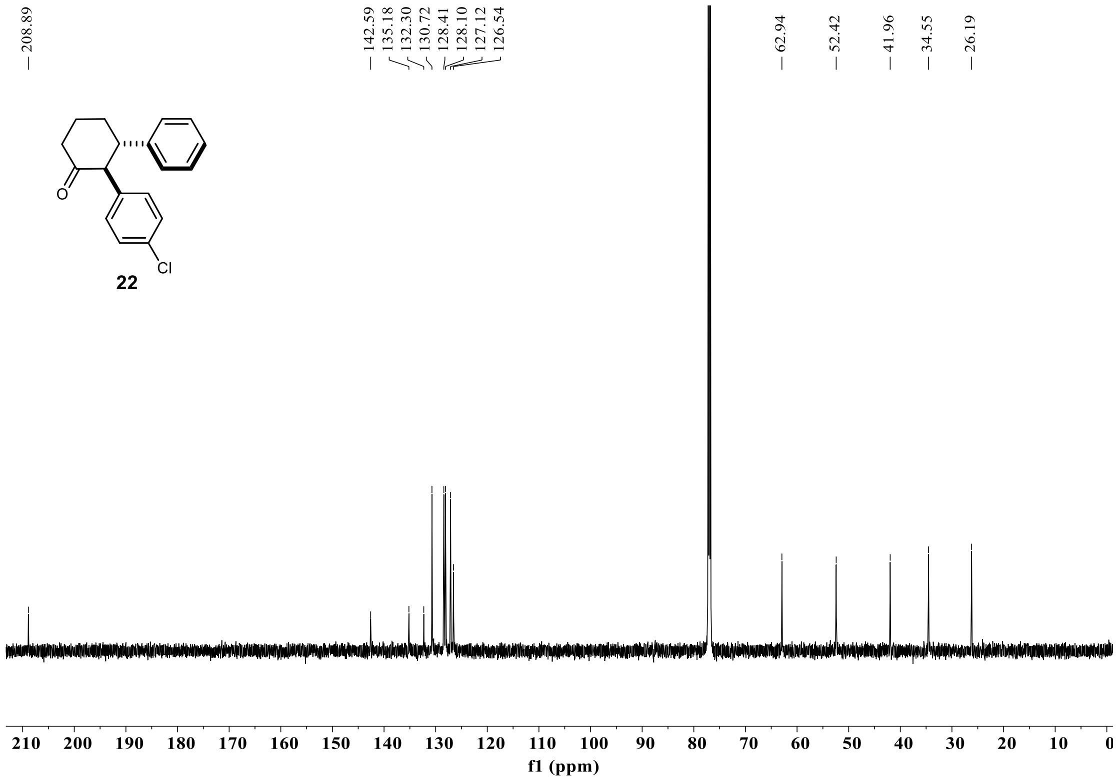

^{13}C NMR (151 MHz, CDCl₃)

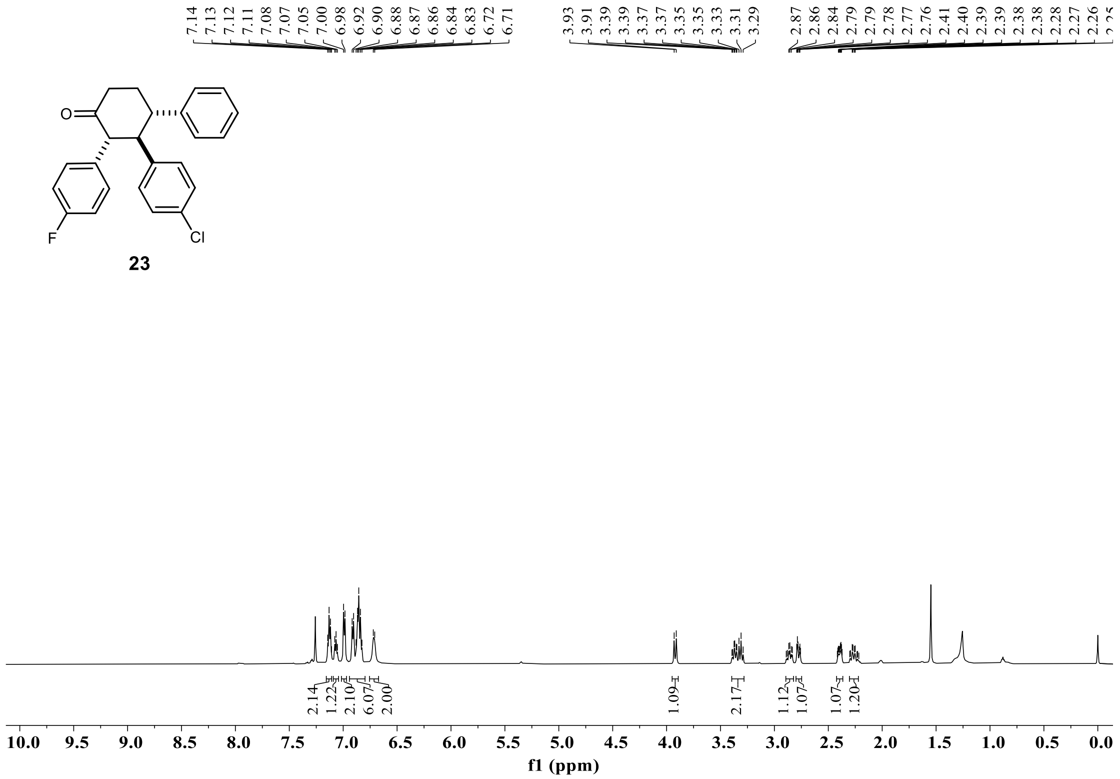

^{1}H NMR (600 MHz, CDCl$_3$)

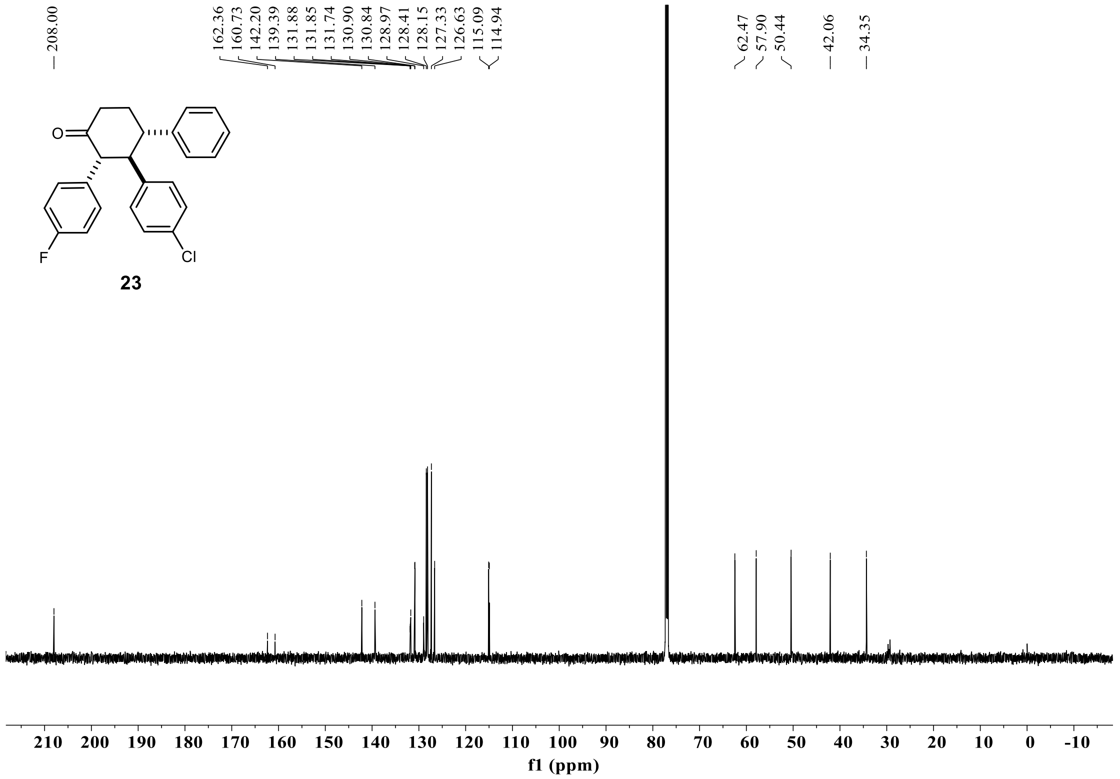

^{13}C NMR (151 MHz, CDCl$_3$)

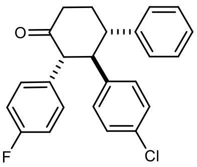

23

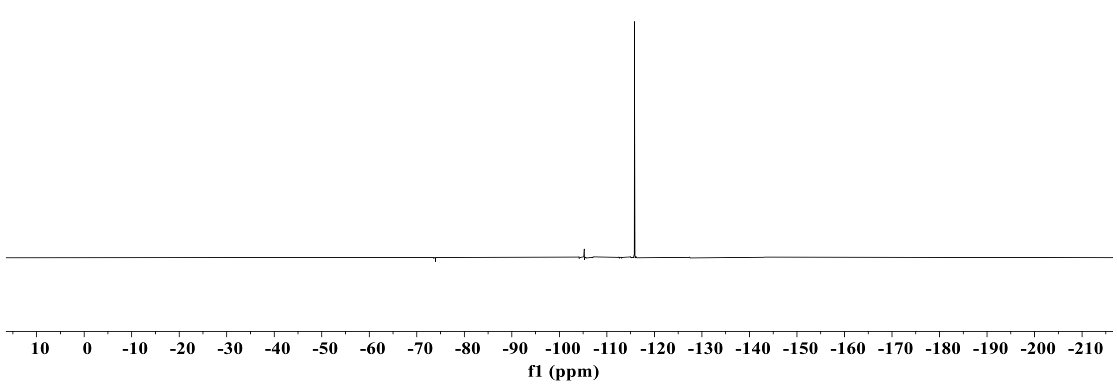

^{19}F NMR (565 MHz, CDCl$_3$)

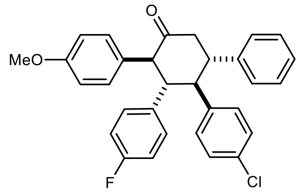

24

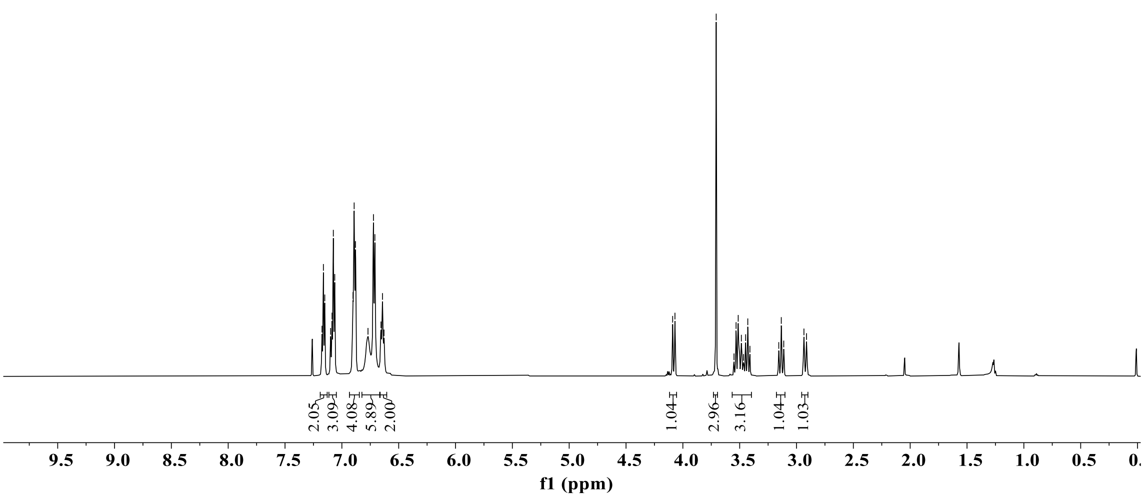

^{1}H NMR (600 MHz, CDCl$_3$)

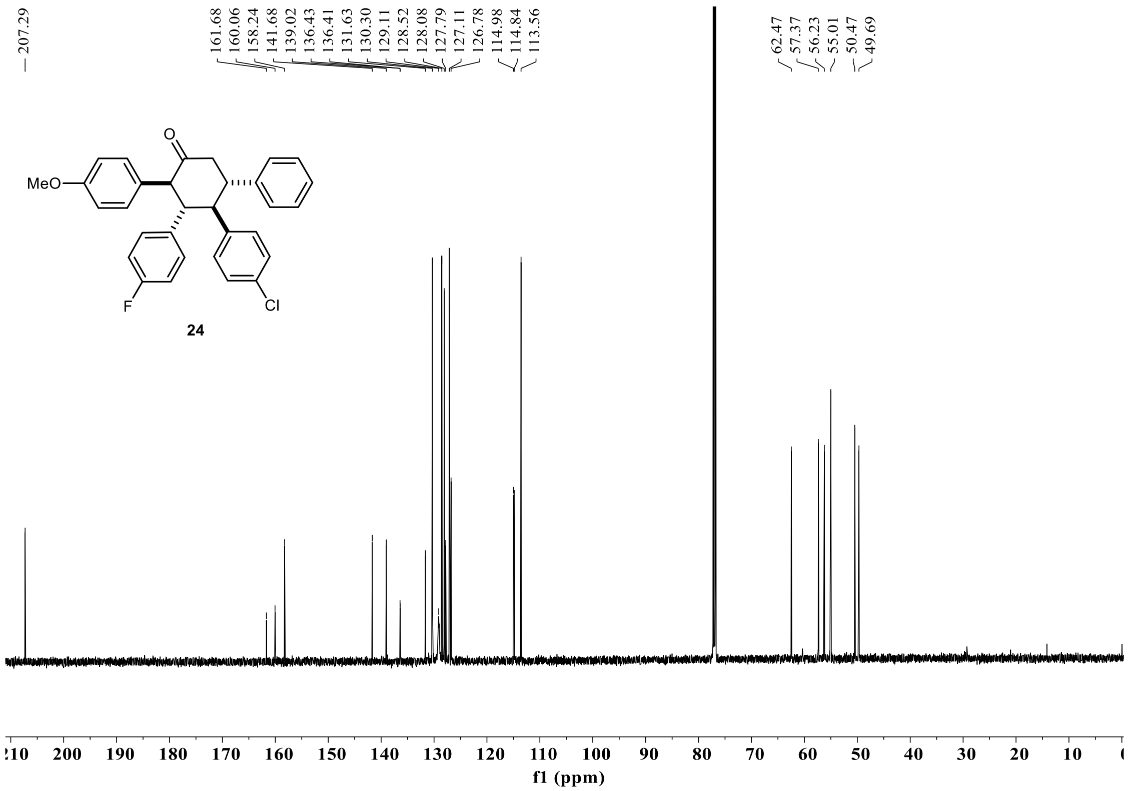

^{13}C NMR (151 MHz, $CDCl_3$)

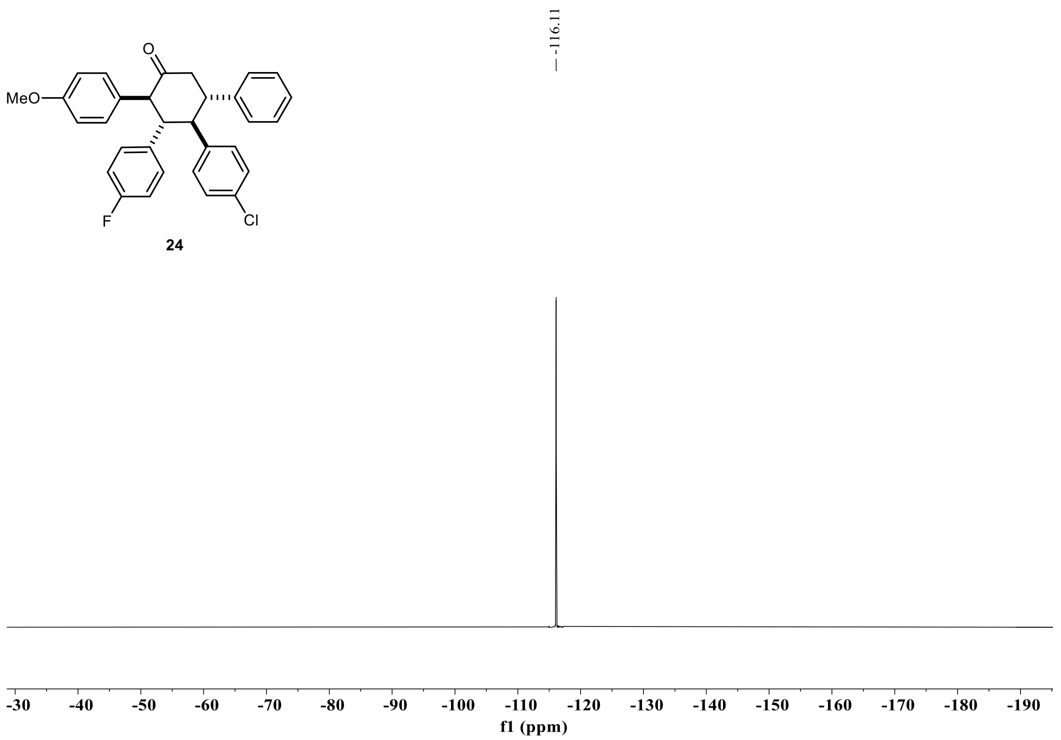

^{19}F NMR (565 MHz, $CDCl_3$)

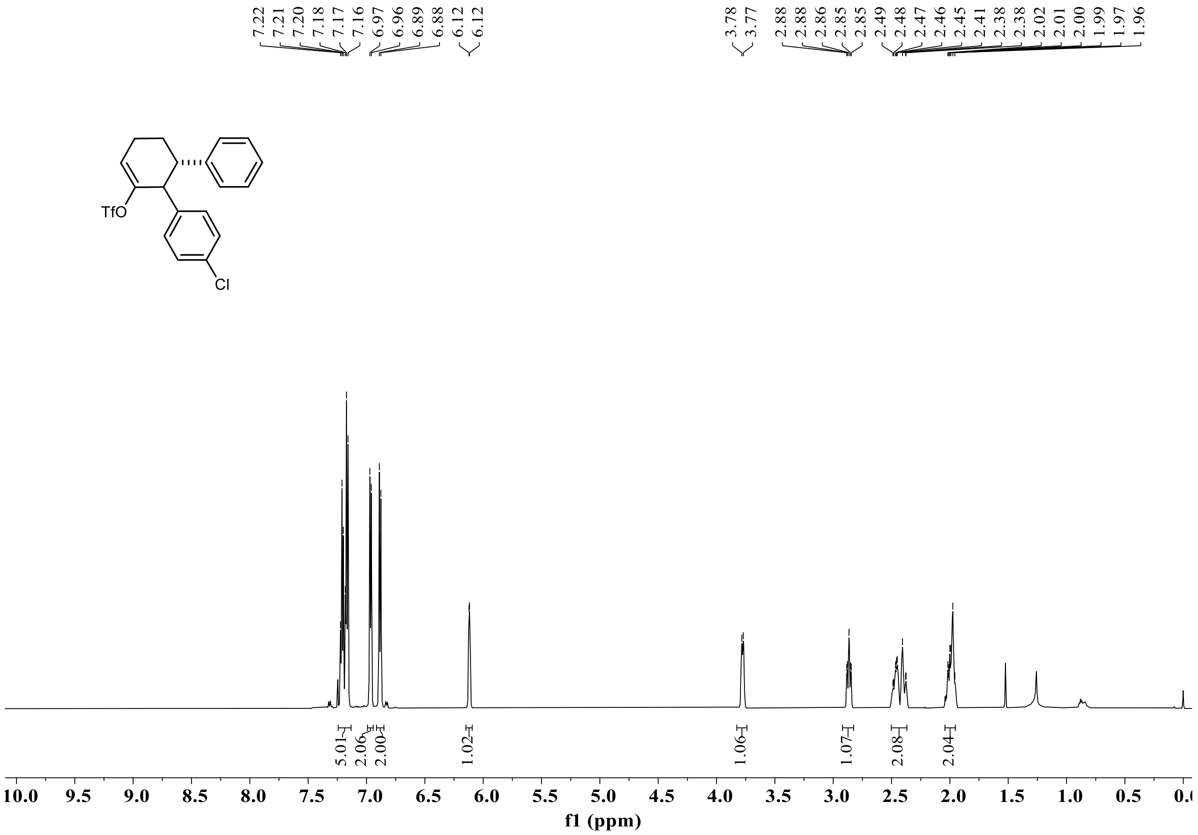

^{1}H NMR (600 MHz, CDCl$_3$)

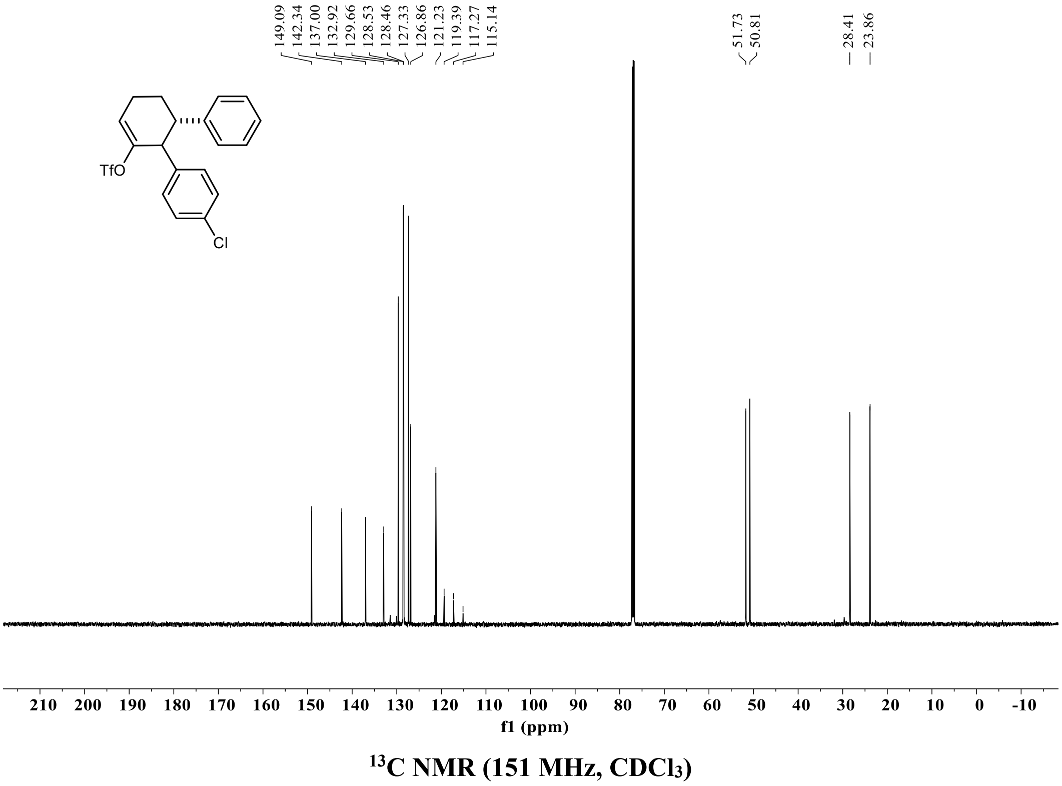

^{13}C NMR (151 MHz, CDCl$_3$)

Supplementary references

1 Duan, J., *et al*. Cross-electrophile C(sp^2)−Si coupling of vinyl chlorosilanes. *Angew. Chem. Int. Ed.* **59**, 23083-23088 (2020).

2 Wu, Z., Xu, X., Wang, J. & Dong, G. Carbonyl 1,2-transposition through triflate-mediated α-amination. *Science* **374**, 734-740 (2021).

3 Tao, X., *et al*. Alkenylation and Arylation of peptides via Ni-catalyzed reductive coupling of *α*-C-tosyl peptides with Csp^2 triflates/halides. *Org. Lett.* **23**, 7418-7422 (2021).

4 Armstrong, R. J., Niwetmarin, W. & Aggarwal, V. K. Synthesis of functionalized alkenes by a transition-metal-free zweifel coupling. *Org. Lett.* **19**, 2762-2765 (2017).

5 Kuwabe, S.-i., Torraca, K. E. & Buchwald, S. L. Palladium-catalyzed intramolecular C−O bond formation. *J. Am. Chem. Soc.* **123**, 12202-12206 (2001).
